# Proton pump inhibitors in invasively ventilated patients with SARS-CoV-2: a substudy of the re-evaluating the inhibition of stress erosions trial

**DOI:** 10.1136/bmjopen-2026-119937

**Published:** 2026-08-31

**Authors:** Brittany Dennis, Diane Heels-Ansdell, Quazi Ibrahim, John Basmaji, Lehana Thabane, Gordon Guyatt, Lois Saunders, Lori Hand, Olivia Loy, Nicole Zytaruk, Miranda Hardie, Adam M Deane, John C Marshall, Yaseen Arabi, François Lauzier, Joanna C Dionne, Karen EA Burns, Waleed Alhazzani, Gloria Vazquez-Grande, Anna Geagea, John Myburgh, John Muscedere, Shane English, Alexandra Binnie, Jennifer LY Tsang, Miranda Hunt, Marlies Ostermann, Abdulrahman A Al-Fares, Serena Knowles, Aijaz Ahmed, Deborah J Cook

**Affiliations:** 1 Department of Medicine, The University of British Columbia, Vancouver, British Columbia, Canada; 2 Department of Health Research Methods, Evidence and Impact, McMaster University, Hamilton, Ontario, Canada; 3 Department of Medicine, Division of Critical Care, Western University, London, Ontario, Canada; 4 St. Joseph’s Research Institute, Hamilton, Ontario, Canada; 5 Biostatistics Unit, St Joseph’s Healthcare Hamilton, Hamilton, Ontario, Canada; 6 Medicine, McMaster University, Hamilton, Ontario, Canada; 7 Department of Critical Care, Melbourne Medical School, The University of Melbourne, Melbourne, Victoria, Australia; 8 Critical Care Program, The George Institute for Global Health, Sydney, New South Wales, Australia; 9 Intensive Care Unit, Department of Critical Care Medicine, The University of Melbourne - Parkville Campus, Melbourne, Victoria, Australia; 10 Interdepartmental Division of Critical Care, University of Toronto, Toronto, Ontario, Canada; 11 Intensive Care Department, Ministry of National Guard Health Affairs, Riyadh, Saudi Arabia; 12 King Saud bin Abdulaziz University for Health Sciences, Riyadh, Saudi Arabia; 13 King Abdullah International Medical Research Center, Riyadh, Saudi Arabia; 14 Departments of Anesthesiology, Medicine and Critical Care Medicine, Laval University, Québec City, Quebec, Canada; 15 Critical Care and Internal Medicine Department, College of Medicine, Imam Abdulrahman Bin Faisal University, Dammam, Saudi Arabia; 16 Research and Innovation Institute, Ministry of Defense Health Services, Riyadh, Saudi Arabia; 17 Department of Critical Care, University of Manitoba, Winnipeg, Manitoba, Canada; 18 Critical Care, North York General Hospital, Toronto, Ontario, Canada; 19 Faculty of Medicine, University of New South Wales, Sydney, New South Wales, Australia; 20 Intensive Care Unit, Saint George Hospital, Kogarah, New South Wales, Australia; 21 Critical Care Medicine, Queen’s University, Kingston, Ontario, Canada; 22 Department of Medicine, University of Ottawa, Ottawa, Ontario, Canada; 23 Clinical Epidemiology Program, Ottawa Hospital Research Institute, Ottawa, Ontario, Canada; 24 Department of Critical Care Medicine, William Osler Health System, Brampton, Ontario, Canada; 25 Department of Critical Care Medicine, Niagara Health System, St Catharines, Ontario, Canada; 26 Department of Critical Care, King's College London, Guy's & St Thomas’ Hospital, London, UK; 27 Department of Anesthesia, Critical Care, Pain Medicine and Kuwait Extracorporeal Life Support, Al-Amiri Hospital, Kuwait City, Kuwait; 28 Department of Gastroenterology & Hepatology, Stanford University, Stanford, California, US

**Keywords:** COVID-19, Adult intensive & critical care, Research Design

## Abstract

**Objectives:**

Observational studies suggest that acid suppression may worsen outcomes among patients infected with SARS-CoV-2. The objectives of this embedded substudy of a randomised controlled trial evaluating pantoprazole in mechanically ventilated patients were to (1) describe the clinical characteristics of critically ill patients with SARS-CoV-2, (2) compare clinical outcomes with a propensity-matched non-infected cohort and (3) assess whether pantoprazole’s treatment effects differed by SARS-CoV-2 infection status.

**Design:**

A pre-planned substudy of the re-evaluating the inhibition of stress erosions (REVISE) trial, including a propensity-matched analysis of infected and non-infected patients comparing the effect of pantoprazole between patients with and without SARS-CoV-2.

**Setting:**

68 intensive care units (ICUs) in eight countries.

**Participants:**

From July 2019 to October 2023, 4821 eligible participants were enrolled in REVISE whether or not they had SARS-CoV-2 infection.

**Primary and secondary outcome measures:**

Participants enrolled in REVISE with SARS-CoV-2 infection had additional data collection, including biomarkers, venous thromboembolism, SARS-CoV-2 therapies and tracheostomy timing. The primary outcomes were clinically important upper gastrointestinal bleeding and 90-day mortality. Secondary outcomes included ventilator-associated pneumonia, *Clostridioides difficile* infection, patient-important upper GI bleeding, renal replacement therapy, ICU and hospital mortality and duration of mechanical ventilation, ICU and hospital stay.

**Results:**

Of the eligible trial cohort, 11.9% (540/4550) had SARS-CoV-2; 532 patients had additional SARS-CoV-2-specific data collection. Of these 532 patients, 87.8% received COVID-19-directed treatments—(dexamethasone 75.2%), 11.7% developed pulmonary embolism and 9.2% developed deep-vein thrombosis. After propensity matching, SARS-CoV-2 infection was not associated with clinically important upper gastrointestinal bleeding (adjusted HR 0.78, 95% CI 0.40 to 1.50) but was associated with significantly higher ICU, hospital and 90-day mortality, as well as longer duration of ventilation and ICU and hospital length of stay. The effect of pantoprazole on clinically important upper GI bleeding and 90-day mortality was consistent regardless of SARS-CoV-2 status.

**Conclusions:**

SARS-CoV-2 infection was associated with higher mortality and longer duration of mechanical ventilation, ICU and hospital stays, without an increased risk of clinically important upper gastrointestinal bleeding. Pantoprazole reduced clinically important upper gastrointestinal bleeding without adversely affecting other outcomes.

**Trial registration number:**

REVISE trial (NCT03374800), SARS-CoV-2 cohort study (NCT05715567).

**STRENGTHS AND LIMITATIONS OF THIS STUDY:**

- This SARS-CoV-2 cohort substudy was embedded in a large randomised trial, registered and guided by a published protocol and statistical analysis plan with pre-specified hypotheses.
- This substudy offers the first randomised trial data on the effect of proton pump inhibitors among patients with SARS-CoV-2 on a range of clinical outcomes.
- We lack information about SARS-CoV-2 variants and SARS-CoV-2 vaccine timing.
- Our definition of COVID-19 is limited to patients who received COVID-19-specific treatment; other infected patients may not have been symptomatic.
- The propensity analysis could not adjust for all possible factors predisposing to SARS-CoV-2, leading to possible unrecognised confounding.

## Introduction

Early in the pandemic, proton pump inhibitors (PPIs) were hypothesised to influence the susceptibility to SARS-CoV-2 and result in worse clinical outcomes among infected patients, although findings were inconsistent. Among older adults, early observational data suggested that recent exposure to PPIs was associated with a reduced risk of infection.^1^ However, several retrospective studies subsequently raised concern that antacid use among SARS-CoV-2-infected patients may worsen clinical outcomes.^2–4^ Across large claims-based and hospital-based retrospective cohorts, PPI use was linked to a higher risk of severe COVID-19, intensive care unit (ICU) admission, mechanical ventilation and death, although some associations were attenuated after adjustment for age and comorbidities.^2 3^ Consistent with these signals, a pooled analysis of retrospective studies also suggested an increased risk of mortality associated with COVID-19 among PPI users,^4^ as well as a more severe course of infection.^5^

To reduce the risk of upper gastrointestinal (GI) bleeding, clinicians often administer PPIs prophylactically in critically ill patients.^6^ During the COVID-19 pandemic, international guidance for the management of these patients supported the use of PPIs or histamine-2-receptor antagonists (H2RAs) in high-risk patients,^7^ yet sepsis guidance did not directly address stress ulcer prophylaxis,^8^ and in the early pandemic period, randomised trials had not yet evaluated whether PPIs affected clinical outcomes for ICU patients with COVID-19. Given unclear guidelines for COVID-19 and practices during the pandemic that could have increased bleeding risk (eg, anticoagulation^9^ and high-dose corticosteroids^10^) and an apparent increased risk of ventilator-associated pneumonia,^11^ we conducted a planned substudy within the re-evaluating the inhibition of stress erosions (REVISE) trial to evaluate the association between PPI exposure and clinically important outcomes in ICU patients tested positive for SARS-CoV-2.^12^

This prospective substudy was pre-planned and nested within an international randomised blinded trial in critically ill patients, investigating the effect of pantoprazole 40 mg intravenously compared with placebo on the primary *efficacy* outcome of clinically important upper GI bleeding and the primary *safety* outcome of 90-day mortality. REVISE found that pantoprazole significantly lowered the risk of clinically important and patient-important upper GI bleeding in invasively ventilated patients but had no effect on other outcomes, including 90-day mortality, ventilator-associated pneumonia, *Clostridioides difficile (C. difficil*e) infection, renal replacement therapy or mortality at ICU and hospital discharge.^13^ While testing positive for SARS-CoV-2 during hospital admission did not influence treatment effect for the primary outcome,^13^ the effect of pantoprazole in patients with COVID-19 requiring treatment has not been determined on ventilator-associated pneumonia, *C. difficile* infection or other outcomes in patients.

As SARS-CoV-2 is now endemic and PPIs are commonly prescribed in the ICU, understanding the relationship between PPI exposure and clinical outcomes in this population warrants investigation. Accordingly, within this multi-centre SARS-CoV-2 substudy of the REVISE trial (NCT05715567),^12^ we (1) describe the clinical characteristics and hospital admission trajectory of critically ill patients tested positive for SARS-CoV-2, (2) compare clinical outcomes with a propensity-matched non-infected cohort and (3) assess whether pantoprazole’s treatment effects differed by COVID-19 status.

## Methods

### Nested cohort study design

REVISE was registered (NCT03374800), and the trial design,^14^ statistical analysis plan^15^ and pandemic adaptations^16^ were previously published, as designed by the Canadian Critical Care Trials Group and the Australian and New Zealand Intensive Care Society Clinical Trials Group. Overall, 4821 patients undergoing invasive mechanical ventilation were enrolled in 68 ICUs in Canada, Australia, Saudi Arabia, the UK, the US, Kuwait, Pakistan and Brazil from July 2019 to October 2023.

This multi-centre cohort design was registered (NCT05715567), funded and guided by a protocol and statistical analysis plan.^12^ (online supplemental material 1) We included patients in this substudy if they were enrolled in REVISE on or after 11 March 2020. Notwithstanding the operational constraints imposed by the SARS-CoV-2 pandemic on ICU-based research,^17^ REVISE enrolment experienced only temporary recruitment suspensions at participating centres and did not impact eligibility criteria: patients both with and without SARS-CoV-2 were included.^16^ The substudy protocol outlined the objectives, a priori hypotheses and outcomes,^12^ summarised below.

10.1136/bmjopen-2026-119937.supp1 Supplementary data

#### Trial intervention

A study drug (40 mg intravenous pantoprazole or matching placebo) was prescribed in the ICU until discontinuation of invasive mechanical ventilation, the occurrence of a prespecified indication or contraindication to PPI use or death, up to 90 days.

#### Outcomes

The clinical outcomes for REVISE and this substudy were outlined in the trial registration (NCT03374800), trial protocol,^14^ trial statistical analysis plan,^15^ trial report^13^ and protocol for this substudy.^12^

#### Characterising patients with COVID-19

Briefly, patients were eligible for this substudy according to the inclusion and exclusion criteria of REVISE.^13^ Inclusion in the SARS-CoV-2 cohort was based on SARS-CoV-2 viral nucleic acid test positivity by PCR from a nasopharyngeal swab, oropharyngeal swab, sputum, endotracheal aspirate or bronchoalveolar lavage sample, as clinically determined rather than per protocol.^12^ Patients were eligible if they tested positive for SARS-CoV-2 in hospital (whether contracted preceding the index ICU admission, pre-hospital or in-hospital).

Patients were eligible for this substudy if they tested positive. These patients contributed to Objective 1. However, only patients receiving COVID-19-specific treatment were considered symptomatic for SARS-CoV-2 and were included in analyses pertaining to Objectives 2 and 3.

No additional tests or treatments were protocolised, and care was at the discretion of the treating team.

Additional data collection on SARS-CoV-2 positive patients included biomarkers, venous thromboembolic events, tracheostomy occurrence and timing and COVID-19 therapies. In the ICU, detailed daily data collection occurred. On the ward thereafter, *C. difficile* infection, vital status and hospital length of stay were captured. Mortality was documented at 90 days.

#### Covariates for matching

The patients’ age was abstracted from the medical record in years. Biological sex was abstracted from the medical record; gender was not considered. Pre-hospital acid suppression and use of corticosteroids in the week pre-randomisation were documented by research personnel from the patient’s medical record and clarified with a hospital or community pharmacist or substitute decision-maker if necessary. The ICU admission category of medical versus surgical/trauma was classified by research personnel based on the APACHE III admission diagnostic categories. Days from the start of the pandemic to enrolment were calculated from 11 March 2020.

### Analysis

As per the substudy statistical analysis plan,^12^ to characterise the cohort of patients tested positive for SARS-CoV-2 within the REVISE trial (*Objective 1*), we used descriptive statistics. Continuous variables were analysed and presented using mean±SD or median (IQR) for non-normally distributed or skewed data. Categorical variables are presented using frequencies and percentages.

We examined associations between SARS-CoV-2 status and clinical outcomes after adjusting for age, sex, pre-hospital acid suppression, ICU admission category (medical or surgical/trauma), corticosteroids 1 week pre-randomisation and days from the start of the pandemic to enrolment (*Objective 2*). Propensity matching only considered patients enrolled after or on 11 March 2020.

We implemented two methods to address confounding: (1) multivariable regressions and (2) an optimal propensity score matching (PSM) of a 2:1 ratio of patients with non-COVID-19 to patients with COVID-19. The score was the probability of COVID-19 predicted by the confounders. This method identified the set of matched triplets that minimised the overall difference in propensity scores across all possible matching combinations. We used optimal propensity matching at a 2:1 ratio, matching two patients with non-COVID-19 to each COVID-19 patient. This method identified the set of matched triplets that minimised the overall difference in propensity scores across all possible matching combinations. While multivariable regression was not originally proposed in the protocol, we added it to evaluate the adjusted association between COVID-19 and clinical outcomes while preserving sample size lost from matching. Using Cox-proportional hazard (PH) regressions, we studied the association between COVID-19 and clinically important upper GI bleeds, incident *C. difficile*, new renal replacement therapy, patient-important upper GI bleeding, ventilator-associated pneumonia and mortality in the ICU and hospital and within 90 days. Ventilator-associated pneumonia was classified in this substudy using the Clinical Pulmonary Infection Score as in the main trial report. The PH assumption was checked using the interaction of an explanatory variable with log (time). In case the PH assumption was violated for an explanatory variable, an interaction of that variable with log time was considered in the model, and relative effects and HRs were reported at different timepoints. ICU and hospital length of stay and duration of mechanical ventilation were highly skewed to the right. Thus, natural logarithms of these three variables were regressed on COVID-19 status and a priori confounders, and the adjusted (exponentiated) relative effects of COVID-19 on length of stays were reported. For propensity-matching analysis, patients within a matched triplet were correlated. To account for correlation in the time-to-event analyses, a robust sandwich-type variance estimator for clusters was used. For the length-of-stay outcomes, to account for within-matched triplet (cluster) correlations, random-intercept models were used in multivariable linear regressions. All analyses were adjusted for randomised allocation; mortality was also adjusted for APACHE II score. A sensitivity analysis was originally planned to evaluate only pre-hospital PPI use (not H2RAs); however, this was not performed, as less than 1% of patients used H2RAs.^13^

Responding to unexpected findings of a lower risk of ventilator-associated pneumonia among patients with COVID-19 compared with those without COVID-19, exploratory post hoc analyses further evaluated the association between SARS-CoV-2 status and ventilator-associated pneumonia. Both the propensity-matched analysis and the multivariable regression models for the outcome of ventilator-associated pneumonia were revised, adjusting for baseline pneumonia at ICU admission, alongside prespecified confounders and randomised assignment (pantoprazole vs placebo) included in the original models. A second post hoc analysis to evaluate the association between COVID-19 status and ventilator-associated pneumonia was repeated after excluding patients with baseline pneumonia. This involved using both propensity-matching and multivariable regression methods. Propensity scores were re-estimated in the reduced cohort, and prespecified models were refit. Additional exploratory post hoc analyses evaluated the association between COVID-19 status and clinically important upper GI bleeds, incident *C. difficile*, new renal replacement therapy, patient-important upper GI bleeding and ventilator-associated pneumonia, accounting for the competing risk of death.

Finally, we examined whether pantoprazole had a differential effect on clinically important upper GI bleeding or 90-day mortality among patients with COVID-19 compared with those without (*Objective 3*), using a prespecified subgroup analysis of REVISE.^15^ Subgroup effects were also evaluated for secondary trial outcomes of ventilator-associated pneumonia, *C. difficile* infection, patient-important upper GI bleeding, renal replacement therapy, ICU and hospital mortality and duration of mechanical ventilation, ICU stay and hospital stay. For time-to-event outcomes, subgroup analyses were conducted using Cox PH models that included COVID-19 status, randomised treatment and an interaction term between SARS-CoV-2 status and treatment. Analyses followed the intention-to-treat principle, with separate models fitted for each outcome. Models were adjusted for pre-hospital acid suppression use (either PPI or H2RA; exposure to either was a stratification variable), and mortality models were additionally adjusted for APACHE II score. As per the REVISE trial statistical analysis plan, if any subgroup effects were identified, using the interaction term when p-values were <0.10, we planned to assess the credibility of subgroup effects, interpreted using ICEMAN.^18^ Results were reported as HRs with 95% CIs. For duration outcomes (mechanical ventilation, ICU stay and hospital stay), subgroup analyses were performed using linear regression models on the natural logarithms of the outcomes, including COVID-19 status, randomised treatment, their interaction term and pre-hospital acid suppression.

Given negligible missing data (<2%), we did not perform imputation.^13^ As specified in the protocol, we did not adjust for multiple comparison adjustments. Analyses were conducted using SAS V.9.4^19^ and STATA 15.1 (College Station, Texas).^19^

### Patient and public involvement

Members of the public who were substitute decision-makers for critically ill patients and sometimes critically ill patients themselves were involved in the informed consent process to participate in the parent REVISE trial. One outcome reported herein (patient important bleeding) was developed by ICU survivors and family members of survivors or decedents.^13^

## Results

**Figure 1:**
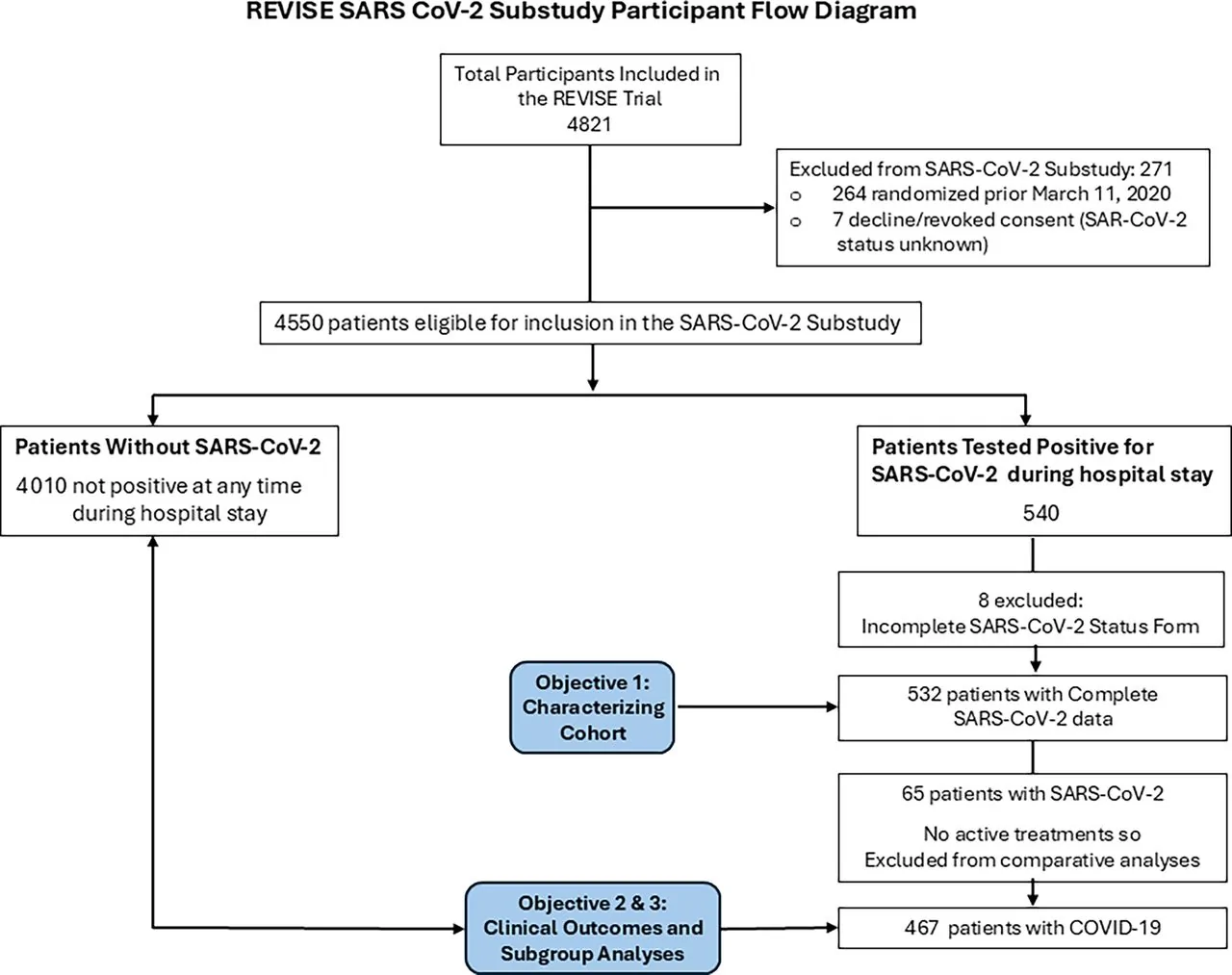
This figure summarises participant inclusion into the SARS-CoV-2 substudy, highlighting differences in sample size for each of the analyses addressing the three study objectives. REVISE, re-evaluating the inhibition of stress erosions.

Of 4821 enrolled patients in the trial, 264 participants were not eligible for this substudy due to pre-pandemic randomisation. An additional 7 patients were excluded due to declined or revoked consent or whose SARS-CoV-2 status remained unknown, resulting in a final cohort of 4550 patients. Within the remaining study cohort, 11.9% (540/4550) were tested positive for SARS-CoV-2 at any time during hospitalisation. The SARS-CoV-2-specific case report form was not completed in eight patients in one centre who were therefore excluded in analyses for Objective 1 examining treatments and other characteristics. There were an additional 65 patients for whom the SARS-CoV-2 form indicated no SARS-CoV-2-directed treatment. Because COVID-19 status could not be definitively classified for these 65 patients, they were included in Objective 1 analyses but excluded from comparative analyses (Objective 2: propensity-matched analyses and Objective 3: REVISE SARS-CoV-2 subgroup analyses). Accordingly, the final analytic sample for Objectives 2 and 3 comprised 4477 patients, including 4010 patients with non-SARS-CoV-2 and 467 patients with COVID-19 (figure 1).

### Characteristics of participants with SARS-CoV-2

Among the 532 patients with complete information on their SARS-CoV-2 status (ie, treatments received) enrolled in the REVISE trial, 268 were randomised to pantoprazole and 264 to placebo. Table 1 shows the characteristics of the overall SARS-CoV-2 cohort. Briefly, almost half of infected participants were unvaccinated (n=226, 42.5%). Treatments for COVID-19 were administered to most participants (n=467, 87.8%); dexamethasone was most commonly prescribed (n=400, 75.2%).

**Table 1:** Characterisation of REVISE patients who tested positive for SARS-CoV-2 during hospital stay

Tested positive SARS-CoV-2 at any time during hospital stay and part of the SARS-CoV-2 substudy*	Pantoprazole n=268	Placebo n=264	Total n=532
Vaccination status pre-ICU			
Not vaccinated	111 (41.4)	115 (43.6)	226 (42.5)
Unknown	52 (19.4)	60 (22.7)	112 (21.1)
One dose	21 (7.8)	12 (4.5)	33 (6.2)
Two doses	46 (17.2)	26 (9.8)	72 (13.5)
Three doses	38 (14.2)	51 (19.3)	89 (16.7)
CT positive for PE, number (%)			
Yes	28 (10.4)	34 (12.9)	62 (11.7)
No	94 (35.1)	115 (43.6)	209 (39.3)
Test not done	146 (54.5)	115 (43.6)	261 (49.1)
Ultrasound positive for one or more DVTs, number (%)			
Yes	24 (9.0)	25 (9.5)	49 (9.2)
No	61 (22.8)	55 (20.8)	116 (21.8)
Test not done	183 (68.3)	184 (69.7)	367 (69.0)
Positive for bowel ischaemia, number (%)			
Yes	3 (1.1)	5 (1.9)	8 (1.5)
No	53 (19.8)	58 (22.0)	111 (20.9)
Test not done	212 (79.1)	201 (76.1)	413 (77.6)
D Dimer level, mg/L (highest), median (IQR)	2460 (1020–5000) n=142	3485 (1250–6240) n=154	2855.5 (1180–5000) n=296
C-reactive protein level, mg/dL (highest), median (IQR)	148.0 (72.3–243.0) n=185	163.5 (89.8–252.0) n=199	156.1 (78.2–243.5) n=384
Ferritin level, ng/mL (highest), median (IQR)	905 (440–1717) n=113	866.5 (370–1935) n=124	883 (403–1870) n=237
Possible SARS-CoV-2-related treatments			
ACE2 renin–angiotensin Inhibitor	30 (11.2)	20 (7.6)	50 (9.4)
Azithromycin	114 (42.5)	117 (44.3)	231 (43.4)
Baricitinib (Janus-associated kinase (JAK) inhibitor)	23 (8.6)	18 (6.8)	41 (7.7)
*Casirivimab*	2 (0.7)	4 (1.5)	6 (1.1)
*Casirivimab* and imdevimab (*casirivimab* / imdevimab monoclonal antibody)	2 (0.7)	0 (0.0)	2 (0.4)
Convalescent plasma	0 (0.0)	0 (0.0)	0 (0.0)
Dexamethasone	201 (75.0)	199 (75.4)	400 (75.2)
Other high-dose steroid	32 (11.9)	46 (17.4)	78 (14.7)
Nirmatrelvir/ritonavir (*Paxlovid*)	3 (1.1)	2 (0.8)	5 (0.9)
Molnupiravir	2 (0.7)	1 (0.4)	3 (0.6)
Oseltamivir	3 (1.1)	6 (2.3)	9 (1.7)
Remdesivir	57 (21.3)	60 (22.7)	117 (22.0)
Ruxolitinib	0 (0.0)	1 (0.4)	1 (0.2)
Sarilumab	5 (1.9)	3 (1.1)	8 (1.5)
Sotrovimab (monoclonal antibody)	0 (0.0)	1 (0.4)	1 (0.2)
Statin	51 (19.0)	46 (17.4)	97 (18.2)
Tixagevimab/cilgavimab	0 (0.0)	1 (0.4)	1 (0.2)
Tocilizumab	77 (28.7)	76 (28.8)	153 (28.8)
Intravenous vitamin C	20 (7.5)	21 (8.0)	41 (7.7)
Other*	16 (6.0)	17 (6.4)	33 (6.2)
Extra-corporeal membrane oxygenation	2 (0.7)	7 (2.7)	9 (1.7)
Any of the above	237 (88.4)	230 (87.1)	467 (87.8)
None of the above	31 (11.6)	34 (12.9)	65 (12.2)
Tracheostomy, number (%)	37 (13.8)	47 (17.8)	84 (15.8)
Days from randomisation to tracheostomy, in those with a tracheostomy	n=37	n=47	n=84
Tracheostomy prior to day of randomisation	4 (10.8)	3 (6.4)	7 (8.3)
0–6 days	2 (5.4)	2 (4.3)	4 (4.8)
7–13 days	6 (16.2)	10 (21.3)	16 (19.0)
14–20 days	10 (27.0)	15 (31.9)	25 (29.8)
21–27 days	11 (29.7)	7 (14.9)	18 (21.4)
28–34 days	2 (5.4)	6 (12.8)	8 (9.5)
35 or more days	2 (5.4)	4 (8.5)	6 (7.1)
Days from randomisation to tracheostomy in those with no tracheostomy prior to day of randomisation	n=33	n=44	n=77
Mean (SD)	19.0 (8.9)	21.1 (16.2)	20.2 (13.5)
Median (IQR)	19 (14–22)	17.5 (12.5–24)	18 (13–23)
Total range	0-47	0-105	0-105

In this table, we describe the 532 patients with confirmed SARS-CoV-2 at any time during their hospital stay who are part of the comparative analyses for this substudy. However, there are eight SARS-CoV-2-positive patients from one centre which did not participate in the SARS-CoV-2 substudy.

*Other treatments include hydroxychloroquine, ivermectin, colchicine, acetylsalicylic acid, vitamin D, acyclovir, valacyclovir, zinc and rivaroxaban.

ACE2, angiotensin-converting enzyme-2; ASA, acetylsalicylic acid; DVT, deep vein thrombosis; ICU, intensive care unit; PE, pulmonary embolism; REVISE, re-evaluating the inhibition of stress erosions.

Complications of SARS-CoV-2 were common (eg, pulmonary embolism and deep vein thrombosis occurred in 62 (11.7%) and 49 (9.2%) participants, respectively). Biomarkers after enrolment captured in this cohort demonstrated high rates of inflammation, with median D-dimer levels at 2855.5 mg/L (IQR: 1180–5000), C-reactive protein of 156.1 mg/dL (IQR: 78.2–243.5) and ferritin of 883 ng/mL (IQR 403–1870).

### Comparison of participants with and without COVID-19

Clinical characteristics and ICU admission data for the COVID-19 cohort are summarised in table 2. Patients with COVID-19 were primarily admitted to the ICU for medical reasons (COVID-19: 440, 94.2% vs non-SARS-CoV-2: 2819, 70.3%) and most received corticosteroids in the week preceding randomisation (COVID-19: 362, 77.5% vs non-SARS-CoV-2: 1206, 30.1%). Evaluation of a priori selected variables used in the propensity score model demonstrated that baseline variables were significantly associated with COVID-19 status (online supplemental table S1). After propensity matching, patients with COVID-19 and without SARS-CoV-2 were well balanced across baseline demographic and clinical characteristics, as demonstrated in online supplemental table S2.

10.1136/bmjopen-2026-119937.supp2 Supplementary data

**Table 2:** Clinical characteristics of patients by COVID-19 status

	Patients not tested positive for SARS-CoV-2 n=4010	Patients with COVID-19 n=467	Total n=4477
Age in years, mean (SD)	57.9 (16.5)	61.4 (14.0)	58.2 (16.3)
APACHE II score, mean (SD)	21.8 (8.3)	20.9 (8.0)	21.7 (8.3)
Sex, number (%)			
Female	1439 (35.9)	187 (40.0)	1626 (36.3)
Male	2571 (64.1)	280 (60.0)	2851 (63.7)
Patient status, number (%)			
Medical	2819 (70.3)	440 (94.2)	3259 (72.8)
Surgical	564 (14.1)	15 (3.2)	579 (12.9)
Trauma	627 (15.6)	12 (2.6)	639 (14.3)
Admitting diagnostic category, number (%)			
Cardiovascular	441 (11.0)	23 (4.9)	464 (10.4)
Respiratory	1037 (25.9)	365 (78.2)	1402 (31.3)
Gastrointestinal	191 (4.8)	6 (1.3)	197 (4.4)
Neurologic	979 (24.4)	31 (6.6)	1010 (22.6)
Sepsis	338 (8.4)	14 (3.0)	352 (7.9)
Trauma	627 (15.6)	12 (2.6)	639 (14.3)
Metabolic	169 (4.2)	8 (1.7)	177 (4.0)
Renal	59 (1.5)	2 (0.4)	61 (1.4)
Other medical	61 (1.5)	4 (0.9)	65 (1.5)
Other surgical	108 (2.7)	2 (0.4)	110 (2.5)
Pre-hospital acid suppression, number (%)			
Yes	920 (22.9)	104 (22.3)	1024 (22.9)
No	3090 (77.1)	363 (77.7)	3453 (77.1)
Corticosteroids in the week pre-randomisation, number (%)	1206 (30.1)	362 (77.5)	1568 (35.0)
Study day 1, number (%)			
Invasive mechanical ventilation	4010 (100.0)	467 (100.0)	4477 (100.0)
Inotrope or vasopressor infusion	2825 (70.4)	343 (73.4)	3168 (70.8)
Renal replacement therapy	272 (6.8)	17 (3.6)	289 (6.5)

This table summarises the clinical characteristics and information on the critical admission for patients from the REVISE trial, with data presented disaggregated by SARS-CoV-2 status.

APACHE II score, Acute Physiology and Chronic Health Evaluation II Score; ICU, intensive care unit; REVISE, re-evaluating the inhibition of stress erosions.

No differences in the propensity-matched analysis were observed between patients with COVID-19 and those without SARS-CoV-2 for clinically important upper GI bleeding (adjusted HR (aHR): 0.78; 95% CI 0.40 to 1.50; p=0.45). However, patients with COVID-19 had a 73% higher hazard of mortality in the ICU at 30 days compared with non-SARS-CoV-2 propensity-matched patients (aHR: 1.73; 95% CI 1.06 to 2.40). At 60 days, this increased to a 150% higher hazard (aHR: 2.50; 95% CI 1.13 to 3.87) and further to 210% at 90 days (aHR: 3.10; 95% CI 1.10 to 5.10). Similarly, we observed higher mortality among patients with COVID-19, both during hospitalisation and throughout the 90-day follow-up (table 3).

**Table 3:** Associations between SARS-CoV-2 status and clinical outcomes

Outcomes	Confounders adjustment using propensity matching (2:1) (n=1401)		Confounders adjustment using multivariable regressions* (n=4477)	
	Adjusted hazard ratio (95% CI) SARS-CoV-2 (n=467) versus non-SARS-CoV-2 (n=934)	P value	Adjusted hazard ratio (95% CI) SARS-CoV-2 (n=467) versus non-SARS-CoV-2 (n=4010)	P value
Time to event outcome: no violation of proportional hazard assumption by SARS-CoV-2 status				
Clinically important upper GI bleed*	0.78 (0.40 to 1.50)	0.45	0.74 (0.41 to 1.34)	0.32
Incident C. difficile*	0.67 (0.21 to 2.10)	0.49	0.75 (0.25 to 2.23)	0.61
New renal replacement therapy*	1.24 (0.85 to 1.81)	0.26	1.25 (0.88 to 1.77)	0.21
Patient important upper GI bleed*	0.77 (0.43 to 1.38)	0.38	0.76 (0.45 to 1.31)	0.32
Ventilator-associated pneumonia*	0.50 (0.37 to 0.69)	< 0.001	0.47 (0.35 to 0.63)	< 0.001
Time-to-event outcome: violation of proportional hazard assumption by SARS-CoV-2 status				
ICU mortality† at				
30 days	1.73 (1.06 to 2.40)	< 0.001	1.47 (1.02 to 1.91)	< 0.001
60 days	2.50 (1.13 to 3.87)	< 0.001	2.07 (1.17 to 2.98)	< 0.001
90 days	3.10 (1.10 to 5.10)	0.002	2.54 (1.21 to 3.87)	< 0.001
Hospital mortality† at				
30 days	1.62 (1.17 to 2.07)	< 0.001	1.49 (1.14 to 1.84)	< 0.001
60 days	2.18 (1.33 to 3.03)	< 0.001	2.03 (1.35 to 2.70)	< 0.001
90 days	2.59 (1.39 to 3.79)	< 0.001	2.43 (1.46 to 3.39)	< 0.001
Mortality within 90 days†				
30 days	1.91 (1.45 to 2.37)	< 0.001	1.78 (1.41 to 2.16)	< 0.001
60 days	2.65 (1.76 to 3.55)	< 0.001	2.56 (1.79 to 3.32)	< 0.001
90 days	3.22 (1.93 to 4.50)	< 0.001	3.16 (2.01 to 4.30)	< 0.001

Linear regression models studying associations between length of stay outcomes and SARS-CoV-2 status				
	Adjusted ratio of duration (95% CI)‡	P value	Adjusted ratio of duration (95% CI)‡	P value
Outcomes				
Duration of ICU stay in days*§				
Exposure of interest: SARS-CoV-2 versus non-SARS-CoV-2	1.45 (1.33 to 1.58)	< 0.001	1.45 (1.35 to 1.57)	< 0.001
Duration of hospital stay in days*§				
Exposure of interest: SARS-CoV-2 versus non-SARS-CoV-2	1.30 (1.18 to 1.43)	< 0.001	1.35 (1.24 to 1.48)	< 0.001
Duration of mechanical ventilation in days*§				
Exposure of interest: SARS-CoV-2 versus non-SARS-CoV-2	1.69 (1.53 to 1.87)	< 0.001	1.66 (1.52 to 1.81)	< 0.001

In this table, we show the effect of SARS-CoV-2 status on clinical outcomes among mechanically ventilated patients admitted to the ICU. Cox proportional hazard regression models and linear regression models were used to study associations between SARS-CoV-2 status and clinical outcomes using two methods of confounder adjustment: (1) propensity matching (2:1) and (2) multivariable regression.

*Adjusted for patients’ age, sex, pre-hospital acid suppression, ICU admission category (medical or surgical/trauma), corticosteroids in the week pre-randomisation, days from the start of the pandemic to enrolment and randomisation group (pantoprazole vs placebo).

†Adjusted for variables listed in 1 and APACHE II score quartiles.

‡Exponentiation of the adjusted regression coefficient and its CIs are presented.

§Natural logarithm of length of stay was the dependent variable in the linear regressions.

*C. difficile*, *Clostridioides difficile*; GI, gastrointestinal; ICU, intensive care unit.

Other outcomes, including incident *C. difficile* infection, patient-important upper GI bleeding and new renal replacement therapy, were not associated with COVID-19 status (table 3). However, incident infection with VAP appeared to be lower among participants who had COVID-19 (aHR: 0.50; 95% CI 0.37 to 0.69).

Post hoc exploratory adjustment for baseline pneumonia diminished the initial apparent association between COVID-19 status and ventilator-associated pneumonia (online supplemental table S3) (aHR: 1.08; 95% CI 0.78 to 1.50). Further, no association between COVID-19 status and ventilator-associated pneumonia was observed after excluding patients with pneumonia at baseline from the propensity-matched analysis (online supplemental table S3). Competing risk analyses yielded similar results to those presented in table 3 and online supplemental table S3.

COVID-19 was associated with a 45% longer ICU stay (adjusted ratio of length of stay 1.45; 95% CI 1.33 to 1.58, p<0.001), a 30% longer hospital stay (adjusted ratio 1.30; 95% CI 1.18 to 1.43, p<0.001) and a 69% increase in the number of days of mechanical ventilation (adjusted ratio of duration 1.69; 95% CI 1.53 to 1.87, p<0.001)

Overall, results were consistent between multivariable regression models and the primary propensity-matched analyses (table 3, online supplemental tables S3 and S4).

### Substudy subgroup analyses

COVID-19 status in subgroup analyses did not suggest an effect modification of pantoprazole on the primary efficacy outcome of clinically important upper GI bleeding (non-SARS-CoV-2 HR: 0.30: 95% CI 0.18 to 0.49; COVID-19 HR: 0.27: 95% CI 0.08 to 0.95, interaction p-value: 0.868) or the primary safety outcome of 90-day mortality (non-SARS-CoV-2 HR: 0.97: 95% CI 0.86 to 1.09; COVID-19 HR: 0.94: 95% CI 0.69 to 1.27, interaction p-value: 0.855). Similarly, no differences were observed for other outcomes of patient-important upper GI bleeding, renal replacement therapy, VAP, *C. difficile* infection in hospital, ICU and hospital mortality, duration of mechanical ventilation, ICU stay or hospital length of stay. Table 4 summarises the results of all subgroup analyses.

**Table 4:** Efficacy of pantoprazole in patients with SARS-CoV-2 in REVISE: subgroup analyses

	Pantoprazole n (%)	Placebo n (%)	HR (95% CI)	P-value for the interaction term
Primary efficacy outcome (clinically important upper GI bleeding)				
Non-SARS-CoV-2	20/1983 (1.0)	67/1982 (3.4)	0.30 (0.18 to 0.49)	0.868
SARS-CoV-2	3/235 (1.3)	12/226 (5.3)	0.27 (0.08 to 0.95)	
Primary safety outcome (90-day mortality)				
Non-SARS-CoV-2	567/1987 (28.5)	583/1986 (29.4)	0.97 (0.86 to 1.09)*	0.855*
SARS-CoV-2	82/235 (34.9)	88/224 (39.3)	0.94 (0.69 to 1.27)*	
Incident ventilator-associated pneumonia (clinical pulmonary infection score≥6) in ICU				
Non-SARS-CoV-2	487/1990 (24.5)	495/1986 (24.9)	1.00 (0.88 to 1.13)	0.881
SARS-CoV-2	26/235 (11.1)	26/226 (11.5)	1.04 (0.61 to 1.80)	
Incident *C. difficile* infection in hospital				
Non-SARS-CoV-2	21/1983 (1.1)	16/1982 (0.8)	1.33 (0.69 to 2.55)	0.985
SARS-CoV-2	4/235 (1.7)	0/226 (0.0)	–	
New renal replacement therapy in ICU				
Non-SARS-CoV-2	110/1983 (5.5)	110/1985 (5.5)	1.01 (0.77 to 1.31)	0.308
SARS-CoV-2	26/235 (11.1)	20/226 (8.8)	1.41 (0.78 to 2.52)	
ICU mortality				
Non-SARS-CoV-2	392/1998 (19.6)	401/1997 (20.1)	1.00 (0.87 to 1.15)	0.944
SARS-CoV-2	60/236 (25.4)	67/227 (29.5)	1.01 (0.71 to 1.44)	
Hospital mortality				
Non-SARS-CoV-2	514/1996 (25.8)	536/1988 (27.0)	0.99 (0.87 to 1.11)	0.663
SARS-CoV-2	71/236 (30.1)	80/227 (35.2)	0.92 (0.66 to 1.26)	
Incident patient important upper GI bleeding				
Non-SARS-CoV-2	28/1983 (1.4)	81/1982 (4.1)	0.34 (0.22 to 0.53)	0.763
SARS-CoV-2	5/235 (2.1)	13/226 (5.8)	0.41 (0.15 to 1.15)	

	Pantoprazole median (IQR)	Placebo median (IQR)	Ratio of duration (95% CI)	P-value for the interaction term
Duration of mechanical ventilation in days				
Non-SARS-CoV-2	6 (3-11)	6 (3-11)	0.97 (0.92 to 1.03)	0.170
SARS-CoV-2	9 (5-16)	10 (6–19)	0.87 (0.74 to 1.01)	
Duration of ICU stay in days				
Non-SARS-CoV-2	9 (6-15)	9 (6-16)	0.98 (0.93 to 1.02)	0.129
SARS-CoV-2	13 (7–21)	14 (9–23)	0.87 (0.76 to 1.00)	
Duration of hospital stay in days				
Non-SARS-CoV-2	20 (11–35)	21 (11–37)	0.96 (0.91 to 1.02)	0.690
SARS-CoV-2	22 (15–40)	25 (14–43)	0.93 (0.79 to 1.09)	

In this table, we show the effect of pantoprazole versus placebo on clinical outcomes among patients with and without SARS-CoV-2.

*For 90-day mortality, the SARS-CoV-2 subgroup variable violates the assumption of proportional hazards. Therefore, the first Cox proportional hazards model includes only patients with ‘Non-SARS-CoV-2’, and a separate Cox proportional hazards model includes only patients with ‘SARS-CoV-2’. The two HRs are compared by performing a z-test on the log(HR). We did not apply criteria to assess subgroup credibility because all p-values were above 0.10, according to our statistical analysis plan.

*C. difficile*, *Clostridioides difficile*; GI, gastrointestinal; ICU, intensive care unit; REVISE, re-evaluating the inhibition of stress erosions.

## Discussion

Among 4550 patients in this stress ulcer prophylaxis trial enrolled after the beginning of the pandemic, 12% had tested positive for SARS-CoV-2, most of whom received directed therapies and were deemed to have COVID-19. These patients frequently developed thrombotic complications, and biomarkers demonstrated an intense inflammatory burden. After propensity matching with patients not tested positive for SARS-CoV-2, COVID-19 infection was not associated with clinically important upper GI bleeding but was associated with higher ICU, hospital and 90-day mortality and longer duration of mechanical ventilation and ICU and hospital length of stay. The effect of pantoprazole reducing clinically important upper GI bleeding with no effect on 90-day mortality was consistent irrespective of COVID-19 status. Overall, we found no evidence that SARS-CoV-2 modified the treatment effect of pantoprazole on any outcome.

This study evaluated several outcomes in patients with COVID-19 that have not been well studied, including *C. difficile* infection, patient-important upper GI bleeding and new renal replacement therapy. Our initial analyses suggested that COVID-19 was associated with a lower risk of ventilator-associated pneumonia, inconsistent with previous literature.^11 20–22^ In fact, most studies characterising patients with COVID-19 found a high rate of secondary pneumonia with an incidence ranging between 18 and 39/1000 mechanically ventilated days.^20–22^ A high risk of ventilator-associated pneumonia was also observed in a systematic review by Ippolito *et al*, which found a pooled rate of 45.4% (95% CI 37.8 to 53.2%; 2611/5593 patients).^11^ The initial counterintuitive finding of COVID-19 being associated with a lower risk of ventilator-associated pneumonia prompted post hoc exploratory analyses, which suggested that the apparent association was driven by incomplete adjustment for baseline pneumonia—likely more common among patients with COVID-19. Another possible explanation may be difficulty ascertaining a new episode of ventilator-associated pneumonia in patients with COVID-19, which could have led to missed diagnoses and confounding that attenuated once baseline pneumonia was accounted for (or excluded), resulting in no independent association.

Limitations of this study include the lack of information about SARS-CoV-2 variants and SARS-CoV-2 vaccine timing. We also cannot exclude the possibility of selection bias in some centres given a lack of information about patients who were eligible for REVISE but who were not enrolled. Our definition of COVID-19 is limited to patients who received COVID-19-specific treatment; others may have been symptomatic. On the other hand, we assumed that those who received specific treatments for COVID-19 actually had COVID-19, whereas a patient could have received dexamethasone for upper respiratory tract oedema after dental abscess drainage and tested positive for SARS-CoV-2, without the virus necessarily causing symptoms. The REVISE trial was not designed a priori to study COVID-19, as the pandemic was declared after the trial had launched. Furthermore, no pre-specified sample size calculation was possible to discern an effect modification based on SARS-CoV-2 status. The CIs around the HRs for some results are relatively wide, suggesting uncertainty about the power to detect an interaction effect if one existed. Finally, our propensity analysis could not adjust for all possible factors predisposing to SARS-CoV-2, leading to possible unrecognised confounding.

Strengths of this study include a large and prospectively collected trial dataset. This SARS-CoV-2 cohort substudy was registered (NCT05715567), hypotheses were pre-specified for each objective and analysis was guided by a statistical analysis plan.^12^ The point estimates and CIs for the propensity-matching and multivariable regression analyses are similar, increasing our confidence in the inferences about these results. This substudy offers the first randomised trial data on the effect of PPIs among patients with SARS-CoV-2 on a range of clinical outcomes.

## Conclusion

In this preplanned substudy, COVID-19 infection was associated with higher mortality and longer ICU and hospital stays without an increased risk of clinically important upper GI bleeding in critically ill mechanically ventilated patients compared with patients who were not tested positive for SARS-CoV-2. Pantoprazole demonstrated similar effects on bleeding and safety outcomes regardless of COVID-19 status. Despite observational reports suggesting potential harm from acid suppression in patients with COVID-19, pantoprazole reduced clinically important and patient-important upper GI bleeding without affecting other outcomes, offering consistent benefit regardless of COVID-19 status, supporting its consideration for stress ulcer prophylaxis.

## Supplementary Material

Reviewer comments

Author's manuscript

## Data Availability

Data are available upon reasonable request. The REVISE dataset will be used for secondary observational studies such as this one, addressing additional hypothesis-driven questions. The study protocol and statistical analysis plan for this substudy are published. Access by REVISE investigators to the REVISE dataset will follow a submitted rationale, analysis plan and approval by the Management Committee. Requests for access to the data for this substudy and the parent REVISE trial can be sent by external academic investigators for investigator-initiated studies. These will be considered following a submitted rationale, analysis plan and approval by the REVISE Management Committee and research ethics boards as relevant. Requirements will be stipulated in a pre-specified data sharing agreement. If approved, only de-identified trial data and standard operating procedures will be provided with data dictionaries to achieve aims in the approved proposal, transferred via a secure web portal. Data are available now until 2030.
