# Supplementary material for: Proton pump inhibitors in invasively ventilated patients with SARS-CoV-2: a substudy of the re-evaluating the inhibition of stress erosions trial
Source: BMJ Open. 2026 Aug 31;16(8):e119937. doi: 10.1136/bmjopen-2026-119937 (PMC13536124; doi:10.1136/bmjopen-2026-119937)
Supplement: Supplementary file 1 [file bmjopen-16-8-s001.pdf]

Dennis *et al. Trials* (2023) 24:561
https://doi.org/10.1186/s13063-023-07589-2

Trials

STUDY PROTOCOL

Open Access

Proton pump inhibitors in critically ill mechanically ventilated patients with COVID-19: protocol for a substudy of the Re-EValuating the Inhibition of Stress Erosions (REVISE) Trial

Brittany B. Dennis[1], Lehana Thabane[2,3,4], Diane Heels-Ansdell[2], Joanna C. Dionne[5], Alexandra Binnie[6], Jennifer Tsang[1,6], Gordon Guyatt[5], Aijaz Ahmed[7], François Lauzier[8], Adam Deane[9], Yaseen Arabi[10,11,12], John Marshall[13], Nicole Zytaruk[2,4], Lois Saunders[4], Simon Finfer[14], John Myburgh[14,15], John Muscedere[16], Shane English[17,18], Marlies Ostermann[19], Miranda Hardie[14], Serena Knowles[14], Deborah Cook[4,5*] and For the REVISE Investigators the Canadian Critical Care Trials Group

Abstract

Background Critically ill patients commonly receive proton pump inhibitors (PPIs) to prevent gastrointestinal (GI) bleeding from stress-induced ulceration. Despite widespread use in the intensive care unit (ICU), observational data suggest that PPIs may be associated with adverse outcomes in patients with COVID-19 infection. This pre-planned study is nested within a large randomized trial evaluating pantoprazole versus placebo in invasively ventilated patients. The 3 objectives are as follows: (1) to describe the characteristics of patients with COVID-19 in terms of demographics, biomarkers, venous thromboembolism, tracheostomy incidence and timing, and other clinical outcomes; (2) to evaluate the impact of COVID-19 infection on clinically important GI bleeding, 90-day mortality, and other outcomes compared to a propensity-matched non-infected cohort; and (3) to explore whether pantoprazole has a differential treatment effect on clinically important GI bleeding, 90-day mortality, and other outcomes in patients with and without COVID-19 infection.

Methods The ongoing trial Re-EValuating the Inhibition of Stress Erosions (REVISE) compares pantoprazole 40 mg IV to placebo on the primary efficacy outcome of clinically important GI bleeding and the primary safety outcome of 90-day mortality. The protocol described in this report is for a substudy focused on patients with COVID-19 infection that was not in the original pre-pandemic trial protocol. We developed a one-page case report form to characterize these patients including data related to biomarkers, venous thromboembolism, COVID-19 therapies, tracheostomy incidence and timing, duration of mechanical ventilation, and ICU and hospital stay. Our analysis will describe the trajectory of patients with COVID-19 infection, a propensity-matched analysis of infected and non-infected patients, and an extended subgroup analysis comparing the effect of PPI among patients with and without COVID-19 infection.

*Correspondence:
Deborah Cook
debcook@mcmaster.ca
Full list of author information is available at the end of the article

© The Author(s) 2023. **Open Access** This article is licensed under a Creative Commons Attribution 4.0 International License, which permits use, sharing, adaptation, distribution and reproduction in any medium or format, as long as you give appropriate credit to the original author(s) and the source, provide a link to the Creative Commons licence, and indicate if changes were made. The images or other third party material in this article are included in the article's Creative Commons licence, unless indicated otherwise in a credit line to the material. If material is not included in the article's Creative Commons licence and your intended use is not permitted by statutory regulation or exceeds the permitted use, you will need to obtain permission directly from the copyright holder. To view a copy of this licence, visit http://creativecommons.org/licenses/by/4.0/. The Creative Commons Public Domain Dedication waiver (http://creativecommons.org/publicdomain/zero/1.0/) applies to the data made available in this article, unless otherwise stated in a credit line to the data.

Discussion Prophylactic acid suppression in invasively ventilated critically ill patients with COVID-19 infection has unknown consequences. The results of these investigations will inform practice, guidelines, and future research.

Trial registration REVISE Trial [NCT03374800 December 15, 2017], COVID-19 Cohort Study [NCT05715567 February 8, 2023].

Keywords Randomization, Critically ill, COVID-19 pandemic, Hospital transfers, Stress ulceration, Gastrointestinal bleeding, Ventilator-associated pneumonia

Introduction

For patients with COVID-19 receiving invasive mechanical ventilation, standard critical care therapies, including prophylactic acid suppression, have unclear consequences. Concerns emerged early in the pandemic that some common medications may predispose to COVID-19 infection or worsen outcomes among infected patients. For example, observational studies suggested that angiotensin-converting enzyme (ACE) inhibitors were harmful as they were associated with increased mortality [1, 2]. However, this finding was not supported in larger, more robust observational studies [3, 4] or randomized trials [5].

The possible impact of proton pump inhibitors (PPIs) on COVID-19 infection susceptibility and outcomes has also drawn attention. In one observational study from the UK Biobank cohort, PPIs were associated with a 23% reduction in susceptibility to COVID-19 infection (odds ratio [OR] at 1 year = 0.77, 95% confidence interval [CI]: 0.71–0.83), with the greatest protective effects observed with recent use of PPIs (within 6 months) and in people at least 70 years of age [6]. However, these findings of benefit were in contrast with other studies suggesting associated harm. In a retrospective study of Korean national insurance claims including 132,316 patients, short-term use of PPIs was associated with a 79% increased risk of a composite outcome of admission to the ICU, use of mechanical ventilation, and death [7]. These findings were limited by low event rates, reliance on registry data to ascertain PPI exposure, and the use of a composite outcome measure due to insufficient events. In another retrospective case review of 2164 patients with COVID-19 admitted to a US county hospital, PPI use was associated with a 2.8-fold increased risk for death (OR) [95% CI, 1.4–5.5], although findings were not statistically significant following adjustment for age, renal, cardiovascular, and pulmonary comorbidities [8]. Furthermore, a pooled analysis of six retrospective studies of 5884 COVID-19 patients also suggested that PPI use may be associated with an increased risk of death from COVID-19 (risk ratio 1.7, 95% CI 1.02–2.9, $I^2 = 66\%$) [9]. However, all of these study designs generate limited inferences for practice or research purposes.

PPIs are common medications among hospitalized patients, particularly in the intensive care unit (ICU). Whether used pre-hospital or not, critically ill mechanically ventilated patients are usually prescribed PPIs to prevent gastrointestinal (GI) bleeding from stress-induced ulceration [10]. The potential adverse effects of PPI use include ventilator-associated pneumonia (VAP) and *Clostridioides difficile* (*C. difficile*) infection—nosocomial infections that are together more common than GI bleeding and are associated with substantially greater morbidity, mortality, and costs [11]. Herein, we describe a protocol to examine the association between COVID-19 infection and outcomes and PPI use among invasively mechanically ventilated patients nested within an ongoing clinical trial testing pantoprazole versus placebo [12]. This ongoing trial is relevant for patients with COVID-19 infection, given their increased risk of VAP [13], treatment with high-dose corticosteroids [14], and varying anticoagulation intensities [15].

The three objectives of this nested study are as follows: (1) to describe the characteristics of patients with COVID-19 in terms of demographics, biomarkers, COVID-19 treatment, rates of venous thromboembolism, tracheostomy incidence and timing, and other outcomes (e.g., duration of mechanical ventilation, ICU and hospital stay, and mortality); (2) to evaluate the impact of COVID-19 on clinically important GI bleeding, 90-day mortality, infectious, and other outcomes compared to a propensity-matched non-COVID-19 cohort; and (3) to explore whether pantoprazole has a differential treatment effect on clinically important GI bleeding and 90-day mortality, in addition to VAP and *C. difficile* infection and other outcomes, in patients with and without COVID-19.

Methods

Project design for overall REVISE Trial

The Re-EValuating the Inhibition of Stress Erosions (REVISE) is an international randomized stratified, concealed, blinded, parallel-group trial in mechanically ventilated patients, investigating the effect of the PPI pantoprazole 40 mg IV compared to placebo, on the primary *efficacy* outcome of clinically important GI bleeding, the primary *safety* outcome of 90-day mortality, rates of VAP and *C. difficile* infection, patient-important

bleeding, use of renal replacement therapy, and hospital mortality [12]. Led by the Canadian Critical Care Trials Group and the Australian & New Zealand Intensive Care Society Clinical Trials Group, the REVISE Methods Centers are located at McMaster University in Hamilton for international centers and The George Institute in Sydney for Australia. Research Coordinators screen patients from Monday to Friday, using an *a priori* or a consent-to-continue model as locally approved, holding consent encounters with patients if able, or substitute decision-makers in-person or by telephone as needed. Randomization is conducted by research pharmacists to ensure double blinding. Methods for the REVISE trial have been described elsewhere [NCT03374800] [12].

Design for nested COVID cohort study

This study uses a multicenter cohort design embedded within a randomized trial [NCT05715567]. This is the first version (version 1.0; date: June 13, 2023) of the sub-study protocol. Figure 1 highlights how it is integrated into the stress ulcer prophylaxis research program. Patients with COVID-19 have been eligible for REVISE since March 11, 2020. Despite the difficulties of continuing non-COVID research in ICUs during the pandemic, recruitment to REVISE was paused for the shortest possible period in each participating center throughout the pandemic. The objectives, a priori hypotheses and outcomes for this COVID-19 study are outlined in Table 1. Although the protocol described in this work would be characterized as a study within a trial, we adhered to the Standard Protocol Items for Clinical Trials (SPIRIT) reporting guidelines where appropriate [16]. Accordingly, the recommended SPIRIT diagram was not included in this work, this will be published as part of the full REVISE randomized trial protocol.

Setting

Patients are being enrolled in 63 ICUs worldwide (39 Canada, 19 Australia, 1 Saudi Arabia, 1 the UK, 1 the US, 1 Kuwait, and 1 Pakistan).

Population

Patients are eligible for this COVID-19 substudy according to the inclusion and exclusion criteria of the REVISE Trial, as outlined in Table 2. Randomization is not stratified by COVID-19 disease status. The inclusion in the COVID-19 cohort will be based on COVID-19 viral nucleic acid test positivity by polymerase chain reaction (PCR) from a nasopharyngeal swab, oropharyngeal swab, sputum, endotracheal aspirate, or bronchoalveolar lavage sample, as clinically determined rather than per protocol. The COVID-19 classification in this study will be made via PCR testing, due to the heightened risk for misclassification using other forms of testing, which will not

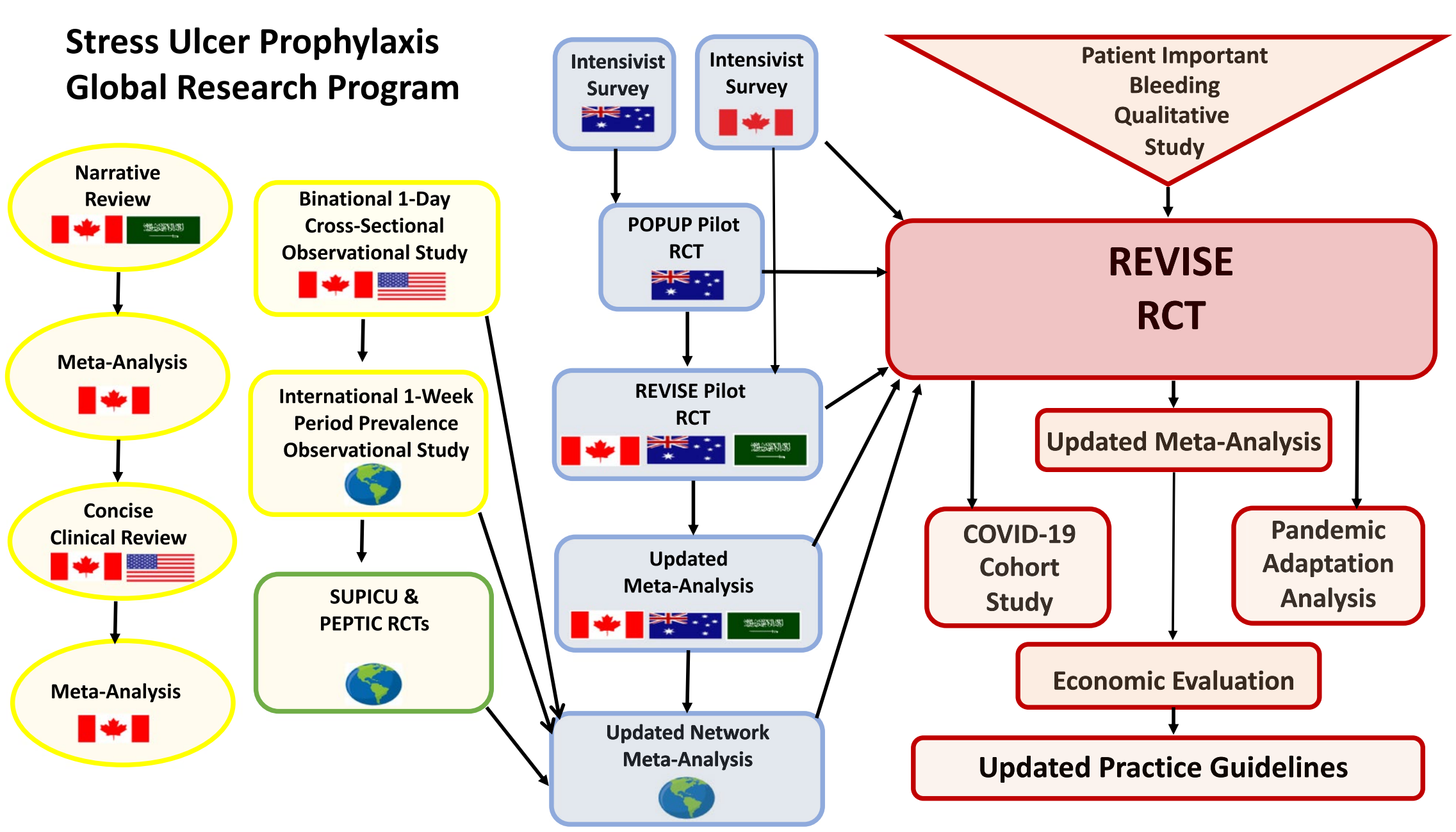

Fig. 1 COVID-19 cohort study within the stress ulcer prophylaxis research program. This figure displays how this COVID cohort study is integrated into the international stress ulcer prophylaxis research program

Table 1 Overview of research objectives, hypotheses, outcomes, and analytic methods

Objectives	Hypotheses	Outcomes	Methods of analysis
**Question 1:** What prognostically relevant demographic, biomarker, and clinical data characterize invasively ventilated patients with COVID-19? (Explanatory variable: not applicable; no comparisons)			
To establish a clinical profile of critically ill patients with COVID-19, incorporating demographics, biomarkers, rates of venous thromboembolism, COVID-19 therapies, duration of mechanical ventilation, ICU, hospital stay, and tracheostomy timing	Patients with COVID-19 will have a high burden of comorbidity and, as a consequence of their underlying severe viral illness, will exhibit high inflammatory biomarkers (D-dimer, CRP, ferritin), receive targeted COVID-19 therapies, experience long duration of mechanical ventilation, and undergo tracheostomy later than current norms	Description of demographics, biomarkers, and course in the ICU including venous thromboembolism rates and tracheostomy timing	Descriptive analysis<br>Continuous variables will be summarized using mean ± standard deviation (SD), while frequencies and percentages will be used to summarize categorical variables
**Question 2:** Do critically ill patients with COVID-19 have higher rates of clinically important GI bleeding, 90-day mortality, and infection compared to a propensity-matched non-COVID cohort? (explanatory variable: COVID-19)			
To understand the impact of COVID-19 on CIGIB, 90-day mortality, and rates of VAP, *C. difficile* infection, and patient-important bleeding; use of renal replacement therapy; and duration of mechanical ventilation, ICU stay and hospital stay, and ICU and hospital mortality compared to patients without COVID-19	Patients with COVID-19 will have a poorer prognosis than propensity-matched control patients without COVID-19. Patients with COVID-19 will have higher rates of CIGIB, 90-day mortality, VAP, *C. difficile* infection, and patient-important GI bleeding, use of renal replacement therapy, ICU and hospital mortality, and longer durations of mechanical ventilation and ICU and hospital stay, compared to a propensity-matched non-COVID cohort	CIGIB, 90-day mortality<br>VAP, *C. difficile* infection, patient-important bleeding, and renal replacement therapy<br>Duration of mechanical ventilation, ICU stay, and hospital stay<br>ICU and hospital mortality	Propensity-matched analysis of patients in REVISE with and without COVID-19 infection
**Question 3**: Do critically ill patients with COVID-19 projected to receive mechanical ventilation for > 48 h have different clinical outcomes with versus without PPIs? (explanatory variable: pantoprazole)			
To examine whether pantoprazole has a different treatment effect on CIGIB or 90-day mortality in patients with and without COVID-19 (REVISE subgroup analysis)<br>To determine whether pantoprazole has a differential treatment effect in patients with or without COVID-19 on VAP, *C. difficile* infection, patient-important GI bleeding, use of renal replacement therapy, hospital mortality, and duration of mechanical ventilation, ICU stay and hospital stay, and ICU and hospital mortality	We hypothesize no modification of the effect of pantoprazole on CIGIB. We hypothesize that pantoprazole is more harmful in patients with versus without SARS-CoV-2 in terms of increased risk of 90-day mortality<br>We hypothesize no modification of the effect of pantoprazole on VAP, *C. difficile* infection, patient-important GI bleeding, use of renal replacement therapy, and duration of mechanical ventilation, ICU stay and hospital stay, and ICU and hospital mortality. We hypothesize that pantoprazole is more harmful in patients with versus without SARS-CoV-2 in terms of increased risk of hospital mortality	CIGIB, 90-day mortality (REVISE subgroup analysis)<br>VAP, *C. difficile* infection, patient-important bleeding, and renal replacement therapy (extended outcomes)<br>Duration of mechanical ventilation, ICU stay and hospital stay, and ICU and hospital mortality (extended outcomes)	Subgroup analysis: Comparison of outcomes for patients with and without COVID-19 receiving either pantoprazole or placebo<br>Comparison of the time to the primary and secondary binary outcomes in the two groups using Cox proportional hazards regression with threshold *P*-values of 0.05, adjusted for the center and pre-hospital use of acid suppression (stratification variables). A test of interaction will be performed for this subgroup analysis in REVISE, and the results will be interpreted per the ICEMAN criteria<br>For binary outcomes, we will report hazard ratios with 95% CIs as well as the absolute risk increase or decrease and 95% CIs<br>For continuous outcomes, we will compare the two arms using a *t*-test on the log scale (to normalize the distribution) or a nonparametric approach if needed

Research question, objectives, hypotheses, outcomes, and analytic approaches for the COVID-19 REVISE substudy

CIGIB clinically important GI bleeding, *ICU* intensive care unit, *PPI* proton pump inhibitor, *COVID-19* coronavirus disease of 2019, *VAP* ventilator-associated pneumonia, *C. difficile Clostridioides difficile*

Table 2 Inclusion and exclusion criteria for REVISE

**Inclusion criterion**	
Adults ≥ 18 years old projected to receive invasive mechanical ventilation for ≥ 48 h according to the treating physician	
**Exclusion criteria**	
1. Already received invasive mechanical ventilation ≥ 72 h during this hospital admission	
2. Acid suppression for active GI bleeding or high risk of bleeding (e.g., current bleeding, peptic ulcer bleeding within 8 weeks, recent severe esophagitis, Barrett's esophagus, Zollinger-Ellison syndrome) [dyspepsia or gastroesophageal reflux is not an exclusion criterion]	
3. Acid suppression in the ICU for > 1 daily dose equivalent of a PPI or H2RA	
4. Dual antiplatelet therapy	
5. Combined antiplatelet and therapeutic anticoagulation	
6. Pantoprazole contraindication per local product information	
7. Palliative care or anticipated withdrawal of advanced life support	
8. Pregnancy	
9. Previous enrollment in REVISE, a related trial, or trial for which co-enrollment is prohibited	
10. Patient, proxy, or physician declines	

In this table, we list the eligibility criteria for this study, based on the inclusion and exclusion criteria for the REVISE trial

GI gastrointestinal, *ICU* intensive care unit, *PPI* proton pump inhibitor, *H2RA* histamine-2-receptor antagonists

be accepted for COVID-19 diagnosis, including antigen testing. Patients will be eligible for this substudy if they test positive for SARS-CoV-2 in the month preceding the index ICU admission, whether pre-hospital, in hospital, or in the ICU. Patients diagnosed as contracting SARS-CoV-2 more than 48 h after enrollment in REVISE will be ineligible.

Summary of outcomes for REVISE

The primary *efficacy* outcome for REVISE is clinically important GI bleeding occurring in the ICU or resulting in ICU readmission during the index hospital stay. Adjudicator agreement for this metric was excellent in two prior studies [17, 18] which was associated with increased attributable length of stay and mortality [19]. The definition of clinically important GI bleeding is overt GI bleeding (i.e., hematemesis, frank blood or coffee ground nasogastric aspirate, melena or hematochezia) *plus 1 of the following in the absence of other causes*: (1) hemodynamic change defined as a spontaneous decrease in invasively monitored mean arterial pressure or non-invasive systolic or diastolic blood pressure of ≥ 20 mmHg or an orthostatic increase in pulse rate of ≥ 20 beats/min and a decrease in systolic blood pressure of ≥ 10 mmHg, with or without vasopressor initiation or increase; (2) vasopressor initiation; (3) hemoglobin decrease of ≥ 2 g/dL (20 g/L) within 24 h of bleeding; (4) transfusion of ≥ 2 units red blood cells within 24 h of bleeding; or (5) therapeutic intervention (e.g., therapeutic endoscopy, angioemoblization, surgery). The primary *safety* outcome is mortality defined as all-cause mortality at 90 days after randomization to evaluate a suggestion of increased risk of death associated with pantoprazole among the sickest subgroup of patients in a recent placebo-controlled trial [20]. Secondary outcomes for REVISE are listed in Table 3.

Table 3 Secondary outcome definitions in REVISE

Ventilator-associated pneumonia	VAP is diagnosed in patients who received invasive mechanical ventilation for ≥ 48 h when there is a new, progressive, or persistent radiographic infiltrate *plus at least 2 of the following without other obvious cause*: (1) fever (temperature > 38 °C) or hypothermia (temperature < 36 °C), (2) leukopenia (< 4.0×10^6/L) or leukocytosis (> 12×10^6/L), (3) purulent sputum, or (4) gas exchange deterioration. Given no universally accepted VAP criteria, data collection and central adjudication allows analysis of several other VAP definitions. We do not collect infection-related ventilator-associated conditions as they are modifiable by ventilator settings and volume status
C. difficile infection	Defined as clinical features (diarrhea [≥ 3 episodes of unformed stools or Bristol type 6 or 7], ileus, or toxic megacolon) and either microbiological evidence of toxin-producing *C. difficile* or pseudomembranous colitis on colonoscopy in hospital
Patient-important GI bleeding	Defined as overt GI bleeding, *plus* an invasive intervention (e.g., therapeutic endoscopy, angioembolization, surgery), acknowledging how some clinically important GI bleeds in prior studies did not actually require any tests or treatments, and thus may not be important to patients, to be refined following completion of patient and family interviews from a Patient Important Bleeding Study [NCT05506150]
Renal replacement therapy	Defined as the initiation of new renal replacement therapy in the ICU
Hospital mortality	Defined as all-cause mortality in the hospital

In this table, we show the secondary outcomes and their definitions for the REVISE trial

VAP ventilator-associated pneumonia, *ICU* intensive care unit, *GI* gastrointestinal, *C. difficile Clostridioides difficile*

Pilot work

Investigators developed a one-page draft case report form to capture new information which was not part of the original REVISE trial to better characterize the COVID-19 cohort. A group of eight research coordinators reviewed and refined the form to capture COVID-19 vaccination status, biomarkers, venous thromboembolic events, tracheostomy occurrence and timing, and COVID-19 therapies as per Table 4. The form was pre-tested on 15 patients by abstracting data from either electronic or paper-based medical records. Accessing the chart, scanning contents, extracting relevant information, and entering data into the password-protected REVISE website required an estimated 40 min per patient. After this pilot exercise, we oriented research coordinators in participating centers to the study objectives, methods, and the case report form during telephone, videoconference, and in-person investigators' meetings.

Data collection and outcomes for the COVID-19 cohort study

Retrospective data collection for newly added variables for the COVID-19 study was required for participants enrolled prior to the implementation of this COVID-19 study protocol. Following relevant ethics review, COVID-19-specific variables were incorporated into existing data-collection forms, affording prospective data collection for COVID-19 variables for the remainder of the study. For any patients transferred to non-REVISE centers during the pandemic, all efforts are being made to complete the COVID-19 case report form, including a collection of data on outcomes occurring after transfer to a non-REVISE center.

Biologic specimens (e.g., sputum culture for COVID-19 testing) and laboratory results (blood samples to evaluate for hemoglobin levels) are collected as part of routine medical care. These samples are not being procured or stored for study purposes.

Data management and quality control

Data management and quality control will be overseen by the REVISE Methods Centers, located at McMaster University in Hamilton and The George Institute (TGI) in Sydney. These centers are responsible for ensuring optimal trial conduct. Oversight and quality control will be achieved through close collaboration between these centers, who hold twice monthly teleconferences to harmonize approaches and share management efficiencies. The Methods Center Leads and relevant investigators agreed to train local principal investigator(s), research coordinator(s), and research pharmacist(s), on the protocol. Throughout the trial, the methods center staff will engage in compliance-enhancing strategies, set targets, and track progress. REVISE analyst Ms. D Heels-Ansdell will conduct periodic central statistical monitoring to generate overall and site-specific metrics for internal use and site feedback. The methods center staff will adopt a risk-adapted approach to monitor each site (in-person or by videoconference per pandemic adaptations) to assess and enhance protocol fidelity. The foci will include eligibility adherence, stratification, informed consent documentation, regulatory compliance, and case report form accuracy.

In the event of any modifications made to the existing protocol for the COVID-19 substudy, the REVISE methods center will take responsibility for notifying all participating centers, sponsors, and funding institutions. All participating centers would receive a copy of the revised protocol and meet to review and discuss any implications of such changes to study implementation.

Sample size

The cohort size is based on the number of patients with COVID-19 enrolled in REVISE. As of June 1, 2023, 484 patients with COVID-19 have been enrolled in REVISE, representing 10.1% of the sample size of 4800 patients. Acknowledging the inherent challenges in predicting the incidence of and the means ± standard deviation (SD)

Table 4 COVID cohort substudy biomarkers and other clinical characteristics

New variables	Definitions and measures
**D-dimer**	Highest D-dimer level (μg/L) recorded post-COVID diagnosis
**C-reactive protein**	Highest C-reactive protein level (mg/L) recorded post-COVID diagnosis
**Ferritin level**	Highest ferritin level (μg/L) recorded post-COVID diagnosis
**Venous thromboembolism**	Defined as (1) new symptomatic or asymptomatic deep-vein thrombosis or pulmonary embolism, diagnosed using accepted imaging tests in practice
**Days of mechanical ventilation**	Defined as the number of days requiring mechanical ventilation either invasive or non-invasive
**Tracheostomy and timing of insertion**	Defined as time from intubation to tracheostomy insertion during the ICU stay

In this table, we list the additional data required for this COVID-19 C cohort study

COVID-19 coronavirus disease of 2019, *ICU* intensive care unit

Supplemental material
BMJ Publishing Group Limited (BMJ) disclaims all liability and responsibility arising from any reliance placed on this supplemental material which has been supplied by the author(s)
BMJ Open

or median (interquartile range [IQR]) of the continuous variables will be calculated. Baseline characteristics (e.g., age, sex, APACHE II Score, admission diagnostic category, comorbidities, COVID-19 vaccination status, and pre-hospital acid suppression) will be described. Table 1 presents the statistical analysis plans for the 3 study objectives, hypotheses, and outcomes.

For any outcome that is missing for more than 2% of patients, we will perform multiple imputation. Adjustments for multiple comparisons will not be made for these exploratory analyses. All analyses will be performed using the statistical software SAS version 9.4 [21].

Analysis of objective 1: Characterization of the COVID-19 cohort

New continuous variables added to the REVISE dataset to help characterize the COVID-19 cohort of patients include inflammatory biomarker levels and time until tracheostomy. New dichotomous variables include the incidence of thromboembolic events and treatment with COVID-19-specific therapies. Continuous variables will be summarized using mean ± standard deviation (SD), or median (interquartile range [IQR]), while frequencies and percentages will be used to summarize categorical variables.

Analysis of objective 2: Propensity-matched comparison of patients with and without COVID-19

To explore whether patients with COVID-19 experience different clinical outcomes relative to patients without COVID-19, we will compare the COVID-19 cohort and a propensity-matched non-COVID cohort from REVISE. Propensity score matching attempts to estimate the effect of COVID-19 status by accounting for important covariates that may predict or explain this status. Patients with COVID-19 will be matched with other patients within REVISE using propensity score analysis. We will consider an optimal matching ratio of 2:1 or 1:1 depending on the availability of optimal matches.

The propensity score will be generated using potentially important pre-randomization demographic variables, including age, sex, pre-hospital acid suppression (PPIs or histamine-2-receptor antagonists (H2RAs)), COVID-19 vaccination status, ICU admission category of medical or surgical/trauma, and date of enrollment. Propensity matching will only include patients enrolled after the declaration of the COVID-19 pandemic on March 11, 2020. As a sensitivity analysis, we will match only on pre-hospital PPI use, not H2RAs, given limited evidence to suggest that H2RAs influence susceptibility to COVID-19 infection [22].

Propensity score matching helps to ensure that the distribution of the foregoing covariates is similar between the compared populations [23]. Comparisons between the COVID-19 and matched cohorts will be made on clinically important bleeding and 90-day mortality, incidence of VAP, *C. difficile* infection, patient-important GI bleeding, renal replacement therapy, ICU and hospital mortality, and duration of mechanical ventilation and ICU and hospital stay. We will adjust all analyses for randomized allocation; mortality outcomes will be adjusted for the APACHE II score.

Objective 3: Treatment effect of PPIs in patients with COVID-19 versus other patients

We will explore whether pantoprazole in the ICU has a differential treatment effect on clinically important GI bleeding or 90-day mortality for patients with COVID-19 relative to those without COVID-19. This is a subgroup analysis of the primary REVISE trial. However, in addition, we aim to further evaluate the subgroup effects of pantoprazole in patients with COVID-19 across secondary REVISE outcomes including VAP and *C. difficile* infection, patient-important bleeding, renal replacement therapy, and ICU and hospital mortality, as well as the duration of mechanical ventilation and ICU and hospital stay.

This analysis will be conducted per the intention-to-treat principle. For time to the primary and secondary binary outcomes, we will use Cox proportional hazards regression, adjusted for pre-hospital use of acid suppression (a stratification variable). Analysis of our mortality outcomes will also be adjusted for the APACHE II score. An interaction term will be included in the model to test for this subgroup effect and results will be interpreted per the ICEMAN criteria [24]. We will report hazard ratios with 95% CIs as well as the absolute risk increase or decrease and 95% CIs. For continuous outcomes, we will perform linear regression which will include an interaction term. We will report mean differences with 95% CIs. If needed, we will perform the linear regression on the log-transformed outcome.

Ethics and regulatory approval

The main REVISE trial was approved in participating hospitals by all relevant ethics committees and regulatory authorities prior to starting enrollment. The Hamilton Integrated Research Ethics Board (HiREB) under Clinical Trials Ontario (CTO Study ID 1360) is the main REB of record, which also approved this substudy. Ethics review for the additional COVID-19-specific data collection was undertaken by the Research Ethics Board of record

(the Hamilton Integrated Research Ethics Board), and as needed to comply with local regulations in each participating center. In the absence of any modifications to the REVISE study design, this substudy involved additional data collection to characterize the patients with COVID-19 and facilitate additional future analyses. Therefore, no modifications were made to the consenting process for REVISE patients involved in this embedded COVID-19 study, and no requirements were issued to either reconsent or inform prior REVISE participants of the substudy. Specifically, there was no requirement to inform participants of this substudy, including an additional review of participants' medical records for new data collection. Participants previously randomized in REVISE were not re-consented to participate in the COVID-19 embedded substudy.

Integrated knowledge translation plan

After completion of the COVID-19 REVISE substudy, we will rapidly publish the results. All results will be published with open access to ensure wide dissemination and accessibility of study findings. We plan to publish and present the COVID-19 substudy work at an international congress. We will disseminate a lay summary of findings to the general public through health networks of patient advocates and persons with lived or living experiences. We will generate lay language summaries and visual abstracts for traditional (paper, radio, television) and social media. We will also host videoconferences and regional rounds. We will disseminate structured abstracts and slide decks to local ICU quality councils, provincial organizations (e.g., Alberta Health Services Critical Care Strategic Clinical Network), national policymakers (e.g., Accreditation Canada), and professional societies. Participating organizations, sponsors, and university institutions will be provided with press release briefs to disseminate to their respective audiences.

Discussion

This study, embedded within a global, randomized trial of stress ulcer prophylaxis, will fill important knowledge gaps in the literature that prompted our three research questions and three study objectives. The first is a descriptive analysis of the COVID-19 cohort in REVISE. The second is a propensity-matched analysis of patients with and without COVID-19. The third is an extended subgroup analysis of the REVISE trial, evaluating the effect of pantoprazole on a range of additional clinical outcomes among patients with and without COVID-19.

Evidence describing the association between acid suppression and poor prognosis among patients with COVID-19 has emerged, but results have been variable. To prevent complications among critically ill patients with COVID-19, interim WHO guidance in 2020 suggested H2RAs or PPIs for patients with risk factors for GI bleeding, including mechanical ventilation for ≥ 48 h, coagulopathy, renal replacement therapy, liver disease, multiple comorbidities, and higher organ failure score [25]. Surviving Sepsis Guidelines for the management of critically ill patients with COVID-19 do not address stress ulcer prophylaxis [26]. Randomized trial data on the effect of PPIs among patients with COVID-19 is insufficient to guide clinical practice, and this study will provide direct evidence on this topic.

Limitations of this substudy include the risk of missing 90-day outcomes due to high levels of inter-hospital transfers experienced during COVID-19 [27]. Patients transferred to centers not participating in REVISE or other loss to follow-up. Our propensity analysis cannot adjust for all possible factors predisposing to infection with SARS-CoV-2. We will have no information on patients eligible for REVISE who were not approached for the trial due to concerns about bleeding risk due to COVID-19 therapies or increased risk of VAP due to COVID-19 infection itself. In addition, the number of patients enrolled in REVISE with COVID-19 depends on the pandemic status in participating centers, such that no sample size calculation is possible; thus, we will interpret the findings in light of the 95% confidence intervals.

Strengths of this study include the timely development of a protocol addressing essential research questions relevant to a high-risk subgroup of critically ill mechanically ventilated patients that emerged during the pandemic. The main REVISE Trial [NCT03374800], and this COVID-19 Cohort Study [NCT05715567] are registered. We have specified a priori hypotheses for each of the 3 objectives. As this study was developed after the REVISE Trial began recruitment, we are using both retrospective and prospective data collection methods to identify relevant patients with COVID-19. All necessary data is derived from the hospital stay, except 90-day mortality for those discharged alive; thus, we anticipate minimal missing data.

Trial status

As of June 1, 2023, more than 90% of relevant data for REVISE patients with COVID-19 are in the REVISE database to inform this work. REVISE enrollment is anticipated to close byNovember 2023; 90-day follow-up, chart closure, and analyses for this study will follow later in 2024.

Acknowledgements
The trial was designed by the REVISE Steering Committee including the National and International Management Committees, the REVISE Investigators and Research Coordinators, the Canadian Critical Care Trials Group, and the Australian and New Zealand Intensive Care Society Clinical Trials Group. We are grateful to others at the Methods Center at McMaster University (Lisa Buckingham, France Clarke, Mary Copland, Karlo Matic, Ashley Sawyer, Alyson Takaoka) and The George Institute (Fatima Butt, Anna Cheng, Conrad Nangla, Fiona Osborne, Tina Schneider, Isabella Schoeler, Anna Tippet) for their expertise.

Authors' contributions
Concept and design: Y Arabi, A Binnie, D Cook, A Deane, B Dennis, J Dionne, S English, S Finfer, G Guyatt, M Hardie, D Heels-Ansdell, S Knowles, F Lauzier, J Marshall, J Muscedere, J Myburgh, M Ostermann, L Thabane, J Tsang, and N Zytaruk. Acquisition, analysis, or interpretation of the data: D Cook, B Dennis, M Hardie, D Heels-Ansdell, S Knowles, L Saunders, L Thabane, and N Zytaruk. Drafting of the manuscript: D Cook, B Dennis, D Heels-Ansdell, and N Zytaruk. Statistical analysis: B Dennis, D Heels-Ansdell, and L Thabane. Obtained funding: A Ahmed, A Binnie, D Cook, B Dennis, J Dionne, G Guyatt, D Heels-Ansdell, L Thabane, and J Tsang. Administrative, technical, or material support: D Cook, A Deane, B Dennis, S Finfer, G Guyatt, M Hardie, D Heels-Ansdell, S Knowles, F Lauzier, D Rajbhandari, L Saunders, A Poole, L Thabane, and N Zytaruk. Data integrity: D Cook, B Dennis, M Hardie, D Heels-Ansdell, S Knowles, L Saunders, and N Zytaruk. Ethics approval and consent to participate: not applicable for this retrospective chart review protocol. Approved final manuscript: all authors.

Funding
REVISE is funded by peer-reviewed grants [Canadian Institutes of Health Research 201610PJT-378226-PJT-CEBA-18373 and Canadian Institutes of Health Research 202207CL3-492565-CTP-CEBA-19215], the Hamilton Academy of Health Sciences Organization [HAH-22–009], Physician Services Incorporated (R22-33) and funds from St. Joseph's Healthcare Hamilton and McMaster University. The National Health and Medical Research Council of Australia grant [GNT1124675] funds enrollment in Australia. REVISE was approved by the National Institute for Health Research (NIHR) in the UK as a Portfolio Study [CPMS ID 45782], eligible for support from the NIHR Clinical Research Network. [https://www.nihr.ac.uk/researchers/collaborations-services-and-support-for-your-research/run-your-study/crn-portfolio.htm]. This trial received no support from the commercial or private sector. The funders/sponsors have no role in the conception, design, conduct, oversight, analysis, interpretation, write-up, review, or approval of the manuscript or the decision to submit the manuscript for publication.
Role of the funders: The funders/sponsors have no role in the conception, design, conduct, oversight, analysis, interpretation, write-up, review, or approval of the manuscript or the decision to submit the manuscript for publication.
Composition and responsibilities of participating centers and steering committee: Led by the Canadian Critical Care Trials Group and the Australian & New Zealand Intensive Care Society Clinical Trials Group, the REVISE Methods Centers are located at McMaster University in Hamilton for international centers and The George Institute in Sydney for Australia. These centers (REVISE Methods Centre, The George Institute) housed the primary data management and endpoint adjudication teams, who worked in close collaboration to ensure standardization of data collection and adjudication procedures. The REVISE Canadian Steering Committee comprises Drs. Guyatt, Cook, Alhazzani, Marshall, Lauzier, English, Muscedere, Hall, Xie, and Thabane. The International Management Committee includes Drs. Finfer and Myburgh, Deane, Ostermann, and Arabi joined by Canadian leaders Drs. Guyatt, Cook, Alhazzani, Marshall, and Thabane. These teams meet approximately quarterly, to anticipate and pre-empt challenges and generate solutions to the operational and financial hurdles characterizing global trials. The Data Safety & Monitoring Committee includes Professor Roberts (University of Oxford), Dr. McAuley (Queen's University Belfast), and Chair Dr. Thomlinson (University of Toronto).

Availability of data and materials
Readers are welcome to contact the research team for further information. There are no original data associated with this research protocol.
The datasets analyzed during the current study would only be available following relevant research ethics board discussion and approval. However, the statistical code will be available and any requested details of the protocol.

Declarations

Ethics approval and consent to participate
The main REVISE trial was approved in participating hospitals by all relevant ethics committees and regulatory authorities prior to starting enrollment. The Hamilton Integrated Research Ethics Board (HiREB) under Clinical Trials Ontario (CTO Study ID 1360) is the main REB of record, which also approved this substudy. Ethics review for the additional COVID-19-specific data collection was undertaken as needed to comply with local regulations in each participating center.

Consent for publication
Not applicable for this protocol.

Competing interests
All authors are involved in the REVISE Trial in some capacity. Otherwise, the authors declare that they have no competing interests.

Author details
[1]Department of Medicine, McMaster University, Hamilton, ON, Canada. [2]Department of Health Research Methods, Evidence, and Impact, McMaster University, Hamilton, ON, Canada. [3]Biostatistics Unit, St. Joseph's Healthcare Hamilton, Hamilton, ON, Canada. [4]Division of Critical Care, Research Institute, St. Joseph's Healthcare Hamilton, Hamilton, ON, Canada. [5]Departments of Medicine and Health Research Methods, Evidence, and Impact, McMaster University, McMaster University Health Sciences Center, Room 2C11, 1200 Main Street West, Hamilton, ON L8N 3Z5, Canada. [6]Department of Critical Care Medicine, Niagara Health System, St. Catharines, ON, Canada. [7]Department of Gastroenterology and Hepatology, Stanford University, Palo Alto, CA, USA. [8]Departments of Anesthesiology and Medicine and Critical Care Medicine, Université Laval, Québec, Québec, Canada. [9]Department of Critical Care Medicine, University of Melbourne, Melbourne Medical School, Parkville, VIC, Australia. [10]Intensive Care Department, Ministry of the National Guard-Health Affairs, Riyadh, Kingdom of Saudi Arabia. [11]King Saud Bin Abdulaziz University for Health Sciences, Riyadh, Kingdom of Saudi Arabia. [12]King Abdullah International Medical Research Center, Riyadh, Kingdom of Saudi Arabia. [13]Interdepartmental Division of Critical Care, University of Toronto, Toronto, ON, Canada. [14]Critical Care Program, The George Institute for Global Health, Faculty of Medicine, University of New South Wales, Sydney, NSW, Australia. [15]Intensive Care Unit, St. George Hospital, Sydney, Australia. [16]Department of Critical Care Medicine, Queen's University, Kingston, ON, Canada. [17]Department of Medicine, University of Ottawa, Ottawa, ON, Canada. [18]Clinical Epidemiology Program, Ottawa Hospital Research Institute, Ottawa, ON, Canada. [19]Department of Critical Care, King's College London, Guy's & St Thomas' Hospital, London, UK.

Received: 3 July 2023 Accepted: 16 August 2023
Published online: 30 August 2023

References
1. Richardson S, Hirsch JS, Narasimhan M, Crawford JM, McGinn T, Davidson KW, Barnaby DP, Becker LB, Chelico JD, Cohen SL, et al. Presenting characteristics, comorbidities, and outcomes among 5700 patients hospitalized with COVID-19 in the New York City area. JAMA. 2020;323(20):2052.
2. Guan W-J, Ni Z-Y, Hu Y, Liang W-H, Ou C-Q, He J-X, Liu L, Shan H, Lei C-L, Hui DSC, et al. Clinical characteristics of coronavirus disease 2019 in China. N Engl J Med. 2020;382(18):1708–20.
3. Zhang P, Zhu L, Cai J, Lei F, Qin J-J, Xie J, Liu Y-M, Zhao Y-C, Huang X, Lin L, et al. Association of inpatient use of angiotensin-converting enzyme inhibitors and angiotensin II receptor blockers with mortality among patients with hypertension hospitalized with COVID-19. Circ Res. 2020;126(12):1671–81.
4. Reynolds HR, Adhikari S, Pulgarin C, Troxel AB, Iturrate E, Johnson SB, Hausvater A, Newman JD, Berger JS, Bangalore S, et al. Renin–angiotensin–aldosterone system inhibitors and risk of COVID-19. N Engl J Med. 2020;382(25):2441–8.
5. Lopes RD, Macedo AVS, De BarrosESilva PGM, Moll-Bernardes RJ, Dos Santos TM, Mazza L, Feldman A, D'AndréaSabaArruda G, De Albuquerque

DC, Camiletti AS, et al. Effect of discontinuing vs continuing angiotensin-converting enzyme inhibitors and angiotensin II receptor blockers on days alive and out of the hospital in patients admitted with COVID-19. JAMA. 2021;325(3):254.
6. Xiang Y, Wong KC, So HC. Exploring drugs and vaccines associated with altered risks and severity of COVID-19: a UK Biobank cohort study of all ATC level-4 drug categories reveals repositioning opportunities. Pharmaceutics. 2021;13(9):1514.
7. Lee SW, Ha EK, Yeniova AÖ, Moon SY, Kim SY, Koh HY, Yang JM, Jeong SJ, Moon SJ, Cho JY, et al. Severe clinical outcomes of COVID-19 associated with proton pump inhibitors: a nationwide cohort study with propensity score matching. Gut. 2021;70(1):76–84.
8. Aby ES, Tappata M, Rodin H, Debes JD. Assessment of proton-pump inhibitor use, race, socioeconomic status, and mortality in individuals with COVID-19. Eur J Gastroenterol Hepatol. 2022;34(2):239.
9. Hariyanto TI, Prasetya IB, Kurniawan A. Proton pump inhibitor use is associated with increased risk of severity and mortality from coronavirus disease 2019 (COVID-19) infection. Dig Liver Dis. 2020;52(12):1410–2.
10. Krag M, Perner A, Wetterslev J, Wise MP, Borthwick M, Bendel S, McArthur C, Cook D, Nielsen N, Pelosi P, et al. Prevalence and outcome of gastrointestinal bleeding and use of acid suppressants in acutely ill adult intensive care patients. Intensive Care Med. 2015;41(5):833–45.
11. Cook D, Guyatt G. Prophylaxis against upper gastrointestinal bleeding in hospitalized patients. N Engl J Med. 2018;378(26):2506–16.
12. Re-EValuating the Inhibition of Stress Erosions (REVISE) Trial (REVISE), [NCT03374800]. Clinical trial registry from clinicalTrials.gov; 2018. https://classic.clinicaltrials.gov/ct2/show/NCT03374800. Accessed 28 Aug 2023.
13. Ippolito M, Misseri G, Catalisano G, Marino C, Ingoglia G, Alessi M, Consiglio E, Gregoretti C, Giarratano A, Cortegiani A. Ventilator-associated pneumonia in patients with COVID-19: a systematic review and meta-analysis. Antibiotics (Basel). 2021;10(5):545.
14. Dexamethasone in hospitalized patients with COVID-19. N Engl J Med. 2020;384(8):693–704. https://www.nejm.org/doi/full/10.1056/NEJMoa2021436.
15. Therapeutic anticoagulation with heparin in critically ill patients with COVID-19. N Engl J Med. 2021;385(9);777–89. https://www.nejm.org/doi/full/10.1056/NEJMoa2021436.
16. Chan AW, Tetzlaff JM, Altman DG, Laupacis A, Gøtzsche PC, Krleža-Jerić K, Hróbjartsson A, Mann H, Dickersin K, Berlin JA, et al. SPIRIT 2013 statement: defining standard protocol items for clinical trials. Ann Intern Med. 2013;158(3):200–7.
17. Cook D, Guyatt G, Marshall J, Leasa D, Fuller H, Hall R, Peters S, Rutledge F, Griffith L, McLellan A, et al. A comparison of sucralfate and ranitidine for the prevention of upper gastrointestinal bleeding in patients requiring mechanical ventilation. N Engl J Med. 1998;338(12):791–7.
18. Cook DJ, Fuller HD, Guyatt GH, Marshall JC, Leasa D, Hall R, Winton TL, Rutledge F, Todd T, Roy P, et al. Risk factors for gastrointestinal bleeding in critically ill patients. N Engl J Med. 1994;330(6):377–81.
19. Cook DJ, Griffith LE, Walter SD, Guyatt GH, Meade MO, Heyland DK, Kirby A, Tryba M. Crit Care. 2001;5(6):368.
20. Krag M, Marker S, Perner A, Wetterslev J, Wise MP, Schefold JC, Keus F, Guttormsen AB, Bendel S, Borthwick M, et al. Pantoprazole in patients at risk for gastrointestinal bleeding in the ICU. N Engl J Med. 2018;379(23):2199–208.
21. SAS 9.4. In. Edited by Inc SI. Cary, NC.
22. Hirota K. H_2 antagonists, proton pump inhibitors and COVID-19. J Anesth. 2022;36(3):329–31.
23. Rosenbaum PR, Rubin DB. The central role of the propensity score in observational studies for causal effects. Biometrika. 1983;70(1):41–55.
24. Schandelmaier S, Briel M, Varadhan R, Schmid CH, Devasenapathy N, Hayward RA, Gagnier J, Borenstein M, van der Heijden GJMG, Dahabreh IJ, et al. Development of the Instrument to assess the Credibility of Effect Modification Analyses (ICEMAN) in randomized controlled trials and meta-analyses. Can Med Assoc J. 2020;192(32):E901–6.
25. World Health O. Clinical management of severe acute respiratory infection (SARI) when COVID-19 disease is suspected: interim guidance, 13 March 2020. In. Geneva: World Health Organization; 2020.
26. Alhazzani W, Evans L, Alshamsi F, Møller MH, Ostermann M, Prescott HC, Arabi YM, Loeb M, Ng Gong M, Fan E, et al. Surviving sepsis campaign guidelines on the management of adults with coronavirus disease 2019 (COVID-19) in the ICU: first update. Crit Care Med. 2021;49(3):e219–34.
27. Pett E, Leung HL, Taylor E, Chong MSF, Hla TTW, Sartori G, Sathianathan V, Husain T, Suntharalingam G, Rosenberg A, et al. Critical care transfers and COVID-19: managing capacity challenges through critical care networks. J Intensive Care Soc. 2022;23(2):203–9.

Publisher's Note

Springer Nature remains neutral with regard to jurisdictional claims in published maps and institutional affiliations.

Ready to submit your research? Choose BMC and benefit from:

- fast, convenient online submission
- thorough peer review by experienced researchers in your field
- rapid publication on acceptance
- support for research data, including large and complex data types
- gold Open Access which fosters wider collaboration and increased citations
- maximum visibility for your research: over 100M website views per year

At BMC, research is always in progress.

Learn more biomedcentral.com/submissions

BMC
