# Supplementary material for: Proton pump inhibitors in invasively ventilated patients with SARS-CoV-2: a substudy of the re-evaluating the inhibition of stress erosions trial
Source: BMJ Open. 2026 Aug 31;16(8):e119937. doi: 10.1136/bmjopen-2026-119937 (PMC13536124; doi:10.1136/bmjopen-2026-119937)
Supplement: Supplementary file 2 [file bmjopen-16-8-s002.pdf]

Supplementary Tables:

Supplementary Table S1: Propensity Model Regressing SARS-CoV-2 Status on confounders using multivariable logistic regression

	Adjusted Odds Ratio (95% CI)
**Confounders**	
Age, 10-year increment	1.10 (1.03, 1.19)
Female	1.06 (0.86, 1.30)
Prehospital acid suppression	0.69 (0.54, 0.89)
ICU admission category: medical vs. surgical/trauma	4.34 (2.90, 6.51)
Corticosteroids in the week pre randomization	6.71 (5.33, 8.47)
Days from start of pandemic to enrollment, 1-year increment	0.73 (0.65, 0.81)

Legend Table S1: This stable shows a summary of results of a multivariable logistic regression model (*n*=4477) regressing SARS-CoV-2 status on the *a priori*-selected confounders being used in propensity-matching. SARS-COV-yes (n=467) or SARS-CoV-2-no (n=4010). The model demonstrates significant association between *a priori* selected pre-randomization confounder variables (i.e., age, pre-hospital acid suppression) and SARS-CoV-2 status.

ICU=intensive care unit

Supplementary Table S2: Patient Characteristics and Propensity Scores According to SARS-CoV-2 Status in the Propensity-Matched Cohort

	SARS-CoV-2 Patients	Non- SARS-CoV-2 Patients	Propensity-matched Non- SARS-CoV-2 Patients (2:1)
Number of patients, n (%)	467	4010	934
Age (years), mean ± SD	61 ± 14	58 ± 17	62 ± 13
Female, n (%)	187 (40.0)	1439 (35.9)	365 (39.1)
Prehospital acid suppression, n (%)	104 (22.3)	920 (22.9)	217 (23.2)
ICU admission category, n (%)			
Medical	440 (94.2)	2819 (70.3)	878 (94.0)
Surgical/trauma	27 (5.8)	1191 (29.7)	56 (6.0)
Corticosteroids in the week pre randomization, n (%)	362 (77.5)	1206 (30.1)	726 (77.7)
Days from start of pandemic to enrollment, mean ± SD	669 ± 280	787 ± 344	692 ± 339
Propensity Score			
mean ± SD	0.221 ± 0.110	0.091 ± 0.100	0.219 ± 0.110
median (IQR)	0.257 (0.145-0.305)	0.048 (0.024-0.112)	0.245 (0.146-0.295)
minimum	0.006	0.004	0.006
maximum	0.411	0.439	0.411

Legend Supplementary Table S2: This table shows the baseline characteristics of patients with SARS-CoV-2 and propensity-matched patients without SARS-CoV-2. This table also shows the propensity scores according to SARS-CoV-2 Status in the propensity-matched cohort.
SARS-CoV-2=severe acute respiratory syndrome coronavirus-2
SD=standard deviation
ICU=intensive care unit
IQR=interquartile range

Supplementary Table S3. Association between SARS-CoV-2 Status and Ventilator-associated Pneumonia

	Confounders adjustment using propensity matching (2:1) (n=1401)		Confounders adjustment using multivariable regressions (n=4477)	
Exploratory Model 1	Adjusted Hazard Ratio (95% CI) SARS-CoV-2 (n=467) vs. non- SARS-CoV-2 (n=934)	p-value	Adjusted Hazard Ratio (95% CI) SARS-CoV-2 (n=467) vs. non- SARS-CoV-2 (n=4010)	p-value
Ventilator-associated Pneumonia[1]	1.08 (0.78, 1.50)	0.63	1.00 (0.75, 1.34)	0.98
Exploratory Model 2	Adjusted Hazard Ratio (95% CI) SARS-CoV-2 (n=157) vs. non- SARS-CoV-2 (n=314)	p-value	Adjusted Hazard Ratio (95% CI) SARS-CoV-2 (n=157) vs. non- SARS-CoV-2 (n=3431)	p-value
Ventilator-associated Pneumonia[2]	0.98 (0.72, 1.35)	0.92	1.09 (0.82, 1.46)	0.55

Legend Supplementary Table S3: This table shows results from 2 exploratory analyses evaluating the association between SARS-CoV-2 status and ventilator-associated pneumonia, using different adjustment methods to account for pneumonia diagnoses on admission to the ICU. The 2 methods of confounder adjustment were: 1) Propensity-matching (2:1) and 2) Cox proportional hazards multivariable regression.

[1]Multivariable regression: adjusted for patients' age, sex, pre-hospital acid suppression, ICU admission category (medical or surgical/trauma), corticosteroids in the week pre randomization, days from start of pandemic to enrollment, randomization group (Pantoprazole vs placebo), and baseline pneumonia. Original Propensity-matched data: adjusted for randomization group (Pantoprazole vs placebo), and baseline pneumonia

[2]Patients with pneumonia at baseline were removed and original propensity scores were re-estimated on the reduced data. Exploratory models 1 were refitted in the reduced data.

SARS-CoV-2=severe acute respiratory syndrome coronavirus-2

Supplementary Table S4. Associations between SARS-CoV-2 Status and Clinical Outcomes, accounting for the competing risk of death

	Confounders adjustment using propensity matching (2:1) (n=1401)		Confounders adjustment using multivariable regressions (n=4477)	
	Adj SHR (95% CI) SARS-CoV-2 (n=467) vs. non- SARS-CoV-2 (n=934)	p-value	Adj SHR (95% CI) SARS-CoV-2 (n=467) vs. non- SARS-CoV-2 (n=4010)	p-value
Clinically important upper GI bleed[1]	0.86 (0.45, 1.64)	0.65	0.82 (0.46, 1.48)	0.52
Incident *C. difficile*[1]	0.69 (0.22, 2.17)	0.53	0.79 (0.28, 2.27)	0.67
New renal replacement therapy[1]	1.30 (0.90, 1.90)	0.17	1.33 (0.95, 1.86)	0.09
Patient important upper GI bleed[1]	0.85 (0.48, 1.51)	0.58	0.85 (0.50, 1.43)	0.54
Ventilator-associated pneumonia[1]	0.54 (0.40, 0.74)	< 0.001	0.51 (0.38, 0.68)	< 0.001
Ventilator-associated pneumonia[2]	1.21 (0.87, 1.67)	0.26	1.11 (0.84, 1.46)	0.48

Legend Supplementary Table S4: This table shows results from exploratory analyses evaluating the association between SARS-CoV-2 status and clinical outcomes, accounting for the competing risk of death using Fine and Gray subdistribution hazard regression models.

[1]Adjusted for patients' age, sex, pre-hospital acid suppression, ICU admission category (medical or surgical/trauma), corticosteroids in the week pre randomization, days from start of pandemic to enrollment, and randomization group (Pantoprazole vs placebo)

[2]Adjusted for variables listed in 1 plus baseline pneumonia
