# Supplementary material for: Proton pump inhibitors in invasively ventilated patients with SARS-CoV-2: a substudy of the re-evaluating the inhibition of stress erosions trial
Source: BMJ Open. 2026 Aug 31;16(8):e119937. doi: 10.1136/bmjopen-2026-119937 (PMC13536124; doi:10.1136/bmjopen-2026-119937)
Supplement: Supplementary file 3 [file bmjopen-2026-119937.draft_revisions.pdf]

BMJ Open

BMJ Open is committed to open peer review. As part of this commitment we make the peer review history of every article we publish publicly available.

When an article is published we post the peer reviewers' comments and the authors' responses online. We also post the versions of the paper that were used during peer review. These are the versions that the peer review comments apply to.

The versions of the paper that follow are the versions that were submitted during the peer review process. They are not the versions of record or the final published versions. They should not be cited or distributed as the published version of this manuscript.

BMJ Open is an open access journal and the full, final, typeset and author-corrected version of record of the manuscript is available on our site with no access controls, subscription charges or pay-per-view fees (http://bmjopen.bmj.com).

If you have any questions on BMJ Open's open peer review process please email info.bmjopen@bmj.com

BMJ Open

Proton Pump Inhibitors in Invasively Ventilated Patients with SARS-CoV-2: A Substudy of the Re-EValuating the Inhibition of Stress Erosions (REVISE) Trial

Journal:	*BMJ Open*
Manuscript ID	bmjopen-2026-119937
Article Type:	Original research
Date Submitted by the Author:	24-Mar-2026

Complete List of Authors:	Dennis, Brittany; The University of British Columbia, Department of Medicine<br>Heels-Ansdell, Diane; McMaster University, Department of Health Research Methods, Evidence and Impact<br>Quazi, Ibrahim; McMaster University, Department of Health Research Methods, Evidence and Impact<br>Basmaji, John; Western University, Department of Medicine, Division of Critical Care<br>Thabane, Lehana; McMaster University, Department of Health Research Methods, Evidence, and Impact; St. Joseph's Research Institute; St Joseph's Healthcare Hamilton, Biostatistics Unit<br>Guyatt, Gordon; McMaster University, Department of Medicine, Health Research Methods, Evidence and Impact,<br>Saunders, Lois; St. Joseph’s Healthcare , Research Institute<br>Hand, Lori; McMaster University, Department of Health Research Methods, Evidence and Impact<br>Loy, Olivia; The Royal Melbourne Hospital, Department of Critical Care<br>Zytaruk, Nicole; McMaster University, Health Research Methods, Evidence & Impact; St. Joseph’s Healthcare, Research Institute, Division of Critical Care<br>Hardie, Miranda; University of New South Wales, The George Institute for Global Health, Critical Care Program, Faculty of Medicine<br>Deane, Adam; The University of Melbourne, Department of Critical Care, Melbourne Medical School; The University of Melbourne - Parkville Campus, Intensive Care Unit, Department of Critical Care Medicine<br>Marshall, John; University of Toronto, Interdepartmental Division of Critical Care<br>Arabi, Yaseen; Ministry of National Guard Health Affairs, Intensive Care Department; King Saud bin Abdulaziz University for Health Sciences; King Abdullah International Medical Research Center<br>Lauzier, François; Laval University, Departments of Anesthesiology, Medicine and Critical Care Medicine<br>Dionne, Joanna; McMaster University, Departments of Medicine, Health Research Methods, Evidence, and Impact,<br>Burns, Karen; University of Toronto, Interdepartmental Division of Critical Care<br>Alhazzani, Waleed; Imam Abdulrahman Bin Faisal University, Critical Care and Internal Medicine Department, College of Medicine; Ministry of Defense Health Services, Research and Innovation Institute

For peer review only - http://bmjopen.bmj.com/site/about/guidelines.xhtml

Vazquez-Grande, Gloria; University of Manitoba, Department of Critical Care
Geagea, Anna; North York General Hospital, Critical Care
Myburgh, John; University of New South Wales, Faculty of Medicine; The George Institute for Global Health, Critical Care Program; Saint George Hospital, Intensiv Care Unit
Muscedere, John; Queen's University, Department of Critical Care Medicine
English, Shane; University of Ottawa, Department of Medicine; Ottawa Hospital Research Institute, Clinical Epidemiology Program
Binnie, Alexandra; William Osler Health System, Department of Critical Care Medicine
Tsang, Jennifer; McMaster University Department of Medicine; Niagara Health System, Department of Critical Care Medicine
Hunt, Miranda; Queen's University, Critical Care Medicine
Ostermann, Marlies; King's College London, Guy's & St Thomas' Hospital , Department of Critical Care
Al-Fares, Abdulrahman A; Al-Amiri Hospital, Department of Anesthesia, Critical Care, Pain Medicine and Kuwait Extracorporeal Life Support
Knowles, Serena; The George Institute for Global Health, Critical Care program
Ahmed, Aijaz; Stanford University, Department of Gastroenterology & Hepatology
Cook, Deborah; McMaster University, Departments of Medicine, Health Research Methods, Evidence and Impact; St. Joseph's Healthcare Hamilton Research Institute
The REVISE Investigators, The Canadian Critical Care Trials Group; Sunnybrook and Women's College Health Sciences Centre

Keywords: COVID-19, Adult intensive & critical care < INTENSIVE & CRITICAL CARE, Research Design

SCHOLARONE MANUSCRIPTS

I, the Submitting Author has the right to grant and does grant on behalf of all authors of the Work (as defined in the below author licence), an exclusive licence and/or a non-exclusive licence for contributions from authors who are: i) UK Crown employees; ii) where BMJ has agreed a CC-BY licence shall apply, and/or iii) in accordance with the terms applicable for US Federal Government officers or employees acting as part of their official duties; on a worldwide, perpetual, irrevocable, royalty-free basis to BMJ Publishing Group Ltd ("BMJ") its licensees and where the relevant Journal is co-owned by BMJ to the co-owners of the Journal, to publish the Work in this journal and any other BMJ products and to exploit all rights, as set out in our licence.

The Submitting Author accepts and understands that any supply made under these terms is made by BMJ to the Submitting Author unless you are acting as an employee on behalf of your employer or a postgraduate student of an affiliated institution which is paying any applicable article publishing charge ("APC") for Open Access articles. Where the Submitting Author wishes to make the Work available on an Open Access basis (and intends to pay the relevant APC), the terms of reuse of such Open Access shall be governed by a Creative Commons licence – details of these licences and which Creative Commons licence will apply to this Work are set out in our licence referred to above.

Other than as permitted in any relevant BMJ Author's Self Archiving Policies, I confirm this Work has not been accepted for publication elsewhere, is not being considered for publication elsewhere and does not duplicate material already published. I confirm all authors consent to publication of this Work and authorise the granting of this licence.

For peer review only

Proton Pump Inhibitors in Invasively Ventilated Patients with SARS-CoV-2: A Substudy of the Re-EValuating the Inhibition of Stress Erosions (REVISE) Trial

Brittany Dennis MBBS, PhD Brittany.dennis@ubc.ca
Department of Medicine, University of British Columbia, Canada

Diane Heels-Ansdell, MSc ansdell@mcmaster.ca
Department of Health Research Methods, Evidence, and Impact, McMaster University, Hamilton, Ontario, Canada

Quazi Ibrahim, MSc ibrahiq@mcmaster.ca
Department of Health Research Methods, Evidence, and Impact, McMaster University, Hamilton, Ontario, Canada

John Basmaji, MD, MSc jbasmaji2013@gmail.com
Department of Medicine, Division of Critical Care, Western University, London, Ontario, Canada

Lehana Thabane, PhD thabanl@mcmaster.ca
Department of Health Research Methods, Evidence, and Impact, McMaster University, Hamilton, Ontario, Canada, Biostatistics Unit, St. Joseph's Healthcare Hamilton, Hamilton, Ontario, Canada
St. Joseph's Healthcare Hamilton Research Institute, Hamilton, Ontario, Canada

Gordon Guyatt, MD, MSc guyatt@mcmaster.ca
Department of Health Research Methods, Evidence, and Impact, McMaster University, Hamilton, Ontario, Canada
Department of Medicine, McMaster University, Hamilton, Ontario, Canada

Lois Saunders lsaunde@mcmaster.ca
Research Institute, St. Joseph's Healthcare Hamilton, Ontario, Canada

Lori Hand, RRT handlori@hhsc.ca
Department of Health Research Methods, Evidence, and Impact, McMaster University, Hamilton, Ontario, Canada

Olivia Loy, BSc, MBChB olivia.loy@nhs.net
Department of Critical Care, The Royal Melbourne Hospital, ICU

Nicole Zytaruk, RN zytarn@mcmaster.ca
Department of Health Research Methods, Evidence, and Impact, McMaster University, Hamilton, Ontario, Canada
Research Institute, Division of Critical Care, St. Joseph's Healthcare Hamilton, Ontario, Canada

Miranda Hardie, MClinEpi mhardie@georgeinstitute.org.au
Critical Care Program, The George Institute for Global Health, Faculty of Medicine, University of New South Wales, Sydney, New South Wales, Australia

Adam M Deane, MD Adam.Deane@mh.org.au
Intensive Care Unit, University of Melbourne, Melbourne, Victoria, Australia
Department of Critical Care Medicine, University of Melbourne Parkville Campus, Victoria, Australia

For peer review only

John C Marshall, MD John.Marshall@unityhealth.to
Interdepartmental Division of Critical Care, University of Toronto, Toronto, Ontario, Canada

Yaseen Arabi, MD Arabi@mngha.med.sa
Intensive Care Department, Ministry of the National Guard-Health Affairs, Riyadh, Kingdom of Saudi Arabia
King Saud bin Abdulaziz University for Health Sciences, Riyadh, Kingdom of Saudi Arabia
King Abdullah International Medical Research Center, Riyadh, Kingdom of Saudi Arabia

François Lauzier, MD, MSc Francois.Lauzier@fmed.ulaval.ca
Departments of Anesthesiology, Medicine and Critical Care Medicine, Université Laval, Québec, Québec, Canada

Joanna C Dionne MD, MSc, PhD dionnejc@mcmaster.ca
Department of Medicine, McMaster University, Hamilton, Ontario, Canada
Departments of Health Research Methods, Evidence, and Impact, McMaster University, Hamilton, Ontario, Canada

Karen EA Burns, MD, MSc karen.burns@unityhealth.to
Interdepartmental Division of Critical Care, University of Toronto, Toronto, Ontario, Canada

Waleed Alhazzani, MD, MSc waleed.al-hazzani@medportal.ca
Critical Care and Internal Medicine Department, College of Medicine, Imam Abdulrahman Bin Faisal University, Dammam, Eastern Province, Saudi Arabia
Research and Innovation Institute, Ministry of Defense Health Services, Riyadh, Saudi Arabia

Gloria Vazquez-Grande, MD, PhD gloriavazquezgrande@gmail.com
Department of Critical Care, University of Manitoba, Winnipeg, Manitoba, Canada

Anna Geagea, MD, PhD anna.geagea@nygh.on.ca
Department of Critical Care, North York General Hospital, North York, Ontario, Canada

John Myburgh, MD, PhD jmyburgh@georgeinstitute.org.au
Faculty of Medicine, University of New South Wales, Sydney, New South Wales, Australia
Critical Care Program, The George Institute for Global Health, Sydney, New South Wales, Australia
Intensive Care Unit, St. George Hospital, Kogorah, New South Wales, Australia

John Muscedere, MD John.Muscedere@kingstonhsc.ca
Department of Critical Care Medicine, Queen's University, Kingston, Ontario, Canada

Shane English MD, MSc senglish@toh.ca
Department of Medicine, Division of Critical Care, University of Ottawa, Ottawa, Ontario Canada
Clinical Epidemiology Program, Ottawa Hospital Research Institute, Ottawa, Ontario, Canada

Alexandra Binnie MD, PhD allie@binnie.ca
Department of Critical Care Medicine, William Osler Health System, Brampton, Ontario, Canada

Jennifer LY Tsang MD, PhD Jennifer.Tsang@niagarahealth.on.ca
Department of Medicine, Division of Critical CareMcMaster University, Hamilton, Ontario, Canada
Department of Critical Care Medicine, Niagara Health System, St. Catharines, Ontario, Canada

Miranda Hunt, BA Miranda.hunt@kingstonhsc.ca

Department of Critical Care Medicine, Queen's University, Kingston, Ontario, Canada

Marlies Ostermann, MBBS Marlies.Ostermann@gstt.nhs.uk
Department of Critical Care, King's College London, Guy's & St Thomas' Hospital, London, UK

Abdulrahaman A Al-Fares, MD abdulrahman.alfares@gmail.com
Departments of Anesthesia, Critical Care, Pain Medicine and Kuwait Extracorporeal Life Support Program, Al-Amiri Hospital, Kuwait City, Al Asimah Governate, Kuwait

Serena Knowles, PhD sknowles@georgeinstitute.org.au
Critical Care Program, The George Institute for Global Health, Newton, New South Wales, Australia

Aijaz Ahmed, MD safia93@stanford.edu
Department of Gastroenterology and Hepatology, Stanford University, Palo Alto, California, USA

*Deborah J Cook, MD, MSc debcook@mcmaster.ca
Department of Medicine, McMaster University, Hamilton, Ontario, Canada
Department of Health Research Methods, Evidence, and Impact, McMaster University, Hamilton, Ontario, Canada
St. Joseph's Healthcare Hamilton Research Institute, Hamilton, Ontario, Canada

For the REVISE Investigators and the Canadian Critical Care Trials Group

*Correspondence: DJ Cook, Departments of Medicine & Health Research Methods, Evidence and Impact, McMaster University Health Sciences Center, Room 2C11, 1200 Main Street West, Hamilton, Ontario, Canada, L8N 3Z5; debcook@mcmaster.ca

For peer review only

Proton Pump Inhibitors in Invasively Ventilated Patients with SARS-CoV-2: A Substudy of the Re-EValuating the Inhibition of Stress Erosions (REVISE) Trial

ABSTRACT

Background: Observational studies suggest that acid suppression may worsen outcomes among patients infected with severe acute respiratory syndrome coronavirus-2 (SARS-CoV-2). This pre-planned substudy of the REVISE trial—a randomized controlled trial evaluating the efficacy and safety of pantoprazole in mechanically ventilated patients—sought to (1) describe the clinical characteristics of critically ill patients with SARS-CoV-2, (2) compare clinical outcomes with a propensity-matched non-infected cohort, and (3) assess whether pantoprazole's treatment effects differed by SARS-CoV-2 infection status.

Methods: We included patients enrolled in REVISE with SARS-CoV-2 infection, for whom additional data collection included biomarkers, venous thromboembolism, SARS-CoV-2 therapies, and tracheostomy timing. We performed a propensity-matched analysis of infected and non-infected patients and compared the effect of pantoprazole between patients with and without SARS-CoV-2.

Results: Of the eligible trial cohort, 11.9% (540/4550) had SARS-CoV-2; 532 patients had additional SARS-CoV-2-specific data collection. Of these 532 patients, 87.8% received Covid-19-directed treatments – (dexamethasone 75.2%), 11.7% developed pulmonary embolism and 9.2% developed deep-vein thrombosis. After propensity-matching, SARS-CoV-2 infection was not associated with clinically important upper gastrointestinal bleeding (adjusted Hazard Ratio [aHR] 0.78, 95% Confidence Interval [CI] 0.40–1.50), but was associated with significantly higher ICU, hospital, and 90-day mortality, as well as longer duration of ventilation, ICU and hospital length of stay. The effect of pantoprazole on clinically important upper GI bleeding and 90-day mortality was consistent regardless of SARS-CoV-2 status.

Conclusion: SARS-CoV-2 infection was associated with higher mortality and longer duration of mechanical ventilation, ICU and hospital stay, without an increased risk of clinically important upper gastrointestinal bleeding. Pantoprazole reduced clinically important upper gastrointestinal bleeding without adversely affecting other outcomes.

Trial Registration: REVISE Trial [NCT03374800], SARS-CoV-2 Cohort Study [NCT05715567]
Abstract Word Count: 265
Manuscript Word Count: 3185
Figure: 1; **Tables:** 4; **Supplemental Tables**: 4; **Supplemental Material**: 1
References: 22

Article Summary

Strengths and Limitations of this Study:

- This SARS-CoV-2 Cohort substudy was embedded in a large randomized trial, registered, and guided by a published protocol and statistical analysis plan with pre-specified hypotheses.
- This substudy offers the first randomized trial data on the effect of PPIs among patients with SARS-CoV-2 on a range of clinical outcomes.
- We lack information about SARS-CoV-2 variants and SARS-CoV-2 vaccine timing.
- Our definition of COVID-19 is limited to patients who received COVID-19 specific treatment; other infected patients may have been symptomatic.
- The propensity analysis could not adjust for all possible factors predisposing to SARS-CoV-2 leading to possible unrecognized confounding.

For peer review only - http://bmjopen.bmj.com/site/about/guidelines.xhtml
For peer review only

INTRODUCTION

Early in the pandemic, proton pump inhibitors (PPIs) were hypothesized to influence the susceptibility to severe acute respiratory syndrome coronavirus-2 (SARS-CoV-2) and result in worse clinical outcomes among infected patients, although findings were inconsistent. Among older adults, early observational data suggested that recent exposure to PPIs was associated with a reduced risk of infection. [1] However, several retrospective studies subsequently raised concern that antacid use among SARS-CoV-2 infected patients may worsen clinical outcomes.[2-4] Across large claims-based and hospital-based retrospective cohorts, PPI use was linked to a higher risk of severe coronavirus disease of 2019 (COVID-19), ICU admission, mechanical ventilation, and death, although some associations were attenuated after adjustment for age and comorbidities. [2, 3] Consistent with these signals, a pooled analysis of retrospective studies also suggested an increased risk of mortality associated with COVID-19 among PPI users,[4] as well as a more severe course of infection. [5]

To reduce the risk of upper gastrointestinal (GI) bleeding, clinicians often administer PPIs prophylactically in critically ill patients.[6] During the COVID-19 pandemic, international guidance for the management of these patients supported use of PPIs or histamine-2-receptor antagonists (H2Ras) in high-risk patients,[7] yet sepsis guidance did not directly address stress ulcer prophylaxis,[8] and in the early pandemic period, randomized trials had not yet evaluated whether PPIs affected clinical outcomes for ICU patients with COVID-19. Given unclear guidelines for COVID-19 and practices during the pandemic that could have increased bleeding risk (e.g., anticoagulation [9], high-dose corticosteroids [10]) and an apparent increased risk of ventilator-associated pneumonia [11], we conducted a planned substudy within the Re-EValuating the Inhibition of Stress Erosions (REVISE) trial to evaluate the association between PPI exposure and clinically important outcomes in ICU patients tested positive for SARS-CoV-2.[12]

This prospective substudy was pre-planned, nested within an international randomized blinded trial in critically ill patients, investigating the effect of pantoprazole 40 mg intravenously compared to placebo on the primary *efficacy* outcome of clinically important upper GI bleeding and the primary *safety* outcome of 90-day mortality. REVISE found that pantoprazole significantly lowered the risk of clinically important and patient important upper GI bleeding in invasively ventilated patients, but had no effect on other outcomes including 90-day mortality, ventilator-associated pneumonia, *C. difficil*e infection*,* renal replacement therapy, or mortality at ICU and hospital discharge. [13] While testing positive for SARS-CoV-2 during hospital admission did not influence treatment effect for the primary outcome,[13] the effect of pantoprazole in patients with COVID-19 requiring treatment has not been determined on ventilator-associated pneumonia, *Clostridioides difficile (C. difficile)* infection, and other outcomes in patients.

As SARS-CoV-2 is now endemic and PPIs are commonly prescribed in the ICU, understanding the relationship between PPI exposure and clinical outcomes in this population warrants investigation. Accordingly, within this multi-center SARS-CoV-2 substudy of the REVISE trial [NCT05715567],[12] we (1) describe the clinical characteristics and hospital admission trajectory of critically ill patients tested positive for SARS-CoV-2, (2) compare clinical outcomes with a propensity-matched non-infected cohort, and (3) assess whether pantoprazole's treatment effects differed by COVID-19 status.

METHODS

Nested Cohort Study Design

REVISE was registered [NCT03374800], and the trial design [14], statistical analysis plan, [15] and pandemic adaptations[16] were previously published, as designed by the Canadian Critical Care Trials Group and the Australian and New Zealand Intensive Care Society Clinical Trials Group**..** Overall, 4,821

For peer review only - http://bmjopen.bmj.com/site/about/guidelines.xhtml

patients undergoing invasive mechanical ventilation were enrolled in 68 ICUs in Canada, Australia, Saudi Arabia, United Kingdom, United States, Kuwait, Pakistan and Brazil from July 2019 to October 2023. Study drug (pantoprazole or placebo) was prescribed in the ICU until discontinuation of invasive mechanical ventilation, the occurrence of a prespecified indication or contraindication to PPI use, or death, up to 90 days. In the ICU, detailed daily data collection occurred. On the ward thereafter, *C. difficile* infection, vital status and hospital length of stay were captured. Mortality was documented at 90 days.

This multi-center cohort design was registered [NCT05715567], separately peer-review funded, and guided by a protocol and statistical analysis plan.[12] (**Supplemental Material 1**) We included patients in this substudy if they were enrolled in REVISE on or after March 11, 2020. Notwithstanding the operational constraints imposed by the SARS-CoV-2 pandemic on ICU-based research,[17] REVISE enrollment experienced only temporary recruitment suspensions at participating centers, and did not impact eligibility criteria: patients both with and without SARS-CoV-2 were included.[16] The substudy protocol outlined the objectives, *a priori* hypotheses, and outcomes[12], summarized below.

Briefly, patients were eligible for this substudy according to the inclusion and exclusion criteria of REVISE.[13] Inclusion in the SARS-CoV-2 cohort was based on SARS-CoV-2 viral nucleic acid test positivity by polymerase chain reaction (PCR) from a nasopharyngeal swab, oropharyngeal swab, sputum, endotracheal aspirate, or bronchoalveolar lavage sample, as clinically determined rather than per protocol.[12] Patients were eligible if they tested positive for SARS-CoV-2 in hospital (whether contracted preceding the index ICU admission, pre-hospital, or in hospital).

Patients were eligible for this substudy if they tested positive. These patients contributed to objective 1. However, only patients receiving COVID-19 specific treatment were considered symptomatic for SARS-CoV-2 and were included in analyses pertaining to objectives 2 and 3.

No additional tests or treatments were protocolized, and care was at the discretion of the treating team. Additional data collection on SARS-CoV-2 positive patients included biomarkers, venous thromboembolic events, tracheostomy occurrence and timing, and COVID-19 therapies.

Analysis

As per the substudy statistical analysis plan [12], to characterize the cohort of patients tested positive for SARS-CoV-2 within the REVISE trial (*Objective 1*), we used descriptive statistics. Continuous variables were analyzed and presented using mean ± standard deviation (SD), or median (interquartile range [IQR]) for non-normally distributed or skewed data. Categorical variables are presented using frequencies and percentages.

We examined associations between SARS-CoV-2 status and clinical outcomes after adjusting for age, sex, pre-hospital acid suppression, ICU admission category (medical or surgical/trauma), corticosteroids one week pre-randomization, and days from start of pandemic to enrollment (*Objective 2*). Propensity matching only considered patients enrolled after or on March 11th, 2020.

We implemented two methods to address confounding: 1) multivariable regressions and 2) an optimal propensity score matching (PSM) of 2:1 ratio of non-COVID-19 patients with COVID-19 patients. The score was the probability of COVID-19 predicted by the confounders. While multivariable regression was not originally proposed in the protocol, we added it to evaluate the adjusted association between COVID-19 and clinical outcomes while preserving sample size lost from matching. Using Cox-proportional hazards regressions, we studied the association between COVID-19 and clinically important upper GI bleeds, incident *C difficile*, new renal replacement therapy, patient important upper GI bleeding,

For peer review only - http://bmjopen.bmj.com/site/about/guidelines.xhtml

ventilator-associated pneumonia, and mortality in ICU, hospital, and within 90 days. Ventilator-associated pneumonia was classified in this substudy using the Clinical Pulmonary Infection Score as in the main trial report. The proportional hazard (PH) assumption was checked using interaction of an explanatory variable with log (time). In case the PH assumption was violated for an explanatory variable, an interaction of that variable with log time was considered in the model and relative effects, hazard ratios, were reported at different timepoints. ICU and hospital length of stay were highly skewed to the right. Thus, natural logarithms of these two variables were regressed on COVID-19 status and *a priori* confounders and adjusted (exponentiated) relative effect of COVID-19 on length of stays were reported. For propensity-matching analysis, patients within a matched pair were correlated. To account for correlation in the time-to-event analyses, a robust sandwich type variance estimator for clusters was used. For the length-of-stay outcomes, to account for within matched-pair (cluster) correlations, random intercepts models were used in multivariable linear regressions. All analyses were adjusted for randomized allocation; mortality was also adjusted for APACHE II score. A sensitivity analysis was originally planned to evaluate only pre-hospital PPI use (not H2RAs); however, this was not performed, as less than 1% of patients used H2RAs.[13]

Responding to unexpected findings of a lower risk of ventilator-associated pneumonia among patients with COVID-19 compared to those without COVID-19, exploratory post hoc analyses further evaluated the association between SARS-CoV-2 status and ventilator-associated pneumonia. Both the propensity-matched analysis and the multivariable regression models for the outcome of ventilator-associated pneumonia were revised, adjusting for baseline pneumonia at ICU admission, alongside prespecified confounders and randomized assignment (pantoprazole vs placebo) included in the original models. A second post hoc analysis involved evaluating the association between COVID-19 status and ventilator-associated pneumonia (using both propensity-matching and the multivariable regression methods) was repeated after excluding patients with baseline pneumonia; propensity scores were re-estimated in the reduced cohort and prespecified models were refit. Additional exploratory post hoc analyses evaluated the association between COVID-19 status and clinically important upper GI bleeds, incident *C. difficile*, new renal replacement therapy, patient important upper GI bleeding, and ventilator-associated pneumonia, accounting for the competing risk of death.

Finally, we examined whether pantoprazole had a differential effect on clinically important upper GI bleeding or 90-day mortality among patients with COVID-19 compared with those without (*Objective 3*), using a prespecified subgroup analysis of REVISE.[15] Subgroup effects were also evaluated for secondary trial outcomes of ventilator-associated pneumonia, *C. difficile* infection, patient important upper GI bleeding, renal replacement therapy, ICU and hospital mortality, and duration of mechanical ventilation, ICU stay, and hospital stay. For time-to-event outcomes, subgroup analyses were conducted using Cox proportional hazards models that included COVID-19 status, randomized treatment, and an interaction term between SARS-CoV-2 status and treatment. Analyses followed the intention-to-treat principle, with separate models fitted for each outcome. Models were adjusted for pre-hospital acid suppression use (either PPI or H2RA; exposure to either was a stratification variable), and mortality models were additionally adjusted for APACHE II score. We planned to assess subgroup effects, if any were identified, using the interaction term and interpreted using ICEMAN.[18] Results were reported as hazard ratios [HR]with 95% confidence intervals [CI]. For duration outcomes (mechanical ventilation, ICU stay, and hospital stay), subgroup analyses were performed using linear regression models on the natural logarithms of the outcomes including COVID-19 status, randomized treatment, their interaction term, and pre-hospital acid suppression.

Given negligible missing data (<2%), we did not perform imputation.[13] As specified in the protocol, we did not adjust for multiple comparison adjustments. Analyses were conducted using SAS version 9.4 [19] and STATA 15.1 (College Station, Texas).[19]

For peer review only - http://bmjopen.bmj.com/site/about/guidelines.xhtml

Patient and Public Involvement
Members of the public who were substitute decision makers for critically ill patients, and sometimes critically ill patients themselves, were involved in the informed consent process to participate in the parent REVISE trial. One outcome reported herein (patient important bleeding) was developed by ICU survivors and family members of survivors or decedents [13].

Ethics
Relevant ethics committees and regulatory authorities in participating hospitals approved REVISE. The Hamilton Integrated Research Ethics Board under Clinical Trials Ontario (CTO Study ID 1360), the main board of record, also approved this substudy.

RESULTS

Of 4,821 enrolled patients in the trial, 264 participants were not eligible from this substudy due to pre-pandemic randomization. An additional 7 patients were excluded due to declined or revoked consent, or whose SARS-CoV-2 status remained unknown, resulting in a final cohort of 4,550 patients. Within the remaining study cohort, 11.9% (540/4,010) were tested positive for SARS-CoV-2 at any time during hospitalization. The SARS-CoV-2 -specific case report form was not completed in 8 patients in one center who were therefore excluded in analyses for Objective 1 examining treatments and other characteristics. There were an additional 65 patients for whom the SARS-CoV-2 form indicated no SARS-CoV-2-directed treatment. Because COVID-19 status could not be definitively classified for these 65 patients, they were included in Objective 1 analyses but excluded from comparative analyses (Objective 2: propensity-matched analyses, Objective 3: REVISE SARS-CoV-2 subgroup analyses). Accordingly, the final analytic sample for Objectives 2 and 3 comprised 4,477 patients, including 4,010 non–SARS-CoV-2 patients and 467 COVID-19 patients (Figure 1).

Characteristics of Participants with SARS-CoV-2

Among the 532 patients with complete information on their SARS-CoV-2 status (i.e., treatments received) enrolled in the REVISE trial, 268 were randomized to pantoprazole, and 264 to placebo. Table 1 shows the characteristics of the overall SARS-CoV-2 Cohort. Briefly, almost half of infected participants were unvaccinated *(n*=226, 42.5%). Treatments for COVID-19 were administered to most participants (*n*=467, 87.8%); dexamethasone was most commonly prescribed (*n*=400, 75.2%).

Complications of SARS-CoV-2 were common (e.g., pulmonary embolism and deep vein thrombosis occurred in 62 (11.7%), and 49 (9.2%) participants, respectively). Biomarkers after enrolment captured in this cohort demonstrated high rates of inflammation, with median d-dimer levels at 2855.5 mg/L (IQR: 1180-5000); C-reactive protein of 156.1 mg/dL (IQR: 78.2-243.5); and ferritin of 883 ng/mL (IQR 403-1870).

Comparison of Participants with and without COVID-19

Clinical characteristics and ICU admission data for the COVID-19 cohort are summarized in Table 2. COVID-19 patients were primarily admitted to ICU for medical reasons (COVID-19: 440, 94.2% vs. Non-SARS-CoV-2: 2,819, 70.3%), and most received corticosteroids in the week preceding randomization (COVID-19: 362, 77.5% vs. Non-SARS-CoV-2: 1,206, 30.1%). Evaluation of *a priori* selected variables used in the propensity score model demonstrated that baseline variables were significantly associated with COVID-19 status (Supplementary Table S1). After propensity-matching, patients with COVID-19 and without SARS-CoV-2 were well balanced across baseline demographic and clinical characteristics, as demonstrated in Supplementary Table S2.

For peer review only - http://bmjopen.bmj.com/site/about/guidelines.xhtml

No differences in the propensity-matched analysis were observed between patients with COVID-19 and without SARS-CoV-2 for clinically important upper GI bleeding (adjusted HR [aHR]: 0.78, 95% CI 0.40, 1.50, p=0.45). However, COVID-19 patients had 73% higher hazard of mortality at 30 days compared to non-SARS-CoV-2 propensity-matched patients (aHR: 1.73; 95% CI: 1.06, 2.40). At 60 days, this increased to 150% higher hazard (aHR: 2.50; 95% CI: 1.13, 3.87), and further to 210% at 90-days (aHR: 3.10; 95% CI: 1.10, 5.10). Similarly, we observed higher mortality among COVID-19 patients, both during hospitalization and throughout the 90-day follow-up (Table 3).

Other outcomes including incident C. *difficile* infection, patient important upper GI bleeding, and new renal replacement therapy were not associated with COVID-19 status (Table 3). However, incident infection with VAP appeared to be lower amongst participants who had COVID-19 (aHR: 0.50; 95% CI: 0.37, 0.69).

Post hoc exploratory adjustment for baseline pneumonia diminished the initial apparent association between COVID-19 status and ventilator-associated pneumonia (Supplementary Table S3) (aHR: 1.08; 95% CI: 0.78, 1.50). Further, no association between COVID-19 status and ventilator-associated pneumonia was observed after excluding patients with pneumonia at baseline from the propensity-matched analysis (Supplementary Table S3). Competing risk analyses yielded similar results to those presented in Table 3 and Supplemental Table S3.

COVID-19 was associated with a 45% longer ICU stay (adjusted ratio of length of stay 1.45; 95% CI: 1.33, 1.58, p<0.001), a 30% longer hospital stay (adjusted ratio 1.30; 95% CI: 1.18, 1.43, p<0.001) and a 69% increase in the number of days of mechanical ventilation (adjusted ratio of duration 1.69; 95% CI: 1.53, 1.87, p<0.001)

Overall, results were consistent between multivariable regression models and the primary propensity-matched analyses (Table 3, Supplemental Tables S3 and S4).

Substudy Subgroup Analyses

COVID-19 status in subgroup analyses did not suggest an effect modification of pantoprazole on the primary efficacy outcome of clinically important upper GI bleeding (Non-SARS-CoV-2 HR: 0.30: 95% CI: 0.18, 0.49; COVID-19 HR: 0.27: 95% CI: 0.08, 0.95, interaction p-value: 0.868) or the primary safety outcome of 90-day mortality (Non-SARS-CoV-2 HR: 0.97: 95% CI: 0.86, 1.09; COVID-19 HR: 0.94: 95% CI: 0.69, 1.27, interaction p-value: 0.855). Similarly, no differences were observed for other outcomes of patient important upper GI bleeding, renal replacement therapy, VAP, *C. difficile* infection in hospital, ICU and hospital mortality, duration of mechanical ventilation, ICU stay, or hospital length of stay. Table 4 summarizes the results of all subgroup analyses.

DISCUSSION

Among 4,550 patients in this stress ulcer prophylaxis trial enroled after the beginning of the pandemic, 11% had were tested positive for SARS-CoV-2, most of whom received directed therapies and were deemed to have COVID-19. These patients frequently developed thrombotic complications, and biomarkers demonstrated an intense inflammatory burden. After propensity-matching with patients not tested positive for SARS-CoV-2, COVID-19 infection was not associated with clinically important upper GI bleeding, but was associated with higher ICU, hospital, and 90-day mortality and longer duration of mechanical ventilation, and ICU and hospital length of stay. The effect of pantoprazole reducing clinically important upper GI bleeding with no effect on 90-day mortality was consistent irrespective of

For peer review only - http://bmjopen.bmj.com/site/about/guidelines.xhtml

COVID-19 status. Overall, we found no evidence that SARS-CoV-2 modified the treatment effect of pantoprazole on any outcome.

This study evaluated several outcomes in patients with COVID-19 that have not been well studied, including *C. difficile* infection, patient important upper GI bleeding, and new renal replacement therapy. Our initial analyses suggested that COVID-19 was associated with a lower risk of ventilator-associated pneumonia, inconsistent with previous literature.[11, 20-22] In fact, most studies characterizing COVID-19 patients found a high rate of secondary pneumonia with an incidence ranging between 18-39/1,000 mechanically ventilated days.[20-22] A high risk of ventilator-associated pneumonia was also observed in a systematic review by Ippolito et. al, which found a pooled rate of 45.4% (95% C.I. 37.8-53.2%; 2611/5593 patients).[11] The initial counterintuitive finding of COVID-19 being associated with a lower risk of ventilator-associated pneumonia prompted post hoc exploratory analyses, which suggested that the apparent association was driven by incomplete adjustment for baseline pneumonia – likely more common among patients with COVID-19. Another possible explanation may be difficulty ascertaining a new episode of ventilator-associated pneumonia in patients with COVID-19, which could have led to missed diagnoses and confounding that attenuated once baseline pneumonia was accounted for (or excluded), resulting in no independent association.

Limitations of this study include the lack of information about SARS-CoV-2 variants and SARS-CoV-2 vaccine timing. We also cannot exclude the possibility of selection bias in some centers given a lack of information about patients were eligible for REVISE but who were not enrolled. Our definition of COVID-19 is limited to patients who received COVID-19 specific treatment; others may have been symptomatic. On the other hand, we assumed that those who received specific treatments for COVID-19 actually had COVID-19, whereas a patient could have received dexamethasone for upper respiratory tract edema after dental abscess drainage and tested positive for SARS-CoV-2, without the virus necessarily causing symptoms. No pre-specified sample size calculation was possible to discern an effect modification based on SARS-CoV-2 status. Finally, our propensity analysis could not adjust for all possible factors predisposing to SARS-CoV-2 leading to possible unrecognized confounding.

Strengths of this study include a large and prospectively collected trial dataset. This SARS-CoV-2 Cohort substudy was registered [NCT05715567], hypotheses were pre-specified for each objective and analysis was guided by a statistical analysis plan.[12] This substudy offers the first randomized trial data on the effect of PPIs among patients with SARS-CoV-2 on a range of clinical outcomes.

CONCLUSION

In this preplanned substudy, COVID-19 infection was associated with higher mortality and longer ICU and hospital stays without an increased risk of clinically important upper GI bleeding in critically ill mechanically ventilated patients compared to patients who were not tested positive for SARS-CoV-2. Pantoprazole demonstrated similar effects on bleeding and safety outcomes regardless of COVID-19 status. Despite observational reports suggesting potential harm from acid suppression in patients with COVID-19, pantoprazole reduced clinically important and patient important upper GI bleeding without affecting other outcomes, offering consistent benefit regardless of COVID-19 status, supporting its consideration for stress ulcer prophylaxis.

For peer review only - http://bmjopen.bmj.com/site/about/guidelines.xhtml

Table 1: Characterization of REVISE Patients with tested positive for SARS-CoV-2 During Hospital Stay

Tested positive SARS-CoV-2 positive any time during hospital stay AND part of the SARS-CoV-2 substudy*	Pantoprazole n=268	Placebo n=264	Total n=532
Vaccination status pre-ICU			
Not vaccinated	111 (41.4)	115 (43.6)	226 (42.5)
Unknown	52 (19.4)	60 (22.7)	112 (21.1)
1 dose	21 (7.8)	12 (4.5)	33 (6.2)
2 doses	46 (17.2)	26 (9.8)	72 (13.5)
3 doses	38 (14.2)	51 (19.3)	89 (16.7)
Computed tomography positive for PE, number (%)			
Yes	28 (10.4)	34 (12.9)	62 (11.7)
No	94 (35.1)	115 (43.6)	209 (39.3)
Test not done	146 (54.5)	115 (43.6)	261 (49.1)
Ultrasound positive for one or more DVT, number (%)			
Yes	24 (9.0)	25 (9.5)	49 (9.2)
No	61 (22.8)	55 (20.8)	116 (21.8)
Test not done	183 (68.3)	184 (69.7)	367 (69.0)
Positive for bowel ischemia, number (%)			
Yes	3 (1.1)	5 (1.9)	8 (1.5)
No	53 (19.8)	58 (22.0)	111 (20.9)
Test not done	212 (79.1)	201 (76.1)	413 (77.6)
D Dimer level, mg/L (highest), median (IQR)	2460 (1020-5000) n=142	3485 (1250-6240) n=154	2855.5 (1180-5000) n=296
C-reactive protein level, mg/dL (highest), median (IQR)	148.0 (72.3-243.0) n=185	163.5 (89.8-252.0) n=199	156.1 (78.2-243.5) n=384
Ferritin level, ng/mL (highest), median (IQR)	905 (440-1717) n=113	866.5 (370-1935) n=124	883 (403-1870) n=237
Possible SARS-CoV-2-related treatments			
ACE2 Renin-angiotensin Inhibitor	30 (11.2)	20 (7.6)	50 (9.4)
Azithromycin	114 (42.5)	117 (44.3)	231 (43.4)
Baricitinib (Janus-associated kinase (JAK) inhibitor)	23 (8.6)	18 (6.8)	41 (7.7)
Casirivimab	2 (0.7)	4 (1.5)	6 (1.1)
Casirivimab and imdevimab (*casirivimab* / imdevimab monoclonal antibody)	2 (0.7)	0 (0.0)	2 (0.4)
Convalescent plasma	0 (0.0)	0 (0.0)	0 (0.0)
Dexamethasone	201 (75.0)	199 (75.4)	400 (75.2)
Other high dose steroid	32 (11.9)	46 (17.4)	78 (14.7)
Nirmatrelvir/ritonavir *(Paxlovid)*	3 (1.1)	2 (0.8)	5 (0.9)
Molnupiravir	2 (0.7)	1 (0.4)	3 (0.6)
Oseltamivir	3 (1.1)	6 (2.3)	9 (1.7)
Remdesivir	57 (21.3)	60 (22.7)	117 (22.0)
Ruxolitinib	0 (0.0)	1 (0.4)	1 (0.2)
Sarilumab	5 (1.9)	3 (1.1)	8 (1.5)
Sotrovimab (monoclonal antibody)	0 (0.0)	1 (0.4)	1 (0.2)

For peer review only

Statin	51 (19.0)	46 (17.4)	97 (18.2)
Tixagevimab/cilgavimab	0 (0.0)	1 (0.4)	1 (0.2)
Tocilizumab	77 (28.7)	76 (28.8)	153 (28.8)
Intravenous vitamin C	20 (7.5)	21 (8.0)	41 (7.7)
Other†	16 (6.0)	17 (6.4)	33 (6.2)
Extra-corporeal membrane oxygenation	2 (0.7)	7 (2.7)	9 (1.7)
Any of the above	237 (88.4)	230 (87.1)	467 (87.8)
None of the above	31 (11.6)	34 (12.9)	65 (12.2)
Tracheostomy, number (%)	37 (13.8)	47 (17.8)	84 (15.8)
Days from randomization to tracheostomy, in those with a tracheostomy	n=37	n=47	n=84
Tracheostomy prior to day of randomization	4 (10.8)	3 (6.4)	7 (8.3)
0-6 days	2 (5.4)	2 (4.3)	4 (4.8)
7-13 days	6 (16.2)	10 (21.3)	16 (19.0)
14-20 days	10 (27.0)	15 (31.9)	25 (29.8)
21-27 days	11 (29.7)	7 (14.9)	18 (21.4)
28-34 days	2 (5.4)	6 (12.8)	8 (9.5)
35 or more days	2 (5.4)	4 (8.5)	6 (7.1)
Days from randomization to tracheostomy in those with no tracheostomy prior to day of randomization	n=33	n=44	n=77
mean (SD)	19.0 (8.9)	21.1 (16.2)	20.2 (13.5)
median (IQR)	19 (14-22)	17.5 (12.5-24)	18 (13-23)
total range	0-47	0-105	0-105

Legend for Table 1: In this table, we describe the 532 patients with confirmed SARS-CoV-2 at any time during hospital stay who are part of the comparative analyses for this substudy. However, there are 8 SARS-CoV-2 positive patients from 1 center which did not participate in the SARS-CoV-2 substudy.

† Other treatments include: hydroxychloroquine, ivermectin, colchicine, ASA, vitamin D, acyclovir, valacyclovir, zinc, rivaroxaban.

SARS-CoV-2= severe acute respiratory syndrome coronavirus-2
ICU=intensive care unit
PE=pulmonary embolism
DVT=deep vein thrombosis
ACE2=angiotensin converting enzyme-2
IQR=interquartile range
SD=standard deviation

For peer review only

Table 2: Clinical Characteristics of Patients by COVID-19 Status

	Patients not tested positive for SARS-CoV-2 n=4010	COVID-19 Patients n=467	Total n=4477
Age in years, mean (SD)	57.9 (16.5)	61.4 (14.0)	58.2 (16.3)
APACHE II score, mean (SD)	21.8 (8.3)	20.9 (8.0)	21.7 (8.3)
Sex, number (%)			
Female	1439 (35.9)	187 (40.0)	1626 (36.3)
Male	2571 (64.1)	280 (60.0)	2851 (63.7)
Patient status, number (%)			
Medical	2819 (70.3)	440 (94.2)	3259 (72.8)
Surgical	564 (14.1)	15 (3.2)	579 (12.9)
Trauma	627 (15.6)	12 (2.6)	639 (14.3)
Admitting diagnostic category, number (%)			
Cardiovascular	441 (11.0)	23 (4.9)	464 (10.4)
Respiratory	1037 (25.9)	365 (78.2)	1402 (31.3)
Gastrointestinal	191 (4.8)	6 (1.3)	197 (4.4)
Neurologic	979 (24.4)	31 (6.6)	1010 (22.6)
Sepsis	338 (8.4)	14 (3.0)	352 (7.9)
Trauma	627 (15.6)	12 (2.6)	639 (14.3)
Metabolic	169 (4.2)	8 (1.7)	177 (4.0)
Renal	59 (1.5)	2 (0.4)	61 (1.4)
Other Medical	61 (1.5)	4 (0.9)	65 (1.5)
Other Surgical	108 (2.7)	2 (0.4)	110 (2.5)
Pre-hospital acid suppression, number (%)			
Yes	920 (22.9)	104 (22.3)	1024 (22.9)
No	3090 (77.1)	363 (77.7)	3453 (77.1)
Corticosteroids in the week pre-randomization, number (%)	1206 (30.1)	362 (77.5)	1568 (35.0)
**Study Day 1**, number (%)			
Invasive mechanical ventilation	4010 (100.0)	467 (100.0)	4477 (100.0)
Inotrope or vasopressor infusion	2825 (70.4)	343 (73.4)	3168 (70.8)
Renal replacement therapy	272 (6.8)	17 (3.6)	289 (6.5)

Legend for Table 2: This table summarizes the clinical characteristics and information on the critical admission for patients from the REVISE trial with data presented disaggregated by SARS-CoV-2 status.

SD=standard deviation
APACHE II score= Acute Physiology and Chronic Health Evaluation II Score
SARS-CoV-2= severe acute respiratory syndrome coronavirus-2

For peer review only

Table 3. Associations between SARS-CoV-2 status and Clinical Outcomes

	**Confounders adjustment using propensity-matching (2:1) (n=1401)**		**Confounders adjustment using multivariable regressions[1] (n=4477)**	
**Outcomes**	Adjusted Hazard Ratio (95% CI) SARS-CoV-2 (n=467) vs. non-SARS-CoV-2 (n=934)	p-value	Adjusted Hazard Ratio (95% CI) SARS-CoV-2 (n=467) vs. non-SARS-CoV-2 (n=4010)	p-value
**Time to event outcome: no violation of Proportional Hazards assumption by SARS-CoV-2 status**				
Clinically important upper GI bleed[1]	0.78 (0.40, 1.50)	0.45	0.74 (0.41, 1.34)	0.32
Incident *C. difficile*[1]	0.67 (0.21, 2.10)	0.49	0.75 (0.25, 2.23)	0.61
New renal replacement therapy[1]	1.24 (0.85, 1.81)	0.26	1.25 (0.88, 1.77)	0.21
Patient important upper GI bleed[1]	0.77 (0.43, 1.38)	0.38	0.76 (0.45, 1.31)	0.32
Ventilator-associated pneumonia[1]	0.50 (0.37, 0.69)	< 0.001	0.47 (0.35, 0.63)	< 0.001
**Time to event outcome: violation of Proportional Hazards assumption by SARS-CoV-2 status**				
ICU mortality[2] at				
30 days	1.73 (1.06, 2.40)	< 0.001	1.47 (1.02, 1.91)	< 0.001
60 days	2.50 (1.13, 3.87)	< 0.001	2.07 (1.17, 2.98)	< 0.001
90 days	3.10 (1.10, 5.10)	0.002	2.54 (1.21, 3.87)	< 0.001
Hospital mortality[2] at				
30 days	1.62 (1.17, 2.07)	< 0.001	1.49 (1.14, 1.84)	< 0.001
60 days	2.18 (1.33, 3.03)	< 0.001	2.03 (1.35, 2.70)	< 0.001
90 days	2.59 (1.39, 3.79)	< 0.001	2.43 (1.46, 3.39)	< 0.001
Mortality within 90 days[2]				
30 days	1.91 (1.45, 2.37)	< 0.001	1.78 (1.41, 2.16)	< 0.001
60 days	2.65 (1.76, 3.55)	< 0.001	2.56 (1.79, 3.32)	< 0.001
90 days	3.22 (1.93, 4.50)	< 0.001	3.16 (2.01, 4.30)	< 0.001
**Linear regression models studying associations between length of stay outcomes and SARS-CoV-2 status**				
	Adjusted Ratio of Length of Stay/Duration (95% CI)[¥]	p-value	Adjusted Ratio of Length of Stay/Duration (95% CI)[¥]	p-value
Outcomes				
**Duration of ICU stay in days**[¶, 1]				

For peer review only - http://bmjopen.bmj.com/site/about/guidelines.xhtml

Exposure of interest: SARS-CoV-2 vs. non-SARS-CoV-2	1.45 (1.33, 1.58)	< 0.001	1.45 (1.35, 1.57)	< 0.001
**Duration of hospital stay in days¶, 1**				
Exposure of interest: SARS-CoV-2 vs. non-SARS-CoV-2	1.30 (1.18, 1.43)	< 0.001	1.35 (1.24, 1.48)	< 0.001
**Duration of mechanical ventilation in days¶, 1**				
Exposure of interest: SARS-CoV-2 vs. non-SARS-CoV-2	1.69 (1.53, 1.87)	< 0.001	1.66 (1.52, 1.81)	< 0.001

Legend for Table 3: In this table we show the effect of SARS-CoV-2 status on clinical outcomes among mechanically ventilated patients admitted to the ICU. Cox proportional hazard regression models and linear regression models were used to study associations between SARS-CoV-2 status and clinical outcomes using two methods of confounders adjustment: 1) Propensity-matching (2:1) and 2) multivariable regression.

[1]Adjusted for patients' age, sex, pre-hospital acid suppression, ICU admission category (medical or surgical/trauma), corticosteroids in the week pre randomization, days from start of pandemic to enrollment, and randomization group (Pantoprazole vs placebo)
[2]Adjusted for variables listed in 1, and APACHE II score quartiles
¶Natural logarithm of length of stay was the dependent variable in the linear regressions
¥Exponentiation of the adjusted regression coefficient and its' confidence intervals are presented

SARS-CoV-2= severe acute respiratory syndrome coronavirus-2
GI=gastrointestinal
C. difficile=Clostridioides difficile
ICU=intensive care unit
CI=confidence interval

For peer review only

Table 4: Efficacy of Pantoprazole in Patients with SARS-CoV-2 in REVISE: Subgroup Analyses

	Pantoprazole n (%)	Placebo n (%)	Hazard Ratio (95% CI)	P-value for the interaction term
**Primary Efficacy Outcome (Clinically Important Upper GI Bleeding)**				
Non-SARS-CoV-2	20/1983 (1.0)	67/1982 (3.4)	**0.30 (0.18, 0.49)**	0.868
SARS-CoV-2	3/235 (1.3)	12/226 (5.3)	**0.27 (0.08, 0.95)**	
**Primary Safety Outcome (90-day Mortality)**				
Non-SARS-CoV-2	567/1987 (28.5)	583/1986 (29.4)	0.97 (0.86, 1.09)†	0.855†
SARS-CoV-2	82/235 (34.9)	88/224 (39.3)	0.94 (0.69, 1.27)†	
**Incident Ventilator-associated pneumonia (Clinical Pulmonary Infection Score ≥6) in ICU**				
Non-SARS-CoV-2	487/1990 (24.5)	495/1986 (24.9)	1.00 (0.88, 1.13)	0.881
SARS-CoV-2	26/235 (11.1)	26/226 (11.5)	1.04 (0.61, 1.80)	
**Incident *C. difficile* infection in hospital**				
Non-SARS-CoV-2	21/1983 (1.1)	16/1982 (0.8)	1.33 (0.69, 2.55)	0.985
SARS-CoV-2	4/235 (1.7)	0/226 (0.0)	-	
**New renal replacement therapy in ICU**				
Non-SARS-CoV-2	110/1983 (5.5)	110/1985 (5.5)	1.01 (0.77, 1.31)	0.308
SARS-CoV-2	26/235 (11.1)	20/226 (8.8)	1.41 (0.78, 2.52)	
**ICU mortality**				
Non-SARS-CoV-2	392/1998 (19.6)	401/1997 (20.1)	1.00 (0.87, 1.15)	0.944
SARS-CoV-2	60/236 (25.4)	67/227 (29.5)	1.01 (0.71, 1.44)	
**Hospital mortality**				
Non-SARS-CoV-2	514/1996 (25.8)	536/1988 (27.0)	0.99 (0.87, 1.11)	0.663
SARS-CoV-2	71/236 (30.1)	80/227 (35.2)	0.92 (0.66, 1.26)	
**Incident Patient important Upper GI Bleeding**				
Non-SARS-CoV-2	28/1983 (1.4)	81/1982 (4.1)	0.34 (0.22, 0.53)	0.763
SARS-CoV-2	5/235 (2.1)	13/226 (5.8)	0.41 (0.15, 1.15)	
	**Pantoprazole median (IQR)**	**Placebo median (IQR)**	**Ratio of Duration (95% CI)**	**P-value for the interaction term**
**Duration of mechanical ventilation in days**				
Non-SARS-CoV-2	6 (3-11)	6 (3-11)	0.97 (0.92, 1.03)	0.170
SARS-CoV-2	9 (5-16)	10 (6-19)	0.87 (0.74, 1.01)	
**Duration of ICU stay in days**				

For peer review only - http://bmjopen.bmj.com/site/about/guidelines.xhtml

Non-SARS-CoV-2	9 (6-15)	9 (6-16)	0.98 (0.93, 1.02)	0.129
SARS-CoV-2	13 (7-21)	14 (9-23)	0.87 (0.76, 1.00)	
**Duration of hospital stay in days**				
Non-SARS-CoV-2	20 (11-35)	21 (11-37)	0.96 (0.91, 1.02)	0.690
SARS-CoV-2	22 (15-40)	25 (14-43)	0.93 (0.79, 1.09)	

Legend for Table 4: In this table we show the effect of pantoprazole versus placebo on clinical outcomes among patients with and without SARS-CoV-2.
† For 90-day mortality, the SARS-CoV-2 subgroup variable violates the assumption of proportional hazards. Therefore, the first Cox proportional hazards model includes only “Non-SARS-CoV-2” patients, and a separate Cox proportional hazards model including only “SARS-CoV-2” patients. The two hazard ratios are compared by performing a z-test on the log(hazard ratio). We did not apply criteria to assess subgroup credibility because all p-values were above 0.10, according to our statistical analysis plan.

SARS-CoV-2= severe acute respiratory syndrome coronavirus-2
GI=gastrointestinal
C. difficile =*Clostridioides difficile*
ICU=intensive care unit

For peer review only

Acknowledgements: The trial was designed by the REVISE Steering Committee including National and International Management Committees, the REVISE Investigators and Research Coordinators, the Canadian Critical Care Trials Group and the Australian and New Zealand Intensive Care Society Clinical Trials Group. We are grateful to others at the Methods Center at McMaster University (Lisa Buckingham, France Clarke, Mary Copland, Karlo Matic, Ashley Sawyer, Alyson Takaoka) and The George Institute (Fatima Butt, Anna Cheng, Conrad Nangla, Fiona Osborne, Tina Schneider, Isabella Schoeler, Anna Tippet) for their expertise.

Competing interests: All authors are involved in the REVISE Trial in some capacity except OL and AA. Otherwise, the authors declare that they have no competing interests.

Funding Statement: This SARS-CoV-2 Substudy was funded by a peer-review grant from Physicians Services Incorporated of Ontario (R22-33). REVISE is funded by peer-reviewed grants [Canadian Institutes of Health Research (201610PJT-378226-PJT-CEBA-18373) and Canadian Institutes of Health Research (202207CL3-492565-CTP-CEBA-19215)], the Hamilton Academy of Health Sciences Organization (HAH-22-009), Physician Services Incorporated (R22-33), funds from St. Joseph's Healthcare Hamilton and McMaster University. The National Health and Medical Research Council of Australia grant [GNT1124675] funds enrolment in Australia. REVISE was approved by the National Institute for Health Research (NIHR) in the UK as a Portfolio Study [CPMS ID 45782], eligible for support from the NIHR Clinical Research Network. [https://www.nihr.ac.uk/researchers/collaborations-services-and-support-for-your-research/run-your-study/crn-portfolio.htm]. This trial received no support from the commercial or private sector. The funders/sponsors have no role in the conception, design, conduct, oversight, analysis, interpretation, write-up, review or approval of the manuscript, or decision to submit the manuscript for publication.

Role of the Funders: The funders and sponsors have no role in the conception, design, conduct, oversight, analysis, interpretation, write-up, review or approval of the manuscript, or decision to submit the manuscript for publication.

Ethics and Regulatory Approval: The main REVISE trial was approved in participating hospitals by all relevant ethics committees and regulatory authorities prior to starting enrolment. The Hamilton Integrated Research Ethics Board (HiREB) under Clinical Trials Ontario (CTO) Study ID 1360 is the main REB of record, which also approved this substudy. Ethics review for the additional SARS-CoV-2-specific data collection was undertaken as needed to comply with local regulations in each participating center.

Authors' contributions:

Concept and design: Y Arabi, A Binnie, D Cook, A Deane, B Dennis, J Dionne, S English, G Guyatt, M Hardie, D Heels-Ansdell, S Knowles, F Lauzier, J Marshall, J Muscedere, J Myburgh, M Ostermann, Saunders, L Thabane, J Tsang, N Zytaruk

Acquisition, analysis, or interpretation of data: J Basmaji, D Cook, B Dennis, L Hand, M Hardie, D Heels-Ansdell, S Knowles, O Loy, L Saunders, L Thabane, N Zytaruk

Drafting of the manuscript: D Cook, B Dennis, D Heels-Ansdell, Q Ibrahim

Statistical analysis: J Basmaji, G Guyatt, D Heels-Ansdell, Q Ibrahim, L Thabane

Obtained funding for this substudy: A Ahmed, A Binnie, D Cook, B Dennis, J Dionne, G Guyatt, D Heels-Ansdell, L Thabane, J Tsang

For peer review only - http://bmjopen.bmj.com/site/about/guidelines.xhtml

Administrative, technical, or material support: J Basmaji, D Cook, A Deane, B Dennis, G Guyatt, M Hardie, D Heels-Ansdell, Q Ibrahim, S Knowles, F Lauzier, O Loy, L Saunders, A Poole, L Thabane, N Zytaruk

Data Integrity: J Basmaji, D Cook, B Dennis, M Hardie, D Heels-Ansdell, Q Ibrahim, S Knowles, N Zytaruk

Approved final manuscript: All authors

Availability of data and materials: Readers are welcome to contact the research team for further information.

For peer review only

REFERENCES

1. Xiang Y, Wong KC, So HC: **Exploring Drugs and Vaccines Associated with Altered Risks and Severity of COVID-19: A UK Biobank Cohort Study of All ATC Level-4 Drug Categories Reveals Repositioning Opportunities**. *Pharmaceutics* 2021, **13**(9).
2. Lee SW, Ha EK, Yeniova AÖ, Moon SY, Kim SY, Koh HY, Yang JM, Jeong SJ, Moon SJ, Cho JY *et al*: **Severe clinical outcomes of COVID-19 associated with proton pump inhibitors: a nationwide cohort study with propensity score matching**. *Gut* 2021, **70**(1):76-84.
3. Aby ES, Tappata M, Rodin H, Debes JD: **Assessment of proton-pump inhibitor use, race, socioeconomic status, and mortality in individuals with COVID-19**. *European Journal of Gastroenterology & Hepatology* 2022, **34**(2):239.
4. Hariyanto TI, Prasetya IB, Kurniawan A: **Proton pump inhibitor use is associated with increased risk of severity and mortality from coronavirus disease 2019 (COVID-19) infection**. *Dig Liver Dis* 2020, **52**(12):1410-1412.
5. Pranata R, Huang I, Lawrensia S, Henrina J, Lim MA, Lukito AA, Kuswardhani RAT, Wibawa IDN: **Proton pump inhibitor on susceptibility to COVID-19 and its severity: a systematic review and meta-analysis**. *Pharmacol Rep* 2021, **73**(6):1642-1649.
6. Krag M, Perner A, Wetterslev J, Wise MP, Borthwick M, Bendel S, McArthur C, Cook D, Nielsen N, Pelosi P *et al*: **Prevalence and outcome of gastrointestinal bleeding and use of acid suppressants in acutely ill adult intensive care patients**. *Intensive Care Med* 2015, **41**(5):833-845.
7. World Health O: **Clinical management of severe acute respiratory infection (SARI) when COVID-19 disease is suspected: interim guidance, 13 March 2020**. In. Geneva: World Health Organization; 2020.
8. Alhazzani W, Evans L, Alshamsi F, Møller MH, Ostermann M, Prescott HC, Arabi YM, Loeb M, Ng Gong M, Fan E *et al*: **Surviving Sepsis Campaign Guidelines on the Management of Adults With Coronavirus Disease 2019 (COVID-19) in the ICU: First Update**. *Crit Care Med* 2021, **49**(3):e219-e234.
9. **Therapeutic Anticoagulation with Heparin in Critically Ill Patients with Covid-19**. *New England Journal of Medicine* 2021, **385**(9):777-789.
10. **Dexamethasone in Hospitalized Patients with Covid-19**. *New England Journal of Medicine* 2020, **384**(8):693-704.
11. Ippolito M, Misseri G, Catalisano G, Marino C, Ingoglia G, Alessi M, Consiglio E, Gregoretti C, Giarratano A, Cortegiani A: **Ventilator-Associated Pneumonia in Patients with COVID-19: A Systematic Review and Meta-Analysis**. *Antibiotics (Basel)* 2021, **10**(5).
12. Dennis BB, Thabane L, Heels-Ansdell D, Dionne JC, Binnie A, Tsang J, Guyatt G, Ahmed A, Lauzier F, Deane A *et al*: **Proton pump inhibitors in critically ill mechanically ventilated patients with COVID-19: protocol for a substudy of the Re-EValuating the Inhibition of Stress Erosions (REVISE) Trial**. *Trials* 2023, **24**(1):561.
13. Cook D, Deane A, Lauzier F, Zytaruk N, Guyatt G, Saunders L, Hardie M, Heels-Ansdell D, Alhazzani W, Marshall J *et al*: **Stress Ulcer Prophylaxis during Invasive Mechanical Ventilation**. *N Engl J Med* 2024, **391**(1):9-20.
14. **Re-EValuating the Inhibition of Stress Erosions (REVISE) Trial (REVISE)**. In. ClinicalTrials.Gov; 2018.
15. Heels-Ansdell D, Billot L, Thabane L, Alhazzani W, Deane A, Guyatt G, Finfer S, Lauzier F, Myburgh J, Young P *et al*: **REVISE: re-evaluating the inhibition of stress erosions in the ICU-statistical analysis plan for a randomized trial**. *Trials* 2023, **24**(1):796.
16. Dennis B, Deane A, Lauzier F, Zytaruk N, Hardie M, Hammond N, Finfer S, Arabi Y, Marshall J,

For peer review only - http://bmjopen.bmj.com/site/about/guidelines.xhtml

Saunders L *et al*: **Protocol implementation during the COVID-19 pandemic: experiences from a randomized trial of stress ulcer prophylaxis**. *BMC Med Res Methodol* 2024, **24**(1):109.
17. Cook DJ, Kho ME, Duan EH, Alhazzani W, Takaoka A, Clarke FJ, Zytaruk N, Vanstone M: **Principles Guiding Nonpandemic Critical Care Research During a Pandemic**. *Crit Care Med* 2020, **48**(10):1403-1410.
18. Schandelmaier S, Briel M, Varadhan R, Schmid CH, Devasenapathy N, Hayward RA, Gagnier J, Borenstein M, van der Heijden GJMG, Dahabreh IJ *et al*: **Development of the Instrument to assess the Credibility of Effect Modification Analyses (ICEMAN) in randomized controlled trials and meta-analyses**. *Canadian Medical Association Journal* 2020, **192**(32):E901-E906.
19. **SAS 9.4**. In. Edited by Inc SI. Cary, NC.
20. Maes M, Higginson E, Pereira-Dias J, Curran MD, Parmar S, Khokhar F, Cuchet-Lourenço D, Lux J, Sharma-Hajela S, Ravenhill B *et al*: **Ventilator-associated pneumonia in critically ill patients with COVID-19**. *Crit Care* 2021, **25**(1):25.
21. Giacobbe DR, Battaglini D, Enrile EM, Dentone C, Vena A, Robba C, Ball L, Bartoletti M, Coloretti I, Di Bella S *et al*: **Incidence and Prognosis of Ventilator-Associated Pneumonia in Critically Ill Patients with COVID-19: A Multicenter Study**. *J Clin Med* 2021, **10**(4).
22. Blonz G, Kouatchet A, Chudeau N, Pontis E, Lorber J, Lemeur A, Planche L, Lascarrou JB, Colin G: **Epidemiology and microbiology of ventilator-associated pneumonia in COVID-19 patients: a multicenter retrospective study in 188 patients in an un-inundated French region**. *Crit Care* 2021, **25**(1):72.

For peer review only

For peer review only - http://bmjopen.bmj.com/site/about/guidelines.xhtml

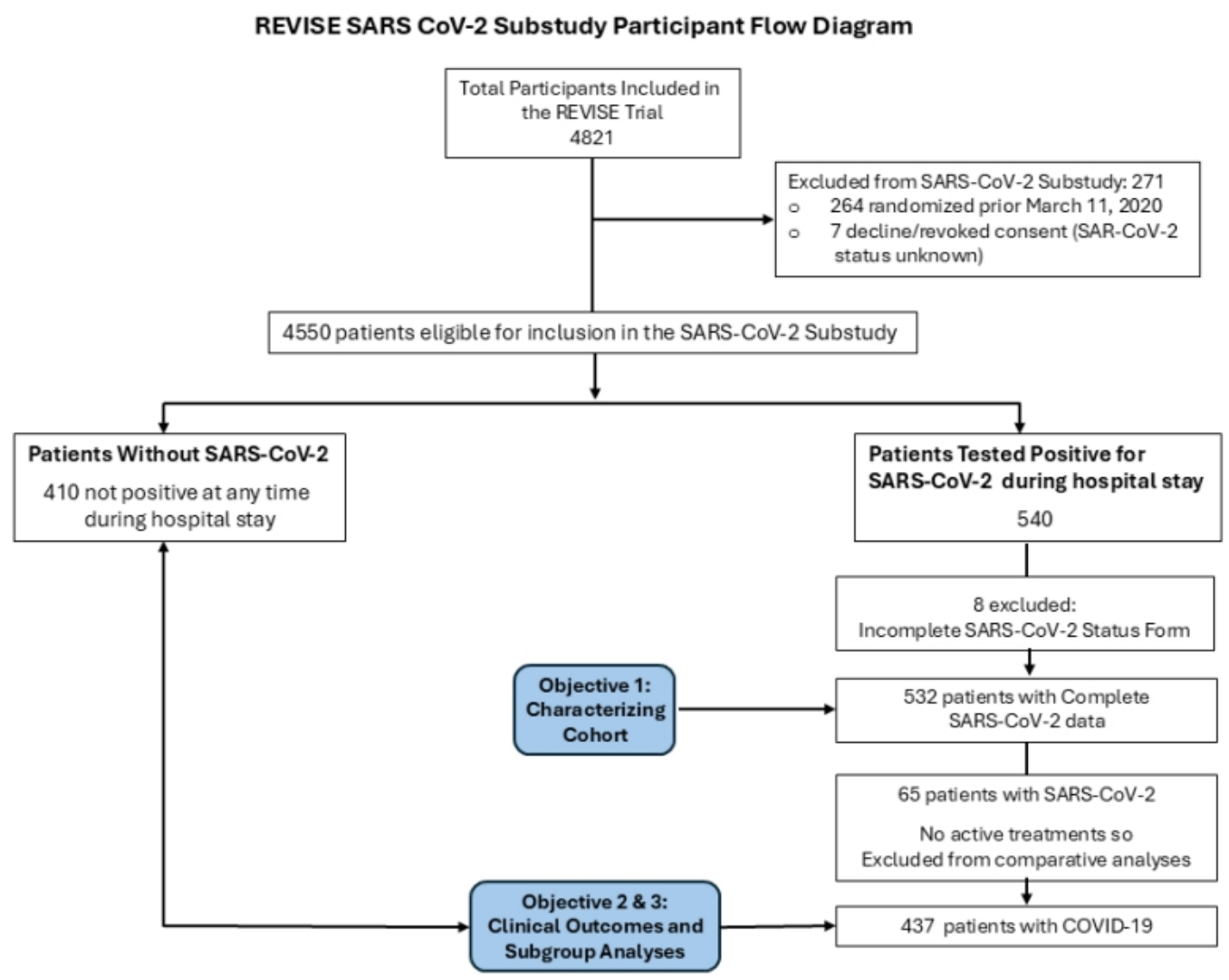

This figure summarizes participant inclusion into the SARS-CoV-2 Substudy, highlighting differences in sample size for each of the analyses addressing the 3 study objectives.

338x190mm (96 x 96 DPI)

Supplementary Tables:

Supplementary Table S1: Propensity Model Regressing SARS-CoV-2 Status on confounders using multivariable logistic regression

	Adjusted Odds Ratio (95% CI)
**Confounders**	
Age, 10-year increment	1.10 (1.03, 1.19)
Female	1.06 (0.86, 1.30)
Prehospital acid suppression	0.69 (0.54, 0.89)
ICU admission category: medical vs. surgical/trauma	4.34 (2.90, 6.51)
Corticosteroids in the week pre randomization	6.71 (5.33, 8.47)
Days from start of pandemic to enrollment, 1-year increment	0.73 (0.65, 0.81)

Legend Table S1: This stable shows a summary of results of a multivariable logistic regression model (*n*=4477) regressing SARS-CoV-2 status on the *a priori*-selected confounders being used in propensity-matching. SARS-COV-yes (n=467) or SARS-CoV-2-no (n=4010). The model demonstrates significant association between *a priori* selected pre-randomization confounder variables (i.e., age, pre-hospital acid suppression) and SARS-CoV-2 status.

ICU=intensive care unit

For peer review only - http://bmjopen.bmj.com/site/about/guidelines.xhtml

Supplementary Table S2: Baseline Characteristics According to SARS-CoV-2 Status in the propensity-matched cohort

	SARS-CoV-2 Patients	Propensity-matched Non- SARS-CoV-2 Patients (2:1)
Number of patients, n (%)	467	934
Age (years), mean ± SD	61 ± 14	62 ± 13
Female, n (%)	187 (40.0)	365 (39.1)
Prehospital acid suppression, n (%)	104 (22.3)	217 (23.2)
ICU admission category, n (%)		
Medical	440 (94.2)	878 (94.0)
Surgical/trauma	27 (5.8)	56 (6.0)
Corticosteroids in the week pre randomization, n (%)	362 (77.5)	726 (77.7)
Days from start of pandemic to enrollment, mean ± SD	669 ± 280	692 ± 339

Legend Supplementary Table S2: This table shows the baseline characteristics of patients with SARS-CoV-2 and propensity-matched patients without SARS-CoV-2.

SARS-CoV-2=severe acute respiratory syndrome coronavirus-2
SD=standard deviation
ICU=intensive care unit

review only

Supplementary Table S3. Association between SARS-CoV-2 Status and Ventilator-associated Pneumonia

	Confounders adjustment using propensity matching (2:1) (n=1401)		Confounders adjustment using multivariable regressions (n=4477)	
Exploratory Model 1	Adjusted Hazard Ratio (95% CI) SARS-CoV-2 (n=467) vs. non- SARS-CoV-2 (n=934)	p-value	Adjusted Hazard Ratio (95% CI) SARS-CoV-2 (n=467) vs. non- SARS-CoV-2 (n=4010)	p-value
Ventilator-associated Pneumonia[1]	1.08 (0.78, 1.50)	0.63	1.00 (0.75, 1.34)	0.98
Exploratory Model 2	Adjusted Hazard Ratio (95% CI) SARS-CoV-2 (n=157) vs. non- SARS-CoV-2 (n=314)	p-value	Adjusted Hazard Ratio (95% CI) SARS-CoV-2 (n=157) vs. non- SARS-CoV-2 (n=3431)	p-value
Ventilator-associated Pneumonia[2]	0.98 (0.72, 1.35)	0.92	1.09 (0.82, 1.46)	0.55

Legend Supplementary Table S3: This table shows results from 2 exploratory analyses evaluating the association between SARS-CoV-2 status and ventilator-associated pneumonia, using different adjustment methods to account for pneumonia diagnoses on admission to the ICU. The 2 methods of confounder adjustment were: 1) Propensity-matching (2:1) and 2) Cox proportional hazards multivariable regression.

[1]Multivariable regression: adjusted for patients' age, sex, pre-hospital acid suppression, ICU admission category (medical or surgical/trauma), corticosteroids in the week pre randomization, days from start of pandemic to enrollment, randomization group (Pantoprazole vs placebo), and baseline pneumonia. Original Propensity-matched data: adjusted for randomization group (Pantoprazole vs placebo), and baseline pneumonia

[2]Patients with pneumonia at baseline were removed and original propensity scores were re-estimated on the reduced data. Exploratory models 1 were refitted in the reduced data.

SARS-CoV-2=severe acute respiratory syndrome coronavirus-2

Supplementary Table S4. Associations between SARS-CoV-2 Status and Clinical Outcomes, accounting for the competing risk of death

	Confounders adjustment using propensity matching (2:1) (n=1401)		Confounders adjustment using multivariable regressions (n=4477)	
	Adj SHR (95% CI) SARS-CoV-2 (n=467) vs. non- SARS-CoV-2 (n=934)	p-value	Adj SHR (95% CI) SARS-CoV-2 (n=467) vs. non- SARS-CoV-2 (n=4010)	p-value
Clinically important upper GI bleed[1]	0.86 (0.45, 1.64)	0.65	0.82 (0.46, 1.48)	0.52
Incident *C. difficile*[1]	0.69 (0.22, 2.17)	0.53	0.79 (0.28, 2.27)	0.67
New renal replacement therapy[1]	1.30 (0.90, 1.90)	0.17	1.33 (0.95, 1.86)	0.09
Patient important upper GI bleed[1]	0.85 (0.48, 1.51)	0.58	0.85 (0.50, 1.43)	0.54
Ventilator-associated pneumonia[1]	0.54 (0.40, 0.74)	< 0.001	0.51 (0.38, 0.68)	< 0.001
Ventilator-associated pneumonia[2]	1.21 (0.87, 1.67)	0.26	1.11 (0.84, 1.46)	0.48

Legend Supplementary Table S4: This table shows results from exploratory analyses evaluating the association between SARS-CoV-2 status and clinical outcomes, accounting for the competing risk of death using Fine and Gray subdistribution hazard regression models.

[1]Adjusted for patients' age, sex, pre-hospital acid suppression, ICU admission category (medical or surgical/trauma), corticosteroids in the week pre randomization, days from start of pandemic to enrollment, and randomization group (Pantoprazole vs placebo)

[2]Adjusted for variables listed in 1 plus baseline pneumonia

For peer review only

Dennis *et al. Trials* (2023) 24:561
https://doi.org/10.1186/s13063-023-07589-2

Trials

STUDY PROTOCOL

Open Access

Proton pump inhibitors in critically ill mechanically ventilated patients with COVID-19: protocol for a substudy of the Re-EValuating the Inhibition of Stress Erosions (REVISE) Trial

Brittany B. Dennis[1], Lehana Thabane[2,3,4], Diane Heels-Ansdell[2], Joanna C. Dionne[5], Alexandra Binnie[6], Jennifer Tsang[1,6], Gordon Guyatt[5], Aijaz Ahmed[7], François Lauzier[8], Adam Deane[9], Yaseen Arabi[10,11,12], John Marshall[13], Nicole Zytaruk[2,4], Lois Saunders[4], Simon Finfer[14], John Myburgh[14,15], John Muscedere[16], Shane English[17,18], Marlies Ostermann[19], Miranda Hardie[14], Serena Knowles[14], Deborah Cook[4,5*] and For the REVISE Investigators the Canadian Critical Care Trials Group

Abstract

Background Critically ill patients commonly receive proton pump inhibitors (PPIs) to prevent gastrointestinal (GI) bleeding from stress-induced ulceration. Despite widespread use in the intensive care unit (ICU), observational data suggest that PPIs may be associated with adverse outcomes in patients with COVID-19 infection. This preplanned study is nested within a large randomized trial evaluating pantoprazole versus placebo in invasively ventilated patients. The 3 objectives are as follows: (1) to describe the characteristics of patients with COVID-19 in terms of demographics, biomarkers, venous thromboembolism, tracheostomy incidence and timing, and other clinical outcomes; (2) to evaluate the impact of COVID-19 infection on clinically important GI bleeding, 90-day mortality, and other outcomes compared to a propensity-matched non-infected cohort; and (3) to explore whether pantoprazole has a differential treatment effect on clinically important GI bleeding, 90-day mortality, and other outcomes in patients with and without COVID-19 infection.

Methods The ongoing trial Re-EValuating the Inhibition of Stress Erosions (REVISE) compares pantoprazole 40 mg IV to placebo on the primary efficacy outcome of clinically important GI bleeding and the primary safety outcome of 90-day mortality. The protocol described in this report is for a substudy focused on patients with COVID-19 infection that was not in the original pre-pandemic trial protocol. We developed a one-page case report form to characterize these patients including data related to biomarkers, venous thromboembolism, COVID-19 therapies, tracheostomy incidence and timing, duration of mechanical ventilation, and ICU and hospital stay. Our analysis will describe the trajectory of patients with COVID-19 infection, a propensity-matched analysis of infected and non-infected patients, and an extended subgroup analysis comparing the effect of PPI among patients with and without COVID-19 infection.

*Correspondence:
Deborah Cook
debcook@mcmaster.ca
Full list of author information is available at the end of the article

© The Author(s) 2023. **Open Access** This article is licensed under a Creative Commons Attribution 4.0 International License, which permits use, sharing, adaptation, distribution and reproduction in any medium or format, as long as you give appropriate credit to the original author(s) and the source, provide a link to the Creative Commons licence, and indicate if changes were made. The images or other third party material in this article are included in the article's Creative Commons licence, unless indicated otherwise in a credit line to the material. If material is not included in the article's Creative Commons licence and your intended use is not permitted by statutory regulation or exceeds the permitted use, you will need to obtain permission directly from the copyright holder. To view a copy of this licence, visit http://creativecommons.org/licenses/by/4.0/. The Creative Commons Public Domain Dedication waiver (http://creativecommons.org/publicdomain/zero/1.0/) applies to the data made available in this article, unless otherwise stated in a credit line to the data.

Discussion Prophylactic acid suppression in invasively ventilated critically ill patients with COVID-19 infection has unknown consequences. The results of these investigations will inform practice, guidelines, and future research.

Trial registration REVISE Trial [NCT03374800 December 15, 2017], COVID-19 Cohort Study [NCT05715567 February 8, 2023].

Keywords Randomization, Critically ill, COVID-19 pandemic, Hospital transfers, Stress ulceration, Gastrointestinal bleeding, Ventilator-associated pneumonia

Introduction

For patients with COVID-19 receiving invasive mechanical ventilation, standard critical care therapies, including prophylactic acid suppression, have unclear consequences. Concerns emerged early in the pandemic that some common medications may predispose to COVID-19 infection or worsen outcomes among infected patients. For example, observational studies suggested that angiotensin-converting enzyme (ACE) inhibitors were harmful as they were associated with increased mortality [1, 2]. However, this finding was not supported in larger, more robust observational studies [3, 4] or randomized trials [5].

The possible impact of proton pump inhibitors (PPIs) on COVID-19 infection susceptibility and outcomes has also drawn attention. In one observational study from the UK Biobank cohort, PPIs were associated with a 23% reduction in susceptibility to COVID-19 infection (odds ratio [OR] at 1 year = 0.77, 95% confidence interval [CI]: 0.71–0.83), with the greatest protective effects observed with recent use of PPIs (within 6 months) and in people at least 70 years of age [6]. However, these findings of benefit were in contrast with other studies suggesting associated harm. In a retrospective study of Korean national insurance claims including 132,316 patients, short-term use of PPIs was associated with a 79% increased risk of a composite outcome of admission to the ICU, use of mechanical ventilation, and death [7]. These findings were limited by low event rates, reliance on registry data to ascertain PPI exposure, and the use of a composite outcome measure due to insufficient events. In another retrospective case review of 2164 patients with COVID-19 admitted to a US county hospital, PPI use was associated with a 2.8-fold increased risk for death (OR) [95% CI, 1.4–5.5], although findings were not statistically significant following adjustment for age, renal, cardiovascular, and pulmonary comorbidities [8]. Furthermore, a pooled analysis of six retrospective studies of 5884 COVID-19 patients also suggested that PPI use may be associated with an increased risk of death from COVID-19 (risk ratio 1.7, 95% CI 1.02–2.9, $I^2 = 66\%$) [9]. However, all of these study designs generate limited inferences for practice or research purposes.

PPIs are common medications among hospitalized patients, particularly in the intensive care unit (ICU). Whether used pre-hospital or not, critically ill mechanically ventilated patients are usually prescribed PPIs to prevent gastrointestinal (GI) bleeding from stress-induced ulceration [10]. The potential adverse effects of PPI use include ventilator-associated pneumonia (VAP) and *Clostridioides difficile* (*C. difficile*) infection—nosocomial infections that are together more common than GI bleeding and are associated with substantially greater morbidity, mortality, and costs [11]. Herein, we describe a protocol to examine the association between COVID-19 infection and outcomes and PPI use among invasively mechanically ventilated patients nested within an ongoing clinical trial testing pantoprazole versus placebo [12]. This ongoing trial is relevant for patients with COVID-19 infection, given their increased risk of VAP [13], treatment with high-dose corticosteroids [14], and varying anticoagulation intensities [15].

The three objectives of this nested study are as follows: (1) to describe the characteristics of patients with COVID-19 in terms of demographics, biomarkers, COVID-19 treatment, rates of venous thromboembolism, tracheostomy incidence and timing, and other outcomes (e.g., duration of mechanical ventilation, ICU and hospital stay, and mortality); (2) to evaluate the impact of COVID-19 on clinically important GI bleeding, 90-day mortality, infectious, and other outcomes compared to a propensity-matched non-COVID-19 cohort; and (3) to explore whether pantoprazole has a differential treatment effect on clinically important GI bleeding and 90-day mortality, in addition to VAP and *C. difficile* infection and other outcomes, in patients with and without COVID-19.

Methods

Project design for overall REVISE Trial

The Re-EValuating the Inhibition of Stress Erosions (REVISE) is an international randomized stratified, concealed, blinded, parallel-group trial in mechanically ventilated patients, investigating the effect of the PPI pantoprazole 40 mg IV compared to placebo, on the primary *efficacy* outcome of clinically important GI bleeding, the primary *safety* outcome of 90-day mortality, rates of VAP and *C. difficile* infection, patient-important

bleeding, use of renal replacement therapy, and hospital mortality [12]. Led by the Canadian Critical Care Trials Group and the Australian & New Zealand Intensive Care Society Clinical Trials Group, the REVISE Methods Centers are located at McMaster University in Hamilton for international centers and The George Institute in Sydney for Australia. Research Coordinators screen patients from Monday to Friday, using an *a priori* or a consent-to-continue model as locally approved, holding consent encounters with patients if able, or substitute decision-makers in-person or by telephone as needed. Randomization is conducted by research pharmacists to ensure double blinding. Methods for the REVISE trial have been described elsewhere [NCT03374800] [12].

Design for nested COVID cohort study

This study uses a multicenter cohort design embedded within a randomized trial [NCT05715567]. This is the first version (version 1.0; date: June 13, 2023) of the substudy protocol. Figure 1 highlights how it is integrated into the stress ulcer prophylaxis research program. Patients with COVID-19 have been eligible for REVISE since March 11, 2020. Despite the difficulties of continuing non-COVID research in ICUs during the pandemic, recruitment to REVISE was paused for the shortest possible period in each participating center throughout the pandemic. The objectives, a priori hypotheses and outcomes for this COVID-19 study are outlined in Table 1. Although the protocol described in this work would be characterized as a study within a trial, we adhered to the Standard Protocol Items for Clinical Trials (SPIRIT) reporting guidelines where appropriate [16]. Accordingly, the recommended SPIRIT diagram was not included in this work, this will be published as part of the full REVISE randomized trial protocol.

Setting

Patients are being enrolled in 63 ICUs worldwide (39 Canada, 19 Australia, 1 Saudi Arabia, 1 the UK, 1 the US, 1 Kuwait, and 1 Pakistan).

Population

Patients are eligible for this COVID-19 substudy according to the inclusion and exclusion criteria of the REVISE Trial, as outlined in Table 2. Randomization is not stratified by COVID-19 disease status. The inclusion in the COVID-19 cohort will be based on COVID-19 viral nucleic acid test positivity by polymerase chain reaction (PCR) from a nasopharyngeal swab, oropharyngeal swab, sputum, endotracheal aspirate, or bronchoalveolar lavage sample, as clinically determined rather than per protocol. The COVID-19 classification in this study will be made via PCR testing, due to the heightened risk for misclassification using other forms of testing, which will not

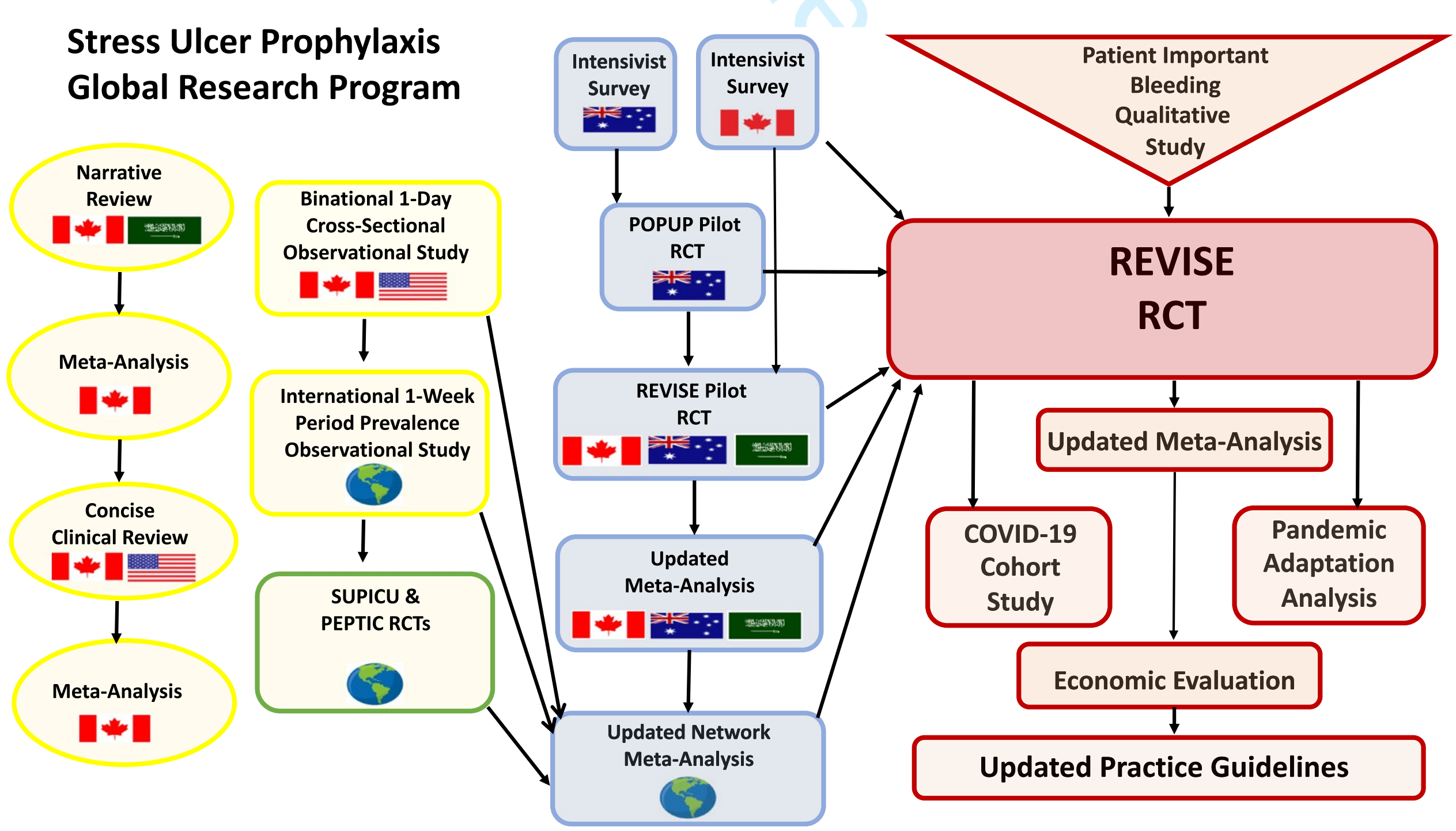

Fig. 1 COVID-19 cohort study within the stress ulcer prophylaxis research program. This figure displays how this COVID cohort study is integrated into the international stress ulcer prophylaxis research program

For peer review only - http://bmjopen.bmj.com/site/about/guidelines.xhtml

Table 1 Overview of research objectives, hypotheses, outcomes, and analytic methods

Objectives	Hypotheses	Outcomes	Methods of analysis
**Question 1:** What prognostically relevant demographic, biomarker, and clinical data characterize invasively ventilated patients with COVID-19? (Explanatory variable: not applicable; no comparisons)			
To establish a clinical profile of critically ill patients with COVID-19, incorporating demographics, biomarkers, rates of venous thromboembolism, COVID-19 therapies, duration of mechanical ventilation, ICU, hospital stay, and tracheostomy timing	Patients with COVID-19 will have a high burden of comorbidity and, as a consequence of their underlying severe viral illness, will exhibit high inflammatory biomarkers (D-dimer, CRP, ferritin), receive targeted COVID-19 therapies, experience long duration of mechanical ventilation, and undergo tracheostomy later than current norms	Description of demographics, biomarkers, and course in the ICU including venous thromboembolism rates and tracheostomy timing	Descriptive analysis<br>Continuous variables will be summarized using mean ± standard deviation (SD), while frequencies and percentages will be used to summarize categorical variables
**Question 2:** Do critically ill patients with COVID-19 have higher rates of clinically important GI bleeding, 90-day mortality, and infection compared to a propensity-matched non-COVID cohort? (explanatory variable: COVID-19)			
To understand the impact of COVID-19 on CIGIB, 90-day mortality, and rates of VAP, *C. difficile* infection, and patient-important bleeding; use of renal replacement therapy; and duration of mechanical ventilation, ICU stay and hospital stay, and ICU and hospital mortality compared to patients without COVID-19	Patients with COVID-19 will have a poorer prognosis than propensity-matched control patients without COVID-19. Patients with COVID-19 will have higher rates of CIGIB, 90-day mortality, VAP, *C. difficile* infection, and patient-important GI bleeding, use of renal replacement therapy, ICU and hospital mortality, and longer durations of mechanical ventilation and ICU and hospital stay, compared to a propensity-matched non-COVID cohort	CIGIB, 90-day mortality<br>VAP, *C. difficile* infection, patient-important bleeding, and renal replacement therapy<br>Duration of mechanical ventilation, ICU stay, and hospital stay<br>ICU and hospital mortality	Propensity-matched analysis of patients in REVISE with and without COVID-19 infection
**Question 3**: Do critically ill patients with COVID-19 projected to receive mechanical ventilation for > 48 h have different clinical outcomes with versus without PPIs? (explanatory variable: pantoprazole)			
To examine whether pantoprazole has a different treatment effect on CIGIB or 90-day mortality in patients with and without COVID-19 (REVISE subgroup analysis)<br>To determine whether pantoprazole has a differential treatment effect in patients with or without COVID-19 on VAP, *C. difficile* infection, patient-important GI bleeding, use of renal replacement therapy, hospital mortality, and duration of mechanical ventilation, ICU stay and hospital stay, and ICU and hospital mortality	We hypothesize no modification of the effect of pantoprazole on CIGIB. We hypothesize that pantoprazole is more harmful in patients with versus without SARS-CoV-2 in terms of increased risk of 90-day mortality<br>We hypothesize no modification of the effect of pantoprazole on VAP, *C. difficile* infection, patient-important GI bleeding, use of renal replacement therapy, and duration of mechanical ventilation, ICU stay and hospital stay, and ICU and hospital mortality. We hypothesize that pantoprazole is more harmful in patients with versus without SARS-CoV-2 in terms of increased risk of hospital mortality	CIGIB, 90-day mortality (REVISE subgroup analysis)<br>VAP, *C. difficile* infection, patient-important bleeding, and renal replacement therapy (extended outcomes)<br>Duration of mechanical ventilation, ICU stay and hospital stay, and ICU and hospital mortality (extended outcomes)	Subgroup analysis: Comparison of outcomes for patients with and without COVID-19 receiving either pantoprazole or placebo<br>Comparison of the time to the primary and secondary binary outcomes in the two groups using Cox proportional hazards regression with threshold *P*-values of 0.05, adjusted for the center and pre-hospital use of acid suppression (stratification variables). A test of interaction will be performed for this subgroup analysis in REVISE, and the results will be interpreted per the ICEMAN criteria<br>For binary outcomes, we will report hazard ratios with 95% CIs as well as the absolute risk increase or decrease and 95% CIs<br>For continuous outcomes, we will compare the two arms using a *t*-test on the log scale (to normalize the distribution) or a nonparametric approach if needed

Research question, objectives, hypotheses, outcomes, and analytic approaches for the COVID-19 REVISE substudy

CIGIB clinically important GI bleeding, *ICU* intensive care unit, *PPI* proton pump inhibitor, *COVID-19* coronavirus disease of 2019, *VAP* ventilator-associated pneumonia, *C. difficile Clostridioides difficile*

For peer review only

Table 2 Inclusion and exclusion criteria for REVISE

**Inclusion criterion**	
Adults ≥ 18 years old projected to receive invasive mechanical ventilation for ≥ 48 h according to the treating physician	
**Exclusion criteria**	
1. Already received invasive mechanical ventilation ≥ 72 h during this hospital admission	
2. Acid suppression for active GI bleeding or high risk of bleeding (e.g., current bleeding, peptic ulcer bleeding within 8 weeks, recent severe esophagitis, Barrett's esophagus, Zollinger-Ellison syndrome) [dyspepsia or gastroesophageal reflux is not an exclusion criterion]	
3. Acid suppression in the ICU for > 1 daily dose equivalent of a PPI or H2RA	
4. Dual antiplatelet therapy	
5. Combined antiplatelet and therapeutic anticoagulation	
6. Pantoprazole contraindication per local product information	
7. Palliative care or anticipated withdrawal of advanced life support	
8. Pregnancy	
9. Previous enrollment in REVISE, a related trial, or trial for which co-enrollment is prohibited	
10. Patient, proxy, or physician declines	

In this table, we list the eligibility criteria for this study, based on the inclusion and exclusion criteria for the REVISE trial

GI gastrointestinal, *ICU* intensive care unit, *PPI* proton pump inhibitor, *H2RA* histamine-2-receptor antagonists

be accepted for COVID-19 diagnosis, including antigen testing. Patients will be eligible for this substudy if they test positive for SARS-CoV-2 in the month preceding the index ICU admission, whether pre-hospital, in hospital, or in the ICU. Patients diagnosed as contracting SARS-CoV-2 more than 48 h after enrollment in REVISE will be ineligible.

Summary of outcomes for REVISE

The primary *efficacy* outcome for REVISE is clinically important GI bleeding occurring in the ICU or resulting in ICU readmission during the index hospital stay. Adjudicator agreement for this metric was excellent in two prior studies [17, 18] which was associated with increased attributable length of stay and mortality [19]. The definition of clinically important GI bleeding is overt GI bleeding (i.e., hematemesis, frank blood or coffee ground nasogastric aspirate, melena or hematochezia) *plus 1 of the following in the absence of other causes*: (1) hemodynamic change defined as a spontaneous decrease in invasively monitored mean arterial pressure or non-invasive systolic or diastolic blood pressure of ≥ 20 mmHg or an orthostatic increase in pulse rate of ≥ 20 beats/min and a decrease in systolic blood pressure of ≥ 10 mmHg, with or without vasopressor initiation or increase; (2) vasopressor initiation; (3) hemoglobin decrease of ≥ 2 g/dL (20 g/L) within 24 h of bleeding; (4) transfusion of ≥ 2 units red blood cells within 24 h of bleeding; or (5) therapeutic intervention (e.g., therapeutic endoscopy, angioemoblization, surgery). The primary *safety* outcome is mortality defined as all-cause mortality at 90 days after randomization to evaluate a suggestion of increased risk of death associated with pantoprazole among the sickest subgroup of patients in a recent placebo-controlled trial [20]. Secondary outcomes for REVISE are listed in Table 3.

Table 3 Secondary outcome definitions in REVISE

Ventilator-associated pneumonia	VAP is diagnosed in patients who received invasive mechanical ventilation for ≥ 48 h when there is a new, progressive, or persistent radiographic infiltrate *plus at least 2 of the following without other obvious cause*: (1) fever (temperature > 38 °C) or hypothermia (temperature < 36 °C), (2) leukopenia (< 4.0×10^6/L) or leukocytosis (> 12×10^6/L), (3) purulent sputum, or (4) gas exchange deterioration. Given no universally accepted VAP criteria, data collection and central adjudication allows analysis of several other VAP definitions. We do not collect infection-related ventilator-associated conditions as they are modifiable by ventilator settings and volume status
C. difficile infection	Defined as clinical features (diarrhea [≥ 3 episodes of unformed stools or Bristol type 6 or 7], ileus, or toxic megacolon) and either microbiological evidence of toxin-producing *C. difficile* or pseudomembranous colitis on colonoscopy in hospital
Patient-important GI bleeding	Defined as overt GI bleeding, *plus* an invasive intervention (e.g., therapeutic endoscopy, angioembolization, surgery), acknowledging how some clinically important GI bleeds in prior studies did not actually require any tests or treatments, and thus may not be important to patients, to be refined following completion of patient and family interviews from a Patient Important Bleeding Study [NCT05506150]
Renal replacement therapy	Defined as the initiation of new renal replacement therapy in the ICU
Hospital mortality	Defined as all-cause mortality in the hospital

In this table, we show the secondary outcomes and their definitions for the REVISE trial

VAP ventilator-associated pneumonia, *ICU* intensive care unit, *GI* gastrointestinal, *C. difficile Clostridioides difficile*

For peer review only

Pilot work

Investigators developed a one-page draft case report form to capture new information which was not part of the original REVISE trial to better characterize the COVID-19 cohort. A group of eight research coordinators reviewed and refined the form to capture COVID-19 vaccination status, biomarkers, venous thromboembolic events, tracheostomy occurrence and timing, and COVID-19 therapies as per Table 4. The form was pre-tested on 15 patients by abstracting data from either electronic or paper-based medical records. Accessing the chart, scanning contents, extracting relevant information, and entering data into the password-protected REVISE website required an estimated 40 min per patient. After this pilot exercise, we oriented research coordinators in participating centers to the study objectives, methods, and the case report form during telephone, videoconference, and in-person investigators' meetings.

Data collection and outcomes for the COVID-19 cohort study

Retrospective data collection for newly added variables for the COVID-19 study was required for participants enrolled prior to the implementation of this COVID-19 study protocol. Following relevant ethics review, COVID-19-specific variables were incorporated into existing data-collection forms, affording prospective data collection for COVID-19 variables for the remainder of the study. For any patients transferred to non-REVISE centers during the pandemic, all efforts are being made to complete the COVID-19 case report form, including a collection of data on outcomes occurring after transfer to a non-REVISE center.

Biologic specimens (e.g., sputum culture for COVID-19 testing) and laboratory results (blood samples to evaluate for hemoglobin levels) are collected as part of routine medical care. These samples are not being procured or stored for study purposes.

Data management and quality control

Data management and quality control will be overseen by the REVISE Methods Centers, located at McMaster University in Hamilton and The George Institute (TGI) in Sydney. These centers are responsible for ensuring optimal trial conduct. Oversight and quality control will be achieved through close collaboration between these centers, who hold twice monthly teleconferences to harmonize approaches and share management efficiencies. The Methods Center Leads and relevant investigators agreed to train local principal investigator(s), research coordinator(s), and research pharmacist(s), on the protocol. Throughout the trial, the methods center staff will engage in compliance-enhancing strategies, set targets, and track progress. REVISE analyst Ms. D Heels-Ansdell will conduct periodic central statistical monitoring to generate overall and site-specific metrics for internal use and site feedback. The methods center staff will adopt a risk-adapted approach to monitor each site (in-person or by videoconference per pandemic adaptations) to assess and enhance protocol fidelity. The foci will include eligibility adherence, stratification, informed consent documentation, regulatory compliance, and case report form accuracy.

In the event of any modifications made to the existing protocol for the COVID-19 substudy, the REVISE methods center will take responsibility for notifying all participating centers, sponsors, and funding institutions. All participating centers would receive a copy of the revised protocol and meet to review and discuss any implications of such changes to study implementation.

Sample size

The cohort size is based on the number of patients with COVID-19 enrolled in REVISE. As of June 1, 2023, 484 patients with COVID-19 have been enrolled in REVISE, representing 10.1% of the sample size of 4800 patients. Acknowledging the inherent challenges in predicting the incidence of and the means ± standard deviation (SD)

Table 4 COVID cohort substudy biomarkers and other clinical characteristics

New variables	Definitions and measures
**D-dimer**	Highest D-dimer level (μg/L) recorded post-COVID diagnosis
**C-reactive protein**	Highest C-reactive protein level (mg/L) recorded post-COVID diagnosis
**Ferritin level**	Highest ferritin level (μg/L) recorded post-COVID diagnosis
**Venous thromboembolism**	Defined as (1) new symptomatic or asymptomatic deep-vein thrombosis or pulmonary embolism, diagnosed using accepted imaging tests in practice
**Days of mechanical ventilation**	Defined as the number of days requiring mechanical ventilation either invasive or non-invasive
**Tracheostomy and timing of insertion**	Defined as time from intubation to tracheostomy insertion during the ICU stay

In this table, we list the additional data required for this COVID-19 C cohort study

COVID-19 coronavirus disease of 2019, *ICU* intensive care unit

For peer review only - http://bmjopen.bmj.com/site/about/guidelines.xhtml

or median (interquartile range [IQR]) of the continuous variables will be calculated. Baseline characteristics (e.g., age, sex, APACHE II Score, admission diagnostic category, comorbidities, COVID-19 vaccination status, and pre-hospital acid suppression) will be described. Table 1 presents the statistical analysis plans for the 3 study objectives, hypotheses, and outcomes.

For any outcome that is missing for more than 2% of patients, we will perform multiple imputation. Adjustments for multiple comparisons will not be made for these exploratory analyses. All analyses will be performed using the statistical software SAS version 9.4 [21].

Analysis of objective 1: Characterization of the COVID-19 cohort

New continuous variables added to the REVISE dataset to help characterize the COVID-19 cohort of patients include inflammatory biomarker levels and time until tracheostomy. New dichotomous variables include the incidence of thromboembolic events and treatment with COVID-19-specific therapies. Continuous variables will be summarized using mean ± standard deviation (SD), or median (interquartile range [IQR]), while frequencies and percentages will be used to summarize categorical variables.

Analysis of objective 2: Propensity-matched comparison of patients with and without COVID-19

To explore whether patients with COVID-19 experience different clinical outcomes relative to patients without COVID-19, we will compare the COVID-19 cohort and a propensity-matched non-COVID cohort from REVISE. Propensity score matching attempts to estimate the effect of COVID-19 status by accounting for important covariates that may predict or explain this status. Patients with COVID-19 will be matched with other patients within REVISE using propensity score analysis. We will consider an optimal matching ratio of 2:1 or 1:1 depending on the availability of optimal matches.

The propensity score will be generated using potentially important pre-randomization demographic variables, including age, sex, pre-hospital acid suppression (PPIs or histamine-2-receptor antagonists (H2RAs)), COVID-19 vaccination status, ICU admission category of medical or surgical/trauma, and date of enrollment. Propensity matching will only include patients enrolled after the declaration of the COVID-19 pandemic on March 11, 2020. As a sensitivity analysis, we will match only on pre-hospital PPI use, not H2RAs, given limited evidence to suggest that H2RAs influence susceptibility to COVID-19 infection [22].

Propensity score matching helps to ensure that the distribution of the foregoing covariates is similar between the compared populations [23]. Comparisons between the COVID-19 and matched cohorts will be made on clinically important bleeding and 90-day mortality, incidence of VAP, *C. difficile* infection, patient-important GI bleeding, renal replacement therapy, ICU and hospital mortality, and duration of mechanical ventilation and ICU and hospital stay. We will adjust all analyses for randomized allocation; mortality outcomes will be adjusted for the APACHE II score.

Objective 3: Treatment effect of PPIs in patients with COVID-19 versus other patients

We will explore whether pantoprazole in the ICU has a differential treatment effect on clinically important GI bleeding or 90-day mortality for patients with COVID-19 relative to those without COVID-19. This is a subgroup analysis of the primary REVISE trial. However, in addition, we aim to further evaluate the subgroup effects of pantoprazole in patients with COVID-19 across secondary REVISE outcomes including VAP and *C. difficile* infection, patient-important bleeding, renal replacement therapy, and ICU and hospital mortality, as well as the duration of mechanical ventilation and ICU and hospital stay.

This analysis will be conducted per the intention-to-treat principle. For time to the primary and secondary binary outcomes, we will use Cox proportional hazards regression, adjusted for pre-hospital use of acid suppression (a stratification variable). Analysis of our mortality outcomes will also be adjusted for the APACHE II score. An interaction term will be included in the model to test for this subgroup effect and results will be interpreted per the ICEMAN criteria [24]. We will report hazard ratios with 95% CIs as well as the absolute risk increase or decrease and 95% CIs. For continuous outcomes, we will perform linear regression which will include an interaction term. We will report mean differences with 95% CIs. If needed, we will perform the linear regression on the log-transformed outcome.

Ethics and regulatory approval

The main REVISE trial was approved in participating hospitals by all relevant ethics committees and regulatory authorities prior to starting enrollment. The Hamilton Integrated Research Ethics Board (HiREB) under Clinical Trials Ontario (CTO Study ID 1360) is the main REB of record, which also approved this substudy. Ethics review for the additional COVID-19-specific data collection was undertaken by the Research Ethics Board of record

For peer review only

(the Hamilton Integrated Research Ethics Board), and as needed to comply with local regulations in each participating center. In the absence of any modifications to the REVISE study design, this substudy involved additional data collection to characterize the patients with COVID-19 and facilitate additional future analyses. Therefore, no modifications were made to the consenting process for REVISE patients involved in this embedded COVID-19 study, and no requirements were issued to either reconsent or inform prior REVISE participants of the substudy. Specifically, there was no requirement to inform participants of this substudy, including an additional review of participants' medical records for new data collection. Participants previously randomized in REVISE were not re-consented to participate in the COVID-19 embedded substudy.

Integrated knowledge translation plan

After completion of the COVID-19 REVISE substudy, we will rapidly publish the results. All results will be published with open access to ensure wide dissemination and accessibility of study findings. We plan to publish and present the COVID-19 substudy work at an international congress. We will disseminate a lay summary of findings to the general public through health networks of patient advocates and persons with lived or living experiences. We will generate lay language summaries and visual abstracts for traditional (paper, radio, television) and social media. We will also host videoconferences and regional rounds. We will disseminate structured abstracts and slide decks to local ICU quality councils, provincial organizations (e.g., Alberta Health Services Critical Care Strategic Clinical Network), national policymakers (e.g., Accreditation Canada), and professional societies. Participating organizations, sponsors, and university institutions will be provided with press release briefs to disseminate to their respective audiences.

Discussion

This study, embedded within a global, randomized trial of stress ulcer prophylaxis, will fill important knowledge gaps in the literature that prompted our three research questions and three study objectives. The first is a descriptive analysis of the COVID-19 cohort in REVISE. The second is a propensity-matched analysis of patients with and without COVID-19. The third is an extended subgroup analysis of the REVISE trial, evaluating the effect of pantoprazole on a range of additional clinical outcomes among patients with and without COVID-19.

Evidence describing the association between acid suppression and poor prognosis among patients with COVID-19 has emerged, but results have been variable. To prevent complications among critically ill patients with COVID-19, interim WHO guidance in 2020 suggested H2RAs or PPIs for patients with risk factors for GI bleeding, including mechanical ventilation for ≥ 48 h, coagulopathy, renal replacement therapy, liver disease, multiple comorbidities, and higher organ failure score [25]. Surviving Sepsis Guidelines for the management of critically ill patients with COVID-19 do not address stress ulcer prophylaxis [26]. Randomized trial data on the effect of PPIs among patients with COVID-19 is insufficient to guide clinical practice, and this study will provide direct evidence on this topic.

Limitations of this substudy include the risk of missing 90-day outcomes due to high levels of inter-hospital transfers experienced during COVID-19 [27]. Patients transferred to centers not participating in REVISE or other loss to follow-up. Our propensity analysis cannot adjust for all possible factors predisposing to infection with SARS-CoV-2. We will have no information on patients eligible for REVISE who were not approached for the trial due to concerns about bleeding risk due to COVID-19 therapies or increased risk of VAP due to COVID-19 infection itself. In addition, the number of patients enrolled in REVISE with COVID-19 depends on the pandemic status in participating centers, such that no sample size calculation is possible; thus, we will interpret the findings in light of the 95% confidence intervals.

Strengths of this study include the timely development of a protocol addressing essential research questions relevant to a high-risk subgroup of critically ill mechanically ventilated patients that emerged during the pandemic. The main REVISE Trial [NCT03374800], and this COVID-19 Cohort Study [NCT05715567] are registered. We have specified a priori hypotheses for each of the 3 objectives. As this study was developed after the REVISE Trial began recruitment, we are using both retrospective and prospective data collection methods to identify relevant patients with COVID-19. All necessary data is derived from the hospital stay, except 90-day mortality for those discharged alive; thus, we anticipate minimal missing data.

Trial status

As of June 1, 2023, more than 90% of relevant data for REVISE patients with COVID-19 are in the REVISE database to inform this work. REVISE enrollment is anticipated to close byNovember 2023; 90-day follow-up, chart closure, and analyses for this study will follow later in 2024.

For peer review only - http://bmjopen.bmj.com/site/about/guidelines.xhtml

Acknowledgements
The trial was designed by the REVISE Steering Committee including the National and International Management Committees, the REVISE Investigators and Research Coordinators, the Canadian Critical Care Trials Group, and the Australian and New Zealand Intensive Care Society Clinical Trials Group. We are grateful to others at the Methods Center at McMaster University (Lisa Buckingham, France Clarke, Mary Copland, Karlo Matic, Ashley Sawyer, Alyson Takaoka) and The George Institute (Fatima Butt, Anna Cheng, Conrad Nangla, Fiona Osborne, Tina Schneider, Isabella Schoeler, Anna Tippet) for their expertise.

Authors' contributions
Concept and design: Y Arabi, A Binnie, D Cook, A Deane, B Dennis, J Dionne, S English, S Finfer, G Guyatt, M Hardie, D Heels-Ansdell, S Knowles, F Lauzier, J Marshall, J Muscedere, J Myburgh, M Ostermann, L Thabane, J Tsang, and N Zytaruk. Acquisition, analysis, or interpretation of the data: D Cook, B Dennis, M Hardie, D Heels-Ansdell, S Knowles, L Saunders, L Thabane, and N Zytaruk. Drafting of the manuscript: D Cook, B Dennis, D Heels-Ansdell, and N Zytaruk. Statistical analysis: B Dennis, D Heels-Ansdell, and L Thabane. Obtained funding: A Ahmed, A Binnie, D Cook, B Dennis, J Dionne, G Guyatt, D Heels-Ansdell, L Thabane, and J Tsang. Administrative, technical, or material support: D Cook, A Deane, B Dennis, S Finfer, G Guyatt, M Hardie, D Heels-Ansdell, S Knowles, F Lauzier, D Rajbhandari, L Saunders, A Poole, L Thabane, and N Zytaruk. Data integrity: D Cook, B Dennis, M Hardie, D Heels-Ansdell, S Knowles, L Saunders, and N Zytaruk. Ethics approval and consent to participate: not applicable for this retrospective chart review protocol. Approved final manuscript: all authors.

Funding
REVISE is funded by peer-reviewed grants [Canadian Institutes of Health Research 201610PJT-378226-PJT-CEBA-18373 and Canadian Institutes of Health Research 202207CL3-492565-CTP-CEBA-19215], the Hamilton Academy of Health Sciences Organization [HAH-22–009], Physician Services Incorporated (R22-33) and funds from St. Joseph's Healthcare Hamilton and McMaster University. The National Health and Medical Research Council of Australia grant [GNT1124675] funds enrollment in Australia. REVISE was approved by the National Institute for Health Research (NIHR) in the UK as a Portfolio Study [CPMS ID 45782], eligible for support from the NIHR Clinical Research Network. [https://www.nihr.ac.uk/researchers/collaborations-services-and-support-for-your-research/run-your-study/crn-portfolio.htm]. This trial received no support from the commercial or private sector. The funders/sponsors have no role in the conception, design, conduct, oversight, analysis, interpretation, write-up, review, or approval of the manuscript or the decision to submit the manuscript for publication.
Role of the funders: The funders/sponsors have no role in the conception, design, conduct, oversight, analysis, interpretation, write-up, review, or approval of the manuscript or the decision to submit the manuscript for publication.
Composition and responsibilities of participating centers and steering committee: Led by the Canadian Critical Care Trials Group and the Australian & New Zealand Intensive Care Society Clinical Trials Group, the REVISE Methods Centers are located at McMaster University in Hamilton for international centers and The George Institute in Sydney for Australia. These centers (REVISE Methods Centre, The George Institute) housed the primary data management and endpoint adjudication teams, who worked in close collaboration to ensure standardization of data collection and adjudication procedures. The REVISE Canadian Steering Committee comprises Drs. Guyatt, Cook, Alhazzani, Marshall, Lauzier, English, Muscedere, Hall, Xie, and Thabane. The International Management Committee includes Drs. Finfer and Myburgh, Deane, Ostermann, and Arabi joined by Canadian leaders Drs. Guyatt, Cook, Alhazzani, Marshall, and Thabane. These teams meet approximately quarterly, to anticipate and pre-empt challenges and generate solutions to the operational and financial hurdles characterizing global trials. The Data Safety & Monitoring Committee includes Professor Roberts (University of Oxford), Dr. McAuley (Queen's University Belfast), and Chair Dr. Thomlinson (University of Toronto).

Availability of data and materials
Readers are welcome to contact the research team for further information. There are no original data associated with this research protocol.
The datasets analyzed during the current study would only be available following relevant research ethics board discussion and approval. However, the statistical code will be available and any requested details of the protocol.

Declarations

Ethics approval and consent to participate
The main REVISE trial was approved in participating hospitals by all relevant ethics committees and regulatory authorities prior to starting enrollment. The Hamilton Integrated Research Ethics Board (HiREB) under Clinical Trials Ontario (CTO Study ID 1360) is the main REB of record, which also approved this substudy. Ethics review for the additional COVID-19-specific data collection was undertaken as needed to comply with local regulations in each participating center.

Consent for publication
Not applicable for this protocol.

Competing interests
All authors are involved in the REVISE Trial in some capacity. Otherwise, the authors declare that they have no competing interests.

Author details
[1]Department of Medicine, McMaster University, Hamilton, ON, Canada. [2]Department of Health Research Methods, Evidence, and Impact, McMaster University, Hamilton, ON, Canada. [3]Biostatistics Unit, St. Joseph's Healthcare Hamilton, Hamilton, ON, Canada. [4]Division of Critical Care, Research Institute, St. Joseph's Healthcare Hamilton, Hamilton, ON, Canada. [5]Departments of Medicine and Health Research Methods, Evidence, and Impact, McMaster University, McMaster University Health Sciences Center, Room 2C11, 1200 Main Street West, Hamilton, ON L8N 3Z5, Canada. [6]Department of Critical Care Medicine, Niagara Health System, St. Catharines, ON, Canada. [7]Department of Gastroenterology and Hepatology, Stanford University, Palo Alto, CA, USA. [8]Departments of Anesthesiology and Medicine and Critical Care Medicine, Université Laval, Québec, Québec, Canada. [9]Department of Critical Care Medicine, University of Melbourne, Melbourne Medical School, Parkville, VIC, Australia. [10]Intensive Care Department, Ministry of the National Guard-Health Affairs, Riyadh, Kingdom of Saudi Arabia. [11]King Saud Bin Abdulaziz University for Health Sciences, Riyadh, Kingdom of Saudi Arabia. [12]King Abdullah International Medical Research Center, Riyadh, Kingdom of Saudi Arabia. [13]Interdepartmental Division of Critical Care, University of Toronto, Toronto, ON, Canada. [14]Critical Care Program, The George Institute for Global Health, Faculty of Medicine, University of New South Wales, Sydney, NSW, Australia. [15]Intensive Care Unit, St. George Hospital, Sydney, Australia. [16]Department of Critical Care Medicine, Queen's University, Kingston, ON, Canada. [17]Department of Medicine, University of Ottawa, Ottawa, ON, Canada. [18]Clinical Epidemiology Program, Ottawa Hospital Research Institute, Ottawa, ON, Canada. [19]Department of Critical Care, King's College London, Guy's & St Thomas' Hospital, London, UK.

Received: 3 July 2023 Accepted: 16 August 2023
Published online: 30 August 2023

References
1. Richardson S, Hirsch JS, Narasimhan M, Crawford JM, McGinn T, Davidson KW, Barnaby DP, Becker LB, Chelico JD, Cohen SL, et al. Presenting characteristics, comorbidities, and outcomes among 5700 patients hospitalized with COVID-19 in the New York City area. JAMA. 2020;323(20):2052.
2. Guan W-J, Ni Z-Y, Hu Y, Liang W-H, Ou C-Q, He J-X, Liu L, Shan H, Lei C-L, Hui DSC, et al. Clinical characteristics of coronavirus disease 2019 in China. N Engl J Med. 2020;382(18):1708–20.
3. Zhang P, Zhu L, Cai J, Lei F, Qin J-J, Xie J, Liu Y-M, Zhao Y-C, Huang X, Lin L, et al. Association of inpatient use of angiotensin-converting enzyme inhibitors and angiotensin II receptor blockers with mortality among patients with hypertension hospitalized with COVID-19. Circ Res. 2020;126(12):1671–81.
4. Reynolds HR, Adhikari S, Pulgarin C, Troxel AB, Iturrate E, Johnson SB, Hausvater A, Newman JD, Berger JS, Bangalore S, et al. Renin–angiotensin–aldosterone system inhibitors and risk of COVID-19. N Engl J Med. 2020;382(25):2441–8.
5. Lopes RD, Macedo AVS, De BarrosESilva PGM, Moll-Bernardes RJ, Dos Santos TM, Mazza L, Feldman A, D'AndréaSabaArruda G, De Albuquerque

For peer review only

DC, Camiletti AS, et al. Effect of discontinuing vs continuing angiotensin-converting enzyme inhibitors and angiotensin II receptor blockers on days alive and out of the hospital in patients admitted with COVID-19. JAMA. 2021;325(3):254.
6. Xiang Y, Wong KC, So HC. Exploring drugs and vaccines associated with altered risks and severity of COVID-19: a UK Biobank cohort study of all ATC level-4 drug categories reveals repositioning opportunities. Pharmaceutics. 2021;13(9):1514.
7. Lee SW, Ha EK, Yeniova AÖ, Moon SY, Kim SY, Koh HY, Yang JM, Jeong SJ, Moon SJ, Cho JY, et al. Severe clinical outcomes of COVID-19 associated with proton pump inhibitors: a nationwide cohort study with propensity score matching. Gut. 2021;70(1):76–84.
8. Aby ES, Tappata M, Rodin H, Debes JD. Assessment of proton-pump inhibitor use, race, socioeconomic status, and mortality in individuals with COVID-19. Eur J Gastroenterol Hepatol. 2022;34(2):239.
9. Hariyanto TI, Prasetya IB, Kurniawan A. Proton pump inhibitor use is associated with increased risk of severity and mortality from coronavirus disease 2019 (COVID-19) infection. Dig Liver Dis. 2020;52(12):1410–2.
10. Krag M, Perner A, Wetterslev J, Wise MP, Borthwick M, Bendel S, McArthur C, Cook D, Nielsen N, Pelosi P, et al. Prevalence and outcome of gastrointestinal bleeding and use of acid suppressants in acutely ill adult intensive care patients. Intensive Care Med. 2015;41(5):833–45.
11. Cook D, Guyatt G. Prophylaxis against upper gastrointestinal bleeding in hospitalized patients. N Engl J Med. 2018;378(26):2506–16.
12. Re-EValuating the Inhibition of Stress Erosions (REVISE) Trial (REVISE), [NCT03374800]. Clinical trial registry from clinicalTrials.gov; 2018. https://classic.clinicaltrials.gov/ct2/show/NCT03374800. Accessed 28 Aug 2023.
13. Ippolito M, Misseri G, Catalisano G, Marino C, Ingoglia G, Alessi M, Consiglio E, Gregoretti C, Giarratano A, Cortegiani A. Ventilator-associated pneumonia in patients with COVID-19: a systematic review and meta-analysis. Antibiotics (Basel). 2021;10(5):545.
14. Dexamethasone in hospitalized patients with COVID-19. N Engl J Med. 2020;384(8):693–704. https://www.nejm.org/doi/full/10.1056/NEJMoa2021436.
15. Therapeutic anticoagulation with heparin in critically ill patients with COVID-19. N Engl J Med. 2021;385(9);777–89. https://www.nejm.org/doi/full/10.1056/NEJMoa2021436.
16. Chan AW, Tetzlaff JM, Altman DG, Laupacis A, Gøtzsche PC, Krleža-Jerić K, Hróbjartsson A, Mann H, Dickersin K, Berlin JA, et al. SPIRIT 2013 statement: defining standard protocol items for clinical trials. Ann Intern Med. 2013;158(3):200–7.
17. Cook D, Guyatt G, Marshall J, Leasa D, Fuller H, Hall R, Peters S, Rutledge F, Griffith L, McLellan A, et al. A comparison of sucralfate and ranitidine for the prevention of upper gastrointestinal bleeding in patients requiring mechanical ventilation. N Engl J Med. 1998;338(12):791–7.
18. Cook DJ, Fuller HD, Guyatt GH, Marshall JC, Leasa D, Hall R, Winton TL, Rutledge F, Todd T, Roy P, et al. Risk factors for gastrointestinal bleeding in critically ill patients. N Engl J Med. 1994;330(6):377–81.
19. Cook DJ, Griffith LE, Walter SD, Guyatt GH, Meade MO, Heyland DK, Kirby A, Tryba M. Crit Care. 2001;5(6):368.
20. Krag M, Marker S, Perner A, Wetterslev J, Wise MP, Schefold JC, Keus F, Guttormsen AB, Bendel S, Borthwick M, et al. Pantoprazole in patients at risk for gastrointestinal bleeding in the ICU. N Engl J Med. 2018;379(23):2199–208.
21. SAS 9.4. In. Edited by Inc SI. Cary, NC.
22. Hirota K. H_2 antagonists, proton pump inhibitors and COVID-19. J Anesth. 2022;36(3):329–31.
23. Rosenbaum PR, Rubin DB. The central role of the propensity score in observational studies for causal effects. Biometrika. 1983;70(1):41–55.
24. Schandelmaier S, Briel M, Varadhan R, Schmid CH, Devasenapathy N, Hayward RA, Gagnier J, Borenstein M, van der Heijden GJMG, Dahabreh IJ, et al. Development of the Instrument to assess the Credibility of Effect Modification Analyses (ICEMAN) in randomized controlled trials and meta-analyses. Can Med Assoc J. 2020;192(32):E901–6.
25. World Health O. Clinical management of severe acute respiratory infection (SARI) when COVID-19 disease is suspected: interim guidance, 13 March 2020. In. Geneva: World Health Organization; 2020.
26. Alhazzani W, Evans L, Alshamsi F, Møller MH, Ostermann M, Prescott HC, Arabi YM, Loeb M, Ng Gong M, Fan E, et al. Surviving sepsis campaign guidelines on the management of adults with coronavirus disease 2019 (COVID-19) in the ICU: first update. Crit Care Med. 2021;49(3):e219–34.
27. Pett E, Leung HL, Taylor E, Chong MSF, Hla TTW, Sartori G, Sathianathan V, Husain T, Suntharalingam G, Rosenberg A, et al. Critical care transfers and COVID-19: managing capacity challenges through critical care networks. J Intensive Care Soc. 2022;23(2):203–9.

Publisher's Note

Springer Nature remains neutral with regard to jurisdictional claims in published maps and institutional affiliations.

Ready to submit your research? Choose BMC and benefit from:

- fast, convenient online submission
- thorough peer review by experienced researchers in your field
- rapid publication on acceptance
- support for research data, including large and complex data types
- gold Open Access which fosters wider collaboration and increased citations
- maximum visibility for your research: over 100M website views per year

At BMC, research is always in progress.

Learn more biomedcentral.com/submissions

For peer review only

BMJ Open

Proton Pump Inhibitors in Invasively Ventilated Patients with SARS-CoV-2: A Substudy of the Re-EValuating the Inhibition of Stress Erosions (REVISE) Trial

Journal:	*BMJ Open*
Manuscript ID	bmjopen-2026-119937.R1
Article Type:	Original research
Date Submitted by the Author:	21-Jul-2026

Complete List of Authors:	Dennis, Brittany; The University of British Columbia, Department of Medicine<br>Heels-Ansdell, Diane; McMaster University, Department of Health Research Methods, Evidence and Impact<br>Quazi, Ibrahim; McMaster University, Department of Health Research Methods, Evidence and Impact<br>Basmaji, John; Western University, Department of Medicine, Division of Critical Care<br>Thabane, Lehana; McMaster University, Department of Health Research Methods, Evidence, and Impact; St. Joseph's Research Institute; St Joseph's Healthcare Hamilton, Biostatistics Unit<br>Guyatt, Gordon; McMaster University, Department of Medicine, Health Research Methods, Evidence and Impact,<br>Saunders, Lois; St. Joseph's Healthcare , Research Institute<br>Hand, Lori; McMaster University, Department of Health Research Methods, Evidence and Impact<br>Loy, Olivia; The Royal Melbourne Hospital, Department of Critical Care<br>Zytaruk, Nicole; McMaster University, Health Research Methods, Evidence & Impact; St. Joseph's Healthcare, Research Institute, Division of Critical Care<br>Hardie, Miranda; University of New South Wales, The George Institute for Global Health, Critical Care Program, Faculty of Medicine<br>Deane, Adam; The University of Melbourne, Department of Critical Care, Melbourne Medical School; The University of Melbourne - Parkville Campus, Intensive Care Unit, Department of Critical Care Medicine<br>Marshall, John; University of Toronto, Interdepartmental Division of Critical Care<br>Arabi, Yaseen; Ministry of National Guard Health Affairs, Intensive Care Department; King Saud bin Abdulaziz University for Health Sciences; King Abdullah International Medical Research Center<br>Lauzier, François; Laval University, Departments of Anesthesiology, Medicine and Critical Care Medicine<br>Dionne, Joanna; McMaster University, Departments of Medicine, Health Research Methods, Evidence, and Impact,<br>Burns, Karen; University of Toronto, Interdepartmental Division of Critical Care<br>Alhazzani, Waleed; Imam Abdulrahman Bin Faisal University, Critical Care and Internal Medicine Department, College of Medicine; Ministry of Defense Health Services, Research and Innovation Institute

	Vazquez-Grande, Gloria; University of Manitoba, Department of Critical Care<br>Geagea, Anna; North York General Hospital, Critical Care<br>Myburgh, John; University of New South Wales, Faculty of Medicine; The George Institute for Global Health, Critical Care Program; Saint George Hospital, Intensiv Care Unit<br>Muscedere, John; Queen’s University, Department of Critical Care Medicine<br>English, Shane; University of Ottawa, Department of Medicine; Ottawa Hospital Research Institute, Clinical Epidemiology Program<br>Binnie, Alexandra; William Osler Health System, Department of Critical Care Medicine<br>Tsang, Jennifer; McMaster University Department of Medicine; Niagara Health System, Department of Critical Care Medicine<br>Hunt, Miranda; Queen's University, Critical Care Medicine<br>Ostermann, Marlies; King's College London, Guy's & St Thomas' Hospital , Department of Critical Care<br>Al-Fares, Abdulrahman A; Al-Amiri Hospital, Department of Anesthesia, Critical Care, Pain Medicine and Kuwait Extracorporeal Life Support<br>Knowles, Serena; The George Institute for Global Health, Critical Care program<br>Ahmed, Aijaz; Stanford University, Department of Gastroenterology & Hepatology<br>Cook, Deborah; McMaster University, Departments of Medicine, Health Research Methods, Evidence and Impact; St. Joseph’s Healthcare Hamilton Research Institute<br>The REVISE Investigators, The Canadian Critical Care Trials Group; Sunnybrook and Women's College Health Sciences Centre
**Primary Subject Heading**:	Intensive care
Secondary Subject Heading:	Epidemiology, Infectious diseases, Respiratory medicine
Keywords:	COVID-19, Adult intensive & critical care < INTENSIVE & CRITICAL CARE, Research Design

I, the Submitting Author has the right to grant and does grant on behalf of all authors of the Work (as defined in the below author licence), an exclusive licence and/or a non-exclusive licence for contributions from authors who are: i) UK Crown employees; ii) where BMJ has agreed a CC-BY licence shall apply, and/or iii) in accordance with the terms applicable for US Federal Government officers or employees acting as part of their official duties; on a worldwide, perpetual, irrevocable, royalty-free basis to BMJ Publishing Group Ltd ("BMJ") its licensees and where the relevant Journal is co-owned by BMJ to the co-owners of the Journal, to publish the Work in this journal and any other BMJ products and to exploit all rights, as set out in our licence.

The Submitting Author accepts and understands that any supply made under these terms is made by BMJ to the Submitting Author unless you are acting as an employee on behalf of your employer or a postgraduate student of an affiliated institution which is paying any applicable article publishing charge ("APC") for Open Access articles. Where the Submitting Author wishes to make the Work available on an Open Access basis (and intends to pay the relevant APC), the terms of reuse of such Open Access shall be governed by a Creative Commons licence – details of these licences and which Creative Commons licence will apply to this Work are set out in our licence referred to above.

Other than as permitted in any relevant BMJ Author's Self Archiving Policies, I confirm this Work has not been accepted for publication elsewhere, is not being considered for publication elsewhere and does not duplicate material already published. I confirm all authors consent to publication of this Work and authorise the granting of this licence.

For peer review only

Proton Pump Inhibitors in Invasively Ventilated Patients with SARS-CoV-2: A Substudy of the Re-EValuating the Inhibition of Stress Erosions (REVISE) Trial

Brittany Dennis MBBS, PhD Brittany.dennis@ubc.ca
Department of Medicine, University of British Columbia, Canada

Diane Heels-Ansdell, MSc ansdell@mcmaster.ca
Department of Health Research Methods, Evidence, and Impact, McMaster University, Hamilton, Ontario, Canada

Quazi Ibrahim, MSc ibrahiq@mcmaster.ca
Department of Health Research Methods, Evidence, and Impact, McMaster University, Hamilton, Ontario, Canada

John Basmaji, MD, MSc jbasmaji2013@gmail.com
Department of Medicine, Division of Critical Care, Western University, London, Ontario, Canada

Lehana Thabane, PhD thabanl@mcmaster.ca
Department of Health Research Methods, Evidence, and Impact, McMaster University, Hamilton, Ontario, Canada, Biostatistics Unit, St. Joseph's Healthcare Hamilton, Hamilton, Ontario, Canada
St. Joseph's Healthcare Hamilton Research Institute, Hamilton, Ontario, Canada

Gordon Guyatt, MD, MSc guyatt@mcmaster.ca
Department of Health Research Methods, Evidence, and Impact, McMaster University, Hamilton, Ontario, Canada
Department of Medicine, McMaster University, Hamilton, Ontario, Canada

Lois Saunders lsaunde@mcmaster.ca
Research Institute, St. Joseph's Healthcare Hamilton, Ontario, Canada

Lori Hand, RRT handlori@hhsc.ca
Department of Health Research Methods, Evidence, and Impact, McMaster University, Hamilton, Ontario, Canada

Olivia Loy, BSc, MBChB olivia.loy@nhs.net
Department of Critical Care, The Royal Melbourne Hospital, ICU

Nicole Zytaruk, RN zytarn@mcmaster.ca
Department of Health Research Methods, Evidence, and Impact, McMaster University, Hamilton, Ontario, Canada
Research Institute, Division of Critical Care, St. Joseph's Healthcare Hamilton, Ontario, Canada

Miranda Hardie, MClinEpi mhardie@georgeinstitute.org.au
Critical Care Program, The George Institute for Global Health, Faculty of Medicine, University of New South Wales, Sydney, New South Wales, Australia

Adam M Deane, MD Adam.Deane@mh.org.au
Intensive Care Unit, University of Melbourne, Melbourne, Victoria, Australia
Department of Critical Care Medicine, University of Melbourne Parkville Campus, Victoria, Australia

John C Marshall, MD John.Marshall@unityhealth.to
Interdepartmental Division of Critical Care, University of Toronto, Toronto, Ontario, Canada

For peer review only

Yaseen Arabi, MD Arabi@mngha.med.sa
Intensive Care Department, Ministry of the National Guard-Health Affairs, Riyadh, Kingdom of Saudi Arabia
King Saud bin Abdulaziz University for Health Sciences, Riyadh, Kingdom of Saudi Arabia
King Abdullah International Medical Research Center, Riyadh, Kingdom of Saudi Arabia

François Lauzier, MD, MSc Francois.Lauzier@fmed.ulaval.ca
Departments of Anesthesiology, Medicine and Critical Care Medicine, Université Laval, Québec, Québec, Canada

Joanna C Dionne MD, MSc, PhD dionnejc@mcmaster.ca
Department of Medicine, McMaster University, Hamilton, Ontario, Canada
Departments of Health Research Methods, Evidence, and Impact, McMaster University, Hamilton, Ontario, Canada

Karen EA Burns, MD, MSc karen.burns@unityhealth.to
Interdepartmental Division of Critical Care, University of Toronto, Toronto, Ontario, Canada

Waleed Alhazzani, MD, MSc waleed.al-hazzani@medportal.ca
Critical Care and Internal Medicine Department, College of Medicine, Imam Abdulrahman Bin Faisal University, Dammam, Eastern Province, Saudi Arabia
Research and Innovation Institute, Ministry of Defense Health Services, Riyadh, Saudi Arabia

Gloria Vazquez-Grande, MD, PhD gloriavazquezgrande@gmail.com
Department of Critical Care, University of Manitoba, Winnipeg, Manitoba, Canada

Anna Geagea, MD, PhD anna.geagea@nygh.on.ca
Department of Critical Care, North York General Hospital, North York, Ontario, Canada

John Myburgh, MD, PhD jmyburgh@georgeinstitute.org.au
Faculty of Medicine, University of New South Wales, Sydney, New South Wales, Australia
Critical Care Program, The George Institute for Global Health, Sydney, New South Wales, Australia
Intensive Care Unit, St. George Hospital, Kogorah, New South Wales, Australia

John Muscedere, MD John.Muscedere@kingstonhsc.ca
Department of Critical Care Medicine, Queen's University, Kingston, Ontario, Canada

Shane English MD, MSc senglish@toh.ca
Department of Medicine, Division of Critical Care, University of Ottawa, Ottawa, Ontario Canada
Clinical Epidemiology Program, Ottawa Hospital Research Institute, Ottawa, Ontario, Canada

Alexandra Binnie MD, PhD allie@binnie.ca
Department of Critical Care Medicine, William Osler Health System, Brampton, Ontario, Canada

Jennifer LY Tsang MD, PhD Jennifer.Tsang@niagarahealth.on.ca
Department of Medicine, Division of Critical CareMcMaster University, Hamilton, Ontario, Canada
Department of Critical Care Medicine, Niagara Health System, St. Catharines, Ontario, Canada

Miranda Hunt, BA Miranda.hunt@kingstonhsc.ca
Department of Critical Care Medicine, Queen's University, Kingston, Ontario, Canada

Marlies Ostermann, MBBS Marlies.Ostermann@gstt.nhs.uk

For peer review only - http://bmjopen.bmj.com/site/about/guidelines.xhtml

Department of Critical Care, King's College London, Guy's & St Thomas' Hospital, London, UK

Abdulrahaman A Al-Fares, MD abdulrahman.alfares@gmail.com
Departments of Anesthesia, Critical Care, Pain Medicine and Kuwait Extracorporeal Life Support Program, Al-Amiri Hospital, Kuwait City, Al Asimah Governate, Kuwait

Serena Knowles, PhD sknowles@georgeinstitute.org.au
Critical Care Program, The George Institute for Global Health, Newton, New South Wales, Australia

Aijaz Ahmed, MD safia93@stanford.edu
Department of Gastroenterology and Hepatology, Stanford University, Palo Alto, California, USA

*Deborah J Cook, MD, MSc debcook@mcmaster.ca
Department of Medicine, McMaster University, Hamilton, Ontario, Canada
Department of Health Research Methods, Evidence, and Impact, McMaster University, Hamilton, Ontario, Canada
St. Joseph's Healthcare Hamilton Research Institute, Hamilton, Ontario, Canada

For the REVISE Investigators and the Canadian Critical Care Trials Group

*Correspondence: DJ Cook, Departments of Medicine & Health Research Methods, Evidence and Impact, McMaster University Health Sciences Center, Room 2C11, 1200 Main Street West, Hamilton, Ontario, Canada, L8N 3Z5; debcook@mcmaster.ca

For peer review only

Proton Pump Inhibitors in Invasively Ventilated Patients with SARS-CoV-2: A Substudy of the Re-EValuating the Inhibition of Stress Erosions (REVISE) Trial

ABSTRACT

Objectives: Observational studies suggest that acid suppression may worsen outcomes among patients infected with severe acute respiratory syndrome coronavirus-2 (SARS-CoV-2). The objectives of this embedded substudy of a randomized controlled trial evaluating pantoprazole in mechanically ventilated patients were to (1) describe the clinical characteristics of critically ill patients with SARS-CoV-2, (2) compare clinical outcomes with a propensity-matched non-infected cohort, and (3) assess whether pantoprazole's treatment effects differed by SARS-CoV-2 infection status.

Design: Pre-planned substudy of the REVISE trial including a propensity-matched analysis of infected and non-infected patients, comparing the effect of pantoprazole between patients with and without SARS-CoV-2.

Setting: 68 intensive care units (ICUs) in 8 countries.

Participants: From July 2019 to October 2023, 4,821 eligible participants were enrolled in REVISE whether or not they had SARS-CoV-2 infection.

Primary and Secondary Outcome Measures: Participants enrolled in REVISE with SARS-CoV-2 infection had additional data collection including biomarkers, venous thromboembolism, SARS-CoV-2 therapies, and tracheostomy timing. The primary outcomes were clinically important upper gastrointestinal bleeding and 90-day mortality. Secondary outcomes included ventilator-associated pneumonia, *C. difficile* infection, patient important upper GI bleeding, renal replacement therapy, ICU and hospital mortality, and duration of mechanical ventilation, ICU stay, and hospital stay.

Results: Of the eligible trial cohort, 11.9% (540/4550) had SARS-CoV-2; 532 patients had additional SARS-CoV-2-specific data collection. Of these 532 patients, 87.8% received Covid-19-directed treatments – (dexamethasone 75.2%), 11.7% developed pulmonary embolism and 9.2% developed deep-vein thrombosis. After propensity-matching, SARS-CoV-2 infection was not associated with clinically important upper gastrointestinal bleeding (adjusted Hazard Ratio [aHR] 0.78, 95% Confidence Interval [CI] 0.40–1.50), but was associated with significantly higher ICU, hospital, and 90-day mortality, as well as longer duration of ventilation, ICU and hospital length of stay. The effect of pantoprazole on clinically important upper GI bleeding and 90-day mortality was consistent regardless of SARS-CoV-2 status.

Conclusions: SARS-CoV-2 infection was associated with higher mortality and longer duration of mechanical ventilation, ICU and hospital stay, without an increased risk of clinically important upper gastrointestinal bleeding. Pantoprazole reduced clinically important upper gastrointestinal bleeding without adversely affecting other outcomes.

Trial Registration: REVISE Trial [NCT03374800], SARS-CoV-2 Cohort Study [NCT05715567]
Abstract Word Count: 338
Manuscript Word Count: 3470
Figure: 1; **Tables:** 4; **Supplemental Tables**: 4; **Supplemental Material**: 1
References: 22

Article Summary

Strengths and Limitations of this Study:

- This SARS-CoV-2 Cohort substudy was embedded in a large randomized trial, registered, and guided by a published protocol and statistical analysis plan with pre-specified hypotheses.
- This substudy offers the first randomized trial data on the effect of PPIs among patients with SARS-CoV-2 on a range of clinical outcomes.
- We lack information about SARS-CoV-2 variants and SARS-CoV-2 vaccine timing.

For peer review only - http://bmjopen.bmj.com/site/about/guidelines.xhtml

- Our definition of COVID-19 is limited to patients who received COVID-19 specific treatment; other infected patients may not have been symptomatic.
- The propensity analysis could not adjust for all possible factors predisposing to SARS-CoV-2 leading to possible unrecognized confounding.

For peer review only

INTRODUCTION

Early in the pandemic, proton pump inhibitors (PPIs) were hypothesized to influence the susceptibility to severe acute respiratory syndrome coronavirus-2 (SARS-CoV-2) and result in worse clinical outcomes among infected patients, although findings were inconsistent. Among older adults, early observational data suggested that recent exposure to PPIs was associated with a reduced risk of infection. [1] However, several retrospective studies subsequently raised concern that antacid use among SARS-CoV-2 infected patients may worsen clinical outcomes.[2-4] Across large claims-based and hospital-based retrospective cohorts, PPI use was linked to a higher risk of severe coronavirus disease of 2019 (COVID-19), ICU admission, mechanical ventilation, and death, although some associations were attenuated after adjustment for age and comorbidities. [2, 3] Consistent with these signals, a pooled analysis of retrospective studies also suggested an increased risk of mortality associated with COVID-19 among PPI users,[4] as well as a more severe course of infection. [5]

To reduce the risk of upper gastrointestinal (GI) bleeding, clinicians often administer PPIs prophylactically in critically ill patients.[6] During the COVID-19 pandemic, international guidance for the management of these patients supported use of PPIs or histamine-2-receptor antagonists (H2Ras) in high-risk patients,[7] yet sepsis guidance did not directly address stress ulcer prophylaxis,[8] and in the early pandemic period, randomized trials had not yet evaluated whether PPIs affected clinical outcomes for ICU patients with COVID-19. Given unclear guidelines for COVID-19 and practices during the pandemic that could have increased bleeding risk (e.g., anticoagulation [9], high-dose corticosteroids [10]) and an apparent increased risk of ventilator-associated pneumonia [11], we conducted a planned substudy within the Re-EValuating the Inhibition of Stress Erosions (REVISE) trial to evaluate the association between PPI exposure and clinically important outcomes in ICU patients tested positive for SARS-CoV-2.[12]

This prospective substudy was pre-planned, nested within an international randomized blinded trial in critically ill patients, investigating the effect of pantoprazole 40 mg intravenously compared to placebo on the primary *efficacy* outcome of clinically important upper GI bleeding and the primary *safety* outcome of 90-day mortality. REVISE found that pantoprazole significantly lowered the risk of clinically important and patient important upper GI bleeding in invasively ventilated patients, but had no effect on other outcomes including 90-day mortality, ventilator-associated pneumonia, *Clostridioides difficile (C. difficile*) infection, renal replacement therapy, or mortality at ICU and hospital discharge. [13] While testing positive for SARS-CoV-2 during hospital admission did not influence treatment effect for the primary outcome,[13] the effect of pantoprazole in patients with COVID-19 requiring treatment has not been determined on ventilator-associated pneumonia, *C. difficile* infection, and other outcomes in patients.

As SARS-CoV-2 is now endemic and PPIs are commonly prescribed in the ICU, understanding the relationship between PPI exposure and clinical outcomes in this population warrants investigation. Accordingly, within this multi-center SARS-CoV-2 substudy of the REVISE trial [NCT05715567],[12] we (1) describe the clinical characteristics and hospital admission trajectory of critically ill patients tested positive for SARS-CoV-2, (2) compare clinical outcomes with a propensity-matched non-infected cohort, and (3) assess whether pantoprazole's treatment effects differed by COVID-19 status.

METHODS

Nested Cohort Study Design

REVISE was registered [NCT03374800], and the trial design [14], statistical analysis plan, [15] and pandemic adaptations[16] were previously published, as designed by the Canadian Critical Care Trials Group and the Australian and New Zealand Intensive Care Society Clinical Trials Group. Overall, 4,821

For peer review only - http://bmjopen.bmj.com/site/about/guidelines.xhtml

patients undergoing invasive mechanical ventilation were enrolled in 68 ICUs in Canada, Australia, Saudi Arabia, United Kingdom, United States, Kuwait, Pakistan and Brazil from July 2019 to October 2023.

This multi-center cohort design was registered [NCT05715567], funded, and guided by a protocol and statistical analysis plan.[12] (**Supplemental Material 1**) We included patients in this substudy if they were enrolled in REVISE on or after March 11, 2020. Notwithstanding the operational constraints imposed by the SARS-CoV-2 pandemic on ICU-based research,[17] REVISE enrollment experienced only temporary recruitment suspensions at participating centers, and did not impact eligibility criteria: patients both with and without SARS-CoV-2 were included.[16] The substudy protocol outlined the objectives, *a priori* hypotheses, and outcomes[12], summarized below.

Trial Intervention

Study drug (40 mg intravenous pantoprazole or matching placebo) was prescribed in the ICU until discontinuation of invasive mechanical ventilation, the occurrence of a prespecified indication or contraindication to PPI use, or death, up to 90 days.

Outcomes

The clinical outcomes for REVISE and this substudy were outlined in the trial registration [NCT03374800], trial protocol[14], trial statistical analysis plan,[15] trial report,[13] and protocol for this substudy.[12]

Characterizing Patients with COVID-19

Briefly, patients were eligible for this substudy according to the inclusion and exclusion criteria of REVISE.[13] Inclusion in the SARS-CoV-2 cohort was based on SARS-CoV-2 viral nucleic acid test positivity by polymerase chain reaction (PCR) from a nasopharyngeal swab, oropharyngeal swab, sputum, endotracheal aspirate, or bronchoalveolar lavage sample, as clinically determined rather than per protocol.[12] Patients were eligible if they tested positive for SARS-CoV-2 in hospital (whether contracted preceding the index ICU admission, pre-hospital, or in hospital).

Patients were eligible for this substudy if they tested positive. These patients contributed to objective 1. However, only patients receiving COVID-19 specific treatment were considered symptomatic for SARS-CoV-2 and were included in analyses pertaining to objectives 2 and 3.

No additional tests or treatments were protocolized, and care was at the discretion of the treating team. Additional data collection on SARS-CoV-2 positive patients included biomarkers, venous thromboembolic events, tracheostomy occurrence and timing, and COVID-19 therapies. In the ICU, detailed daily data collection occurred. On the ward thereafter, *C. difficile* infection, vital status and hospital length of stay were captured. Mortality was documented at 90 days.

Covariates for Matching

The patients' age was abstracted from the medical record in years. Biological sex was abstracted from the medical record; gender was not considered. Pre-hospital acid suppression and use of corticosteroids in the week pre-randomization was documented by research personnel from the patient's medical record, clarified with a hospital or community pharmacist or substitute decision-maker if necessary. The ICU admission category of medical vs surgical/trauma was classified by research personnel based on the APACHE III admission diagnostic categories. Days from the start of pandemic to enrollment was calculated from March 11, 2020.

Analysis

As per the substudy statistical analysis plan [12], to characterize the cohort of patients tested positive for

For peer review only - http://bmjopen.bmj.com/site/about/guidelines.xhtml

SARS-CoV-2 within the REVISE trial (*Objective 1*), we used descriptive statistics. Continuous variables were analyzed and presented using mean ± standard deviation (SD), or median (interquartile range [IQR]) for non-normally distributed or skewed data. Categorical variables are presented using frequencies and percentages.

We examined associations between SARS-CoV-2 status and clinical outcomes after adjusting for age, sex, pre-hospital acid suppression, ICU admission category (medical or surgical/trauma), corticosteroids one week pre-randomization, and days from start of pandemic to enrollment (*Objective 2*). Propensity matching only considered patients enrolled after or on March 11th, 2020.

We implemented two methods to address confounding: 1) multivariable regressions and 2) an optimal propensity score matching (PSM) of 2:1 ratio of non-COVID-19 patients with COVID-19 patients. The score was the probability of COVID-19 predicted by the confounders. This method identified the set of matched triplets that minimized the overall difference in propensity scores across all possible matching combinations. We used optimal propensity matching at a 2:1 ratio, matching two non-COVID patients to each COVID patient. This method identified the set of matched triplets that minimized the overall difference in propensity scores across all possible matching combinations. While multivariable regression was not originally proposed in the protocol, we added it to evaluate the adjusted association between COVID-19 and clinical outcomes while preserving sample size lost from matching. Using Cox-proportional hazards regressions, we studied the association between COVID-19 and clinically important upper GI bleeds, incident *C difficile*, new renal replacement therapy, patient important upper GI bleeding, ventilator-associated pneumonia, and mortality in ICU, hospital, and within 90 days. Ventilator-associated pneumonia was classified in this substudy using the Clinical Pulmonary Infection Score as in the main trial report. The proportional hazard (PH) assumption was checked using interaction of an explanatory variable with log (time). In case the PH assumption was violated for an explanatory variable, an interaction of that variable with log time was considered in the model and relative effects, hazard ratios, were reported at different timepoints. ICU and hospital length of stay were highly skewed to the right. Thus, natural logarithms of these two variables were regressed on COVID-19 status and *a priori* confounders and adjusted (exponentiated) relative effect of COVID-19 on length of stays were reported. For propensity-matching analysis, patients within a matched triplet were correlated. To account for correlation in the time-to-event analyses, a robust sandwich type variance estimator for clusters was used. For the length-of-stay outcomes, to account for within matched triplet (cluster) correlations, random intercepts models were used in multivariable linear regressions. All analyses were adjusted for randomized allocation; mortality was also adjusted for APACHE II score. A sensitivity analysis was originally planned to evaluate only pre-hospital PPI use (not H2RAs); however, this was not performed, as less than 1% of patients used H2RAs.[13]

Responding to unexpected findings of a lower risk of ventilator-associated pneumonia among patients with COVID-19 compared to those without COVID-19, exploratory post hoc analyses further evaluated the association between SARS-CoV-2 status and ventilator-associated pneumonia. Both the propensity-matched analysis and the multivariable regression models for the outcome of ventilator-associated pneumonia were revised, adjusting for baseline pneumonia at ICU admission, alongside prespecified confounders and randomized assignment (pantoprazole vs placebo) included in the original models. A second post hoc analysis to evaluate the association between COVID-19 status and ventilator-associated pneumonia was repeated after excluding patients with baseline pneumonia. This involved using both propensity-matching and multivariable regression methods. Propensity scores were re-estimated in the reduced cohort and prespecified models were refit. Additional exploratory post hoc analyses evaluated the association between COVID-19 status and clinically important upper GI bleeds, incident *C. difficile*, new renal replacement therapy, patient important upper GI bleeding, and ventilator-associated pneumonia, accounting for the competing risk of death.

For peer review only - http://bmjopen.bmj.com/site/about/guidelines.xhtml

Finally, we examined whether pantoprazole had a differential effect on clinically important upper GI bleeding or 90-day mortality among patients with COVID-19 compared with those without (*Objective 3*), using a prespecified subgroup analysis of REVISE.[15] Subgroup effects were also evaluated for secondary trial outcomes of ventilator-associated pneumonia, *C. difficile* infection, patient important upper GI bleeding, renal replacement therapy, ICU and hospital mortality, and duration of mechanical ventilation, ICU stay, and hospital stay. For time-to-event outcomes, subgroup analyses were conducted using Cox proportional hazards models that included COVID-19 status, randomized treatment, and an interaction term between SARS-CoV-2 status and treatment. Analyses followed the intention-to-treat principle, with separate models fitted for each outcome. Models were adjusted for pre-hospital acid suppression use (either PPI or H2RA; exposure to either was a stratification variable), and mortality models were additionally adjusted for APACHE II score. As per the REVISE trial statistical analysis plan, if any subgroup effects were identified, using the interaction term when p-values were <0.10, we planned to assess the credibility of subgroup effects, interpreted using ICEMAN.[18] Results were reported as hazard ratios [HR]with 95% confidence intervals [CI]. For duration outcomes (mechanical ventilation, ICU stay, and hospital stay), subgroup analyses were performed using linear regression models on the natural logarithms of the outcomes including COVID-19 status, randomized treatment, their interaction term, and pre-hospital acid suppression.

Given negligible missing data (<2%), we did not perform imputation.[13] As specified in the protocol, we did not adjust for multiple comparison adjustments. Analyses were conducted using SAS version 9.4 [19] and STATA 15.1 (College Station, Texas).[19]

Patient and Public Involvement

Members of the public who were substitute decision makers for critically ill patients, and sometimes critically ill patients themselves, were involved in the informed consent process to participate in the parent REVISE trial. One outcome reported herein (patient important bleeding) was developed by ICU survivors and family members of survivors or decedents [13].

Ethics

Relevant ethics committees and regulatory authorities in participating hospitals approved REVISE. The Hamilton Integrated Research Ethics Board under Clinical Trials Ontario (CTO Study ID 1360), the main board of record, also approved this substudy.

RESULTS

Of 4,821 enrolled patients in the trial, 264 participants were not eligible from this substudy due to pre-pandemic randomization. An additional 7 patients were excluded due to declined or revoked consent, or whose SARS-CoV-2 status remained unknown, resulting in a final cohort of 4,550 patients. Within the remaining study cohort, 11.9% (540/4,010) were tested positive for SARS-CoV-2 at any time during hospitalization. The SARS-CoV-2 -specific case report form was not completed in 8 patients in one center who were therefore excluded in analyses for Objective 1 examining treatments and other characteristics. There were an additional 65 patients for whom the SARS-CoV-2 form indicated no SARS-CoV-2-directed treatment. Because COVID-19 status could not be definitively classified for these 65 patients, they were included in Objective 1 analyses but excluded from comparative analyses (Objective 2: propensity-matched analyses, Objective 3: REVISE SARS-CoV-2 subgroup analyses). Accordingly, the final analytic sample for Objectives 2 and 3 comprised 4,477 patients, including 4,010 non–SARS-CoV-2 patients and 467 COVID-19 patients (Figure 1).

Characteristics of Participants with SARS-CoV-2

Among the 532 patients with complete information on their SARS-CoV-2 status (i.e., treatments

For peer review only - http://bmjopen.bmj.com/site/about/guidelines.xhtml

received) enrolled in the REVISE trial, 268 were randomized to pantoprazole, and 264 to placebo. Table 1 shows the characteristics of the overall SARS-CoV-2 Cohort. Briefly, almost half of infected participants were unvaccinated *(n*=226, 42.5%). Treatments for COVID-19 were administered to most participants (*n*=467, 87.8%); dexamethasone was most commonly prescribed (*n*=400, 75.2%).

Complications of SARS-CoV-2 were common (e.g., pulmonary embolism and deep vein thrombosis occurred in 62 (11.7%), and 49 (9.2%) participants, respectively). Biomarkers after enrolment captured in this cohort demonstrated high rates of inflammation, with median d-dimer levels at 2855.5 mg/L (IQR: 1180-5000); C-reactive protein of 156.1 mg/dL (IQR: 78.2-243.5); and ferritin of 883 ng/mL (IQR 403-1870).

Comparison of Participants with and without COVID-19

Clinical characteristics and ICU admission data for the COVID-19 cohort are summarized in Table 2. COVID-19 patients were primarily admitted to ICU for medical reasons (COVID-19: 440, 94.2% vs. Non-SARS-CoV-2: 2,819, 70.3%), and most received corticosteroids in the week preceding randomization (COVID-19: 362, 77.5% vs. Non-SARS-CoV-2: 1,206, 30.1%). Evaluation of *a priori* selected variables used in the propensity score model demonstrated that baseline variables were significantly associated with COVID-19 status (Supplementary Table S1). After propensity-matching, patients with COVID-19 and without SARS-CoV-2 were well balanced across baseline demographic and clinical characteristics, as demonstrated in Supplementary Table S2.

No differences in the propensity-matched analysis were observed between patients with COVID-19 and without SARS-CoV-2 for clinically important upper GI bleeding (adjusted HR [aHR]: 0.78, 95% CI 0.40, 1.50, p=0.45). However, COVID-19 patients had 73% higher hazard of mortality at 30 days compared to non-SARS-CoV-2 propensity-matched patients (aHR: 1.73; 95% CI: 1.06, 2.40). At 60 days, this increased to 150% higher hazard (aHR: 2.50; 95% CI: 1.13, 3.87), and further to 210% at 90-days (aHR: 3.10; 95% CI: 1.10, 5.10). Similarly, we observed higher mortality among COVID-19 patients, both during hospitalization and throughout the 90-day follow-up (Table 3).

Other outcomes including incident C. *difficile* infection, patient important upper GI bleeding, and new renal replacement therapy were not associated with COVID-19 status (Table 3). However, incident infection with VAP appeared to be lower amongst participants who had COVID-19 (aHR: 0.50; 95% CI: 0.37, 0.69).

Post hoc exploratory adjustment for baseline pneumonia diminished the initial apparent association between COVID-19 status and ventilator-associated pneumonia (Supplementary Table S3) (aHR: 1.08; 95% CI: 0.78, 1.50). Further, no association between COVID-19 status and ventilator-associated pneumonia was observed after excluding patients with pneumonia at baseline from the propensity-matched analysis (Supplementary Table S3). Competing risk analyses yielded similar results to those presented in Table 3 and Supplemental Table S3.

COVID-19 was associated with a 45% longer ICU stay (adjusted ratio of length of stay 1.45; 95% CI: 1.33, 1.58, p<0.001), a 30% longer hospital stay (adjusted ratio 1.30; 95% CI: 1.18, 1.43, p<0.001) and a 69% increase in the number of days of mechanical ventilation (adjusted ratio of duration 1.69; 95% CI: 1.53, 1.87, p<0.001)

Overall, results were consistent between multivariable regression models and the primary propensity-matched analyses (Table 3, Supplemental Tables S3 and S4).

Substudy Subgroup Analyses

For peer review only - http://bmjopen.bmj.com/site/about/guidelines.xhtml

COVID-19 status in subgroup analyses did not suggest an effect modification of pantoprazole on the primary efficacy outcome of clinically important upper GI bleeding (Non-SARS-CoV-2 HR: 0.30: 95% CI: 0.18, 0.49; COVID-19 HR: 0.27: 95% CI: 0.08, 0.95, interaction p-value: 0.868) or the primary safety outcome of 90-day mortality (Non-SARS-CoV-2 HR: 0.97: 95% CI: 0.86, 1.09; COVID-19 HR: 0.94: 95% CI: 0.69, 1.27, interaction p-value: 0.855). Similarly, no differences were observed for other outcomes of patient important upper GI bleeding, renal replacement therapy, VAP, *C. difficile* infection in hospital, ICU and hospital mortality, duration of mechanical ventilation, ICU stay, or hospital length of stay. Table 4 summarizes the results of all subgroup analyses.

DISCUSSION

Among 4,550 patients in this stress ulcer prophylaxis trial enrolled after the beginning of the pandemic, 11% had were tested positive for SARS-CoV-2, most of whom received directed therapies and were deemed to have COVID-19. These patients frequently developed thrombotic complications, and biomarkers demonstrated an intense inflammatory burden. After propensity-matching with patients not tested positive for SARS-CoV-2, COVID-19 infection was not associated with clinically important upper GI bleeding, but was associated with higher ICU, hospital, and 90-day mortality and longer duration of mechanical ventilation, and ICU and hospital length of stay. The effect of pantoprazole reducing clinically important upper GI bleeding with no effect on 90-day mortality was consistent irrespective of COVID-19 status. Overall, we found no evidence that SARS-CoV-2 modified the treatment effect of pantoprazole on any outcome.

This study evaluated several outcomes in patients with COVID-19 that have not been well studied, including *C. difficile* infection, patient important upper GI bleeding, and new renal replacement therapy. Our initial analyses suggested that COVID-19 was associated with a lower risk of ventilator-associated pneumonia, inconsistent with previous literature.[11, 20-22] In fact, most studies characterizing COVID-19 patients found a high rate of secondary pneumonia with an incidence ranging between 18-39/1,000 mechanically ventilated days.[20-22] A high risk of ventilator-associated pneumonia was also observed in a systematic review by Ippolito et. al, which found a pooled rate of 45.4% (95% C.I. 37.8-53.2%; 2611/5593 patients).[11] The initial counterintuitive finding of COVID-19 being associated with a lower risk of ventilator-associated pneumonia prompted post hoc exploratory analyses, which suggested that the apparent association was driven by incomplete adjustment for baseline pneumonia – likely more common among patients with COVID-19. Another possible explanation may be difficulty ascertaining a new episode of ventilator-associated pneumonia in patients with COVID-19, which could have led to missed diagnoses and confounding that attenuated once baseline pneumonia was accounted for (or excluded), resulting in no independent association.

Limitations of this study include the lack of information about SARS-CoV-2 variants and SARS-CoV-2 vaccine timing. We also cannot exclude the possibility of selection bias in some centers given a lack of information about patients were eligible for REVISE but who were not enrolled. Our definition of COVID-19 is limited to patients who received COVID-19 specific treatment; others may have been symptomatic. On the other hand, we assumed that those who received specific treatments for COVID-19 actually had COVID-19, whereas a patient could have received dexamethasone for upper respiratory tract edema after dental abscess drainage and tested positive for SARS-CoV-2, without the virus necessarily causing symptoms. The REVISE trial was not designed *a priori* to study COVID-19, as the pandemic was declared after the trial had launched. Furthermore, no pre-specified sample size calculation was possible to discern an effect modification based on SARS-CoV-2 status. The confidence intervals around the hazard ratios for some results are relatively wide, suggesting uncertainty about the power to detect an interaction effect if one existed. Finally, our propensity analysis could not adjust for all possible factors predisposing to SARS-CoV-2 leading to possible unrecognized confounding.

For peer review only - http://bmjopen.bmj.com/site/about/guidelines.xhtml

Strengths of this study include a large and prospectively collected trial dataset. This SARS-CoV-2 Cohort substudy was registered [NCT05715567], hypotheses were pre-specified for each objective and analysis was guided by a statistical analysis plan.[12] The point estimates and confidence intervals for the propensity-matching and multivariable regression analyses are similar, increasing our confidence in the inferences about these results. This substudy offers the first randomized trial data on the effect of PPIs among patients with SARS-CoV-2 on a range of clinical outcomes.

CONCLUSION

In this preplanned substudy, COVID-19 infection was associated with higher mortality and longer ICU and hospital stays without an increased risk of clinically important upper GI bleeding in critically ill mechanically ventilated patients compared to patients who were not tested positive for SARS-CoV-2. Pantoprazole demonstrated similar effects on bleeding and safety outcomes regardless of COVID-19 status. Despite observational reports suggesting potential harm from acid suppression in patients with COVID-19, pantoprazole reduced clinically important and patient important upper GI bleeding without affecting other outcomes, offering consistent benefit regardless of COVID-19 status, supporting its consideration for stress ulcer prophylaxis.

For peer review only

For peer review only - http://bmjopen.bmj.com/site/about/guidelines.xhtml

Table 1: Characterization of REVISE Patients with tested positive for SARS-CoV-2 During Hospital Stay

Tested positive SARS-CoV-2 positive any time during hospital stay AND part of the SARS-CoV-2 substudy*	Pantoprazole n=268	Placebo n=264	Total n=532
Vaccination status pre-ICU			
Not vaccinated	111 (41.4)	115 (43.6)	226 (42.5)
Unknown	52 (19.4)	60 (22.7)	112 (21.1)
1 dose	21 (7.8)	12 (4.5)	33 (6.2)
2 doses	46 (17.2)	26 (9.8)	72 (13.5)
3 doses	38 (14.2)	51 (19.3)	89 (16.7)
Computed tomography positive for PE, number (%)			
Yes	28 (10.4)	34 (12.9)	62 (11.7)
No	94 (35.1)	115 (43.6)	209 (39.3)
Test not done	146 (54.5)	115 (43.6)	261 (49.1)
Ultrasound positive for one or more DVT, number (%)			
Yes	24 (9.0)	25 (9.5)	49 (9.2)
No	61 (22.8)	55 (20.8)	116 (21.8)
Test not done	183 (68.3)	184 (69.7)	367 (69.0)
Positive for bowel ischemia, number (%)			
Yes	3 (1.1)	5 (1.9)	8 (1.5)
No	53 (19.8)	58 (22.0)	111 (20.9)
Test not done	212 (79.1)	201 (76.1)	413 (77.6)
D Dimer level, mg/L (highest), median (IQR)	2460 (1020-5000) n=142	3485 (1250-6240) n=154	2855.5 (1180-5000) n=296
C-reactive protein level, mg/dL (highest), median (IQR)	148.0 (72.3-243.0) n=185	163.5 (89.8-252.0) n=199	156.1 (78.2-243.5) n=384
Ferritin level, ng/mL (highest), median (IQR)	905 (440-1717) n=113	866.5 (370-1935) n=124	883 (403-1870) n=237
Possible SARS-CoV-2-related treatments			
ACE2 Renin-angiotensin Inhibitor	30 (11.2)	20 (7.6)	50 (9.4)
Azithromycin	114 (42.5)	117 (44.3)	231 (43.4)
Baricitinib (Janus-associated kinase (JAK) inhibitor)	23 (8.6)	18 (6.8)	41 (7.7)
Casirivimab	2 (0.7)	4 (1.5)	6 (1.1)
Casirivimab and imdevimab (*casirivimab* / imdevimab monoclonal antibody)	2 (0.7)	0 (0.0)	2 (0.4)
Convalescent plasma	0 (0.0)	0 (0.0)	0 (0.0)
Dexamethasone	201 (75.0)	199 (75.4)	400 (75.2)
Other high dose steroid	32 (11.9)	46 (17.4)	78 (14.7)
Nirmatrelvir/ritonavir *(Paxlovid)*	3 (1.1)	2 (0.8)	5 (0.9)
Molnupiravir	2 (0.7)	1 (0.4)	3 (0.6)
Oseltamivir	3 (1.1)	6 (2.3)	9 (1.7)
Remdesivir	57 (21.3)	60 (22.7)	117 (22.0)
Ruxolitinib	0 (0.0)	1 (0.4)	1 (0.2)
Sarilumab	5 (1.9)	3 (1.1)	8 (1.5)
Sotrovimab (monoclonal antibody)	0 (0.0)	1 (0.4)	1 (0.2)

For peer review only - http://bmjopen.bmj.com/site/about/guidelines.xhtml

Statin	51 (19.0)	46 (17.4)	97 (18.2)
Tixagevimab/cilgavimab	0 (0.0)	1 (0.4)	1 (0.2)
Tocilizumab	77 (28.7)	76 (28.8)	153 (28.8)
Intravenous vitamin C	20 (7.5)	21 (8.0)	41 (7.7)
Other†	16 (6.0)	17 (6.4)	33 (6.2)
Extra-corporeal membrane oxygenation	2 (0.7)	7 (2.7)	9 (1.7)
Any of the above	237 (88.4)	230 (87.1)	467 (87.8)
None of the above	31 (11.6)	34 (12.9)	65 (12.2)
Tracheostomy, number (%)	37 (13.8)	47 (17.8)	84 (15.8)
Days from randomization to tracheostomy, in those with a tracheostomy	n=37	n=47	n=84
Tracheostomy prior to day of randomization	4 (10.8)	3 (6.4)	7 (8.3)
0-6 days	2 (5.4)	2 (4.3)	4 (4.8)
7-13 days	6 (16.2)	10 (21.3)	16 (19.0)
14-20 days	10 (27.0)	15 (31.9)	25 (29.8)
21-27 days	11 (29.7)	7 (14.9)	18 (21.4)
28-34 days	2 (5.4)	6 (12.8)	8 (9.5)
35 or more days	2 (5.4)	4 (8.5)	6 (7.1)
Days from randomization to tracheostomy in those with no tracheostomy prior to day of randomization	n=33	n=44	n=77
mean (SD)	19.0 (8.9)	21.1 (16.2)	20.2 (13.5)
median (IQR)	19 (14-22)	17.5 (12.5-24)	18 (13-23)
total range	0-47	0-105	0-105

Legend for Table 1: In this table, we describe the 532 patients with confirmed SARS-CoV-2 at any time during hospital stay who are part of the comparative analyses for this substudy. However, there are 8 SARS-CoV-2 positive patients from 1 center which did not participate in the SARS-CoV-2 substudy.

† Other treatments include: hydroxychloroquine, ivermectin, colchicine, ASA, vitamin D, acyclovir, valacyclovir, zinc, rivaroxaban.

SARS-CoV-2= severe acute respiratory syndrome coronavirus-2
ICU=intensive care unit
PE=pulmonary embolism
DVT=deep vein thrombosis
ACE2=angiotensin converting enzyme-2
IQR=interquartile range
SD=standard deviation

For peer review only

Table 2: Clinical Characteristics of Patients by COVID-19 Status

	Patients not tested positive for SARS-CoV-2 n=4010	COVID-19 Patients n=467	Total n=4477
Age in years, mean (SD)	57.9 (16.5)	61.4 (14.0)	58.2 (16.3)
APACHE II score, mean (SD)	21.8 (8.3)	20.9 (8.0)	21.7 (8.3)
Sex, number (%)			
Female	1439 (35.9)	187 (40.0)	1626 (36.3)
Male	2571 (64.1)	280 (60.0)	2851 (63.7)
Patient status, number (%)			
Medical	2819 (70.3)	440 (94.2)	3259 (72.8)
Surgical	564 (14.1)	15 (3.2)	579 (12.9)
Trauma	627 (15.6)	12 (2.6)	639 (14.3)
Admitting diagnostic category, number (%)			
Cardiovascular	441 (11.0)	23 (4.9)	464 (10.4)
Respiratory	1037 (25.9)	365 (78.2)	1402 (31.3)
Gastrointestinal	191 (4.8)	6 (1.3)	197 (4.4)
Neurologic	979 (24.4)	31 (6.6)	1010 (22.6)
Sepsis	338 (8.4)	14 (3.0)	352 (7.9)
Trauma	627 (15.6)	12 (2.6)	639 (14.3)
Metabolic	169 (4.2)	8 (1.7)	177 (4.0)
Renal	59 (1.5)	2 (0.4)	61 (1.4)
Other Medical	61 (1.5)	4 (0.9)	65 (1.5)
Other Surgical	108 (2.7)	2 (0.4)	110 (2.5)
Pre-hospital acid suppression, number (%)			
Yes	920 (22.9)	104 (22.3)	1024 (22.9)
No	3090 (77.1)	363 (77.7)	3453 (77.1)
Corticosteroids in the week pre-randomization, number (%)	1206 (30.1)	362 (77.5)	1568 (35.0)
**Study Day 1**, number (%)			
Invasive mechanical ventilation	4010 (100.0)	467 (100.0)	4477 (100.0)
Inotrope or vasopressor infusion	2825 (70.4)	343 (73.4)	3168 (70.8)
Renal replacement therapy	272 (6.8)	17 (3.6)	289 (6.5)

Legend for Table 2: This table summarizes the clinical characteristics and information on the critical admission for patients from the REVISE trial with data presented disaggregated by SARS-CoV-2 status.

SD=standard deviation
APACHE II score= Acute Physiology and Chronic Health Evaluation II Score
SARS-CoV-2= severe acute respiratory syndrome coronavirus-2

For peer review only

For peer review only - http://bmjopen.bmj.com/site/about/guidelines.xhtml

Table 3. Associations between SARS-CoV-2 status and Clinical Outcomes

Outcomes	Confounders adjustment using propensity-matching (2:1) (n=1401) Adjusted Hazard Ratio (95% CI) SARS-CoV-2 (n=467) vs. non-SARS-CoV-2 (n=934)	p-value	Confounders adjustment using multivariable regressions[1] (n=4477) Adjusted Hazard Ratio (95% CI) SARS-CoV-2 (n=467) vs. non-SARS-CoV-2 (n=4010)	p-value
**Time to event outcome: no violation of Proportional Hazards assumption by SARS-CoV-2 status**				
Clinically important upper GI bleed[1]	0.78 (0.40, 1.50)	0.45	0.74 (0.41, 1.34)	0.32
Incident *C. difficile*[1]	0.67 (0.21, 2.10)	0.49	0.75 (0.25, 2.23)	0.61
New renal replacement therapy[1]	1.24 (0.85, 1.81)	0.26	1.25 (0.88, 1.77)	0.21
Patient important upper GI bleed[1]	0.77 (0.43, 1.38)	0.38	0.76 (0.45, 1.31)	0.32
Ventilator-associated pneumonia[1]	0.50 (0.37, 0.69)	< 0.001	0.47 (0.35, 0.63)	< 0.001
**Time to event outcome: violation of Proportional Hazards assumption by SARS-CoV-2 status**				
ICU mortality[2] at				
30 days	1.73 (1.06, 2.40)	< 0.001	1.47 (1.02, 1.91)	< 0.001
60 days	2.50 (1.13, 3.87)	< 0.001	2.07 (1.17, 2.98)	< 0.001
90 days	3.10 (1.10, 5.10)	0.002	2.54 (1.21, 3.87)	< 0.001
Hospital mortality[2] at				
30 days	1.62 (1.17, 2.07)	< 0.001	1.49 (1.14, 1.84)	< 0.001
60 days	2.18 (1.33, 3.03)	< 0.001	2.03 (1.35, 2.70)	< 0.001
90 days	2.59 (1.39, 3.79)	< 0.001	2.43 (1.46, 3.39)	< 0.001
Mortality within 90 days[2]				
30 days	1.91 (1.45, 2.37)	< 0.001	1.78 (1.41, 2.16)	< 0.001
60 days	2.65 (1.76, 3.55)	< 0.001	2.56 (1.79, 3.32)	< 0.001
90 days	3.22 (1.93, 4.50)	< 0.001	3.16 (2.01, 4.30)	< 0.001
**Linear regression models studying associations between length of stay outcomes and SARS-CoV-2 status**				
	Adjusted Ratio of Length of Stay/Duration (95% CI)[¥]	p-value	Adjusted Ratio of Length of Stay/Duration (95% CI)[¥]	p-value
Outcomes				
**Duration of ICU stay in days**[¶, 1]				
Exposure of interest: SARS-CoV-2 vs. non-SARS-CoV-2	1.45 (1.33, 1.58)	< 0.001	1.45 (1.35, 1.57)	< 0.001

For peer review only - http://bmjopen.bmj.com/site/about/guidelines.xhtml

**Duration of hospital stay in days**¶, [1]				
Exposure of interest: SARS-CoV-2 vs. non-SARS-CoV-2	1.30 (1.18, 1.43)	< 0.001	1.35 (1.24, 1.48)	< 0.001
**Duration of mechanical ventilation in days**¶, [1]				
Exposure of interest: SARS-CoV-2 vs. non-SARS-CoV-2	1.69 (1.53, 1.87)	< 0.001	1.66 (1.52, 1.81)	< 0.001

Legend for Table 3: In this table we show the effect of SARS-CoV-2 status on clinical outcomes among mechanically ventilated patients admitted to the ICU. Cox proportional hazard regression models and linear regression models were used to study associations between SARS-CoV-2 status and clinical outcomes using two methods of confounders adjustment: 1) Propensity-matching (2:1) and 2) multivariable regression.

[1]Adjusted for patients' age, sex, pre-hospital acid suppression, ICU admission category (medical or surgical/trauma), corticosteroids in the week pre randomization, days from start of pandemic to enrollment, and randomization group (Pantoprazole vs placebo)
[2]Adjusted for variables listed in 1, and APACHE II score quartiles
¶Natural logarithm of length of stay was the dependent variable in the linear regressions
¥Exponentiation of the adjusted regression coefficient and its' confidence intervals are presented

SARS-CoV-2= severe acute respiratory syndrome coronavirus-2
GI=gastrointestinal
C. difficile=Clostridioides difficile
ICU=intensive care unit
CI=confidence interval

For peer review only

Table 4: Efficacy of Pantoprazole in Patients with SARS-CoV-2 in REVISE: Subgroup Analyses

	Pantoprazole n (%)	Placebo n (%)	Hazard Ratio (95% CI)	P-value for the interaction term
**Primary Efficacy Outcome (Clinically Important Upper GI Bleeding)**				
Non-SARS-CoV-2	20/1983 (1.0)	67/1982 (3.4)	**0.30 (0.18, 0.49)**	0.868
SARS-CoV-2	3/235 (1.3)	12/226 (5.3)	**0.27 (0.08, 0.95)**	
**Primary Safety Outcome (90-day Mortality)**				
Non-SARS-CoV-2	567/1987 (28.5)	583/1986 (29.4)	0.97 (0.86, 1.09)†	0.855†
SARS-CoV-2	82/235 (34.9)	88/224 (39.3)	0.94 (0.69, 1.27)†	
**Incident Ventilator-associated pneumonia (Clinical Pulmonary Infection Score ≥6) in ICU**				
Non-SARS-CoV-2	487/1990 (24.5)	495/1986 (24.9)	1.00 (0.88, 1.13)	0.881
SARS-CoV-2	26/235 (11.1)	26/226 (11.5)	1.04 (0.61, 1.80)	
**Incident *C. difficile* infection in hospital**				
Non-SARS-CoV-2	21/1983 (1.1)	16/1982 (0.8)	1.33 (0.69, 2.55)	0.985
SARS-CoV-2	4/235 (1.7)	0/226 (0.0)	-	
**New renal replacement therapy in ICU**				
Non-SARS-CoV-2	110/1983 (5.5)	110/1985 (5.5)	1.01 (0.77, 1.31)	0.308
SARS-CoV-2	26/235 (11.1)	20/226 (8.8)	1.41 (0.78, 2.52)	
**ICU mortality**				
Non-SARS-CoV-2	392/1998 (19.6)	401/1997 (20.1)	1.00 (0.87, 1.15)	0.944
SARS-CoV-2	60/236 (25.4)	67/227 (29.5)	1.01 (0.71, 1.44)	
**Hospital mortality**				
Non-SARS-CoV-2	514/1996 (25.8)	536/1988 (27.0)	0.99 (0.87, 1.11)	0.663
SARS-CoV-2	71/236 (30.1)	80/227 (35.2)	0.92 (0.66, 1.26)	
**Incident Patient important Upper GI Bleeding**				
Non-SARS-CoV-2	28/1983 (1.4)	81/1982 (4.1)	0.34 (0.22, 0.53)	0.763
SARS-CoV-2	5/235 (2.1)	13/226 (5.8)	0.41 (0.15, 1.15)	
	**Pantoprazole median (IQR)**	**Placebo median (IQR)**	**Ratio of Duration (95% CI)**	**P-value for the interaction term**
**Duration of mechanical ventilation in days**				
Non-SARS-CoV-2	6 (3-11)	6 (3-11)	0.97 (0.92, 1.03)	0.170
SARS-CoV-2	9 (5-16)	10 (6-19)	0.87 (0.74, 1.01)	
**Duration of ICU stay in days**				

For peer review only - http://bmjopen.bmj.com/site/about/guidelines.xhtml

Non-SARS-CoV-2	9 (6-15)	9 (6-16)	0.98 (0.93, 1.02)	0.129
SARS-CoV-2	13 (7-21)	14 (9-23)	0.87 (0.76, 1.00)	
**Duration of hospital stay in days**				
Non-SARS-CoV-2	20 (11-35)	21 (11-37)	0.96 (0.91, 1.02)	0.690
SARS-CoV-2	22 (15-40)	25 (14-43)	0.93 (0.79, 1.09)	

Legend for Table 4: In this table we show the effect of pantoprazole versus placebo on clinical outcomes among patients with and without SARS-CoV-2.
† For 90-day mortality, the SARS-CoV-2 subgroup variable violates the assumption of proportional hazards. Therefore, the first Cox proportional hazards model includes only "Non-SARS-CoV-2" patients, and a separate Cox proportional hazards model including only "SARS-CoV-2" patients. The two hazard ratios are compared by performing a z-test on the log(hazard ratio). We did not apply criteria to assess subgroup credibility because all p-values were above 0.10, according to our statistical analysis plan.

SARS-CoV-2= severe acute respiratory syndrome coronavirus-2
GI=gastrointestinal
C. difficile =*Clostridioides difficile*
ICU=intensive care unit

For peer review only

Acknowledgements: The trial was designed by the REVISE Steering Committee including National and International Management Committees, the REVISE Investigators and Research Coordinators, the Canadian Critical Care Trials Group and the Australian and New Zealand Intensive Care Society Clinical Trials Group. We thank the patients and families participating in this trial, and all the research personnel and bedside clinicians who supported this work. We are grateful to others at the Methods Center at McMaster University (Lisa Buckingham, France Clarke, Mary Copland, Karlo Matic, Ashley Sawyer, Alyson Takaoka) and The George Institute (Fatima Butt, Anna Cheng, Conrad Nangla, Fiona Osborne, Tina Schneider, Isabella Schoeler, Anna Tippet) for their expertise.

Competing interests: All authors are involved in the REVISE Trial in some capacity except Aijaz Ahmed. Otherwise, all authors declare that they have no competing interests.

Funding Statement: REVISE was funded by peer-reviewed grants [Canadian Institutes of Health Research (201610PJT-378226-PJT-CEBA-18373 and 202207CL3-492565-CTP-CEBA-19215)], and the Hamilton Academy of Health Sciences Organization (HAH-22-009). National Health and Medical Research Council of Australia grant [GNT1124675] funded enrolment in Australia. REVISE was approved by the National Institute for Health Research (NIHR) in the UK as a Portfolio Study [CPMS ID 45782], eligible for support from the NIHR Clinical Research Network. [https://www.nihr.ac.uk/researchers/collaborations-services-and-support-for-your-research/run-your-study/crn-portfolio.htm]. This SARS-CoV-2 Substudy was funded by a peer-review grant from Physicians Services Incorporated of Ontario (R22-33), and non-grant funds from St. Joseph's Healthcare Hamilton and McMaster University. The This trial received no support from the commercial or private sector. The funders/sponsors have no role in the conception, design, conduct, oversight, analysis, interpretation, write-up, review or approval of the manuscript, or decision to submit the manuscript for publication.

Role of the Funders: The funders and sponsors have no role in the conception, design, conduct, oversight, analysis, interpretation, write-up, review or approval of the manuscript, or decision to submit the manuscript for publication.

For peer review only

Ethics and Regulatory Approval: The main REVISE trial was approved in participating hospitals by all relevant ethics committees and regulatory authorities prior to starting enrolment. The Hamilton Integrated Research Ethics Board (HiREB) under Clinical Trials Ontario (CTO) Study ID 1360 is the main REB of record, which also approved this substudy. Ethics review for the additional SARS-CoV-2-specific data collection was undertaken as needed to comply with local regulations in each participating center.

Authors' contributions:

Concept and design: Y Arabi, A Binnie, D Cook, A Deane, B Dennis, J Dionne, S English, G Guyatt, M Hardie, D Heels-Ansdell, S Knowles, F Lauzier, J Marshall, J Muscedere, J Myburgh, M Ostermann, Saunders, L Thabane, J Tsang, N Zytaruk

Acquisition, analysis, or interpretation of data: J Basmaji, D Cook, B Dennis, L Hand, M Hardie, D Heels-Ansdell, S Knowles, O Loy, L Saunders, L Thabane, N Zytaruk

Drafting of the manuscript: D Cook, B Dennis, D Heels-Ansdell, Q Ibrahim

Statistical analysis: J Basmaji, G Guyatt, D Heels-Ansdell, Q Ibrahim, L Thabane

Obtained funding for this substudy: A Ahmed, A Binnie, D Cook, B Dennis, J Dionne, G Guyatt, D Heels-Ansdell, L Thabane, J Tsang

Administrative, technical, or material support: J Basmaji, D Cook, A Deane, B Dennis, G Guyatt, M Hardie, D Heels-Ansdell, Q Ibrahim, S Knowles, F Lauzier, O Loy, L Saunders, A Poole, L Thabane, N Zytaruk

Data Integrity: J Basmaji, D Cook, B Dennis, M Hardie, D Heels-Ansdell, Q Ibrahim, S Knowles, N Zytaruk

Approved final manuscript: All authors

Data Sharing Statement: The REVISE dataset will be used for secondary observational studies such as this one, addressing additional hypothesis-driven questions. The study protocol and statistical analysis plan for this substudy are published. Access by REVISE investigators to the REVISE dataset will follow a submitted rationale, analysis plan and approval by the Management Committee. Requests for access to the data for this substudy and the parent REVISE trial for investigator-initiated studies can be sent by external academic investigators. These will be considered following a submitted rationale, methods and analysis plan, then approval by the REVISE Management Committee and research ethics boards as relevant. Requirements will be stipulated in a pre-specified data sharing agreement. If approved, only de-identified trial data, data dictionaries and standard operating procedures will be provided to achieve aims of the proposal. Data will be transferred via a secure web portal. Data are available until 2035.

For peer review only

REFERENCES

1. Xiang Y, Wong KC, So HC: **Exploring Drugs and Vaccines Associated with Altered Risks and Severity of COVID-19: A UK Biobank Cohort Study of All ATC Level-4 Drug Categories Reveals Repositioning Opportunities**. *Pharmaceutics* 2021, **13**(9).
2. Lee SW, Ha EK, Yeniova AÖ, Moon SY, Kim SY, Koh HY, Yang JM, Jeong SJ, Moon SJ, Cho JY *et al*: **Severe clinical outcomes of COVID-19 associated with proton pump inhibitors: a nationwide cohort study with propensity score matching**. *Gut* 2021, **70**(1):76-84.
3. Aby ES, Tappata M, Rodin H, Debes JD: **Assessment of proton-pump inhibitor use, race, socioeconomic status, and mortality in individuals with COVID-19**. *European Journal of Gastroenterology & Hepatology* 2022, **34**(2):239.
4. Hariyanto TI, Prasetya IB, Kurniawan A: **Proton pump inhibitor use is associated with increased risk of severity and mortality from coronavirus disease 2019 (COVID-19) infection**. *Dig Liver Dis* 2020, **52**(12):1410-1412.
5. Pranata R, Huang I, Lawrensia S, Henrina J, Lim MA, Lukito AA, Kuswardhani RAT, Wibawa IDN: **Proton pump inhibitor on susceptibility to COVID-19 and its severity: a systematic review and meta-analysis**. *Pharmacol Rep* 2021, **73**(6):1642-1649.
6. Krag M, Perner A, Wetterslev J, Wise MP, Borthwick M, Bendel S, McArthur C, Cook D, Nielsen N, Pelosi P *et al*: **Prevalence and outcome of gastrointestinal bleeding and use of acid suppressants in acutely ill adult intensive care patients**. *Intensive Care Med* 2015, **41**(5):833-845.
7. World Health O: **Clinical management of severe acute respiratory infection (SARI) when COVID-19 disease is suspected: interim guidance, 13 March 2020**. In. Geneva: World Health Organization; 2020.
8. Alhazzani W, Evans L, Alshamsi F, Møller MH, Ostermann M, Prescott HC, Arabi YM, Loeb M, Ng Gong M, Fan E *et al*: **Surviving Sepsis Campaign Guidelines on the Management of Adults With Coronavirus Disease 2019 (COVID-19) in the ICU: First Update**. *Crit Care Med* 2021, **49**(3):e219-e234.
9. The REMAP-CAP, ACTIV-4a, and ATTACC Investigators. **Therapeutic Anticoagulation with Heparin in Critically Ill Patients with Covid-19**. *New England Journal of Medicine* 2021, **385**(9):777-789.
10. **Dexamethasone in Hospitalized Patients with Covid-19**. *New England Journal of Medicine* 2020, **384**(8):693-704.
11. Ippolito M, Misseri G, Catalisano G, Marino C, Ingoglia G, Alessi M, Consiglio E, Gregoretti C, Giarratano A, Cortegiani A: **Ventilator-Associated Pneumonia in Patients with COVID-19: A Systematic Review and Meta-Analysis**. *Antibiotics (Basel)* 2021, **10**(5).
12. Dennis BB, Thabane L, Heels-Ansdell D, Dionne JC, Binnie A, Tsang J, Guyatt G, Ahmed A, Lauzier F, Deane A *et al*: **Proton pump inhibitors in critically ill mechanically ventilated patients with COVID-19: protocol for a substudy of the Re-EValuating the Inhibition of Stress Erosions (REVISE) Trial**. *Trials* 2023, **24**(1):561.
13. Cook D, Deane A, Lauzier F, Zytaruk N, Guyatt G, Saunders L, Hardie M, Heels-Ansdell D, Alhazzani W, Marshall J *et al*: **Stress Ulcer Prophylaxis during Invasive Mechanical Ventilation**. *N Engl J Med* 2024, **391**(1):9-20.
14. **Re-EValuating the Inhibition of Stress Erosions (REVISE) Trial (REVISE)**. In. ClinicalTrials.Gov; 2018.
15. Heels-Ansdell D, Billot L, Thabane L, Alhazzani W, Deane A, Guyatt G, Finfer S, Lauzier F, Myburgh J, Young P *et al*: **REVISE: re-evaluating the inhibition of stress erosions in the ICU-statistical analysis plan for a randomized trial**. *Trials* 2023, **24**(1):796.

For peer review only - http://bmjopen.bmj.com/site/about/guidelines.xhtml

16. Dennis B, Deane A, Lauzier F, Zytaruk N, Hardie M, Hammond N, Finfer S, Arabi Y, Marshall J, Saunders L *et al*: **Protocol implementation during the COVID-19 pandemic: experiences from a randomized trial of stress ulcer prophylaxis**. *BMC Med Res Methodol* 2024, **24**(1):109.
17. Cook DJ, Kho ME, Duan EH, Alhazzani W, Takaoka A, Clarke FJ, Zytaruk N, Vanstone M: **Principles Guiding Nonpandemic Critical Care Research During a Pandemic**. *Crit Care Med* 2020, **48**(10):1403-1410.
18. Schandelmaier S, Briel M, Varadhan R, Schmid CH, Devasenapathy N, Hayward RA, Gagnier J, Borenstein M, van der Heijden GJMG, Dahabreh IJ *et al*: **Development of the Instrument to assess the Credibility of Effect Modification Analyses (ICEMAN) in randomized controlled trials and meta-analyses**. *Canadian Medical Association Journal* 2020, **192**(32):E901-E906.
19. **SAS 9.4**. In. Edited by Inc SI. Cary, NC.
20. Maes M, Higginson E, Pereira-Dias J, Curran MD, Parmar S, Khokhar F, Cuchet-Lourenço D, Lux J, Sharma-Hajela S, Ravenhill B *et al*: **Ventilator-associated pneumonia in critically ill patients with COVID-19**. *Crit Care* 2021, **25**(1):25.
21. Giacobbe DR, Battaglini D, Enrile EM, Dentone C, Vena A, Robba C, Ball L, Bartoletti M, Coloretti I, Di Bella S *et al*: **Incidence and Prognosis of Ventilator-Associated Pneumonia in Critically Ill Patients with COVID-19: A Multicenter Study**. *J Clin Med* 2021, **10**(4).
22. Blonz G, Kouatchet A, Chudeau N, Pontis E, Lorber J, Lemeur A, Planche L, Lascarrou JB, Colin G: **Epidemiology and microbiology of ventilator-associated pneumonia in COVID-19 patients: a multicenter retrospective study in 188 patients in an un-inundated French region**. *Crit Care* 2021, **25**(1):72.

For peer review only

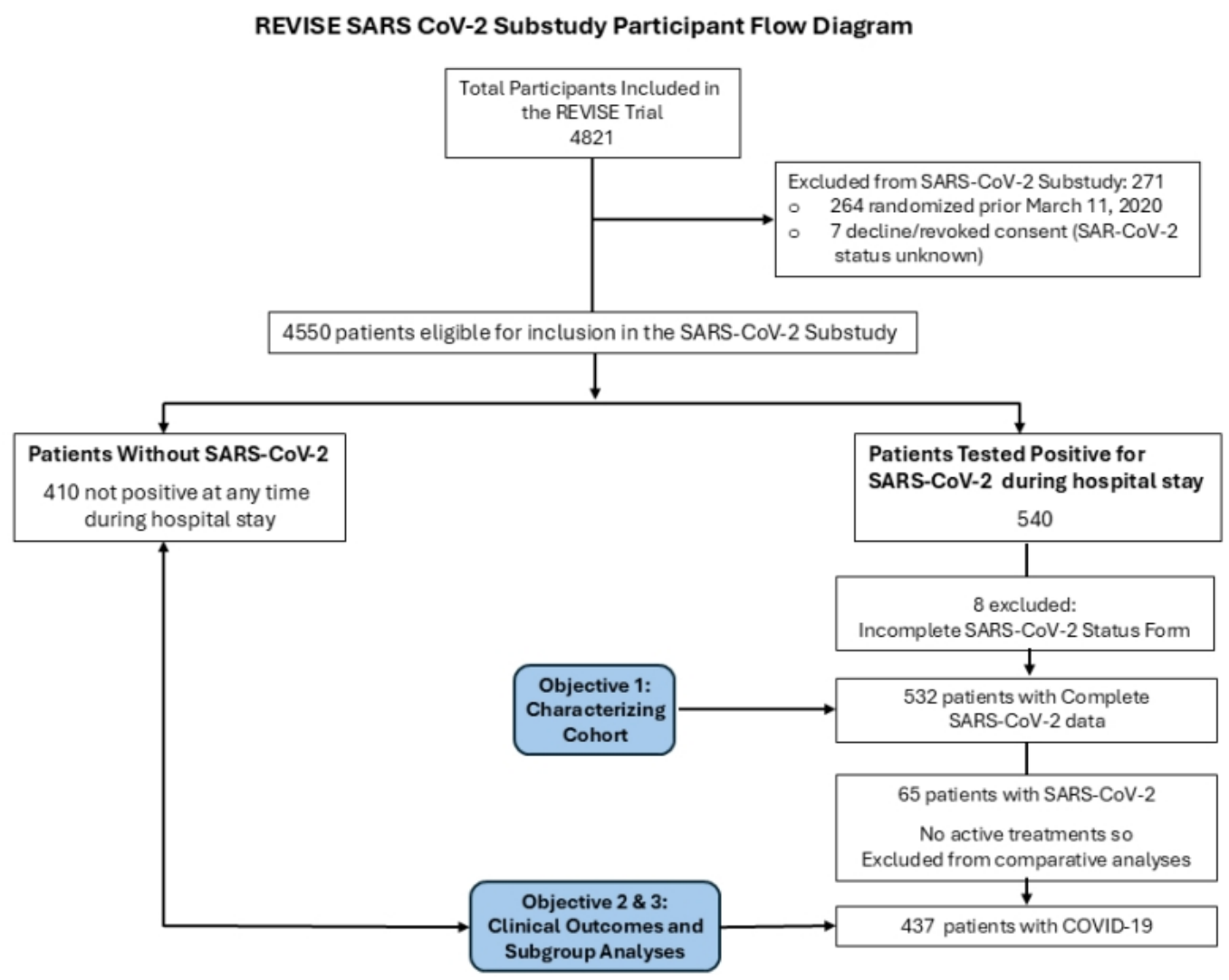

This figure summarizes participant inclusion into the SARS-CoV-2 Substudy, highlighting differences in sample size for each of the analyses addressing the 3 study objectives.

338x190mm (96 x 96 DPI)

Supplementary Tables:

Supplementary Table S1: Propensity Model Regressing SARS-CoV-2 Status on confounders using multivariable logistic regression

	Adjusted Odds Ratio (95% CI)
**Confounders**	
Age, 10-year increment	1.10 (1.03, 1.19)
Female	1.06 (0.86, 1.30)
Prehospital acid suppression	0.69 (0.54, 0.89)
ICU admission category: medical vs. surgical/trauma	4.34 (2.90, 6.51)
Corticosteroids in the week pre randomization	6.71 (5.33, 8.47)
Days from start of pandemic to enrollment, 1-year increment	0.73 (0.65, 0.81)

Legend Table S1: This stable shows a summary of results of a multivariable logistic regression model (*n*=4477) regressing SARS-CoV-2 status on the *a priori*-selected confounders being used in propensity-matching. SARS-COV-yes (n=467) or SARS-CoV-2-no (n=4010). The model demonstrates significant association between *a priori* selected pre-randomization confounder variables (i.e., age, pre-hospital acid suppression) and SARS-CoV-2 status.

ICU=intensive care unit

Supplementary Table S2: Patient Characteristics and Propensity Scores According to SARS-CoV-2 Status in the Propensity-Matched Cohort

	SARS-CoV-2 Patients	Non- SARS-CoV-2 Patients	Propensity-matched Non- SARS-CoV-2 Patients (2:1)
Number of patients, n (%)	467	4010	934
Age (years), mean ± SD	61 ± 14	58 ± 17	62 ± 13
Female, n (%)	187 (40.0)	1439 (35.9)	365 (39.1)
Prehospital acid suppression, n (%)	104 (22.3)	920 (22.9)	217 (23.2)
ICU admission category, n (%)			
Medical	440 (94.2)	2819 (70.3)	878 (94.0)
Surgical/trauma	27 (5.8)	1191 (29.7)	56 (6.0)
Corticosteroids in the week pre randomization, n (%)	362 (77.5)	1206 (30.1)	726 (77.7)
Days from start of pandemic to enrollment, mean ± SD	669 ± 280	787 ± 344	692 ± 339
Propensity Score			
mean ± SD	0.221 ± 0.110	0.091 ± 0.100	0.219 ± 0.110
median (IQR)	0.257 (0.145-0.305)	0.048 (0.024-0.112)	0.245 (0.146-0.295)
minimum	0.006	0.004	0.006
maximum	0.411	0.439	0.411

Legend Supplementary Table S2: This table shows the baseline characteristics of patients with SARS-CoV-2 and propensity-matched patients without SARS-CoV-2. This table also shows the propensity scores according to SARS-CoV-2 Status in the propensity-matched cohort.
SARS-CoV-2=severe acute respiratory syndrome coronavirus-2
SD=standard deviation
ICU=intensive care unit
IQR=interquartile range

For peer review only

Supplementary Table S3. Association between SARS-CoV-2 Status and Ventilator-associated Pneumonia

	Confounders adjustment using propensity matching (2:1) (n=1401)		Confounders adjustment using multivariable regressions (n=4477)	
Exploratory Model 1	Adjusted Hazard Ratio (95% CI) SARS-CoV-2 (n=467) vs. non- SARS-CoV-2 (n=934)	p-value	Adjusted Hazard Ratio (95% CI) SARS-CoV-2 (n=467) vs. non- SARS-CoV-2 (n=4010)	p-value
Ventilator-associated Pneumonia[1]	1.08 (0.78, 1.50)	0.63	1.00 (0.75, 1.34)	0.98
Exploratory Model 2	Adjusted Hazard Ratio (95% CI) SARS-CoV-2 (n=157) vs. non- SARS-CoV-2 (n=314)	p-value	Adjusted Hazard Ratio (95% CI) SARS-CoV-2 (n=157) vs. non- SARS-CoV-2 (n=3431)	p-value
Ventilator-associated Pneumonia[2]	0.98 (0.72, 1.35)	0.92	1.09 (0.82, 1.46)	0.55

Legend Supplementary Table S3: This table shows results from 2 exploratory analyses evaluating the association between SARS-CoV-2 status and ventilator-associated pneumonia, using different adjustment methods to account for pneumonia diagnoses on admission to the ICU. The 2 methods of confounder adjustment were: 1) Propensity-matching (2:1) and 2) Cox proportional hazards multivariable regression.

[1]Multivariable regression: adjusted for patients' age, sex, pre-hospital acid suppression, ICU admission category (medical or surgical/trauma), corticosteroids in the week pre randomization, days from start of pandemic to enrollment, randomization group (Pantoprazole vs placebo), and baseline pneumonia. Original Propensity-matched data: adjusted for randomization group (Pantoprazole vs placebo), and baseline pneumonia

[2]Patients with pneumonia at baseline were removed and original propensity scores were re-estimated on the reduced data. Exploratory models 1 were refitted in the reduced data.

SARS-CoV-2=severe acute respiratory syndrome coronavirus-2

For peer review only

Supplementary Table S4. Associations between SARS-CoV-2 Status and Clinical Outcomes, accounting for the competing risk of death

	Confounders adjustment using propensity matching (2:1) (n=1401)		Confounders adjustment using multivariable regressions (n=4477)	
	Adj SHR (95% CI) SARS-CoV-2 (n=467) vs. non- SARS-CoV-2 (n=934)	p-value	Adj SHR (95% CI) SARS-CoV-2 (n=467) vs. non- SARS-CoV-2 (n=4010)	p-value
Clinically important upper GI bleed[1]	0.86 (0.45, 1.64)	0.65	0.82 (0.46, 1.48)	0.52
Incident *C. difficile*[1]	0.69 (0.22, 2.17)	0.53	0.79 (0.28, 2.27)	0.67
New renal replacement therapy[1]	1.30 (0.90, 1.90)	0.17	1.33 (0.95, 1.86)	0.09
Patient important upper GI bleed[1]	0.85 (0.48, 1.51)	0.58	0.85 (0.50, 1.43)	0.54
Ventilator-associated pneumonia[1]	0.54 (0.40, 0.74)	< 0.001	0.51 (0.38, 0.68)	< 0.001
Ventilator-associated pneumonia[2]	1.21 (0.87, 1.67)	0.26	1.11 (0.84, 1.46)	0.48

Legend Supplementary Table S4: This table shows results from exploratory analyses evaluating the association between SARS-CoV-2 status and clinical outcomes, accounting for the competing risk of death using Fine and Gray subdistribution hazard regression models.

[1]Adjusted for patients' age, sex, pre-hospital acid suppression, ICU admission category (medical or surgical/trauma), corticosteroids in the week pre randomization, days from start of pandemic to enrollment, and randomization group (Pantoprazole vs placebo)

[2]Adjusted for variables listed in 1 plus baseline pneumonia

For peer review only

Dennis *et al. Trials (2023) 24:561*
https://doi.org/10.1186/s13063-023-07589-2

Trials

STUDY PROTOCOL Open Access

Proton pump inhibitors in critically ill mechanically ventilated patients with COVID-19: protocol for a substudy of the Re-EValuating the Inhibition of Stress Erosions (REVISE) Trial

Brittany B. Dennis[1], Lehana Thabane[2,3,4], Diane Heels-Ansdell[2], Joanna C. Dionne[5], Alexandra Binnie[6], Jennifer Tsang[1,6], Gordon Guyatt[5], Aijaz Ahmed[7], François Lauzier[8], Adam Deane[9], Yaseen Arabi[10,11,12], John Marshall[13], Nicole Zytaruk[2,4], Lois Saunders[4], Simon Finfer[14], John Myburgh[14,15], John Muscedere[16], Shane English[17,18], Marlies Ostermann[19], Miranda Hardie[14], Serena Knowles[14], Deborah Cook[4,5*] and For the REVISE Investigators the Canadian Critical Care Trials Group

Abstract

Background Critically ill patients commonly receive proton pump inhibitors (PPIs) to prevent gastrointestinal (GI) bleeding from stress-induced ulceration. Despite widespread use in the intensive care unit (ICU), observational data suggest that PPIs may be associated with adverse outcomes in patients with COVID-19 infection. This pre-planned study is nested within a large randomized trial evaluating pantoprazole versus placebo in invasively ventilated patients. The 3 objectives are as follows: (1) to describe the characteristics of patients with COVID-19 in terms of demographics, biomarkers, venous thromboembolism, tracheostomy incidence and timing, and other clinical outcomes; (2) to evaluate the impact of COVID-19 infection on clinically important GI bleeding, 90-day mortality, and other outcomes compared to a propensity-matched non-infected cohort; and (3) to explore whether pantoprazole has a differential treatment effect on clinically important GI bleeding, 90-day mortality, and other outcomes in patients with and without COVID-19 infection.

Methods The ongoing trial Re-EValuating the Inhibition of Stress Erosions (REVISE) compares pantoprazole 40 mg IV to placebo on the primary efficacy outcome of clinically important GI bleeding and the primary safety outcome of 90-day mortality. The protocol described in this report is for a substudy focused on patients with COVID-19 infection that was not in the original pre-pandemic trial protocol. We developed a one-page case report form to characterize these patients including data related to biomarkers, venous thromboembolism, COVID-19 therapies, tracheostomy incidence and timing, duration of mechanical ventilation, and ICU and hospital stay. Our analysis will describe the trajectory of patients with COVID-19 infection, a propensity-matched analysis of infected and non-infected patients, and an extended subgroup analysis comparing the effect of PPI among patients with and without COVID-19 infection.

*Correspondence:
Deborah Cook
debcook@mcmaster.ca
Full list of author information is available at the end of the article

© The Author(s) 2023. **Open Access** This article is licensed under a Creative Commons Attribution 4.0 International License, which permits use, sharing, adaptation, distribution and reproduction in any medium or format, as long as you give appropriate credit to the original author(s) and the source, provide a link to the Creative Commons licence, and indicate if changes were made. The images or other third party material in this article are included in the article's Creative Commons licence, unless indicated otherwise in a credit line to the material. If material is not included in the article's Creative Commons licence and your intended use is not permitted by statutory regulation or exceeds the permitted use, you will need to obtain permission directly from the copyright holder. To view a copy of this licence, visit http://creativecommons.org/licenses/by/4.0/. The Creative Commons Public Domain Dedication waiver (http://creativecommons.org/publicdomain/zero/1.0/) applies to the data made available in this article, unless otherwise stated in a credit line to the data.

For peer review only - http://bmjopen.bmj.com/site/about/guidelines.xhtml

Discussion Prophylactic acid suppression in invasively ventilated critically ill patients with COVID-19 infection has unknown consequences. The results of these investigations will inform practice, guidelines, and future research.

Trial registration REVISE Trial [NCT03374800 December 15, 2017], COVID-19 Cohort Study [NCT05715567 February 8, 2023].

Keywords Randomization, Critically ill, COVID-19 pandemic, Hospital transfers, Stress ulceration, Gastrointestinal bleeding, Ventilator-associated pneumonia

Introduction

For patients with COVID-19 receiving invasive mechanical ventilation, standard critical care therapies, including prophylactic acid suppression, have unclear consequences. Concerns emerged early in the pandemic that some common medications may predispose to COVID-19 infection or worsen outcomes among infected patients. For example, observational studies suggested that angiotensin-converting enzyme (ACE) inhibitors were harmful as they were associated with increased mortality [1, 2]. However, this finding was not supported in larger, more robust observational studies [3, 4] or randomized trials [5].

The possible impact of proton pump inhibitors (PPIs) on COVID-19 infection susceptibility and outcomes has also drawn attention. In one observational study from the UK Biobank cohort, PPIs were associated with a 23% reduction in susceptibility to COVID-19 infection (odds ratio [OR] at 1 year = 0.77, 95% confidence interval [CI]: 0.71–0.83), with the greatest protective effects observed with recent use of PPIs (within 6 months) and in people at least 70 years of age [6]. However, these findings of benefit were in contrast with other studies suggesting associated harm. In a retrospective study of Korean national insurance claims including 132,316 patients, short-term use of PPIs was associated with a 79% increased risk of a composite outcome of admission to the ICU, use of mechanical ventilation, and death [7]. These findings were limited by low event rates, reliance on registry data to ascertain PPI exposure, and the use of a composite outcome measure due to insufficient events. In another retrospective case review of 2164 patients with COVID-19 admitted to a US county hospital, PPI use was associated with a 2.8-fold increased risk for death (OR) [95% CI, 1.4–5.5], although findings were not statistically significant following adjustment for age, renal, cardiovascular, and pulmonary comorbidities [8]. Furthermore, a pooled analysis of six retrospective studies of 5884 COVID-19 patients also suggested that PPI use may be associated with an increased risk of death from COVID-19 (risk ratio 1.7, 95% CI 1.02–2.9, $I^2 = 66\%$) [9]. However, all of these study designs generate limited inferences for practice or research purposes.

PPIs are common medications among hospitalized patients, particularly in the intensive care unit (ICU). Whether used pre-hospital or not, critically ill mechanically ventilated patients are usually prescribed PPIs to prevent gastrointestinal (GI) bleeding from stress-induced ulceration [10]. The potential adverse effects of PPI use include ventilator-associated pneumonia (VAP) and *Clostridioides difficile* (*C. difficile*) infection—nosocomial infections that are together more common than GI bleeding and are associated with substantially greater morbidity, mortality, and costs [11]. Herein, we describe a protocol to examine the association between COVID-19 infection and outcomes and PPI use among invasively mechanically ventilated patients nested within an ongoing clinical trial testing pantoprazole versus placebo [12]. This ongoing trial is relevant for patients with COVID-19 infection, given their increased risk of VAP [13], treatment with high-dose corticosteroids [14], and varying anticoagulation intensities [15].

The three objectives of this nested study are as follows: (1) to describe the characteristics of patients with COVID-19 in terms of demographics, biomarkers, COVID-19 treatment, rates of venous thromboembolism, tracheostomy incidence and timing, and other outcomes (e.g., duration of mechanical ventilation, ICU and hospital stay, and mortality); (2) to evaluate the impact of COVID-19 on clinically important GI bleeding, 90-day mortality, infectious, and other outcomes compared to a propensity-matched non-COVID-19 cohort; and (3) to explore whether pantoprazole has a differential treatment effect on clinically important GI bleeding and 90-day mortality, in addition to VAP and *C. difficile* infection and other outcomes, in patients with and without COVID-19.

Methods

Project design for overall REVISE Trial

The Re-EValuating the Inhibition of Stress Erosions (REVISE) is an international randomized stratified, concealed, blinded, parallel-group trial in mechanically ventilated patients, investigating the effect of the PPI pantoprazole 40 mg IV compared to placebo, on the primary *efficacy* outcome of clinically important GI bleeding, the primary *safety* outcome of 90-day mortality, rates of VAP and *C. difficile* infection, patient-important

For peer review only - http://bmjopen.bmj.com/site/about/guidelines.xhtml

bleeding, use of renal replacement therapy, and hospital mortality [12]. Led by the Canadian Critical Care Trials Group and the Australian & New Zealand Intensive Care Society Clinical Trials Group, the REVISE Methods Centers are located at McMaster University in Hamilton for international centers and The George Institute in Sydney for Australia. Research Coordinators screen patients from Monday to Friday, using an *a priori* or a consent-to-continue model as locally approved, holding consent encounters with patients if able, or substitute decision-makers in-person or by telephone as needed. Randomization is conducted by research pharmacists to ensure double blinding. Methods for the REVISE trial have been described elsewhere [NCT03374800] [12].

Design for nested COVID cohort study

This study uses a multicenter cohort design embedded within a randomized trial [NCT05715567]. This is the first version (version 1.0; date: June 13, 2023) of the substudy protocol. Figure 1 highlights how it is integrated into the stress ulcer prophylaxis research program. Patients with COVID-19 have been eligible for REVISE since March 11, 2020. Despite the difficulties of continuing non-COVID research in ICUs during the pandemic, recruitment to REVISE was paused for the shortest possible period in each participating center throughout the pandemic. The objectives, a priori hypotheses and outcomes for this COVID-19 study are outlined in Table 1. Although the protocol described in this work would be characterized as a study within a trial, we adhered to the Standard Protocol Items for Clinical Trials (SPIRIT) reporting guidelines where appropriate [16]. Accordingly, the recommended SPIRIT diagram was not included in this work, this will be published as part of the full REVISE randomized trial protocol.

Setting

Patients are being enrolled in 63 ICUs worldwide (39 Canada, 19 Australia, 1 Saudi Arabia, 1 the UK, 1 the US, 1 Kuwait, and 1 Pakistan).

Population

Patients are eligible for this COVID-19 substudy according to the inclusion and exclusion criteria of the REVISE Trial, as outlined in Table 2. Randomization is not stratified by COVID-19 disease status. The inclusion in the COVID-19 cohort will be based on COVID-19 viral nucleic acid test positivity by polymerase chain reaction (PCR) from a nasopharyngeal swab, oropharyngeal swab, sputum, endotracheal aspirate, or bronchoalveolar lavage sample, as clinically determined rather than per protocol. The COVID-19 classification in this study will be made via PCR testing, due to the heightened risk for misclassification using other forms of testing, which will not

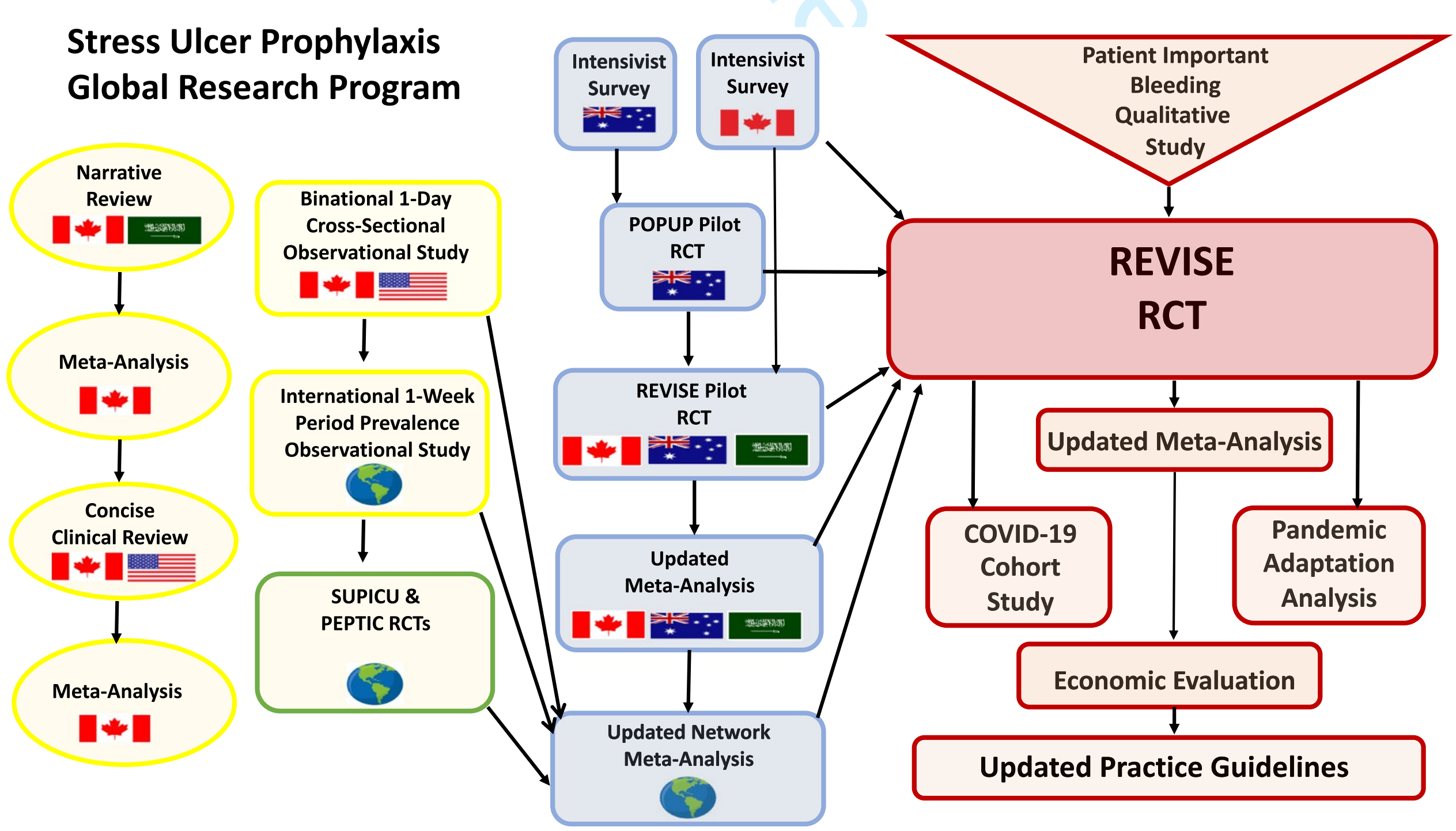

Fig. 1 COVID-19 cohort study within the stress ulcer prophylaxis research program. This figure displays how this COVID cohort study is integrated into the international stress ulcer prophylaxis research program

For peer review only - http://bmjopen.bmj.com/site/about/guidelines.xhtml

Table 1 Overview of research objectives, hypotheses, outcomes, and analytic methods

Objectives	Hypotheses	Outcomes	Methods of analysis
**Question 1:** What prognostically relevant demographic, biomarker, and clinical data characterize invasively ventilated patients with COVID-19? (Explanatory variable: not applicable; no comparisons)			
To establish a clinical profile of critically ill patients with COVID-19, incorporating demographics, biomarkers, rates of venous thromboembolism, COVID-19 therapies, duration of mechanical ventilation, ICU, hospital stay, and tracheostomy timing	Patients with COVID-19 will have a high burden of comorbidity and, as a consequence of their underlying severe viral illness, will exhibit high inflammatory biomarkers (D-dimer, CRP, ferritin), receive targeted COVID-19 therapies, experience long duration of mechanical ventilation, and undergo tracheostomy later than current norms	Description of demographics, biomarkers, and course in the ICU including venous thromboembolism rates and tracheostomy timing	Descriptive analysis<br>Continuous variables will be summarized using mean ± standard deviation (SD), while frequencies and percentages will be used to summarize categorical variables
**Question 2:** Do critically ill patients with COVID-19 have higher rates of clinically important GI bleeding, 90-day mortality, and infection compared to a propensity-matched non-COVID cohort? (explanatory variable: COVID-19)			
To understand the impact of COVID-19 on CIGIB, 90-day mortality, and rates of VAP, *C. difficile* infection, and patient-important bleeding; use of renal replacement therapy; and duration of mechanical ventilation, ICU stay and hospital stay, and ICU and hospital mortality compared to patients without COVID-19	Patients with COVID-19 will have a poorer prognosis than propensity-matched control patients without COVID-19. Patients with COVID-19 will have higher rates of CIGIB, 90-day mortality, VAP, *C. difficile* infection, and patient-important GI bleeding, use of renal replacement therapy, ICU and hospital mortality, and longer durations of mechanical ventilation and ICU and hospital stay, compared to a propensity-matched non-COVID cohort	CIGIB, 90-day mortality<br>VAP, *C. difficile* infection, patient-important bleeding, and renal replacement therapy<br>Duration of mechanical ventilation, ICU stay, and hospital stay<br>ICU and hospital mortality	Propensity-matched analysis of patients in REVISE with and without COVID-19 infection
**Question 3**: Do critically ill patients with COVID-19 projected to receive mechanical ventilation for > 48 h have different clinical outcomes with versus without PPIs? (explanatory variable: pantoprazole)			
To examine whether pantoprazole has a different treatment effect on CIGIB or 90-day mortality in patients with and without COVID-19 (REVISE subgroup analysis)<br>To determine whether pantoprazole has a differential treatment effect in patients with or without COVID-19 on VAP, *C. difficile* infection, patient-important GI bleeding, use of renal replacement therapy, hospital mortality, and duration of mechanical ventilation, ICU stay and hospital stay, and ICU and hospital mortality	We hypothesize no modification of the effect of pantoprazole on CIGIB. We hypothesize that pantoprazole is more harmful in patients with versus without SARS-CoV-2 in terms of increased risk of 90-day mortality<br>We hypothesize no modification of the effect of pantoprazole on VAP, *C. difficile* infection, patient-important GI bleeding, use of renal replacement therapy, and duration of mechanical ventilation, ICU stay and hospital stay, and ICU and hospital mortality. We hypothesize that pantoprazole is more harmful in patients with versus without SARS-CoV-2 in terms of increased risk of hospital mortality	CIGIB, 90-day mortality (REVISE subgroup analysis)<br>VAP, *C. difficile* infection, patient-important bleeding, and renal replacement therapy (extended outcomes)<br>Duration of mechanical ventilation, ICU stay and hospital stay, and ICU and hospital mortality (extended outcomes)	Subgroup analysis: Comparison of outcomes for patients with and without COVID-19 receiving either pantoprazole or placebo<br>Comparison of the time to the primary and secondary binary outcomes in the two groups using Cox proportional hazards regression with threshold *P*-values of 0.05, adjusted for the center and pre-hospital use of acid suppression (stratification variables). A test of interaction will be performed for this subgroup analysis in REVISE, and the results will be interpreted per the ICEMAN criteria<br>For binary outcomes, we will report hazard ratios with 95% CIs as well as the absolute risk increase or decrease and 95% CIs<br>For continuous outcomes, we will compare the two arms using a *t*-test on the log scale (to normalize the distribution) or a nonparametric approach if needed

Research question, objectives, hypotheses, outcomes, and analytic approaches for the COVID-19 REVISE substudy

CIGIB clinically important GI bleeding, *ICU* intensive care unit, *PPI* proton pump inhibitor, *COVID-19* coronavirus disease of 2019, *VAP* ventilator-associated pneumonia, *C. difficile Clostridioides difficile*

For peer review only

Table 2 Inclusion and exclusion criteria for REVISE

**Inclusion criterion**	
Adults ≥ 18 years old projected to receive invasive mechanical ventilation for ≥ 48 h according to the treating physician	
**Exclusion criteria**	
1. Already received invasive mechanical ventilation ≥ 72 h during this hospital admission	
2. Acid suppression for active GI bleeding or high risk of bleeding (e.g., current bleeding, peptic ulcer bleeding within 8 weeks, recent severe esophagitis, Barrett's esophagus, Zollinger-Ellison syndrome) [dyspepsia or gastroesophageal reflux is not an exclusion criterion]	
3. Acid suppression in the ICU for > 1 daily dose equivalent of a PPI or H2RA	
4. Dual antiplatelet therapy	
5. Combined antiplatelet and therapeutic anticoagulation	
6. Pantoprazole contraindication per local product information	
7. Palliative care or anticipated withdrawal of advanced life support	
8. Pregnancy	
9. Previous enrollment in REVISE, a related trial, or trial for which co-enrollment is prohibited	
10. Patient, proxy, or physician declines	

In this table, we list the eligibility criteria for this study, based on the inclusion and exclusion criteria for the REVISE trial

GI gastrointestinal, *ICU* intensive care unit, *PPI* proton pump inhibitor, *H2RA* histamine-2-receptor antagonists

be accepted for COVID-19 diagnosis, including antigen testing. Patients will be eligible for this substudy if they test positive for SARS-CoV-2 in the month preceding the index ICU admission, whether pre-hospital, in hospital, or in the ICU. Patients diagnosed as contracting SARS-CoV-2 more than 48 h after enrollment in REVISE will be ineligible.

Summary of outcomes for REVISE

The primary *efficacy* outcome for REVISE is clinically important GI bleeding occurring in the ICU or resulting in ICU readmission during the index hospital stay. Adjudicator agreement for this metric was excellent in two prior studies [17, 18] which was associated with increased attributable length of stay and mortality [19]. The definition of clinically important GI bleeding is overt GI bleeding (i.e., hematemesis, frank blood or coffee ground nasogastric aspirate, melena or hematochezia) *plus 1 of the following in the absence of other causes*: (1) hemodynamic change defined as a spontaneous decrease in invasively monitored mean arterial pressure or non-invasive systolic or diastolic blood pressure of ≥ 20 mmHg or an orthostatic increase in pulse rate of ≥ 20 beats/min and a decrease in systolic blood pressure of ≥ 10 mmHg, with or without vasopressor initiation or increase; (2) vasopressor initiation; (3) hemoglobin decrease of ≥ 2 g/dL (20 g/L) within 24 h of bleeding; (4) transfusion of ≥ 2 units red blood cells within 24 h of bleeding; or (5) therapeutic intervention (e.g., therapeutic endoscopy, angioemoblization, surgery). The primary *safety* outcome is mortality defined as all-cause mortality at 90 days after randomization to evaluate a suggestion of increased risk of death associated with pantoprazole among the sickest subgroup of patients in a recent placebo-controlled trial [20]. Secondary outcomes for REVISE are listed in Table 3.

Table 3 Secondary outcome definitions in REVISE

Ventilator-associated pneumonia	VAP is diagnosed in patients who received invasive mechanical ventilation for ≥ 48 h when there is a new, progressive, or persistent radiographic infiltrate *plus at least 2 of the following without other obvious cause*: (1) fever (temperature > 38 °C) or hypothermia (temperature < 36 °C), (2) leukopenia (< 4.0×10^6/L) or leukocytosis (> 12×10^6/L), (3) purulent sputum, or (4) gas exchange deterioration. Given no universally accepted VAP criteria, data collection and central adjudication allows analysis of several other VAP definitions. We do not collect infection-related ventilator-associated conditions as they are modifiable by ventilator settings and volume status
C. difficile infection	Defined as clinical features (diarrhea [≥ 3 episodes of unformed stools or Bristol type 6 or 7], ileus, or toxic megacolon) and either microbiological evidence of toxin-producing *C. difficile* or pseudomembranous colitis on colonoscopy in hospital
Patient-important GI bleeding	Defined as overt GI bleeding, *plus* an invasive intervention (e.g., therapeutic endoscopy, angioembolization, surgery), acknowledging how some clinically important GI bleeds in prior studies did not actually require any tests or treatments, and thus may not be important to patients, to be refined following completion of patient and family interviews from a Patient Important Bleeding Study [NCT05506150]
Renal replacement therapy	Defined as the initiation of new renal replacement therapy in the ICU
Hospital mortality	Defined as all-cause mortality in the hospital

In this table, we show the secondary outcomes and their definitions for the REVISE trial

VAP ventilator-associated pneumonia, *ICU* intensive care unit, *GI* gastrointestinal, *C. difficile Clostridioides difficile*

For peer review only

Pilot work

Investigators developed a one-page draft case report form to capture new information which was not part of the original REVISE trial to better characterize the COVID-19 cohort. A group of eight research coordinators reviewed and refined the form to capture COVID-19 vaccination status, biomarkers, venous thromboembolic events, tracheostomy occurrence and timing, and COVID-19 therapies as per Table 4. The form was pre-tested on 15 patients by abstracting data from either electronic or paper-based medical records. Accessing the chart, scanning contents, extracting relevant information, and entering data into the password-protected REVISE website required an estimated 40 min per patient. After this pilot exercise, we oriented research coordinators in participating centers to the study objectives, methods, and the case report form during telephone, videoconference, and in-person investigators' meetings.

Data collection and outcomes for the COVID-19 cohort study

Retrospective data collection for newly added variables for the COVID-19 study was required for participants enrolled prior to the implementation of this COVID-19 study protocol. Following relevant ethics review, COVID-19-specific variables were incorporated into existing data-collection forms, affording prospective data collection for COVID-19 variables for the remainder of the study. For any patients transferred to non-REVISE centers during the pandemic, all efforts are being made to complete the COVID-19 case report form, including a collection of data on outcomes occurring after transfer to a non-REVISE center.

Biologic specimens (e.g., sputum culture for COVID-19 testing) and laboratory results (blood samples to evaluate for hemoglobin levels) are collected as part of routine medical care. These samples are not being procured or stored for study purposes.

Data management and quality control

Data management and quality control will be overseen by the REVISE Methods Centers, located at McMaster University in Hamilton and The George Institute (TGI) in Sydney. These centers are responsible for ensuring optimal trial conduct. Oversight and quality control will be achieved through close collaboration between these centers, who hold twice monthly teleconferences to harmonize approaches and share management efficiencies. The Methods Center Leads and relevant investigators agreed to train local principal investigator(s), research coordinator(s), and research pharmacist(s), on the protocol. Throughout the trial, the methods center staff will engage in compliance-enhancing strategies, set targets, and track progress. REVISE analyst Ms. D Heels-Ansdell will conduct periodic central statistical monitoring to generate overall and site-specific metrics for internal use and site feedback. The methods center staff will adopt a risk-adapted approach to monitor each site (in-person or by videoconference per pandemic adaptations) to assess and enhance protocol fidelity. The foci will include eligibility adherence, stratification, informed consent documentation, regulatory compliance, and case report form accuracy.

In the event of any modifications made to the existing protocol for the COVID-19 substudy, the REVISE methods center will take responsibility for notifying all participating centers, sponsors, and funding institutions. All participating centers would receive a copy of the revised protocol and meet to review and discuss any implications of such changes to study implementation.

Sample size

The cohort size is based on the number of patients with COVID-19 enrolled in REVISE. As of June 1, 2023, 484 patients with COVID-19 have been enrolled in REVISE, representing 10.1% of the sample size of 4800 patients. Acknowledging the inherent challenges in predicting the incidence of and the means ± standard deviation (SD)

Table 4 COVID cohort substudy biomarkers and other clinical characteristics

New variables	Definitions and measures
**D-dimer**	Highest D-dimer level (μg/L) recorded post-COVID diagnosis
**C-reactive protein**	Highest C-reactive protein level (mg/L) recorded post-COVID diagnosis
**Ferritin level**	Highest ferritin level (μg/L) recorded post-COVID diagnosis
**Venous thromboembolism**	Defined as (1) new symptomatic or asymptomatic deep-vein thrombosis or pulmonary embolism, diagnosed using accepted imaging tests in practice
**Days of mechanical ventilation**	Defined as the number of days requiring mechanical ventilation either invasive or non-invasive
**Tracheostomy and timing of insertion**	Defined as time from intubation to tracheostomy insertion during the ICU stay

In this table, we list the additional data required for this COVID-19 C cohort study

COVID-19 coronavirus disease of 2019, *ICU* intensive care unit

For peer review only - http://bmjopen.bmj.com/site/about/guidelines.xhtml

or median (interquartile range [IQR]) of the continuous variables will be calculated. Baseline characteristics (e.g., age, sex, APACHE II Score, admission diagnostic category, comorbidities, COVID-19 vaccination status, and pre-hospital acid suppression) will be described. Table 1 presents the statistical analysis plans for the 3 study objectives, hypotheses, and outcomes.

For any outcome that is missing for more than 2% of patients, we will perform multiple imputation. Adjustments for multiple comparisons will not be made for these exploratory analyses. All analyses will be performed using the statistical software SAS version 9.4 [21].

Analysis of objective 1: Characterization of the COVID-19 cohort

New continuous variables added to the REVISE dataset to help characterize the COVID-19 cohort of patients include inflammatory biomarker levels and time until tracheostomy. New dichotomous variables include the incidence of thromboembolic events and treatment with COVID-19-specific therapies. Continuous variables will be summarized using mean ± standard deviation (SD), or median (interquartile range [IQR]), while frequencies and percentages will be used to summarize categorical variables.

Analysis of objective 2: Propensity-matched comparison of patients with and without COVID-19

To explore whether patients with COVID-19 experience different clinical outcomes relative to patients without COVID-19, we will compare the COVID-19 cohort and a propensity-matched non-COVID cohort from REVISE. Propensity score matching attempts to estimate the effect of COVID-19 status by accounting for important covariates that may predict or explain this status. Patients with COVID-19 will be matched with other patients within REVISE using propensity score analysis. We will consider an optimal matching ratio of 2:1 or 1:1 depending on the availability of optimal matches.

The propensity score will be generated using potentially important pre-randomization demographic variables, including age, sex, pre-hospital acid suppression (PPIs or histamine-2-receptor antagonists (H2RAs)), COVID-19 vaccination status, ICU admission category of medical or surgical/trauma, and date of enrollment. Propensity matching will only include patients enrolled after the declaration of the COVID-19 pandemic on March 11, 2020. As a sensitivity analysis, we will match only on pre-hospital PPI use, not H2RAs, given limited evidence to suggest that H2RAs influence susceptibility to COVID-19 infection [22].

Propensity score matching helps to ensure that the distribution of the foregoing covariates is similar between the compared populations [23]. Comparisons between the COVID-19 and matched cohorts will be made on clinically important bleeding and 90-day mortality, incidence of VAP, *C. difficile* infection, patient-important GI bleeding, renal replacement therapy, ICU and hospital mortality, and duration of mechanical ventilation and ICU and hospital stay. We will adjust all analyses for randomized allocation; mortality outcomes will be adjusted for the APACHE II score.

Objective 3: Treatment effect of PPIs in patients with COVID-19 versus other patients

We will explore whether pantoprazole in the ICU has a differential treatment effect on clinically important GI bleeding or 90-day mortality for patients with COVID-19 relative to those without COVID-19. This is a subgroup analysis of the primary REVISE trial. However, in addition, we aim to further evaluate the subgroup effects of pantoprazole in patients with COVID-19 across secondary REVISE outcomes including VAP and *C. difficile* infection, patient-important bleeding, renal replacement therapy, and ICU and hospital mortality, as well as the duration of mechanical ventilation and ICU and hospital stay.

This analysis will be conducted per the intention-to-treat principle. For time to the primary and secondary binary outcomes, we will use Cox proportional hazards regression, adjusted for pre-hospital use of acid suppression (a stratification variable). Analysis of our mortality outcomes will also be adjusted for the APACHE II score. An interaction term will be included in the model to test for this subgroup effect and results will be interpreted per the ICEMAN criteria [24]. We will report hazard ratios with 95% CIs as well as the absolute risk increase or decrease and 95% CIs. For continuous outcomes, we will perform linear regression which will include an interaction term. We will report mean differences with 95% CIs. If needed, we will perform the linear regression on the log-transformed outcome.

Ethics and regulatory approval

The main REVISE trial was approved in participating hospitals by all relevant ethics committees and regulatory authorities prior to starting enrollment. The Hamilton Integrated Research Ethics Board (HiREB) under Clinical Trials Ontario (CTO Study ID 1360) is the main REB of record, which also approved this substudy. Ethics review for the additional COVID-19-specific data collection was undertaken by the Research Ethics Board of record

For peer review only - http://bmjopen.bmj.com/site/about/guidelines.xhtml

(the Hamilton Integrated Research Ethics Board), and as needed to comply with local regulations in each participating center. In the absence of any modifications to the REVISE study design, this substudy involved additional data collection to characterize the patients with COVID-19 and facilitate additional future analyses. Therefore, no modifications were made to the consenting process for REVISE patients involved in this embedded COVID-19 study, and no requirements were issued to either reconsent or inform prior REVISE participants of the substudy. Specifically, there was no requirement to inform participants of this substudy, including an additional review of participants' medical records for new data collection. Participants previously randomized in REVISE were not re-consented to participate in the COVID-19 embedded substudy.

Integrated knowledge translation plan

After completion of the COVID-19 REVISE substudy, we will rapidly publish the results. All results will be published with open access to ensure wide dissemination and accessibility of study findings. We plan to publish and present the COVID-19 substudy work at an international congress. We will disseminate a lay summary of findings to the general public through health networks of patient advocates and persons with lived or living experiences. We will generate lay language summaries and visual abstracts for traditional (paper, radio, television) and social media. We will also host videoconferences and regional rounds. We will disseminate structured abstracts and slide decks to local ICU quality councils, provincial organizations (e.g., Alberta Health Services Critical Care Strategic Clinical Network), national policymakers (e.g., Accreditation Canada), and professional societies. Participating organizations, sponsors, and university institutions will be provided with press release briefs to disseminate to their respective audiences.

Discussion

This study, embedded within a global, randomized trial of stress ulcer prophylaxis, will fill important knowledge gaps in the literature that prompted our three research questions and three study objectives. The first is a descriptive analysis of the COVID-19 cohort in REVISE. The second is a propensity-matched analysis of patients with and without COVID-19. The third is an extended subgroup analysis of the REVISE trial, evaluating the effect of pantoprazole on a range of additional clinical outcomes among patients with and without COVID-19.

Evidence describing the association between acid suppression and poor prognosis among patients with COVID-19 has emerged, but results have been variable. To prevent complications among critically ill patients with COVID-19, interim WHO guidance in 2020 suggested H2RAs or PPIs for patients with risk factors for GI bleeding, including mechanical ventilation for ≥ 48 h, coagulopathy, renal replacement therapy, liver disease, multiple comorbidities, and higher organ failure score [25]. Surviving Sepsis Guidelines for the management of critically ill patients with COVID-19 do not address stress ulcer prophylaxis [26]. Randomized trial data on the effect of PPIs among patients with COVID-19 is insufficient to guide clinical practice, and this study will provide direct evidence on this topic.

Limitations of this substudy include the risk of missing 90-day outcomes due to high levels of inter-hospital transfers experienced during COVID-19 [27]. Patients transferred to centers not participating in REVISE or other loss to follow-up. Our propensity analysis cannot adjust for all possible factors predisposing to infection with SARS-CoV-2. We will have no information on patients eligible for REVISE who were not approached for the trial due to concerns about bleeding risk due to COVID-19 therapies or increased risk of VAP due to COVID-19 infection itself. In addition, the number of patients enrolled in REVISE with COVID-19 depends on the pandemic status in participating centers, such that no sample size calculation is possible; thus, we will interpret the findings in light of the 95% confidence intervals.

Strengths of this study include the timely development of a protocol addressing essential research questions relevant to a high-risk subgroup of critically ill mechanically ventilated patients that emerged during the pandemic. The main REVISE Trial [NCT03374800], and this COVID-19 Cohort Study [NCT05715567] are registered. We have specified a priori hypotheses for each of the 3 objectives. As this study was developed after the REVISE Trial began recruitment, we are using both retrospective and prospective data collection methods to identify relevant patients with COVID-19. All necessary data is derived from the hospital stay, except 90-day mortality for those discharged alive; thus, we anticipate minimal missing data.

Trial status

As of June 1, 2023, more than 90% of relevant data for REVISE patients with COVID-19 are in the REVISE database to inform this work. REVISE enrollment is anticipated to close byNovember 2023; 90-day follow-up, chart closure, and analyses for this study will follow later in 2024.

For peer review only - http://bmjopen.bmj.com/site/about/guidelines.xhtml

Acknowledgements
The trial was designed by the REVISE Steering Committee including the National and International Management Committees, the REVISE Investigators and Research Coordinators, the Canadian Critical Care Trials Group, and the Australian and New Zealand Intensive Care Society Clinical Trials Group. We are grateful to others at the Methods Center at McMaster University (Lisa Buckingham, France Clarke, Mary Copland, Karlo Matic, Ashley Sawyer, Alyson Takaoka) and The George Institute (Fatima Butt, Anna Cheng, Conrad Nangla, Fiona Osborne, Tina Schneider, Isabella Schoeler, Anna Tippet) for their expertise.

Authors' contributions
Concept and design: Y Arabi, A Binnie, D Cook, A Deane, B Dennis, J Dionne, S English, S Finfer, G Guyatt, M Hardie, D Heels-Ansdell, S Knowles, F Lauzier, J Marshall, J Muscedere, J Myburgh, M Ostermann, L Thabane, J Tsang, and N Zytaruk. Acquisition, analysis, or interpretation of the data: D Cook, B Dennis, M Hardie, D Heels-Ansdell, S Knowles, L Saunders, L Thabane, and N Zytaruk. Drafting of the manuscript: D Cook, B Dennis, D Heels-Ansdell, and N Zytaruk. Statistical analysis: B Dennis, D Heels-Ansdell, and L Thabane. Obtained funding: A Ahmed, A Binnie, D Cook, B Dennis, J Dionne, G Guyatt, D Heels-Ansdell, L Thabane, and J Tsang. Administrative, technical, or material support: D Cook, A Deane, B Dennis, S Finfer, G Guyatt, M Hardie, D Heels-Ansdell, S Knowles, F Lauzier, D Rajbhandari, L Saunders, A Poole, L Thabane, and N Zytaruk. Data integrity: D Cook, B Dennis, M Hardie, D Heels-Ansdell, S Knowles, L Saunders, and N Zytaruk. Ethics approval and consent to participate: not applicable for this retrospective chart review protocol. Approved final manuscript: all authors.

Funding
REVISE is funded by peer-reviewed grants [Canadian Institutes of Health Research 201610PJT-378226-PJT-CEBA-18373 and Canadian Institutes of Health Research 202207CL3-492565-CTP-CEBA-19215], the Hamilton Academy of Health Sciences Organization [HAH-22–009], Physician Services Incorporated (R22-33) and funds from St. Joseph's Healthcare Hamilton and McMaster University. The National Health and Medical Research Council of Australia grant [GNT1124675] funds enrollment in Australia. REVISE was approved by the National Institute for Health Research (NIHR) in the UK as a Portfolio Study [CPMS ID 45782], eligible for support from the NIHR Clinical Research Network. [https://www.nihr.ac.uk/researchers/collaborations-services-and-support-for-your-research/run-your-study/crn-portfolio.htm]. This trial received no support from the commercial or private sector. The funders/sponsors have no role in the conception, design, conduct, oversight, analysis, interpretation, write-up, review, or approval of the manuscript or the decision to submit the manuscript for publication.
Role of the funders: The funders/sponsors have no role in the conception, design, conduct, oversight, analysis, interpretation, write-up, review, or approval of the manuscript or the decision to submit the manuscript for publication.
Composition and responsibilities of participating centers and steering committee: Led by the Canadian Critical Care Trials Group and the Australian & New Zealand Intensive Care Society Clinical Trials Group, the REVISE Methods Centers are located at McMaster University in Hamilton for international centers and The George Institute in Sydney for Australia. These centers (REVISE Methods Centre, The George Institute) housed the primary data management and endpoint adjudication teams, who worked in close collaboration to ensure standardization of data collection and adjudication procedures. The REVISE Canadian Steering Committee comprises Drs. Guyatt, Cook, Alhazzani, Marshall, Lauzier, English, Muscedere, Hall, Xie, and Thabane. The International Management Committee includes Drs. Finfer and Myburgh, Deane, Ostermann, and Arabi joined by Canadian leaders Drs. Guyatt, Cook, Alhazzani, Marshall, and Thabane. These teams meet approximately quarterly, to anticipate and pre-empt challenges and generate solutions to the operational and financial hurdles characterizing global trials. The Data Safety & Monitoring Committee includes Professor Roberts (University of Oxford), Dr. McAuley (Queen's University Belfast), and Chair Dr. Thomlinson (University of Toronto).

Availability of data and materials
Readers are welcome to contact the research team for further information. There are no original data associated with this research protocol.
The datasets analyzed during the current study would only be available following relevant research ethics board discussion and approval. However, the statistical code will be available and any requested details of the protocol.

Declarations

Ethics approval and consent to participate
The main REVISE trial was approved in participating hospitals by all relevant ethics committees and regulatory authorities prior to starting enrollment. The Hamilton Integrated Research Ethics Board (HiREB) under Clinical Trials Ontario (CTO Study ID 1360) is the main REB of record, which also approved this substudy. Ethics review for the additional COVID-19-specific data collection was undertaken as needed to comply with local regulations in each participating center.

Consent for publication
Not applicable for this protocol.

Competing interests
All authors are involved in the REVISE Trial in some capacity. Otherwise, the authors declare that they have no competing interests.

Author details
[1]Department of Medicine, McMaster University, Hamilton, ON, Canada. [2]Department of Health Research Methods, Evidence, and Impact, McMaster University, Hamilton, ON, Canada. [3]Biostatistics Unit, St. Joseph's Healthcare Hamilton, Hamilton, ON, Canada. [4]Division of Critical Care, Research Institute, St. Joseph's Healthcare Hamilton, Hamilton, ON, Canada. [5]Departments of Medicine and Health Research Methods, Evidence, and Impact, McMaster University, McMaster University Health Sciences Center, Room 2C11, 1200 Main Street West, Hamilton, ON L8N 3Z5, Canada. [6]Department of Critical Care Medicine, Niagara Health System, St. Catharines, ON, Canada. [7]Department of Gastroenterology and Hepatology, Stanford University, Palo Alto, CA, USA. [8]Departments of Anesthesiology and Medicine and Critical Care Medicine, Université Laval, Québec, Québec, Canada. [9]Department of Critical Care Medicine, University of Melbourne, Melbourne Medical School, Parkville, VIC, Australia. [10]Intensive Care Department, Ministry of the National Guard-Health Affairs, Riyadh, Kingdom of Saudi Arabia. [11]King Saud Bin Abdulaziz University for Health Sciences, Riyadh, Kingdom of Saudi Arabia. [12]King Abdullah International Medical Research Center, Riyadh, Kingdom of Saudi Arabia. [13]Interdepartmental Division of Critical Care, University of Toronto, Toronto, ON, Canada. [14]Critical Care Program, The George Institute for Global Health, Faculty of Medicine, University of New South Wales, Sydney, NSW, Australia. [15]Intensive Care Unit, St. George Hospital, Sydney, Australia. [16]Department of Critical Care Medicine, Queen's University, Kingston, ON, Canada. [17]Department of Medicine, University of Ottawa, Ottawa, ON, Canada. [18]Clinical Epidemiology Program, Ottawa Hospital Research Institute, Ottawa, ON, Canada. [19]Department of Critical Care, King's College London, Guy's & St Thomas' Hospital, London, UK.

Received: 3 July 2023 Accepted: 16 August 2023
Published online: 30 August 2023

References
1. Richardson S, Hirsch JS, Narasimhan M, Crawford JM, McGinn T, Davidson KW, Barnaby DP, Becker LB, Chelico JD, Cohen SL, et al. Presenting characteristics, comorbidities, and outcomes among 5700 patients hospitalized with COVID-19 in the New York City area. JAMA. 2020;323(20):2052.
2. Guan W-J, Ni Z-Y, Hu Y, Liang W-H, Ou C-Q, He J-X, Liu L, Shan H, Lei C-L, Hui DSC, et al. Clinical characteristics of coronavirus disease 2019 in China. N Engl J Med. 2020;382(18):1708–20.
3. Zhang P, Zhu L, Cai J, Lei F, Qin J-J, Xie J, Liu Y-M, Zhao Y-C, Huang X, Lin L, et al. Association of inpatient use of angiotensin-converting enzyme inhibitors and angiotensin II receptor blockers with mortality among patients with hypertension hospitalized with COVID-19. Circ Res. 2020;126(12):1671–81.
4. Reynolds HR, Adhikari S, Pulgarin C, Troxel AB, Iturrate E, Johnson SB, Hausvater A, Newman JD, Berger JS, Bangalore S, et al. Renin–angiotensin–aldosterone system inhibitors and risk of COVID-19. N Engl J Med. 2020;382(25):2441–8.
5. Lopes RD, Macedo AVS, De BarrosESilva PGM, Moll-Bernardes RJ, Dos Santos TM, Mazza L, Feldman A, D'AndréaSabaArruda G, De Albuquerque

For peer review only - http://bmjopen.bmj.com/site/about/guidelines.xhtml

DC, Camiletti AS, et al. Effect of discontinuing vs continuing angiotensin-converting enzyme inhibitors and angiotensin II receptor blockers on days alive and out of the hospital in patients admitted with COVID-19. JAMA. 2021;325(3):254.
6. Xiang Y, Wong KC, So HC. Exploring drugs and vaccines associated with altered risks and severity of COVID-19: a UK Biobank cohort study of all ATC level-4 drug categories reveals repositioning opportunities. Pharmaceutics. 2021;13(9):1514.
7. Lee SW, Ha EK, Yeniova AÖ, Moon SY, Kim SY, Koh HY, Yang JM, Jeong SJ, Moon SJ, Cho JY, et al. Severe clinical outcomes of COVID-19 associated with proton pump inhibitors: a nationwide cohort study with propensity score matching. Gut. 2021;70(1):76–84.
8. Aby ES, Tappata M, Rodin H, Debes JD. Assessment of proton-pump inhibitor use, race, socioeconomic status, and mortality in individuals with COVID-19. Eur J Gastroenterol Hepatol. 2022;34(2):239.
9. Hariyanto TI, Prasetya IB, Kurniawan A. Proton pump inhibitor use is associated with increased risk of severity and mortality from coronavirus disease 2019 (COVID-19) infection. Dig Liver Dis. 2020;52(12):1410–2.
10. Krag M, Perner A, Wetterslev J, Wise MP, Borthwick M, Bendel S, McArthur C, Cook D, Nielsen N, Pelosi P, et al. Prevalence and outcome of gastrointestinal bleeding and use of acid suppressants in acutely ill adult intensive care patients. Intensive Care Med. 2015;41(5):833–45.
11. Cook D, Guyatt G. Prophylaxis against upper gastrointestinal bleeding in hospitalized patients. N Engl J Med. 2018;378(26):2506–16.
12. Re-EValuating the Inhibition of Stress Erosions (REVISE) Trial (REVISE), [NCT03374800]. Clinical trial registry from clinicalTrials.gov; 2018. https://classic.clinicaltrials.gov/ct2/show/NCT03374800. Accessed 28 Aug 2023.
13. Ippolito M, Misseri G, Catalisano G, Marino C, Ingoglia G, Alessi M, Consiglio E, Gregoretti C, Giarratano A, Cortegiani A. Ventilator-associated pneumonia in patients with COVID-19: a systematic review and meta-analysis. Antibiotics (Basel). 2021;10(5):545.
14. Dexamethasone in hospitalized patients with COVID-19. N Engl J Med. 2020;384(8):693–704. https://www.nejm.org/doi/full/10.1056/NEJMoa2021436.
15. Therapeutic anticoagulation with heparin in critically ill patients with COVID-19. N Engl J Med. 2021;385(9);777–89. https://www.nejm.org/doi/full/10.1056/NEJMoa2021436.
16. Chan AW, Tetzlaff JM, Altman DG, Laupacis A, Gøtzsche PC, Krleža-Jerić K, Hróbjartsson A, Mann H, Dickersin K, Berlin JA, et al. SPIRIT 2013 statement: defining standard protocol items for clinical trials. Ann Intern Med. 2013;158(3):200–7.
17. Cook D, Guyatt G, Marshall J, Leasa D, Fuller H, Hall R, Peters S, Rutledge F, Griffith L, McLellan A, et al. A comparison of sucralfate and ranitidine for the prevention of upper gastrointestinal bleeding in patients requiring mechanical ventilation. N Engl J Med. 1998;338(12):791–7.
18. Cook DJ, Fuller HD, Guyatt GH, Marshall JC, Leasa D, Hall R, Winton TL, Rutledge F, Todd T, Roy P, et al. Risk factors for gastrointestinal bleeding in critically ill patients. N Engl J Med. 1994;330(6):377–81.
19. Cook DJ, Griffith LE, Walter SD, Guyatt GH, Meade MO, Heyland DK, Kirby A, Tryba M. Crit Care. 2001;5(6):368.
20. Krag M, Marker S, Perner A, Wetterslev J, Wise MP, Schefold JC, Keus F, Guttormsen AB, Bendel S, Borthwick M, et al. Pantoprazole in patients at risk for gastrointestinal bleeding in the ICU. N Engl J Med. 2018;379(23):2199–208.
21. SAS 9.4. In. Edited by Inc SI. Cary, NC.
22. Hirota K. H_2 antagonists, proton pump inhibitors and COVID-19. J Anesth. 2022;36(3):329–31.
23. Rosenbaum PR, Rubin DB. The central role of the propensity score in observational studies for causal effects. Biometrika. 1983;70(1):41–55.
24. Schandelmaier S, Briel M, Varadhan R, Schmid CH, Devasenapathy N, Hayward RA, Gagnier J, Borenstein M, van der Heijden GJMG, Dahabreh IJ, et al. Development of the Instrument to assess the Credibility of Effect Modification Analyses (ICEMAN) in randomized controlled trials and meta-analyses. Can Med Assoc J. 2020;192(32):E901–6.
25. World Health O. Clinical management of severe acute respiratory infection (SARI) when COVID-19 disease is suspected: interim guidance, 13 March 2020. In. Geneva: World Health Organization; 2020.
26. Alhazzani W, Evans L, Alshamsi F, Møller MH, Ostermann M, Prescott HC, Arabi YM, Loeb M, Ng Gong M, Fan E, et al. Surviving sepsis campaign guidelines on the management of adults with coronavirus disease 2019 (COVID-19) in the ICU: first update. Crit Care Med. 2021;49(3):e219–34.
27. Pett E, Leung HL, Taylor E, Chong MSF, Hla TTW, Sartori G, Sathianathan V, Husain T, Suntharalingam G, Rosenberg A, et al. Critical care transfers and COVID-19: managing capacity challenges through critical care networks. J Intensive Care Soc. 2022;23(2):203–9.

Publisher's Note

Springer Nature remains neutral with regard to jurisdictional claims in published maps and institutional affiliations.

Ready to submit your research? Choose BMC and benefit from:

- fast, convenient online submission
- thorough peer review by experienced researchers in your field
- rapid publication on acceptance
- support for research data, including large and complex data types
- gold Open Access which fosters wider collaboration and increased citations
- maximum visibility for your research: over 100M website views per year

At BMC, research is always in progress.

Learn more biomedcentral.com/submissions

BMC

For peer review only

BMJ Open

Proton Pump Inhibitors in Invasively Ventilated Patients with SARS-CoV-2: A Substudy of the Re-EValuating the Inhibition of Stress Erosions (REVISE) Trial

Journal:	*BMJ Open*
Manuscript ID	bmjopen-2026-119937.R2
Article Type:	Original research
Date Submitted by the Author:	05-Aug-2026

Complete List of Authors:	Dennis, Brittany; The University of British Columbia, Department of Medicine<br>Heels-Ansdell, Diane; McMaster University, Department of Health Research Methods, Evidence and Impact<br>Quazi, Ibrahim; McMaster University, Department of Health Research Methods, Evidence and Impact<br>Basmaji, John; Western University, Department of Medicine, Division of Critical Care<br>Thabane, Lehana; McMaster University, Department of Health Research Methods, Evidence, and Impact; St. Joseph's Research Institute; St Joseph's Healthcare Hamilton, Biostatistics Unit<br>Guyatt, Gordon; McMaster University, Department of Medicine, Health Research Methods, Evidence and Impact,<br>Saunders, Lois; St. Joseph’s Healthcare , Research Institute<br>Hand, Lori; McMaster University, Department of Health Research Methods, Evidence and Impact<br>Loy, Olivia; The Royal Melbourne Hospital, Department of Critical Care<br>Zytaruk, Nicole; McMaster University, Health Research Methods, Evidence & Impact; St. Joseph’s Healthcare, Research Institute, Division of Critical Care<br>Hardie, Miranda; University of New South Wales, The George Institute for Global Health, Critical Care Program, Faculty of Medicine<br>Deane, Adam; The University of Melbourne, Department of Critical Care, Melbourne Medical School; The University of Melbourne - Parkville Campus, Intensive Care Unit, Department of Critical Care Medicine<br>Marshall, John; University of Toronto, Interdepartmental Division of Critical Care<br>Arabi, Yaseen; Ministry of National Guard Health Affairs, Intensive Care Department; King Saud bin Abdulaziz University for Health Sciences; King Abdullah International Medical Research Center<br>Lauzier, François; Laval University, Departments of Anesthesiology, Medicine and Critical Care Medicine<br>Dionne, Joanna; McMaster University, Departments of Medicine, Health Research Methods, Evidence, and Impact,<br>Burns, Karen; University of Toronto, Interdepartmental Division of Critical Care<br>Alhazzani, Waleed; Imam Abdulrahman Bin Faisal University, Critical Care and Internal Medicine Department, College of Medicine; Ministry of Defense Health Services, Research and Innovation Institute

	Vazquez-Grande, Gloria; University of Manitoba, Department of Critical Care<br>Geagea, Anna; North York General Hospital, Critical Care<br>Myburgh, John; University of New South Wales, Faculty of Medicine; The George Institute for Global Health, Critical Care Program; Saint George Hospital, Intensiv Care Unit<br>Muscedere, John; Queen’s University, Department of Critical Care Medicine<br>English, Shane; University of Ottawa, Department of Medicine; Ottawa Hospital Research Institute, Clinical Epidemiology Program<br>Binnie, Alexandra; William Osler Health System, Department of Critical Care Medicine<br>Tsang, Jennifer; McMaster University Department of Medicine; Niagara Health System, Department of Critical Care Medicine<br>Hunt, Miranda; Queen's University, Critical Care Medicine<br>Ostermann, Marlies; King's College London, Guy's & St Thomas' Hospital , Department of Critical Care<br>Al-Fares, Abdulrahman A; Al-Amiri Hospital, Department of Anesthesia, Critical Care, Pain Medicine and Kuwait Extracorporeal Life Support<br>Knowles, Serena; The George Institute for Global Health, Critical Care program<br>Ahmed, Aijaz; Stanford University, Department of Gastroenterology & Hepatology<br>Cook, Deborah; McMaster University, Departments of Medicine, Health Research Methods, Evidence and Impact; St. Joseph’s Healthcare Hamilton Research Institute<br>The REVISE Investigators, The Canadian Critical Care Trials Group; Sunnybrook and Women's College Health Sciences Centre

**Primary Subject Heading**:	Intensive care
Secondary Subject Heading:	Epidemiology, Infectious diseases, Respiratory medicine
Keywords:	COVID-19, Adult intensive & critical care < INTENSIVE & CRITICAL CARE, Research Design

SCHOLARONE™
MANUSCRIPTS

I, the Submitting Author has the right to grant and does grant on behalf of all authors of the Work (as defined in the below author licence), an exclusive licence and/or a non-exclusive licence for contributions from authors who are: i) UK Crown employees; ii) where BMJ has agreed a CC-BY licence shall apply, and/or iii) in accordance with the terms applicable for US Federal Government officers or employees acting as part of their official duties; on a worldwide, perpetual, irrevocable, royalty-free basis to BMJ Publishing Group Ltd ("BMJ") its licensees and where the relevant Journal is co-owned by BMJ to the co-owners of the Journal, to publish the Work in this journal and any other BMJ products and to exploit all rights, as set out in our licence.

The Submitting Author accepts and understands that any supply made under these terms is made by BMJ to the Submitting Author unless you are acting as an employee on behalf of your employer or a postgraduate student of an affiliated institution which is paying any applicable article publishing charge ("APC") for Open Access articles. Where the Submitting Author wishes to make the Work available on an Open Access basis (and intends to pay the relevant APC), the terms of reuse of such Open Access shall be governed by a Creative Commons licence – details of these licences and which Creative Commons licence will apply to this Work are set out in our licence referred to above.

Other than as permitted in any relevant BMJ Author's Self Archiving Policies, I confirm this Work has not been accepted for publication elsewhere, is not being considered for publication elsewhere and does not duplicate material already published. I confirm all authors consent to publication of this Work and authorise the granting of this licence.

For peer review only

Proton Pump Inhibitors in Invasively Ventilated Patients with SARS-CoV-2: A Substudy of the Re-EValuating the Inhibition of Stress Erosions (REVISE) Trial

Brittany Dennis MBBS, PhD Brittany.dennis@ubc.ca
Department of Medicine, University of British Columbia, Canada

Diane Heels-Ansdell, MSc ansdell@mcmaster.ca
Department of Health Research Methods, Evidence, and Impact, McMaster University, Hamilton, Ontario, Canada

Quazi Ibrahim, MSc ibrahiq@mcmaster.ca
Department of Health Research Methods, Evidence, and Impact, McMaster University, Hamilton, Ontario, Canada

John Basmaji, MD, MSc jbasmaji2013@gmail.com
Department of Medicine, Division of Critical Care, Western University, London, Ontario, Canada

Lehana Thabane, PhD thabanl@mcmaster.ca
Department of Health Research Methods, Evidence, and Impact, McMaster University, Hamilton, Ontario, Canada, Biostatistics Unit, St. Joseph's Healthcare Hamilton, Hamilton, Ontario, Canada
St. Joseph's Healthcare Hamilton Research Institute, Hamilton, Ontario, Canada

Gordon Guyatt, MD, MSc guyatt@mcmaster.ca
Department of Health Research Methods, Evidence, and Impact, McMaster University, Hamilton, Ontario, Canada
Department of Medicine, McMaster University, Hamilton, Ontario, Canada

Lois Saunders lsaunde@mcmaster.ca
Research Institute, St. Joseph's Healthcare Hamilton, Ontario, Canada

Lori Hand, RRT handlori@hhsc.ca
Department of Health Research Methods, Evidence, and Impact, McMaster University, Hamilton, Ontario, Canada

Olivia Loy, BSc, MBChB olivia.loy@nhs.net
Department of Critical Care, The Royal Melbourne Hospital, ICU

Nicole Zytaruk, RN zytarn@mcmaster.ca
Department of Health Research Methods, Evidence, and Impact, McMaster University, Hamilton, Ontario, Canada
Research Institute, Division of Critical Care, St. Joseph's Healthcare Hamilton, Ontario, Canada

Miranda Hardie, MClinEpi mhardie@georgeinstitute.org.au
Critical Care Program, The George Institute for Global Health, Faculty of Medicine, University of New South Wales, Sydney, New South Wales, Australia

Adam M Deane, MD Adam.Deane@mh.org.au
Intensive Care Unit, University of Melbourne, Melbourne, Victoria, Australia
Department of Critical Care Medicine, University of Melbourne Parkville Campus, Victoria, Australia

John C Marshall, MD John.Marshall@unityhealth.to
Interdepartmental Division of Critical Care, University of Toronto, Toronto, Ontario, Canada

For peer review only - http://bmjopen.bmj.com/site/about/guidelines.xhtml

Yaseen Arabi, MD Arabi@mngha.med.sa
Intensive Care Department, Ministry of the National Guard-Health Affairs, Riyadh, Kingdom of Saudi Arabia
King Saud bin Abdulaziz University for Health Sciences, Riyadh, Kingdom of Saudi Arabia
King Abdullah International Medical Research Center, Riyadh, Kingdom of Saudi Arabia

François Lauzier, MD, MSc Francois.Lauzier@fmed.ulaval.ca
Departments of Anesthesiology, Medicine and Critical Care Medicine, Université Laval, Québec, Québec, Canada

Joanna C Dionne MD, MSc, PhD dionnejc@mcmaster.ca
Department of Medicine, McMaster University, Hamilton, Ontario, Canada
Departments of Health Research Methods, Evidence, and Impact, McMaster University, Hamilton, Ontario, Canada

Karen EA Burns, MD, MSc karen.burns@unityhealth.to
Interdepartmental Division of Critical Care, University of Toronto, Toronto, Ontario, Canada

Waleed Alhazzani, MD, MSc waleed.al-hazzani@medportal.ca
Critical Care and Internal Medicine Department, College of Medicine, Imam Abdulrahman Bin Faisal University, Dammam, Eastern Province, Saudi Arabia
Research and Innovation Institute, Ministry of Defense Health Services, Riyadh, Saudi Arabia

Gloria Vazquez-Grande, MD, PhD gloriavazquezgrande@gmail.com
Department of Critical Care, University of Manitoba, Winnipeg, Manitoba, Canada

Anna Geagea, MD, PhD anna.geagea@nygh.on.ca
Department of Critical Care, North York General Hospital, North York, Ontario, Canada

John Myburgh, MD, PhD jmyburgh@georgeinstitute.org.au
Faculty of Medicine, University of New South Wales, Sydney, New South Wales, Australia
Critical Care Program, The George Institute for Global Health, Sydney, New South Wales, Australia
Intensive Care Unit, St. George Hospital, Kogorah, New South Wales, Australia

John Muscedere, MD John.Muscedere@kingstonhsc.ca
Department of Critical Care Medicine, Queen's University, Kingston, Ontario, Canada

Shane English MD, MSc senglish@toh.ca
Department of Medicine, Division of Critical Care, University of Ottawa, Ottawa, Ontario Canada
Clinical Epidemiology Program, Ottawa Hospital Research Institute, Ottawa, Ontario, Canada

Alexandra Binnie MD, PhD allie@binnie.ca
Department of Critical Care Medicine, William Osler Health System, Brampton, Ontario, Canada

Jennifer LY Tsang MD, PhD Jennifer.Tsang@niagarahealth.on.ca
Department of Medicine, Division of Critical CareMcMaster University, Hamilton, Ontario, Canada
Department of Critical Care Medicine, Niagara Health System, St. Catharines, Ontario, Canada

Miranda Hunt, BA Miranda.hunt@kingstonhsc.ca
Department of Critical Care Medicine, Queen's University, Kingston, Ontario, Canada

Marlies Ostermann, MBBS Marlies.Ostermann@gstt.nhs.uk

For peer review only - http://bmjopen.bmj.com/site/about/guidelines.xhtml

Department of Critical Care, King's College London, Guy's & St Thomas' Hospital, London, UK

Abdulrahaman A Al-Fares, MD abdulrahman.alfares@gmail.com
Departments of Anesthesia, Critical Care, Pain Medicine and Kuwait Extracorporeal Life Support Program, Al-Amiri Hospital, Kuwait City, Al Asimah Governate, Kuwait

Serena Knowles, PhD sknowles@georgeinstitute.org.au
Critical Care Program, The George Institute for Global Health, Newton, New South Wales, Australia

Aijaz Ahmed, MD safia93@stanford.edu
Department of Gastroenterology and Hepatology, Stanford University, Palo Alto, California, USA

*Deborah J Cook, MD, MSc debcook@mcmaster.ca
Department of Medicine, McMaster University, Hamilton, Ontario, Canada
Department of Health Research Methods, Evidence, and Impact, McMaster University, Hamilton, Ontario, Canada
St. Joseph's Healthcare Hamilton Research Institute, Hamilton, Ontario, Canada

For the REVISE Investigators and the Canadian Critical Care Trials Group

*Correspondence: DJ Cook, Departments of Medicine & Health Research Methods, Evidence and Impact, McMaster University Health Sciences Center, Room 2C11, 1200 Main Street West, Hamilton, Ontario, Canada, L8N 3Z5; debcook@mcmaster.ca

For peer review only

Proton Pump Inhibitors in Invasively Ventilated Patients with SARS-CoV-2: A Substudy of the Re-EValuating the Inhibition of Stress Erosions (REVISE) Trial

ABSTRACT

Objectives: Observational studies suggest that acid suppression may worsen outcomes among patients infected with severe acute respiratory syndrome coronavirus-2 (SARS-CoV-2). The objectives of this embedded substudy of a randomized controlled trial evaluating pantoprazole in mechanically ventilated patients were to (1) describe the clinical characteristics of critically ill patients with SARS-CoV-2, (2) compare clinical outcomes with a propensity-matched non-infected cohort, and (3) assess whether pantoprazole's treatment effects differed by SARS-CoV-2 infection status.

Design: Pre-planned substudy of the REVISE trial including a propensity-matched analysis of infected and non-infected patients, comparing the effect of pantoprazole between patients with and without SARS-CoV-2.

Setting: 68 intensive care units (ICUs) in 8 countries.

Participants: From July 2019 to October 2023, 4,821 eligible participants were enrolled in REVISE whether or not they had SARS-CoV-2 infection.

Primary and Secondary Outcome Measures: Participants enrolled in REVISE with SARS-CoV-2 infection had additional data collection including biomarkers, venous thromboembolism, SARS-CoV-2 therapies, and tracheostomy timing. The primary outcomes were clinically important upper gastrointestinal bleeding and 90-day mortality. Secondary outcomes included ventilator-associated pneumonia, *C. difficile* infection, patient important upper GI bleeding, renal replacement therapy, ICU and hospital mortality, and duration of mechanical ventilation, ICU stay, and hospital stay.

Results: Of the eligible trial cohort, 11.9% (540/4550) had SARS-CoV-2; 532 patients had additional SARS-CoV-2-specific data collection. Of these 532 patients, 87.8% received Covid-19-directed treatments – (dexamethasone 75.2%), 11.7% developed pulmonary embolism and 9.2% developed deep-vein thrombosis. After propensity-matching, SARS-CoV-2 infection was not associated with clinically important upper gastrointestinal bleeding (adjusted Hazard Ratio [aHR] 0.78, 95% Confidence Interval [CI] 0.40–1.50), but was associated with significantly higher ICU, hospital, and 90-day mortality, as well as longer duration of ventilation, ICU and hospital length of stay. The effect of pantoprazole on clinically important upper GI bleeding and 90-day mortality was consistent regardless of SARS-CoV-2 status.

Conclusions: SARS-CoV-2 infection was associated with higher mortality and longer duration of mechanical ventilation, ICU and hospital stay, without an increased risk of clinically important upper gastrointestinal bleeding. Pantoprazole reduced clinically important upper gastrointestinal bleeding without adversely affecting other outcomes.

Trial Registration: REVISE Trial [NCT03374800], SARS-CoV-2 Cohort Study [NCT05715567]
Abstract Word Count: 338
Manuscript Word Count: 3475
Figure: 1; **Tables:** 4; **Supplemental Tables**: 4; **Supplemental Material**: 1
References: 22

Article Summary

Strengths and Limitations of this Study:

- This SARS-CoV-2 Cohort substudy was embedded in a large randomized trial, registered, and guided by a published protocol and statistical analysis plan with pre-specified hypotheses.
- This substudy offers the first randomized trial data on the effect of PPIs among patients with SARS-CoV-2 on a range of clinical outcomes.
- We lack information about SARS-CoV-2 variants and SARS-CoV-2 vaccine timing.

For peer review only - http://bmjopen.bmj.com/site/about/guidelines.xhtml

- Our definition of COVID-19 is limited to patients who received COVID-19 specific treatment; other infected patients may not have been symptomatic.
- The propensity analysis could not adjust for all possible factors predisposing to SARS-CoV-2 leading to possible unrecognized confounding.

For peer review only

For peer review only - http://bmjopen.bmj.com/site/about/guidelines.xhtml

INTRODUCTION

Early in the pandemic, proton pump inhibitors (PPIs) were hypothesized to influence the susceptibility to severe acute respiratory syndrome coronavirus-2 (SARS-CoV-2) and result in worse clinical outcomes among infected patients, although findings were inconsistent. Among older adults, early observational data suggested that recent exposure to PPIs was associated with a reduced risk of infection. [1] However, several retrospective studies subsequently raised concern that antacid use among SARS-CoV-2 infected patients may worsen clinical outcomes.[2-4] Across large claims-based and hospital-based retrospective cohorts, PPI use was linked to a higher risk of severe coronavirus disease of 2019 (COVID-19), ICU admission, mechanical ventilation, and death, although some associations were attenuated after adjustment for age and comorbidities. [2, 3] Consistent with these signals, a pooled analysis of retrospective studies also suggested an increased risk of mortality associated with COVID-19 among PPI users,[4] as well as a more severe course of infection. [5]

To reduce the risk of upper gastrointestinal (GI) bleeding, clinicians often administer PPIs prophylactically in critically ill patients.[6] During the COVID-19 pandemic, international guidance for the management of these patients supported use of PPIs or histamine-2-receptor antagonists (H2Ras) in high-risk patients,[7] yet sepsis guidance did not directly address stress ulcer prophylaxis,[8] and in the early pandemic period, randomized trials had not yet evaluated whether PPIs affected clinical outcomes for ICU patients with COVID-19. Given unclear guidelines for COVID-19 and practices during the pandemic that could have increased bleeding risk (e.g., anticoagulation [9], high-dose corticosteroids [10]) and an apparent increased risk of ventilator-associated pneumonia [11], we conducted a planned substudy within the Re-EValuating the Inhibition of Stress Erosions (REVISE) trial to evaluate the association between PPI exposure and clinically important outcomes in ICU patients tested positive for SARS-CoV-2.[12]

This prospective substudy was pre-planned, nested within an international randomized blinded trial in critically ill patients, investigating the effect of pantoprazole 40 mg intravenously compared to placebo on the primary *efficacy* outcome of clinically important upper GI bleeding and the primary *safety* outcome of 90-day mortality. REVISE found that pantoprazole significantly lowered the risk of clinically important and patient important upper GI bleeding in invasively ventilated patients, but had no effect on other outcomes including 90-day mortality, ventilator-associated pneumonia, *Clostridioides difficile (C. difficile*) infection, renal replacement therapy, or mortality at ICU and hospital discharge. [13] While testing positive for SARS-CoV-2 during hospital admission did not influence treatment effect for the primary outcome,[13] the effect of pantoprazole in patients with COVID-19 requiring treatment has not been determined on ventilator-associated pneumonia, *C. difficile* infection, and other outcomes in patients.

As SARS-CoV-2 is now endemic and PPIs are commonly prescribed in the ICU, understanding the relationship between PPI exposure and clinical outcomes in this population warrants investigation. Accordingly, within this multi-center SARS-CoV-2 substudy of the REVISE trial [NCT05715567],[12] we (1) describe the clinical characteristics and hospital admission trajectory of critically ill patients tested positive for SARS-CoV-2, (2) compare clinical outcomes with a propensity-matched non-infected cohort, and (3) assess whether pantoprazole's treatment effects differed by COVID-19 status.

METHODS

Nested Cohort Study Design

REVISE was registered [NCT03374800], and the trial design [14], statistical analysis plan, [15] and pandemic adaptations[16] were previously published, as designed by the Canadian Critical Care Trials Group and the Australian and New Zealand Intensive Care Society Clinical Trials Group. Overall, 4,821

For peer review only - http://bmjopen.bmj.com/site/about/guidelines.xhtml

patients undergoing invasive mechanical ventilation were enrolled in 68 ICUs in Canada, Australia, Saudi Arabia, United Kingdom, United States, Kuwait, Pakistan and Brazil from July 2019 to October 2023.

This multi-center cohort design was registered [NCT05715567], funded, and guided by a protocol and statistical analysis plan.[12] (**Supplemental Material 1**) We included patients in this substudy if they were enrolled in REVISE on or after March 11, 2020. Notwithstanding the operational constraints imposed by the SARS-CoV-2 pandemic on ICU-based research,[17] REVISE enrollment experienced only temporary recruitment suspensions at participating centers, and did not impact eligibility criteria: patients both with and without SARS-CoV-2 were included.[16] The substudy protocol outlined the objectives, *a priori* hypotheses, and outcomes[12], summarized below.

Trial Intervention

Study drug (40 mg intravenous pantoprazole or matching placebo) was prescribed in the ICU until discontinuation of invasive mechanical ventilation, the occurrence of a prespecified indication or contraindication to PPI use, or death, up to 90 days.

Outcomes

The clinical outcomes for REVISE and this substudy were outlined in the trial registration [NCT03374800], trial protocol[14], trial statistical analysis plan,[15] trial report,[13] and protocol for this substudy.[12]

Characterizing Patients with COVID-19

Briefly, patients were eligible for this substudy according to the inclusion and exclusion criteria of REVISE.[13] Inclusion in the SARS-CoV-2 cohort was based on SARS-CoV-2 viral nucleic acid test positivity by polymerase chain reaction (PCR) from a nasopharyngeal swab, oropharyngeal swab, sputum, endotracheal aspirate, or bronchoalveolar lavage sample, as clinically determined rather than per protocol.[12] Patients were eligible if they tested positive for SARS-CoV-2 in hospital (whether contracted preceding the index ICU admission, pre-hospital, or in hospital).

Patients were eligible for this substudy if they tested positive. These patients contributed to objective 1. However, only patients receiving COVID-19 specific treatment were considered symptomatic for SARS-CoV-2 and were included in analyses pertaining to objectives 2 and 3.

No additional tests or treatments were protocolized, and care was at the discretion of the treating team. Additional data collection on SARS-CoV-2 positive patients included biomarkers, venous thromboembolic events, tracheostomy occurrence and timing, and COVID-19 therapies. In the ICU, detailed daily data collection occurred. On the ward thereafter, *C. difficile* infection, vital status and hospital length of stay were captured. Mortality was documented at 90 days.

Covariates for Matching

The patients' age was abstracted from the medical record in years. Biological sex was abstracted from the medical record; gender was not considered. Pre-hospital acid suppression and use of corticosteroids in the week pre-randomization was documented by research personnel from the patient's medical record, clarified with a hospital or community pharmacist or substitute decision-maker if necessary. The ICU admission category of medical vs surgical/trauma was classified by research personnel based on the APACHE III admission diagnostic categories. Days from the start of pandemic to enrollment was calculated from March 11, 2020.

Analysis

As per the substudy statistical analysis plan [12], to characterize the cohort of patients tested positive for

For peer review only - http://bmjopen.bmj.com/site/about/guidelines.xhtml

SARS-CoV-2 within the REVISE trial (*Objective 1*), we used descriptive statistics. Continuous variables were analyzed and presented using mean ± standard deviation (SD), or median (interquartile range [IQR]) for non-normally distributed or skewed data. Categorical variables are presented using frequencies and percentages.

We examined associations between SARS-CoV-2 status and clinical outcomes after adjusting for age, sex, pre-hospital acid suppression, ICU admission category (medical or surgical/trauma), corticosteroids one week pre-randomization, and days from start of pandemic to enrollment (*Objective 2*). Propensity matching only considered patients enrolled after or on March 11th, 2020.

We implemented two methods to address confounding: 1) multivariable regressions and 2) an optimal propensity score matching (PSM) of 2:1 ratio of non-COVID-19 patients with COVID-19 patients. The score was the probability of COVID-19 predicted by the confounders. This method identified the set of matched triplets that minimized the overall difference in propensity scores across all possible matching combinations. We used optimal propensity matching at a 2:1 ratio, matching two non-COVID patients to each COVID patient. This method identified the set of matched triplets that minimized the overall difference in propensity scores across all possible matching combinations. While multivariable regression was not originally proposed in the protocol, we added it to evaluate the adjusted association between COVID-19 and clinical outcomes while preserving sample size lost from matching. Using Cox-proportional hazards regressions, we studied the association between COVID-19 and clinically important upper GI bleeds, incident *C difficile*, new renal replacement therapy, patient important upper GI bleeding, ventilator-associated pneumonia, and mortality in ICU, hospital, and within 90 days. Ventilator-associated pneumonia was classified in this substudy using the Clinical Pulmonary Infection Score as in the main trial report. The proportional hazard (PH) assumption was checked using interaction of an explanatory variable with log (time). In case the PH assumption was violated for an explanatory variable, an interaction of that variable with log time was considered in the model and relative effects, hazard ratios, were reported at different timepoints. ICU and hospital length of stay, and duration of mechanical ventilation were highly skewed to the right. Thus, natural logarithms of these three variables were regressed on COVID-19 status and *a priori* confounders and adjusted (exponentiated) relative effect of COVID-19 on length of stays were reported. For propensity-matching analysis, patients within a matched triplet were correlated. To account for correlation in the time-to-event analyses, a robust sandwich type variance estimator for clusters was used. For the length-of-stay outcomes, to account for within matched triplet (cluster) correlations, random intercepts models were used in multivariable linear regressions. All analyses were adjusted for randomized allocation; mortality was also adjusted for APACHE II score. A sensitivity analysis was originally planned to evaluate only pre-hospital PPI use (not H2RAs); however, this was not performed, as less than 1% of patients used H2RAs.[13]

Responding to unexpected findings of a lower risk of ventilator-associated pneumonia among patients with COVID-19 compared to those without COVID-19, exploratory post hoc analyses further evaluated the association between SARS-CoV-2 status and ventilator-associated pneumonia. Both the propensity-matched analysis and the multivariable regression models for the outcome of ventilator-associated pneumonia were revised, adjusting for baseline pneumonia at ICU admission, alongside prespecified confounders and randomized assignment (pantoprazole vs placebo) included in the original models. A second post hoc analysis to evaluate the association between COVID-19 status and ventilator-associated pneumonia was repeated after excluding patients with baseline pneumonia. This involved using both propensity-matching and multivariable regression methods. Propensity scores were re-estimated in the reduced cohort and prespecified models were refit. Additional exploratory post hoc analyses evaluated the association between COVID-19 status and clinically important upper GI bleeds, incident *C. difficile*, new renal replacement therapy, patient important upper GI bleeding, and ventilator-associated pneumonia, accounting for the competing risk of death.

For peer review only - http://bmjopen.bmj.com/site/about/guidelines.xhtml

Finally, we examined whether pantoprazole had a differential effect on clinically important upper GI bleeding or 90-day mortality among patients with COVID-19 compared with those without (*Objective 3*), using a prespecified subgroup analysis of REVISE.[15] Subgroup effects were also evaluated for secondary trial outcomes of ventilator-associated pneumonia, *C. difficile* infection, patient important upper GI bleeding, renal replacement therapy, ICU and hospital mortality, and duration of mechanical ventilation, ICU stay, and hospital stay. For time-to-event outcomes, subgroup analyses were conducted using Cox proportional hazards models that included COVID-19 status, randomized treatment, and an interaction term between SARS-CoV-2 status and treatment. Analyses followed the intention-to-treat principle, with separate models fitted for each outcome. Models were adjusted for pre-hospital acid suppression use (either PPI or H2RA; exposure to either was a stratification variable), and mortality models were additionally adjusted for APACHE II score. As per the REVISE trial statistical analysis plan, if any subgroup effects were identified, using the interaction term when p-values were <0.10, we planned to assess the credibility of subgroup effects, interpreted using ICEMAN.[18] Results were reported as hazard ratios [HR]with 95% confidence intervals [CI]. For duration outcomes (mechanical ventilation, ICU stay, and hospital stay), subgroup analyses were performed using linear regression models on the natural logarithms of the outcomes including COVID-19 status, randomized treatment, their interaction term, and pre-hospital acid suppression.

Given negligible missing data (<2%), we did not perform imputation.[13] As specified in the protocol, we did not adjust for multiple comparison adjustments. Analyses were conducted using SAS version 9.4 [19] and STATA 15.1 (College Station, Texas).[19]

Patient and Public Involvement

Members of the public who were substitute decision makers for critically ill patients, and sometimes critically ill patients themselves, were involved in the informed consent process to participate in the parent REVISE trial. One outcome reported herein (patient important bleeding) was developed by ICU survivors and family members of survivors or decedents [13].

Ethics

Relevant ethics committees and regulatory authorities in participating hospitals approved REVISE. The Hamilton Integrated Research Ethics Board under Clinical Trials Ontario (CTO Study ID 1360), the main board of record, also approved this substudy.

RESULTS

Of 4,821 enrolled patients in the trial, 264 participants were not eligible from this substudy due to pre-pandemic randomization. An additional 7 patients were excluded due to declined or revoked consent, or whose SARS-CoV-2 status remained unknown, resulting in a final cohort of 4,550 patients. Within the remaining study cohort, 11.9% (540/4,010) were tested positive for SARS-CoV-2 at any time during hospitalization. The SARS-CoV-2 -specific case report form was not completed in 8 patients in one center who were therefore excluded in analyses for Objective 1 examining treatments and other characteristics. There were an additional 65 patients for whom the SARS-CoV-2 form indicated no SARS-CoV-2-directed treatment. Because COVID-19 status could not be definitively classified for these 65 patients, they were included in Objective 1 analyses but excluded from comparative analyses (Objective 2: propensity-matched analyses, Objective 3: REVISE SARS-CoV-2 subgroup analyses). Accordingly, the final analytic sample for Objectives 2 and 3 comprised 4,477 patients, including 4,010 non–SARS-CoV-2 patients and 467 COVID-19 patients (Figure 1).

Characteristics of Participants with SARS-CoV-2

Among the 532 patients with complete information on their SARS-CoV-2 status (i.e., treatments

For peer review only - http://bmjopen.bmj.com/site/about/guidelines.xhtml

received) enrolled in the REVISE trial, 268 were randomized to pantoprazole, and 264 to placebo. Table 1 shows the characteristics of the overall SARS-CoV-2 Cohort. Briefly, almost half of infected participants were unvaccinated *(n*=226, 42.5%). Treatments for COVID-19 were administered to most participants (*n*=467, 87.8%); dexamethasone was most commonly prescribed (*n*=400, 75.2%).

Complications of SARS-CoV-2 were common (e.g., pulmonary embolism and deep vein thrombosis occurred in 62 (11.7%), and 49 (9.2%) participants, respectively). Biomarkers after enrolment captured in this cohort demonstrated high rates of inflammation, with median d-dimer levels at 2855.5 mg/L (IQR: 1180-5000); C-reactive protein of 156.1 mg/dL (IQR: 78.2-243.5); and ferritin of 883 ng/mL (IQR 403-1870).

Comparison of Participants with and without COVID-19

Clinical characteristics and ICU admission data for the COVID-19 cohort are summarized in Table 2. COVID-19 patients were primarily admitted to ICU for medical reasons (COVID-19: 440, 94.2% vs. Non-SARS-CoV-2: 2,819, 70.3%), and most received corticosteroids in the week preceding randomization (COVID-19: 362, 77.5% vs. Non-SARS-CoV-2: 1,206, 30.1%). Evaluation of *a priori* selected variables used in the propensity score model demonstrated that baseline variables were significantly associated with COVID-19 status (Supplementary Table S1). After propensity-matching, patients with COVID-19 and without SARS-CoV-2 were well balanced across baseline demographic and clinical characteristics, as demonstrated in Supplementary Table S2.

No differences in the propensity-matched analysis were observed between patients with COVID-19 and without SARS-CoV-2 for clinically important upper GI bleeding (adjusted HR [aHR]: 0.78, 95% CI 0.40, 1.50, p=0.45). However, COVID-19 patients had 73% higher hazard of mortality at 30 days compared to non-SARS-CoV-2 propensity-matched patients (aHR: 1.73; 95% CI: 1.06, 2.40). At 60 days, this increased to 150% higher hazard (aHR: 2.50; 95% CI: 1.13, 3.87), and further to 210% at 90-days (aHR: 3.10; 95% CI: 1.10, 5.10). Similarly, we observed higher mortality among COVID-19 patients, both during hospitalization and throughout the 90-day follow-up (Table 3).

Other outcomes including incident C. *difficile* infection, patient important upper GI bleeding, and new renal replacement therapy were not associated with COVID-19 status (Table 3). However, incident infection with VAP appeared to be lower amongst participants who had COVID-19 (aHR: 0.50; 95% CI: 0.37, 0.69).

Post hoc exploratory adjustment for baseline pneumonia diminished the initial apparent association between COVID-19 status and ventilator-associated pneumonia (Supplementary Table S3) (aHR: 1.08; 95% CI: 0.78, 1.50). Further, no association between COVID-19 status and ventilator-associated pneumonia was observed after excluding patients with pneumonia at baseline from the propensity-matched analysis (Supplementary Table S3). Competing risk analyses yielded similar results to those presented in Table 3 and Supplemental Table S3.

COVID-19 was associated with a 45% longer ICU stay (adjusted ratio of length of stay 1.45; 95% CI: 1.33, 1.58, p<0.001), a 30% longer hospital stay (adjusted ratio 1.30; 95% CI: 1.18, 1.43, p<0.001) and a 69% increase in the number of days of mechanical ventilation (adjusted ratio of duration 1.69; 95% CI: 1.53, 1.87, p<0.001)

Overall, results were consistent between multivariable regression models and the primary propensity-matched analyses (Table 3, Supplemental Tables S3 and S4).

Substudy Subgroup Analyses

For peer review only - http://bmjopen.bmj.com/site/about/guidelines.xhtml

COVID-19 status in subgroup analyses did not suggest an effect modification of pantoprazole on the primary efficacy outcome of clinically important upper GI bleeding (Non-SARS-CoV-2 HR: 0.30: 95% CI: 0.18, 0.49; COVID-19 HR: 0.27: 95% CI: 0.08, 0.95, interaction p-value: 0.868) or the primary safety outcome of 90-day mortality (Non-SARS-CoV-2 HR: 0.97: 95% CI: 0.86, 1.09; COVID-19 HR: 0.94: 95% CI: 0.69, 1.27, interaction p-value: 0.855). Similarly, no differences were observed for other outcomes of patient important upper GI bleeding, renal replacement therapy, VAP, *C. difficile* infection in hospital, ICU and hospital mortality, duration of mechanical ventilation, ICU stay, or hospital length of stay. Table 4 summarizes the results of all subgroup analyses.

DISCUSSION

Among 4,550 patients in this stress ulcer prophylaxis trial enrolled after the beginning of the pandemic, 11% had were tested positive for SARS-CoV-2, most of whom received directed therapies and were deemed to have COVID-19. These patients frequently developed thrombotic complications, and biomarkers demonstrated an intense inflammatory burden. After propensity-matching with patients not tested positive for SARS-CoV-2, COVID-19 infection was not associated with clinically important upper GI bleeding, but was associated with higher ICU, hospital, and 90-day mortality and longer duration of mechanical ventilation, and ICU and hospital length of stay. The effect of pantoprazole reducing clinically important upper GI bleeding with no effect on 90-day mortality was consistent irrespective of COVID-19 status. Overall, we found no evidence that SARS-CoV-2 modified the treatment effect of pantoprazole on any outcome.

This study evaluated several outcomes in patients with COVID-19 that have not been well studied, including *C. difficile* infection, patient important upper GI bleeding, and new renal replacement therapy. Our initial analyses suggested that COVID-19 was associated with a lower risk of ventilator-associated pneumonia, inconsistent with previous literature.[11, 20-22] In fact, most studies characterizing COVID-19 patients found a high rate of secondary pneumonia with an incidence ranging between 18-39/1,000 mechanically ventilated days.[20-22] A high risk of ventilator-associated pneumonia was also observed in a systematic review by Ippolito et. al, which found a pooled rate of 45.4% (95% C.I. 37.8-53.2%; 2611/5593 patients).[11] The initial counterintuitive finding of COVID-19 being associated with a lower risk of ventilator-associated pneumonia prompted post hoc exploratory analyses, which suggested that the apparent association was driven by incomplete adjustment for baseline pneumonia – likely more common among patients with COVID-19. Another possible explanation may be difficulty ascertaining a new episode of ventilator-associated pneumonia in patients with COVID-19, which could have led to missed diagnoses and confounding that attenuated once baseline pneumonia was accounted for (or excluded), resulting in no independent association.

Limitations of this study include the lack of information about SARS-CoV-2 variants and SARS-CoV-2 vaccine timing. We also cannot exclude the possibility of selection bias in some centers given a lack of information about patients were eligible for REVISE but who were not enrolled. Our definition of COVID-19 is limited to patients who received COVID-19 specific treatment; others may have been symptomatic. On the other hand, we assumed that those who received specific treatments for COVID-19 actually had COVID-19, whereas a patient could have received dexamethasone for upper respiratory tract edema after dental abscess drainage and tested positive for SARS-CoV-2, without the virus necessarily causing symptoms. The REVISE trial was not designed *a priori* to study COVID-19, as the pandemic was declared after the trial had launched. Furthermore, no pre-specified sample size calculation was possible to discern an effect modification based on SARS-CoV-2 status. The confidence intervals around the hazard ratios for some results are relatively wide, suggesting uncertainty about the power to detect an interaction effect if one existed. Finally, our propensity analysis could not adjust for all possible factors predisposing to SARS-CoV-2 leading to possible unrecognized confounding.

For peer review only - http://bmjopen.bmj.com/site/about/guidelines.xhtml

Strengths of this study include a large and prospectively collected trial dataset. This SARS-CoV-2 Cohort substudy was registered [NCT05715567], hypotheses were pre-specified for each objective and analysis was guided by a statistical analysis plan.[12] The point estimates and confidence intervals for the propensity-matching and multivariable regression analyses are similar, increasing our confidence in the inferences about these results. This substudy offers the first randomized trial data on the effect of PPIs among patients with SARS-CoV-2 on a range of clinical outcomes.

CONCLUSION

In this preplanned substudy, COVID-19 infection was associated with higher mortality and longer ICU and hospital stays without an increased risk of clinically important upper GI bleeding in critically ill mechanically ventilated patients compared to patients who were not tested positive for SARS-CoV-2. Pantoprazole demonstrated similar effects on bleeding and safety outcomes regardless of COVID-19 status. Despite observational reports suggesting potential harm from acid suppression in patients with COVID-19, pantoprazole reduced clinically important and patient important upper GI bleeding without affecting other outcomes, offering consistent benefit regardless of COVID-19 status, supporting its consideration for stress ulcer prophylaxis.

For peer review only

Table 1: Characterization of REVISE Patients with tested positive for SARS-CoV-2 During Hospital Stay

Tested positive SARS-CoV-2 positive any time during hospital stay AND part of the SARS-CoV-2 substudy*	Pantoprazole n=268	Placebo n=264	Total n=532
Vaccination status pre-ICU			
Not vaccinated	111 (41.4)	115 (43.6)	226 (42.5)
Unknown	52 (19.4)	60 (22.7)	112 (21.1)
1 dose	21 (7.8)	12 (4.5)	33 (6.2)
2 doses	46 (17.2)	26 (9.8)	72 (13.5)
3 doses	38 (14.2)	51 (19.3)	89 (16.7)
Computed tomography positive for PE, number (%)			
Yes	28 (10.4)	34 (12.9)	62 (11.7)
No	94 (35.1)	115 (43.6)	209 (39.3)
Test not done	146 (54.5)	115 (43.6)	261 (49.1)
Ultrasound positive for one or more DVT, number (%)			
Yes	24 (9.0)	25 (9.5)	49 (9.2)
No	61 (22.8)	55 (20.8)	116 (21.8)
Test not done	183 (68.3)	184 (69.7)	367 (69.0)
Positive for bowel ischemia, number (%)			
Yes	3 (1.1)	5 (1.9)	8 (1.5)
No	53 (19.8)	58 (22.0)	111 (20.9)
Test not done	212 (79.1)	201 (76.1)	413 (77.6)
D Dimer level, mg/L (highest), median (IQR)	2460 (1020-5000) n=142	3485 (1250-6240) n=154	2855.5 (1180-5000) n=296
C-reactive protein level, mg/dL (highest), median (IQR)	148.0 (72.3-243.0) n=185	163.5 (89.8-252.0) n=199	156.1 (78.2-243.5) n=384
Ferritin level, ng/mL (highest), median (IQR)	905 (440-1717) n=113	866.5 (370-1935) n=124	883 (403-1870) n=237
Possible SARS-CoV-2-related treatments			
ACE2 Renin-angiotensin Inhibitor	30 (11.2)	20 (7.6)	50 (9.4)
Azithromycin	114 (42.5)	117 (44.3)	231 (43.4)
Baricitinib (Janus-associated kinase (JAK) inhibitor)	23 (8.6)	18 (6.8)	41 (7.7)
Casirivimab	2 (0.7)	4 (1.5)	6 (1.1)
Casirivimab and imdevimab (*casirivimab* / imdevimab monoclonal antibody)	2 (0.7)	0 (0.0)	2 (0.4)
Convalescent plasma	0 (0.0)	0 (0.0)	0 (0.0)
Dexamethasone	201 (75.0)	199 (75.4)	400 (75.2)
Other high dose steroid	32 (11.9)	46 (17.4)	78 (14.7)
Nirmatrelvir/ritonavir *(Paxlovid)*	3 (1.1)	2 (0.8)	5 (0.9)
Molnupiravir	2 (0.7)	1 (0.4)	3 (0.6)
Oseltamivir	3 (1.1)	6 (2.3)	9 (1.7)
Remdesivir	57 (21.3)	60 (22.7)	117 (22.0)
Ruxolitinib	0 (0.0)	1 (0.4)	1 (0.2)
Sarilumab	5 (1.9)	3 (1.1)	8 (1.5)
Sotrovimab (monoclonal antibody)	0 (0.0)	1 (0.4)	1 (0.2)

For peer review only

Statin	51 (19.0)	46 (17.4)	97 (18.2)
Tixagevimab/cilgavimab	0 (0.0)	1 (0.4)	1 (0.2)
Tocilizumab	77 (28.7)	76 (28.8)	153 (28.8)
Intravenous vitamin C	20 (7.5)	21 (8.0)	41 (7.7)
Other†	16 (6.0)	17 (6.4)	33 (6.2)
Extra-corporeal membrane oxygenation	2 (0.7)	7 (2.7)	9 (1.7)
Any of the above	237 (88.4)	230 (87.1)	467 (87.8)
None of the above	31 (11.6)	34 (12.9)	65 (12.2)
Tracheostomy, number (%)	37 (13.8)	47 (17.8)	84 (15.8)
Days from randomization to tracheostomy, in those with a tracheostomy	n=37	n=47	n=84
Tracheostomy prior to day of randomization	4 (10.8)	3 (6.4)	7 (8.3)
0-6 days	2 (5.4)	2 (4.3)	4 (4.8)
7-13 days	6 (16.2)	10 (21.3)	16 (19.0)
14-20 days	10 (27.0)	15 (31.9)	25 (29.8)
21-27 days	11 (29.7)	7 (14.9)	18 (21.4)
28-34 days	2 (5.4)	6 (12.8)	8 (9.5)
35 or more days	2 (5.4)	4 (8.5)	6 (7.1)
Days from randomization to tracheostomy in those with no tracheostomy prior to day of randomization	n=33	n=44	n=77
mean (SD)	19.0 (8.9)	21.1 (16.2)	20.2 (13.5)
median (IQR)	19 (14-22)	17.5 (12.5-24)	18 (13-23)
total range	0-47	0-105	0-105

Legend for Table 1: In this table, we describe the 532 patients with confirmed SARS-CoV-2 at any time during hospital stay who are part of the comparative analyses for this substudy. However, there are 8 SARS-CoV-2 positive patients from 1 center which did not participate in the SARS-CoV-2 substudy.

† Other treatments include: hydroxychloroquine, ivermectin, colchicine, ASA, vitamin D, acyclovir, valacyclovir, zinc, rivaroxaban.

SARS-CoV-2= severe acute respiratory syndrome coronavirus-2
ICU=intensive care unit
PE=pulmonary embolism
DVT=deep vein thrombosis
ACE2=angiotensin converting enzyme-2
IQR=interquartile range
SD=standard deviation

For peer review only

Table 2: Clinical Characteristics of Patients by COVID-19 Status

	Patients not tested positive for SARS-CoV-2 n=4010	COVID-19 Patients n=467	Total n=4477
Age in years, mean (SD)	57.9 (16.5)	61.4 (14.0)	58.2 (16.3)
APACHE II score, mean (SD)	21.8 (8.3)	20.9 (8.0)	21.7 (8.3)
Sex, number (%)			
Female	1439 (35.9)	187 (40.0)	1626 (36.3)
Male	2571 (64.1)	280 (60.0)	2851 (63.7)
Patient status, number (%)			
Medical	2819 (70.3)	440 (94.2)	3259 (72.8)
Surgical	564 (14.1)	15 (3.2)	579 (12.9)
Trauma	627 (15.6)	12 (2.6)	639 (14.3)
Admitting diagnostic category, number (%)			
Cardiovascular	441 (11.0)	23 (4.9)	464 (10.4)
Respiratory	1037 (25.9)	365 (78.2)	1402 (31.3)
Gastrointestinal	191 (4.8)	6 (1.3)	197 (4.4)
Neurologic	979 (24.4)	31 (6.6)	1010 (22.6)
Sepsis	338 (8.4)	14 (3.0)	352 (7.9)
Trauma	627 (15.6)	12 (2.6)	639 (14.3)
Metabolic	169 (4.2)	8 (1.7)	177 (4.0)
Renal	59 (1.5)	2 (0.4)	61 (1.4)
Other Medical	61 (1.5)	4 (0.9)	65 (1.5)
Other Surgical	108 (2.7)	2 (0.4)	110 (2.5)
Pre-hospital acid suppression, number (%)			
Yes	920 (22.9)	104 (22.3)	1024 (22.9)
No	3090 (77.1)	363 (77.7)	3453 (77.1)
Corticosteroids in the week pre-randomization, number (%)	1206 (30.1)	362 (77.5)	1568 (35.0)
**Study Day 1**, number (%)			
Invasive mechanical ventilation	4010 (100.0)	467 (100.0)	4477 (100.0)
Inotrope or vasopressor infusion	2825 (70.4)	343 (73.4)	3168 (70.8)
Renal replacement therapy	272 (6.8)	17 (3.6)	289 (6.5)

Legend for Table 2: This table summarizes the clinical characteristics and information on the critical admission for patients from the REVISE trial with data presented disaggregated by SARS-CoV-2 status.

SD=standard deviation
APACHE II score= Acute Physiology and Chronic Health Evaluation II Score
SARS-CoV-2= severe acute respiratory syndrome coronavirus-2

For peer review only

Table 3. Associations between SARS-CoV-2 status and Clinical Outcomes

Outcomes	Confounders adjustment using propensity-matching (2:1) (n=1401) Adjusted Hazard Ratio (95% CI) SARS-CoV-2 (n=467) vs. non-SARS-CoV-2 (n=934)	p-value	Confounders adjustment using multivariable regressions[1] (n=4477) Adjusted Hazard Ratio (95% CI) SARS-CoV-2 (n=467) vs. non-SARS-CoV-2 (n=4010)	p-value
**Time to event outcome: no violation of Proportional Hazards assumption by SARS-CoV-2 status**				
Clinically important upper GI bleed[1]	0.78 (0.40, 1.50)	0.45	0.74 (0.41, 1.34)	0.32
Incident *C. difficile*[1]	0.67 (0.21, 2.10)	0.49	0.75 (0.25, 2.23)	0.61
New renal replacement therapy[1]	1.24 (0.85, 1.81)	0.26	1.25 (0.88, 1.77)	0.21
Patient important upper GI bleed[1]	0.77 (0.43, 1.38)	0.38	0.76 (0.45, 1.31)	0.32
Ventilator-associated pneumonia[1]	0.50 (0.37, 0.69)	< 0.001	0.47 (0.35, 0.63)	< 0.001
**Time to event outcome: violation of Proportional Hazards assumption by SARS-CoV-2 status**				
ICU mortality[2] at				
30 days	1.73 (1.06, 2.40)	< 0.001	1.47 (1.02, 1.91)	< 0.001
60 days	2.50 (1.13, 3.87)	< 0.001	2.07 (1.17, 2.98)	< 0.001
90 days	3.10 (1.10, 5.10)	0.002	2.54 (1.21, 3.87)	< 0.001
Hospital mortality[2] at				
30 days	1.62 (1.17, 2.07)	< 0.001	1.49 (1.14, 1.84)	< 0.001
60 days	2.18 (1.33, 3.03)	< 0.001	2.03 (1.35, 2.70)	< 0.001
90 days	2.59 (1.39, 3.79)	< 0.001	2.43 (1.46, 3.39)	< 0.001
Mortality within 90 days[2]				
30 days	1.91 (1.45, 2.37)	< 0.001	1.78 (1.41, 2.16)	< 0.001
60 days	2.65 (1.76, 3.55)	< 0.001	2.56 (1.79, 3.32)	< 0.001
90 days	3.22 (1.93, 4.50)	< 0.001	3.16 (2.01, 4.30)	< 0.001
**Linear regression models studying associations between length of stay outcomes and SARS-CoV-2 status**				
	Adjusted Ratio of Duration (95% CI)[¥]	p-value	Adjusted Ratio of Duration (95% CI)[¥]	p-value
Outcomes				
**Duration of ICU stay in days**[¶, 1]				
Exposure of interest: SARS-CoV-2 vs. non-SARS-CoV-2	1.45 (1.33, 1.58)	< 0.001	1.45 (1.35, 1.57)	< 0.001

**Duration of hospital stay in days¶, 1**				
Exposure of interest: SARS-CoV-2 vs. non-SARS-CoV-2	1.30 (1.18, 1.43)	< 0.001	1.35 (1.24, 1.48)	< 0.001
**Duration of mechanical ventilation in days¶, 1**				
Exposure of interest: SARS-CoV-2 vs. non-SARS-CoV-2	1.69 (1.53, 1.87)	< 0.001	1.66 (1.52, 1.81)	< 0.001

Legend for Table 3: In this table we show the effect of SARS-CoV-2 status on clinical outcomes among mechanically ventilated patients admitted to the ICU. Cox proportional hazard regression models and linear regression models were used to study associations between SARS-CoV-2 status and clinical outcomes using two methods of confounders adjustment: 1) Propensity-matching (2:1) and 2) multivariable regression.

[1]Adjusted for patients' age, sex, pre-hospital acid suppression, ICU admission category (medical or surgical/trauma), corticosteroids in the week pre randomization, days from start of pandemic to enrollment, and randomization group (Pantoprazole vs placebo)
[2]Adjusted for variables listed in 1, and APACHE II score quartiles
¶Natural logarithm of length of stay was the dependent variable in the linear regressions
¥Exponentiation of the adjusted regression coefficient and its' confidence intervals are presented

SARS-CoV-2= severe acute respiratory syndrome coronavirus-2
GI=gastrointestinal
C. difficile=Clostridioides difficile
ICU=intensive care unit
CI=confidence interval

For peer review only

Table 4: Efficacy of Pantoprazole in Patients with SARS-CoV-2 in REVISE: Subgroup Analyses

	Pantoprazole n (%)	Placebo n (%)	Hazard Ratio (95% CI)	P-value for the interaction term
**Primary Efficacy Outcome (Clinically Important Upper GI Bleeding)**				
Non-SARS-CoV-2	20/1983 (1.0)	67/1982 (3.4)	**0.30 (0.18, 0.49)**	0.868
SARS-CoV-2	3/235 (1.3)	12/226 (5.3)	**0.27 (0.08, 0.95)**	
**Primary Safety Outcome (90-day Mortality)**				
Non-SARS-CoV-2	567/1987 (28.5)	583/1986 (29.4)	0.97 (0.86, 1.09)†	0.855†
SARS-CoV-2	82/235 (34.9)	88/224 (39.3)	0.94 (0.69, 1.27)†	
**Incident Ventilator-associated pneumonia (Clinical Pulmonary Infection Score ≥6) in ICU**				
Non-SARS-CoV-2	487/1990 (24.5)	495/1986 (24.9)	1.00 (0.88, 1.13)	0.881
SARS-CoV-2	26/235 (11.1)	26/226 (11.5)	1.04 (0.61, 1.80)	
**Incident *C. difficile* infection in hospital**				
Non-SARS-CoV-2	21/1983 (1.1)	16/1982 (0.8)	1.33 (0.69, 2.55)	0.985
SARS-CoV-2	4/235 (1.7)	0/226 (0.0)	-	
**New renal replacement therapy in ICU**				
Non-SARS-CoV-2	110/1983 (5.5)	110/1985 (5.5)	1.01 (0.77, 1.31)	0.308
SARS-CoV-2	26/235 (11.1)	20/226 (8.8)	1.41 (0.78, 2.52)	
**ICU mortality**				
Non-SARS-CoV-2	392/1998 (19.6)	401/1997 (20.1)	1.00 (0.87, 1.15)	0.944
SARS-CoV-2	60/236 (25.4)	67/227 (29.5)	1.01 (0.71, 1.44)	
**Hospital mortality**				
Non-SARS-CoV-2	514/1996 (25.8)	536/1988 (27.0)	0.99 (0.87, 1.11)	0.663
SARS-CoV-2	71/236 (30.1)	80/227 (35.2)	0.92 (0.66, 1.26)	
**Incident Patient important Upper GI Bleeding**				
Non-SARS-CoV-2	28/1983 (1.4)	81/1982 (4.1)	0.34 (0.22, 0.53)	0.763
SARS-CoV-2	5/235 (2.1)	13/226 (5.8)	0.41 (0.15, 1.15)	
	**Pantoprazole median (IQR)**	**Placebo median (IQR)**	**Ratio of Duration (95% CI)**	**P-value for the interaction term**
**Duration of mechanical ventilation in days**				
Non-SARS-CoV-2	6 (3-11)	6 (3-11)	0.97 (0.92, 1.03)	0.170
SARS-CoV-2	9 (5-16)	10 (6-19)	0.87 (0.74, 1.01)	
**Duration of ICU stay in days**				

For peer review only - http://bmjopen.bmj.com/site/about/guidelines.xhtml

Non-SARS-CoV-2	9 (6-15)	9 (6-16)	0.98 (0.93, 1.02)	0.129
SARS-CoV-2	13 (7-21)	14 (9-23)	0.87 (0.76, 1.00)	
**Duration of hospital stay in days**				
Non-SARS-CoV-2	20 (11-35)	21 (11-37)	0.96 (0.91, 1.02)	0.690
SARS-CoV-2	22 (15-40)	25 (14-43)	0.93 (0.79, 1.09)	

Legend for Table 4: In this table we show the effect of pantoprazole versus placebo on clinical outcomes among patients with and without SARS-CoV-2.
† For 90-day mortality, the SARS-CoV-2 subgroup variable violates the assumption of proportional hazards. Therefore, the first Cox proportional hazards model includes only "Non-SARS-CoV-2" patients, and a separate Cox proportional hazards model including only "SARS-CoV-2" patients. The two hazard ratios are compared by performing a z-test on the log(hazard ratio). We did not apply criteria to assess subgroup credibility because all p-values were above 0.10, according to our statistical analysis plan.

SARS-CoV-2= severe acute respiratory syndrome coronavirus-2
GI=gastrointestinal
C. difficile =*Clostridioides difficile*
ICU=intensive care unit

For peer review only

Acknowledgements: The trial was designed by the REVISE Steering Committee including National and International Management Committees, the REVISE Investigators and Research Coordinators, the Canadian Critical Care Trials Group and the Australian and New Zealand Intensive Care Society Clinical Trials Group. We thank the patients and families participating in this trial, and all the research personnel and bedside clinicians who supported this work. We are grateful to others at the Methods Center at McMaster University (Lisa Buckingham, France Clarke, Mary Copland, Karlo Matic, Ashley Sawyer, Alyson Takaoka) and The George Institute (Fatima Butt, Anna Cheng, Conrad Nangla, Fiona Osborne, Tina Schneider, Isabella Schoeler, Anna Tippet) for their expertise.

Competing interests: All authors are involved in the REVISE Trial in some capacity except Aijaz Ahmed. Otherwise, all authors declare that they have no competing interests.

Funding Statement: REVISE was funded by peer-reviewed grants [Canadian Institutes of Health Research (201610PJT-378226-PJT-CEBA-18373 and 202207CL3-492565-CTP-CEBA-19215)], and the Hamilton Academy of Health Sciences Organization (HAH-22-009). National Health and Medical Research Council of Australia grant [GNT1124675] funded enrolment in Australia. REVISE was approved by the National Institute for Health Research (NIHR) in the UK as a Portfolio Study [CPMS ID 45782], eligible for support from the NIHR Clinical Research Network. [https://www.nihr.ac.uk/researchers/collaborations-services-and-support-for-your-research/run-your-study/crn-portfolio.htm]. This SARS-CoV-2 Substudy was funded by a peer-review grant from Physicians Services Incorporated of Ontario (R22-33) and non-grant funds from St. Joseph's Healthcare Hamilton and McMaster University. This trial received no support from the commercial or private sector. The funders/sponsors have no role in the conception, design, conduct, oversight, analysis, interpretation, write-up, review or approval of the manuscript, or decision to submit the manuscript for publication.

Role of the Funders: The funders and sponsors have no role in the conception, design, conduct, oversight, analysis, interpretation, write-up, review or approval of the manuscript, or decision to submit the manuscript for publication.

Ethics and Regulatory Approval: The main REVISE trial was approved in participating hospitals by all relevant ethics committees and regulatory authorities prior to starting enrolment. The Hamilton Integrated Research Ethics Board (HiREB) under Clinical Trials Ontario (CTO) Study ID 1360 is the main REB of record, which also approved this substudy. Ethics review for the additional SARS-CoV-2-specific data collection was undertaken as needed to comply with local regulations in each participating center.

Authors' contributions:

Concept and design: Y Arabi, A Binnie, D Cook, A Deane, B Dennis, J Dionne, S English, G Guyatt, M Hardie, D Heels-Ansdell, S Knowles, F Lauzier, J Marshall, J Muscedere, J Myburgh, M Ostermann, Saunders, L Thabane, J Tsang, N Zytaruk

Guarantor: D Cook

Acquisition, analysis, or interpretation of data: J Basmaji, D Cook, B Dennis, L Hand, M Hardie, D Heels-Ansdell, S Knowles, O Loy, L Saunders, L Thabane, N Zytaruk

Drafting of the manuscript: D Cook, B Dennis, D Heels-Ansdell, Q Ibrahim

Statistical analysis: J Basmaji, G Guyatt, D Heels-Ansdell, Q Ibrahim, L Thabane

For peer review only

Obtained funding for this substudy: A Ahmed, A Binnie, D Cook, B Dennis, J Dionne, G Guyatt, D Heels-Ansdell, L Thabane, J Tsang

Administrative, technical, or material support: J Basmaji, D Cook, A Deane, B Dennis, G Guyatt, M Hardie, D Heels-Ansdell, Q Ibrahim, S Knowles, F Lauzier, O Loy, L Saunders, A Poole, L Thabane, N Zytaruk

Data Integrity: J Basmaji, D Cook, B Dennis, M Hardie, D Heels-Ansdell, Q Ibrahim, S Knowles, N Zytaruk

Approved final manuscript: All authors

Data Sharing Statement: The REVISE dataset will be used for secondary observational studies such as this one, addressing additional hypothesis-driven questions. The study protocol and statistical analysis plan for this substudy are published. Access by REVISE investigators to the REVISE dataset will follow a submitted rationale, analysis plan and approval by the Management Committee. Requests for access to the data for this substudy and the parent REVISE trial for investigator-initiated studies can be sent by external academic investigators. These will be considered following a submitted rationale, methods and analysis plan, then approval by the REVISE Management Committee and research ethics boards as relevant. Requirements will be stipulated in a pre-specified data sharing agreement. If approved, only de-identified trial data, data dictionaries and standard operating procedures will be provided to achieve aims of the proposal. Data will be transferred via a secure web portal. Data are available until 2035.

For peer review only

REFERENCES

1. Xiang Y, Wong KC, So HC: **Exploring Drugs and Vaccines Associated with Altered Risks and Severity of COVID-19: A UK Biobank Cohort Study of All ATC Level-4 Drug Categories Reveals Repositioning Opportunities**. *Pharmaceutics* 2021, **13**(9).
2. Lee SW, Ha EK, Yeniova AÖ, Moon SY, Kim SY, Koh HY, Yang JM, Jeong SJ, Moon SJ, Cho JY *et al*: **Severe clinical outcomes of COVID-19 associated with proton pump inhibitors: a nationwide cohort study with propensity score matching**. *Gut* 2021, **70**(1):76-84.
3. Aby ES, Tappata M, Rodin H, Debes JD: **Assessment of proton-pump inhibitor use, race, socioeconomic status, and mortality in individuals with COVID-19**. *European Journal of Gastroenterology & Hepatology* 2022, **34**(2):239.
4. Hariyanto TI, Prasetya IB, Kurniawan A: **Proton pump inhibitor use is associated with increased risk of severity and mortality from coronavirus disease 2019 (COVID-19) infection**. *Dig Liver Dis* 2020, **52**(12):1410-1412.
5. Pranata R, Huang I, Lawrensia S, Henrina J, Lim MA, Lukito AA, Kuswardhani RAT, Wibawa IDN: **Proton pump inhibitor on susceptibility to COVID-19 and its severity: a systematic review and meta-analysis**. *Pharmacol Rep* 2021, **73**(6):1642-1649.
6. Krag M, Perner A, Wetterslev J, Wise MP, Borthwick M, Bendel S, McArthur C, Cook D, Nielsen N, Pelosi P *et al*: **Prevalence and outcome of gastrointestinal bleeding and use of acid suppressants in acutely ill adult intensive care patients**. *Intensive Care Med* 2015, **41**(5):833-845.
7. World Health O: **Clinical management of severe acute respiratory infection (SARI) when COVID-19 disease is suspected: interim guidance, 13 March 2020**. In. Geneva: World Health Organization; 2020.
8. Alhazzani W, Evans L, Alshamsi F, Møller MH, Ostermann M, Prescott HC, Arabi YM, Loeb M, Ng Gong M, Fan E *et al*: **Surviving Sepsis Campaign Guidelines on the Management of Adults With Coronavirus Disease 2019 (COVID-19) in the ICU: First Update**. *Crit Care Med* 2021, **49**(3):e219-e234.
9. The REMAP-CAP, ACTIV-4a, and ATTACC Investigators. **Therapeutic Anticoagulation with Heparin in Critically Ill Patients with Covid-19**. *New England Journal of Medicine* 2021, **385**(9):777-789.
10. **Dexamethasone in Hospitalized Patients with Covid-19**. *New England Journal of Medicine* 2020, **384**(8):693-704.
11. Ippolito M, Misseri G, Catalisano G, Marino C, Ingoglia G, Alessi M, Consiglio E, Gregoretti C, Giarratano A, Cortegiani A: **Ventilator-Associated Pneumonia in Patients with COVID-19: A Systematic Review and Meta-Analysis**. *Antibiotics (Basel)* 2021, **10**(5).
12. Dennis BB, Thabane L, Heels-Ansdell D, Dionne JC, Binnie A, Tsang J, Guyatt G, Ahmed A, Lauzier F, Deane A *et al*: **Proton pump inhibitors in critically ill mechanically ventilated patients with COVID-19: protocol for a substudy of the Re-EValuating the Inhibition of Stress Erosions (REVISE) Trial**. *Trials* 2023, **24**(1):561.
13. Cook D, Deane A, Lauzier F, Zytaruk N, Guyatt G, Saunders L, Hardie M, Heels-Ansdell D, Alhazzani W, Marshall J *et al*: **Stress Ulcer Prophylaxis during Invasive Mechanical Ventilation**. *N Engl J Med* 2024, **391**(1):9-20.
14. **Re-EValuating the Inhibition of Stress Erosions (REVISE) Trial (REVISE)**. In. ClinicalTrials.Gov; 2018.
15. Heels-Ansdell D, Billot L, Thabane L, Alhazzani W, Deane A, Guyatt G, Finfer S, Lauzier F, Myburgh J, Young P *et al*: **REVISE: re-evaluating the inhibition of stress erosions in the ICU-statistical analysis plan for a randomized trial**. *Trials* 2023, **24**(1):796.

For peer review only - http://bmjopen.bmj.com/site/about/guidelines.xhtml

16. Dennis B, Deane A, Lauzier F, Zytaruk N, Hardie M, Hammond N, Finfer S, Arabi Y, Marshall J, Saunders L *et al*: **Protocol implementation during the COVID-19 pandemic: experiences from a randomized trial of stress ulcer prophylaxis**. *BMC Med Res Methodol* 2024, **24**(1):109.
17. Cook DJ, Kho ME, Duan EH, Alhazzani W, Takaoka A, Clarke FJ, Zytaruk N, Vanstone M: **Principles Guiding Nonpandemic Critical Care Research During a Pandemic**. *Crit Care Med* 2020, **48**(10):1403-1410.
18. Schandelmaier S, Briel M, Varadhan R, Schmid CH, Devasenapathy N, Hayward RA, Gagnier J, Borenstein M, van der Heijden GJMG, Dahabreh IJ *et al*: **Development of the Instrument to assess the Credibility of Effect Modification Analyses (ICEMAN) in randomized controlled trials and meta-analyses**. *Canadian Medical Association Journal* 2020, **192**(32):E901-E906.
19. **SAS 9.4**. In. Edited by Inc SI. Cary, NC.
20. Maes M, Higginson E, Pereira-Dias J, Curran MD, Parmar S, Khokhar F, Cuchet-Lourenço D, Lux J, Sharma-Hajela S, Ravenhill B *et al*: **Ventilator-associated pneumonia in critically ill patients with COVID-19**. *Crit Care* 2021, **25**(1):25.
21. Giacobbe DR, Battaglini D, Enrile EM, Dentone C, Vena A, Robba C, Ball L, Bartoletti M, Coloretti I, Di Bella S *et al*: **Incidence and Prognosis of Ventilator-Associated Pneumonia in Critically Ill Patients with COVID-19: A Multicenter Study**. *J Clin Med* 2021, **10**(4).
22. Blonz G, Kouatchet A, Chudeau N, Pontis E, Lorber J, Lemeur A, Planche L, Lascarrou JB, Colin G: **Epidemiology and microbiology of ventilator-associated pneumonia in COVID-19 patients: a multicenter retrospective study in 188 patients in an un-inundated French region**. *Crit Care* 2021, **25**(1):72.

For peer review only

For peer review only - http://bmjopen.bmj.com/site/about/guidelines.xhtml

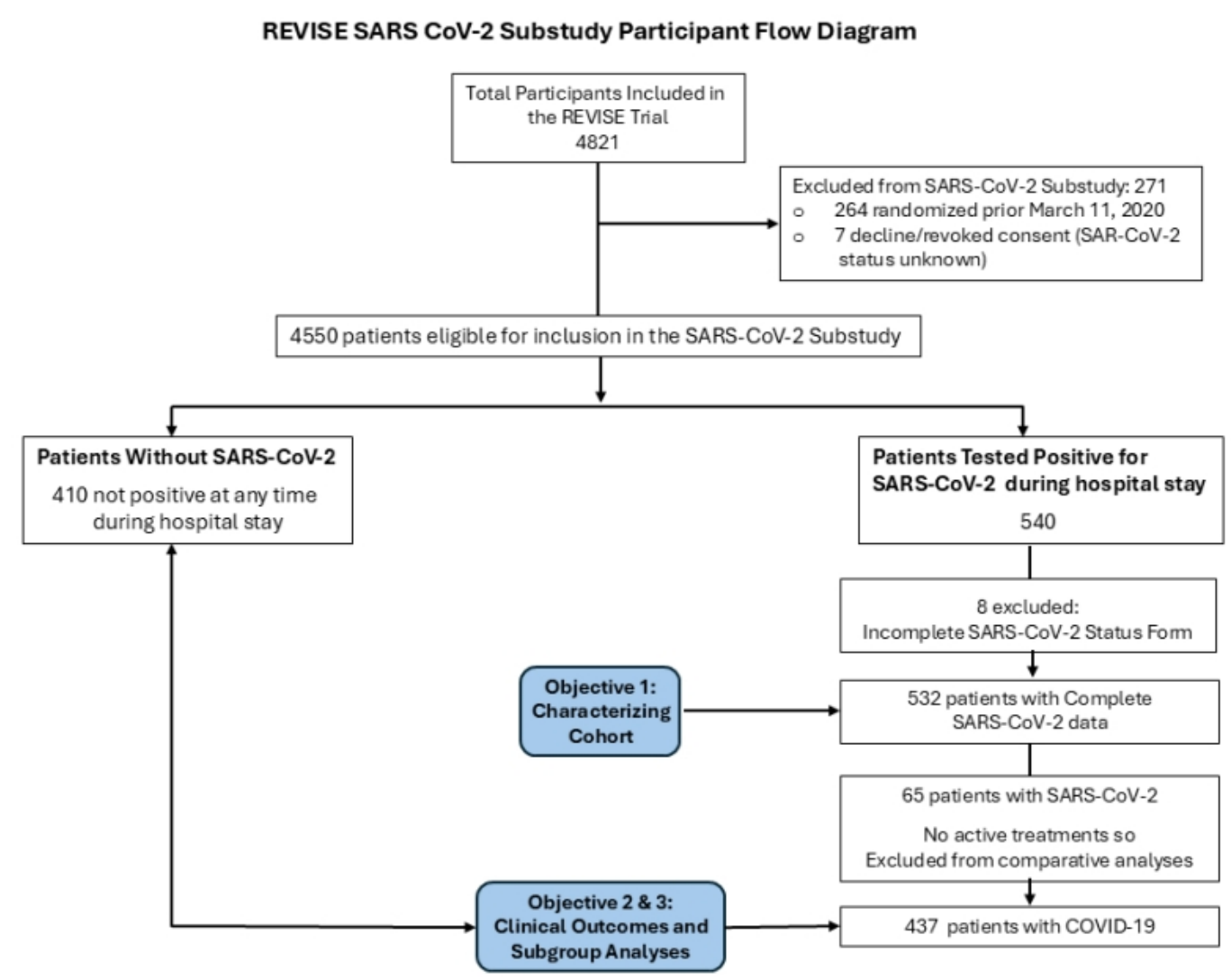

This figure summarizes participant inclusion into the SARS-CoV-2 Substudy, highlighting differences in sample size for each of the analyses addressing the 3 study objectives.

338x190mm (96 x 96 DPI)

Supplementary Tables:

Supplementary Table S1: Propensity Model Regressing SARS-CoV-2 Status on confounders using multivariable logistic regression

	Adjusted Odds Ratio (95% CI)
**Confounders**	
Age, 10-year increment	1.10 (1.03, 1.19)
Female	1.06 (0.86, 1.30)
Prehospital acid suppression	0.69 (0.54, 0.89)
ICU admission category: medical vs. surgical/trauma	4.34 (2.90, 6.51)
Corticosteroids in the week pre randomization	6.71 (5.33, 8.47)
Days from start of pandemic to enrollment, 1-year increment	0.73 (0.65, 0.81)

Legend Table S1: This stable shows a summary of results of a multivariable logistic regression model (*n*=4477) regressing SARS-CoV-2 status on the *a priori*-selected confounders being used in propensity-matching. SARS-COV-yes (n=467) or SARS-CoV-2-no (n=4010). The model demonstrates significant association between *a priori* selected pre-randomization confounder variables (i.e., age, pre-hospital acid suppression) and SARS-CoV-2 status.

ICU=intensive care unit

For peer review only

Supplementary Table S2: Patient Characteristics and Propensity Scores According to SARS-CoV-2 Status in the Propensity-Matched Cohort

	SARS-CoV-2 Patients	Non- SARS-CoV-2 Patients	Propensity-matched Non- SARS-CoV-2 Patients (2:1)
Number of patients, n (%)	467	4010	934
Age (years), mean ± SD	61 ± 14	58 ± 17	62 ± 13
Female, n (%)	187 (40.0)	1439 (35.9)	365 (39.1)
Prehospital acid suppression, n (%)	104 (22.3)	920 (22.9)	217 (23.2)
ICU admission category, n (%)			
Medical	440 (94.2)	2819 (70.3)	878 (94.0)
Surgical/trauma	27 (5.8)	1191 (29.7)	56 (6.0)
Corticosteroids in the week pre randomization, n (%)	362 (77.5)	1206 (30.1)	726 (77.7)
Days from start of pandemic to enrollment, mean ± SD	669 ± 280	787 ± 344	692 ± 339
Propensity Score			
mean ± SD	0.221 ± 0.110	0.091 ± 0.100	0.219 ± 0.110
median (IQR)	0.257 (0.145-0.305)	0.048 (0.024-0.112)	0.245 (0.146-0.295)
minimum	0.006	0.004	0.006
maximum	0.411	0.439	0.411

Legend Supplementary Table S2: This table shows the baseline characteristics of patients with SARS-CoV-2 and propensity-matched patients without SARS-CoV-2. This table also shows the propensity scores according to SARS-CoV-2 Status in the propensity-matched cohort.
SARS-CoV-2=severe acute respiratory syndrome coronavirus-2
SD=standard deviation
ICU=intensive care unit
IQR=interquartile range

For peer review only

Supplementary Table S3. Association between SARS-CoV-2 Status and Ventilator-associated Pneumonia

	Confounders adjustment using propensity matching (2:1) (n=1401)		Confounders adjustment using multivariable regressions (n=4477)	
Exploratory Model 1	Adjusted Hazard Ratio (95% CI) SARS-CoV-2 (n=467) vs. non- SARS-CoV-2 (n=934)	p-value	Adjusted Hazard Ratio (95% CI) SARS-CoV-2 (n=467) vs. non- SARS-CoV-2 (n=4010)	p-value
Ventilator-associated Pneumonia[1]	1.08 (0.78, 1.50)	0.63	1.00 (0.75, 1.34)	0.98
Exploratory Model 2	Adjusted Hazard Ratio (95% CI) SARS-CoV-2 (n=157) vs. non- SARS-CoV-2 (n=314)	p-value	Adjusted Hazard Ratio (95% CI) SARS-CoV-2 (n=157) vs. non- SARS-CoV-2 (n=3431)	p-value
Ventilator-associated Pneumonia[2]	0.98 (0.72, 1.35)	0.92	1.09 (0.82, 1.46)	0.55

Legend Supplementary Table S3: This table shows results from 2 exploratory analyses evaluating the association between SARS-CoV-2 status and ventilator-associated pneumonia, using different adjustment methods to account for pneumonia diagnoses on admission to the ICU. The 2 methods of confounder adjustment were: 1) Propensity-matching (2:1) and 2) Cox proportional hazards multivariable regression.

[1]Multivariable regression: adjusted for patients' age, sex, pre-hospital acid suppression, ICU admission category (medical or surgical/trauma), corticosteroids in the week pre randomization, days from start of pandemic to enrollment, randomization group (Pantoprazole vs placebo), and baseline pneumonia. Original Propensity-matched data: adjusted for randomization group (Pantoprazole vs placebo), and baseline pneumonia

[2]Patients with pneumonia at baseline were removed and original propensity scores were re-estimated on the reduced data. Exploratory models 1 were refitted in the reduced data.

SARS-CoV-2=severe acute respiratory syndrome coronavirus-2

For peer review only - http://bmjopen.bmj.com/site/about/guidelines.xhtml

Supplementary Table S4. Associations between SARS-CoV-2 Status and Clinical Outcomes, accounting for the competing risk of death

	Confounders adjustment using propensity matching (2:1) (n=1401)		Confounders adjustment using multivariable regressions (n=4477)	
	Adj SHR (95% CI) SARS-CoV-2 (n=467) vs. non- SARS-CoV-2 (n=934)	p-value	Adj SHR (95% CI) SARS-CoV-2 (n=467) vs. non- SARS-CoV-2 (n=4010)	p-value
Clinically important upper GI bleed[1]	0.86 (0.45, 1.64)	0.65	0.82 (0.46, 1.48)	0.52
Incident *C. difficile*[1]	0.69 (0.22, 2.17)	0.53	0.79 (0.28, 2.27)	0.67
New renal replacement therapy[1]	1.30 (0.90, 1.90)	0.17	1.33 (0.95, 1.86)	0.09
Patient important upper GI bleed[1]	0.85 (0.48, 1.51)	0.58	0.85 (0.50, 1.43)	0.54
Ventilator-associated pneumonia[1]	0.54 (0.40, 0.74)	< 0.001	0.51 (0.38, 0.68)	< 0.001
Ventilator-associated pneumonia[2]	1.21 (0.87, 1.67)	0.26	1.11 (0.84, 1.46)	0.48

Legend Supplementary Table S4: This table shows results from exploratory analyses evaluating the association between SARS-CoV-2 status and clinical outcomes, accounting for the competing risk of death using Fine and Gray subdistribution hazard regression models.

[1]Adjusted for patients' age, sex, pre-hospital acid suppression, ICU admission category (medical or surgical/trauma), corticosteroids in the week pre randomization, days from start of pandemic to enrollment, and randomization group (Pantoprazole vs placebo)

[2]Adjusted for variables listed in 1 plus baseline pneumonia

For peer review only

Dennis *et al. Trials* (2023) 24:561
https://doi.org/10.1186/s13063-023-07589-2

Trials

STUDY PROTOCOL Open Access

Proton pump inhibitors in critically ill mechanically ventilated patients with COVID-19: protocol for a substudy of the Re-EValuating the Inhibition of Stress Erosions (REVISE) Trial

Brittany B. Dennis[1], Lehana Thabane[2,3,4], Diane Heels-Ansdell[2], Joanna C. Dionne[5], Alexandra Binnie[6], Jennifer Tsang[1,6], Gordon Guyatt[5], Aijaz Ahmed[7], François Lauzier[8], Adam Deane[9], Yaseen Arabi[10,11,12], John Marshall[13], Nicole Zytaruk[2,4], Lois Saunders[4], Simon Finfer[14], John Myburgh[14,15], John Muscedere[16], Shane English[17,18], Marlies Ostermann[19], Miranda Hardie[14], Serena Knowles[14], Deborah Cook[4,5*] and For the REVISE Investigators the Canadian Critical Care Trials Group

For peer review only

Abstract

Background Critically ill patients commonly receive proton pump inhibitors (PPIs) to prevent gastrointestinal (GI) bleeding from stress-induced ulceration. Despite widespread use in the intensive care unit (ICU), observational data suggest that PPIs may be associated with adverse outcomes in patients with COVID-19 infection. This pre-planned study is nested within a large randomized trial evaluating pantoprazole versus placebo in invasively ventilated patients. The 3 objectives are as follows: (1) to describe the characteristics of patients with COVID-19 in terms of demographics, biomarkers, venous thromboembolism, tracheostomy incidence and timing, and other clinical outcomes; (2) to evaluate the impact of COVID-19 infection on clinically important GI bleeding, 90-day mortality, and other outcomes compared to a propensity-matched non-infected cohort; and (3) to explore whether pantoprazole has a differential treatment effect on clinically important GI bleeding, 90-day mortality, and other outcomes in patients with and without COVID-19 infection.

Methods The ongoing trial Re-EValuating the Inhibition of Stress Erosions (REVISE) compares pantoprazole 40 mg IV to placebo on the primary efficacy outcome of clinically important GI bleeding and the primary safety outcome of 90-day mortality. The protocol described in this report is for a substudy focused on patients with COVID-19 infection that was not in the original pre-pandemic trial protocol. We developed a one-page case report form to characterize these patients including data related to biomarkers, venous thromboembolism, COVID-19 therapies, tracheostomy incidence and timing, duration of mechanical ventilation, and ICU and hospital stay. Our analysis will describe the trajectory of patients with COVID-19 infection, a propensity-matched analysis of infected and non-infected patients, and an extended subgroup analysis comparing the effect of PPI among patients with and without COVID-19 infection.

*Correspondence:
Deborah Cook
debcook@mcmaster.ca
Full list of author information is available at the end of the article

BMC

© The Author(s) 2023. **Open Access** This article is licensed under a Creative Commons Attribution 4.0 International License, which permits use, sharing, adaptation, distribution and reproduction in any medium or format, as long as you give appropriate credit to the original author(s) and the source, provide a link to the Creative Commons licence, and indicate if changes were made. The images or other third party material in this article are included in the article's Creative Commons licence, unless indicated otherwise in a credit line to the material. If material is not included in the article's Creative Commons licence and your intended use is not permitted by statutory regulation or exceeds the permitted use, you will need to obtain permission directly from the copyright holder. To view a copy of this licence, visit http://creativecommons.org/licenses/by/4.0/. The Creative Commons Public Domain Dedication waiver (http://creativecommons.org/publicdomain/zero/1.0/) applies to the data made available in this article, unless otherwise stated in a credit line to the data.

Discussion Prophylactic acid suppression in invasively ventilated critically ill patients with COVID-19 infection has unknown consequences. The results of these investigations will inform practice, guidelines, and future research.

Trial registration REVISE Trial [NCT03374800 December 15, 2017], COVID-19 Cohort Study [NCT05715567 February 8, 2023].

Keywords Randomization, Critically ill, COVID-19 pandemic, Hospital transfers, Stress ulceration, Gastrointestinal bleeding, Ventilator-associated pneumonia

Introduction

For patients with COVID-19 receiving invasive mechanical ventilation, standard critical care therapies, including prophylactic acid suppression, have unclear consequences. Concerns emerged early in the pandemic that some common medications may predispose to COVID-19 infection or worsen outcomes among infected patients. For example, observational studies suggested that angiotensin-converting enzyme (ACE) inhibitors were harmful as they were associated with increased mortality [1, 2]. However, this finding was not supported in larger, more robust observational studies [3, 4] or randomized trials [5].

The possible impact of proton pump inhibitors (PPIs) on COVID-19 infection susceptibility and outcomes has also drawn attention. In one observational study from the UK Biobank cohort, PPIs were associated with a 23% reduction in susceptibility to COVID-19 infection (odds ratio [OR] at 1 year = 0.77, 95% confidence interval [CI]: 0.71–0.83), with the greatest protective effects observed with recent use of PPIs (within 6 months) and in people at least 70 years of age [6]. However, these findings of benefit were in contrast with other studies suggesting associated harm. In a retrospective study of Korean national insurance claims including 132,316 patients, short-term use of PPIs was associated with a 79% increased risk of a composite outcome of admission to the ICU, use of mechanical ventilation, and death [7]. These findings were limited by low event rates, reliance on registry data to ascertain PPI exposure, and the use of a composite outcome measure due to insufficient events. In another retrospective case review of 2164 patients with COVID-19 admitted to a US county hospital, PPI use was associated with a 2.8-fold increased risk for death (OR) [95% CI, 1.4–5.5], although findings were not statistically significant following adjustment for age, renal, cardiovascular, and pulmonary comorbidities [8]. Furthermore, a pooled analysis of six retrospective studies of 5884 COVID-19 patients also suggested that PPI use may be associated with an increased risk of death from COVID-19 (risk ratio 1.7, 95% CI 1.02–2.9, $I^2 = 66\%$) [9]. However, all of these study designs generate limited inferences for practice or research purposes.

PPIs are common medications among hospitalized patients, particularly in the intensive care unit (ICU). Whether used pre-hospital or not, critically ill mechanically ventilated patients are usually prescribed PPIs to prevent gastrointestinal (GI) bleeding from stress-induced ulceration [10]. The potential adverse effects of PPI use include ventilator-associated pneumonia (VAP) and *Clostridioides difficile* (*C. difficile*) infection—nosocomial infections that are together more common than GI bleeding and are associated with substantially greater morbidity, mortality, and costs [11]. Herein, we describe a protocol to examine the association between COVID-19 infection and outcomes and PPI use among invasively mechanically ventilated patients nested within an ongoing clinical trial testing pantoprazole versus placebo [12]. This ongoing trial is relevant for patients with COVID-19 infection, given their increased risk of VAP [13], treatment with high-dose corticosteroids [14], and varying anticoagulation intensities [15].

The three objectives of this nested study are as follows: (1) to describe the characteristics of patients with COVID-19 in terms of demographics, biomarkers, COVID-19 treatment, rates of venous thromboembolism, tracheostomy incidence and timing, and other outcomes (e.g., duration of mechanical ventilation, ICU and hospital stay, and mortality); (2) to evaluate the impact of COVID-19 on clinically important GI bleeding, 90-day mortality, infectious, and other outcomes compared to a propensity-matched non-COVID-19 cohort; and (3) to explore whether pantoprazole has a differential treatment effect on clinically important GI bleeding and 90-day mortality, in addition to VAP and *C. difficile* infection and other outcomes, in patients with and without COVID-19.

Methods

Project design for overall REVISE Trial

The Re-EValuating the Inhibition of Stress Erosions (REVISE) is an international randomized stratified, concealed, blinded, parallel-group trial in mechanically ventilated patients, investigating the effect of the PPI pantoprazole 40 mg IV compared to placebo, on the primary *efficacy* outcome of clinically important GI bleeding, the primary *safety* outcome of 90-day mortality, rates of VAP and *C. difficile* infection, patient-important

For peer review only - http://bmjopen.bmj.com/site/about/guidelines.xhtml

bleeding, use of renal replacement therapy, and hospital mortality [12]. Led by the Canadian Critical Care Trials Group and the Australian & New Zealand Intensive Care Society Clinical Trials Group, the REVISE Methods Centers are located at McMaster University in Hamilton for international centers and The George Institute in Sydney for Australia. Research Coordinators screen patients from Monday to Friday, using an *a priori* or a consent-to-continue model as locally approved, holding consent encounters with patients if able, or substitute decision-makers in-person or by telephone as needed. Randomization is conducted by research pharmacists to ensure double blinding. Methods for the REVISE trial have been described elsewhere [NCT03374800] [12].

Design for nested COVID cohort study

This study uses a multicenter cohort design embedded within a randomized trial [NCT05715567]. This is the first version (version 1.0; date: June 13, 2023) of the substudy protocol. Figure 1 highlights how it is integrated into the stress ulcer prophylaxis research program. Patients with COVID-19 have been eligible for REVISE since March 11, 2020. Despite the difficulties of continuing non-COVID research in ICUs during the pandemic, recruitment to REVISE was paused for the shortest possible period in each participating center throughout the pandemic. The objectives, a priori hypotheses and outcomes for this COVID-19 study are outlined in Table 1. Although the protocol described in this work would be characterized as a study within a trial, we adhered to the Standard Protocol Items for Clinical Trials (SPIRIT) reporting guidelines where appropriate [16]. Accordingly, the recommended SPIRIT diagram was not included in this work, this will be published as part of the full REVISE randomized trial protocol.

Setting

Patients are being enrolled in 63 ICUs worldwide (39 Canada, 19 Australia, 1 Saudi Arabia, 1 the UK, 1 the US, 1 Kuwait, and 1 Pakistan).

Population

Patients are eligible for this COVID-19 substudy according to the inclusion and exclusion criteria of the REVISE Trial, as outlined in Table 2. Randomization is not stratified by COVID-19 disease status. The inclusion in the COVID-19 cohort will be based on COVID-19 viral nucleic acid test positivity by polymerase chain reaction (PCR) from a nasopharyngeal swab, oropharyngeal swab, sputum, endotracheal aspirate, or bronchoalveolar lavage sample, as clinically determined rather than per protocol. The COVID-19 classification in this study will be made via PCR testing, due to the heightened risk for misclassification using other forms of testing, which will not

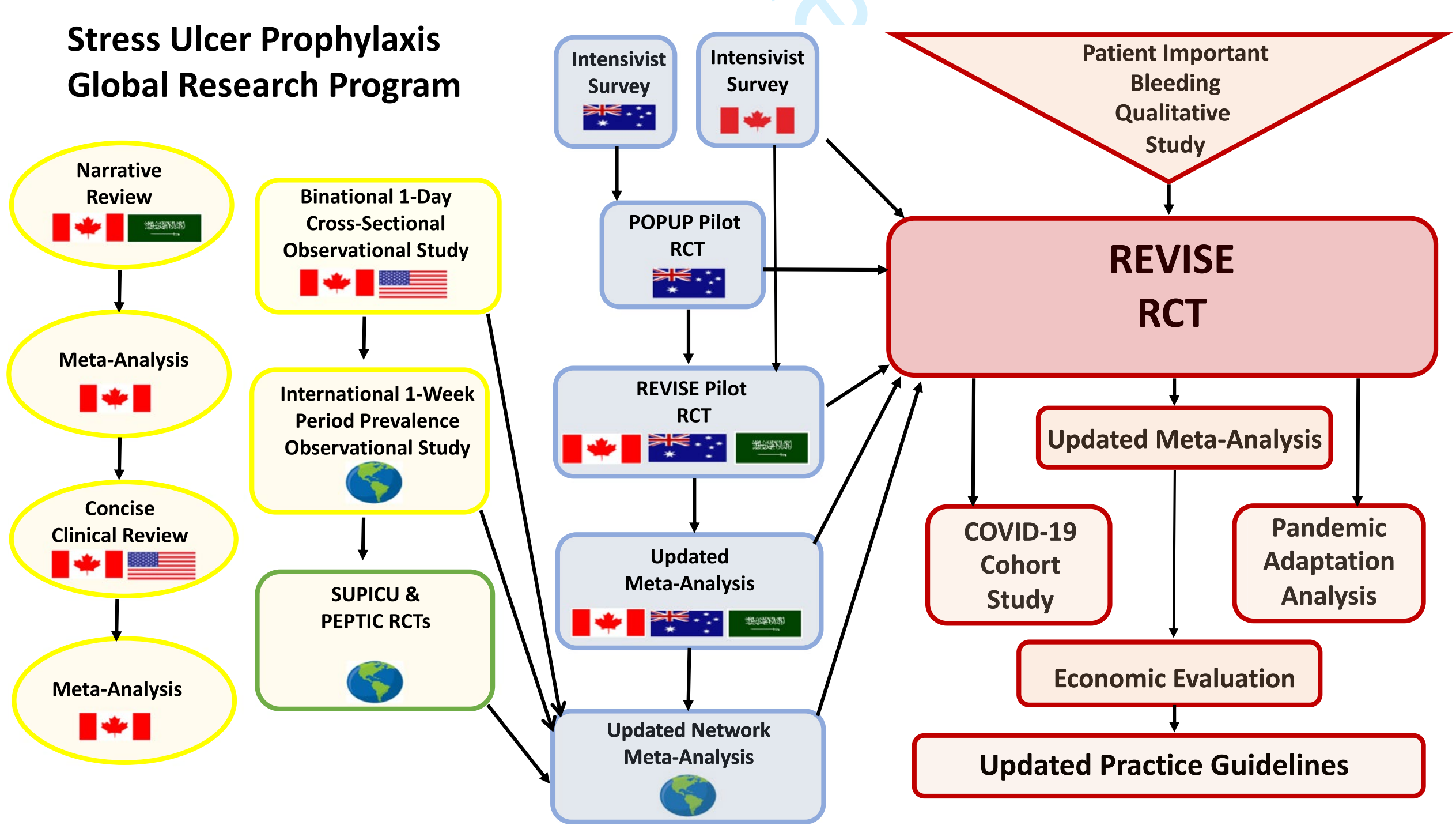

Fig. 1 COVID-19 cohort study within the stress ulcer prophylaxis research program. This figure displays how this COVID cohort study is integrated into the international stress ulcer prophylaxis research program

For peer review only - http://bmjopen.bmj.com/site/about/guidelines.xhtml

Table 1 Overview of research objectives, hypotheses, outcomes, and analytic methods

Objectives	Hypotheses	Outcomes	Methods of analysis
**Question 1:** What prognostically relevant demographic, biomarker, and clinical data characterize invasively ventilated patients with COVID-19? (Explanatory variable: not applicable; no comparisons)			
To establish a clinical profile of critically ill patients with COVID-19, incorporating demographics, biomarkers, rates of venous thromboembolism, COVID-19 therapies, duration of mechanical ventilation, ICU, hospital stay, and tracheostomy timing	Patients with COVID-19 will have a high burden of comorbidity and, as a consequence of their underlying severe viral illness, will exhibit high inflammatory biomarkers (D-dimer, CRP, ferritin), receive targeted COVID-19 therapies, experience long duration of mechanical ventilation, and undergo tracheostomy later than current norms	Description of demographics, biomarkers, and course in the ICU including venous thromboembolism rates and tracheostomy timing	Descriptive analysis<br>Continuous variables will be summarized using mean ± standard deviation (SD), while frequencies and percentages will be used to summarize categorical variables
**Question 2:** Do critically ill patients with COVID-19 have higher rates of clinically important GI bleeding, 90-day mortality, and infection compared to a propensity-matched non-COVID cohort? (explanatory variable: COVID-19)			
To understand the impact of COVID-19 on CIGIB, 90-day mortality, and rates of VAP, *C. difficile* infection, and patient-important bleeding; use of renal replacement therapy; and duration of mechanical ventilation, ICU stay and hospital stay, and ICU and hospital mortality compared to patients without COVID-19	Patients with COVID-19 will have a poorer prognosis than propensity-matched control patients without COVID-19. Patients with COVID-19 will have higher rates of CIGIB, 90-day mortality, VAP, *C. difficile* infection, and patient-important GI bleeding, use of renal replacement therapy, ICU and hospital mortality, and longer durations of mechanical ventilation and ICU and hospital stay, compared to a propensity-matched non-COVID cohort	CIGIB, 90-day mortality<br>VAP, *C. difficile* infection, patient-important bleeding, and renal replacement therapy<br>Duration of mechanical ventilation, ICU stay, and hospital stay<br>ICU and hospital mortality	Propensity-matched analysis of patients in REVISE with and without COVID-19 infection
**Question 3**: Do critically ill patients with COVID-19 projected to receive mechanical ventilation for > 48 h have different clinical outcomes with versus without PPIs? (explanatory variable: pantoprazole)			
To examine whether pantoprazole has a different treatment effect on CIGIB or 90-day mortality in patients with and without COVID-19 (REVISE subgroup analysis)<br>To determine whether pantoprazole has a differential treatment effect in patients with or without COVID-19 on VAP, *C. difficile* infection, patient-important GI bleeding, use of renal replacement therapy, hospital mortality, and duration of mechanical ventilation, ICU stay and hospital stay, and ICU and hospital mortality	We hypothesize no modification of the effect of pantoprazole on CIGIB. We hypothesize that pantoprazole is more harmful in patients with versus without SARS-CoV-2 in terms of increased risk of 90-day mortality<br>We hypothesize no modification of the effect of pantoprazole on VAP, *C. difficile* infection, patient-important GI bleeding, use of renal replacement therapy, and duration of mechanical ventilation, ICU stay and hospital stay, and ICU and hospital mortality. We hypothesize that pantoprazole is more harmful in patients with versus without SARS-CoV-2 in terms of increased risk of hospital mortality	CIGIB, 90-day mortality (REVISE subgroup analysis)<br>VAP, *C. difficile* infection, patient-important bleeding, and renal replacement therapy (extended outcomes)<br>Duration of mechanical ventilation, ICU stay and hospital stay, and ICU and hospital mortality (extended outcomes)	Subgroup analysis: Comparison of outcomes for patients with and without COVID-19 receiving either pantoprazole or placebo<br>Comparison of the time to the primary and secondary binary outcomes in the two groups using Cox proportional hazards regression with threshold *P*-values of 0.05, adjusted for the center and pre-hospital use of acid suppression (stratification variables). A test of interaction will be performed for this subgroup analysis in REVISE, and the results will be interpreted per the ICEMAN criteria<br>For binary outcomes, we will report hazard ratios with 95% CIs as well as the absolute risk increase or decrease and 95% CIs<br>For continuous outcomes, we will compare the two arms using a *t*-test on the log scale (to normalize the distribution) or a nonparametric approach if needed

Research question, objectives, hypotheses, outcomes, and analytic approaches for the COVID-19 REVISE substudy

CIGIB clinically important GI bleeding, *ICU* intensive care unit, *PPI* proton pump inhibitor, *COVID-19* coronavirus disease of 2019, *VAP* ventilator-associated pneumonia, *C. difficile Clostridioides difficile*

For peer review only

For peer review only - http://bmjopen.bmj.com/site/about/guidelines.xhtml

Table 2 Inclusion and exclusion criteria for REVISE

**Inclusion criterion**	
Adults ≥ 18 years old projected to receive invasive mechanical ventilation for ≥ 48 h according to the treating physician	
**Exclusion criteria**	
1. Already received invasive mechanical ventilation ≥ 72 h during this hospital admission	
2. Acid suppression for active GI bleeding or high risk of bleeding (e.g., current bleeding, peptic ulcer bleeding within 8 weeks, recent severe esophagitis, Barrett's esophagus, Zollinger-Ellison syndrome) [dyspepsia or gastroesophageal reflux is not an exclusion criterion]	
3. Acid suppression in the ICU for > 1 daily dose equivalent of a PPI or H2RA	
4. Dual antiplatelet therapy	
5. Combined antiplatelet and therapeutic anticoagulation	
6. Pantoprazole contraindication per local product information	
7. Palliative care or anticipated withdrawal of advanced life support	
8. Pregnancy	
9. Previous enrollment in REVISE, a related trial, or trial for which co-enrollment is prohibited	
10. Patient, proxy, or physician declines	

In this table, we list the eligibility criteria for this study, based on the inclusion and exclusion criteria for the REVISE trial

GI gastrointestinal, *ICU* intensive care unit, *PPI* proton pump inhibitor, *H2RA* histamine-2-receptor antagonists

be accepted for COVID-19 diagnosis, including antigen testing. Patients will be eligible for this substudy if they test positive for SARS-CoV-2 in the month preceding the index ICU admission, whether pre-hospital, in hospital, or in the ICU. Patients diagnosed as contracting SARS-CoV-2 more than 48 h after enrollment in REVISE will be ineligible.

Summary of outcomes for REVISE

The primary *efficacy* outcome for REVISE is clinically important GI bleeding occurring in the ICU or resulting in ICU readmission during the index hospital stay. Adjudicator agreement for this metric was excellent in two prior studies [17, 18] which was associated with increased attributable length of stay and mortality [19]. The definition of clinically important GI bleeding is overt GI bleeding (i.e., hematemesis, frank blood or coffee ground nasogastric aspirate, melena or hematochezia) *plus 1 of the following in the absence of other causes*: (1) hemodynamic change defined as a spontaneous decrease in invasively monitored mean arterial pressure or non-invasive systolic or diastolic blood pressure of ≥ 20 mmHg or an orthostatic increase in pulse rate of ≥ 20 beats/min and a decrease in systolic blood pressure of ≥ 10 mmHg, with or without vasopressor initiation or increase; (2) vasopressor initiation; (3) hemoglobin decrease of ≥ 2 g/dL (20 g/L) within 24 h of bleeding; (4) transfusion of ≥ 2 units red blood cells within 24 h of bleeding; or (5) therapeutic intervention (e.g., therapeutic endoscopy, angioemoblization, surgery). The primary *safety* outcome is mortality defined as all-cause mortality at 90 days after randomization to evaluate a suggestion of increased risk of death associated with pantoprazole among the sickest subgroup of patients in a recent placebo-controlled trial [20]. Secondary outcomes for REVISE are listed in Table 3.

Table 3 Secondary outcome definitions in REVISE

Ventilator-associated pneumonia	VAP is diagnosed in patients who received invasive mechanical ventilation for ≥ 48 h when there is a new, progressive, or persistent radiographic infiltrate *plus at least 2 of the following without other obvious cause*: (1) fever (temperature > 38 °C) or hypothermia (temperature < 36 °C), (2) leukopenia (< 4.0×10^6/L) or leukocytosis (> 12×10^6/L), (3) purulent sputum, or (4) gas exchange deterioration. Given no universally accepted VAP criteria, data collection and central adjudication allows analysis of several other VAP definitions. We do not collect infection-related ventilator-associated conditions as they are modifiable by ventilator settings and volume status
C. difficile infection	Defined as clinical features (diarrhea [≥ 3 episodes of unformed stools or Bristol type 6 or 7], ileus, or toxic megacolon) and either microbiological evidence of toxin-producing *C. difficile* or pseudomembranous colitis on colonoscopy in hospital
Patient-important GI bleeding	Defined as overt GI bleeding, *plus* an invasive intervention (e.g., therapeutic endoscopy, angioembolization, surgery), acknowledging how some clinically important GI bleeds in prior studies did not actually require any tests or treatments, and thus may not be important to patients, to be refined following completion of patient and family interviews from a Patient Important Bleeding Study [NCT05506150]
Renal replacement therapy	Defined as the initiation of new renal replacement therapy in the ICU
Hospital mortality	Defined as all-cause mortality in the hospital

In this table, we show the secondary outcomes and their definitions for the REVISE trial

VAP ventilator-associated pneumonia, *ICU* intensive care unit, *GI* gastrointestinal, *C. difficile Clostridioides difficile*

For peer review only

Pilot work

Investigators developed a one-page draft case report form to capture new information which was not part of the original REVISE trial to better characterize the COVID-19 cohort. A group of eight research coordinators reviewed and refined the form to capture COVID-19 vaccination status, biomarkers, venous thromboembolic events, tracheostomy occurrence and timing, and COVID-19 therapies as per Table 4. The form was pre-tested on 15 patients by abstracting data from either electronic or paper-based medical records. Accessing the chart, scanning contents, extracting relevant information, and entering data into the password-protected REVISE website required an estimated 40 min per patient. After this pilot exercise, we oriented research coordinators in participating centers to the study objectives, methods, and the case report form during telephone, videoconference, and in-person investigators' meetings.

Data collection and outcomes for the COVID-19 cohort study

Retrospective data collection for newly added variables for the COVID-19 study was required for participants enrolled prior to the implementation of this COVID-19 study protocol. Following relevant ethics review, COVID-19-specific variables were incorporated into existing data-collection forms, affording prospective data collection for COVID-19 variables for the remainder of the study. For any patients transferred to non-REVISE centers during the pandemic, all efforts are being made to complete the COVID-19 case report form, including a collection of data on outcomes occurring after transfer to a non-REVISE center.

Biologic specimens (e.g., sputum culture for COVID-19 testing) and laboratory results (blood samples to evaluate for hemoglobin levels) are collected as part of routine medical care. These samples are not being procured or stored for study purposes.

Data management and quality control

Data management and quality control will be overseen by the REVISE Methods Centers, located at McMaster University in Hamilton and The George Institute (TGI) in Sydney. These centers are responsible for ensuring optimal trial conduct. Oversight and quality control will be achieved through close collaboration between these centers, who hold twice monthly teleconferences to harmonize approaches and share management efficiencies. The Methods Center Leads and relevant investigators agreed to train local principal investigator(s), research coordinator(s), and research pharmacist(s), on the protocol. Throughout the trial, the methods center staff will engage in compliance-enhancing strategies, set targets, and track progress. REVISE analyst Ms. D Heels-Ansdell will conduct periodic central statistical monitoring to generate overall and site-specific metrics for internal use and site feedback. The methods center staff will adopt a risk-adapted approach to monitor each site (in-person or by videoconference per pandemic adaptations) to assess and enhance protocol fidelity. The foci will include eligibility adherence, stratification, informed consent documentation, regulatory compliance, and case report form accuracy.

In the event of any modifications made to the existing protocol for the COVID-19 substudy, the REVISE methods center will take responsibility for notifying all participating centers, sponsors, and funding institutions. All participating centers would receive a copy of the revised protocol and meet to review and discuss any implications of such changes to study implementation.

Sample size

The cohort size is based on the number of patients with COVID-19 enrolled in REVISE. As of June 1, 2023, 484 patients with COVID-19 have been enrolled in REVISE, representing 10.1% of the sample size of 4800 patients. Acknowledging the inherent challenges in predicting the incidence of and the means ± standard deviation (SD)

Table 4 COVID cohort substudy biomarkers and other clinical characteristics

New variables	Definitions and measures
**D-dimer**	Highest D-dimer level (μg/L) recorded post-COVID diagnosis
**C-reactive protein**	Highest C-reactive protein level (mg/L) recorded post-COVID diagnosis
**Ferritin level**	Highest ferritin level (μg/L) recorded post-COVID diagnosis
**Venous thromboembolism**	Defined as (1) new symptomatic or asymptomatic deep-vein thrombosis or pulmonary embolism, diagnosed using accepted imaging tests in practice
**Days of mechanical ventilation**	Defined as the number of days requiring mechanical ventilation either invasive or non-invasive
**Tracheostomy and timing of insertion**	Defined as time from intubation to tracheostomy insertion during the ICU stay

In this table, we list the additional data required for this COVID-19 C cohort study

COVID-19 coronavirus disease of 2019, *ICU* intensive care unit

For peer review only - http://bmjopen.bmj.com/site/about/guidelines.xhtml

or median (interquartile range [IQR]) of the continuous variables will be calculated. Baseline characteristics (e.g., age, sex, APACHE II Score, admission diagnostic category, comorbidities, COVID-19 vaccination status, and pre-hospital acid suppression) will be described. Table 1 presents the statistical analysis plans for the 3 study objectives, hypotheses, and outcomes.

For any outcome that is missing for more than 2% of patients, we will perform multiple imputation. Adjustments for multiple comparisons will not be made for these exploratory analyses. All analyses will be performed using the statistical software SAS version 9.4 [21].

Analysis of objective 1: Characterization of the COVID-19 cohort

New continuous variables added to the REVISE dataset to help characterize the COVID-19 cohort of patients include inflammatory biomarker levels and time until tracheostomy. New dichotomous variables include the incidence of thromboembolic events and treatment with COVID-19-specific therapies. Continuous variables will be summarized using mean ± standard deviation (SD), or median (interquartile range [IQR]), while frequencies and percentages will be used to summarize categorical variables.

Analysis of objective 2: Propensity-matched comparison of patients with and without COVID-19

To explore whether patients with COVID-19 experience different clinical outcomes relative to patients without COVID-19, we will compare the COVID-19 cohort and a propensity-matched non-COVID cohort from REVISE. Propensity score matching attempts to estimate the effect of COVID-19 status by accounting for important covariates that may predict or explain this status. Patients with COVID-19 will be matched with other patients within REVISE using propensity score analysis. We will consider an optimal matching ratio of 2:1 or 1:1 depending on the availability of optimal matches.

The propensity score will be generated using potentially important pre-randomization demographic variables, including age, sex, pre-hospital acid suppression (PPIs or histamine-2-receptor antagonists (H2RAs)), COVID-19 vaccination status, ICU admission category of medical or surgical/trauma, and date of enrollment. Propensity matching will only include patients enrolled after the declaration of the COVID-19 pandemic on March 11, 2020. As a sensitivity analysis, we will match only on pre-hospital PPI use, not H2RAs, given limited evidence to suggest that H2RAs influence susceptibility to COVID-19 infection [22].

Propensity score matching helps to ensure that the distribution of the foregoing covariates is similar between the compared populations [23]. Comparisons between the COVID-19 and matched cohorts will be made on clinically important bleeding and 90-day mortality, incidence of VAP, *C. difficile* infection, patient-important GI bleeding, renal replacement therapy, ICU and hospital mortality, and duration of mechanical ventilation and ICU and hospital stay. We will adjust all analyses for randomized allocation; mortality outcomes will be adjusted for the APACHE II score.

Objective 3: Treatment effect of PPIs in patients with COVID-19 versus other patients

We will explore whether pantoprazole in the ICU has a differential treatment effect on clinically important GI bleeding or 90-day mortality for patients with COVID-19 relative to those without COVID-19. This is a subgroup analysis of the primary REVISE trial. However, in addition, we aim to further evaluate the subgroup effects of pantoprazole in patients with COVID-19 across secondary REVISE outcomes including VAP and *C. difficile* infection, patient-important bleeding, renal replacement therapy, and ICU and hospital mortality, as well as the duration of mechanical ventilation and ICU and hospital stay.

This analysis will be conducted per the intention-to-treat principle. For time to the primary and secondary binary outcomes, we will use Cox proportional hazards regression, adjusted for pre-hospital use of acid suppression (a stratification variable). Analysis of our mortality outcomes will also be adjusted for the APACHE II score. An interaction term will be included in the model to test for this subgroup effect and results will be interpreted per the ICEMAN criteria [24]. We will report hazard ratios with 95% CIs as well as the absolute risk increase or decrease and 95% CIs. For continuous outcomes, we will perform linear regression which will include an interaction term. We will report mean differences with 95% CIs. If needed, we will perform the linear regression on the log-transformed outcome.

Ethics and regulatory approval

The main REVISE trial was approved in participating hospitals by all relevant ethics committees and regulatory authorities prior to starting enrollment. The Hamilton Integrated Research Ethics Board (HiREB) under Clinical Trials Ontario (CTO Study ID 1360) is the main REB of record, which also approved this substudy. Ethics review for the additional COVID-19-specific data collection was undertaken by the Research Ethics Board of record

For peer review only

(the Hamilton Integrated Research Ethics Board), and as needed to comply with local regulations in each participating center. In the absence of any modifications to the REVISE study design, this substudy involved additional data collection to characterize the patients with COVID-19 and facilitate additional future analyses. Therefore, no modifications were made to the consenting process for REVISE patients involved in this embedded COVID-19 study, and no requirements were issued to either reconsent or inform prior REVISE participants of the substudy. Specifically, there was no requirement to inform participants of this substudy, including an additional review of participants' medical records for new data collection. Participants previously randomized in REVISE were not re-consented to participate in the COVID-19 embedded substudy.

Integrated knowledge translation plan

After completion of the COVID-19 REVISE substudy, we will rapidly publish the results. All results will be published with open access to ensure wide dissemination and accessibility of study findings. We plan to publish and present the COVID-19 substudy work at an international congress. We will disseminate a lay summary of findings to the general public through health networks of patient advocates and persons with lived or living experiences. We will generate lay language summaries and visual abstracts for traditional (paper, radio, television) and social media. We will also host videoconferences and regional rounds. We will disseminate structured abstracts and slide decks to local ICU quality councils, provincial organizations (e.g., Alberta Health Services Critical Care Strategic Clinical Network), national policymakers (e.g., Accreditation Canada), and professional societies. Participating organizations, sponsors, and university institutions will be provided with press release briefs to disseminate to their respective audiences.

Discussion

This study, embedded within a global, randomized trial of stress ulcer prophylaxis, will fill important knowledge gaps in the literature that prompted our three research questions and three study objectives. The first is a descriptive analysis of the COVID-19 cohort in REVISE. The second is a propensity-matched analysis of patients with and without COVID-19. The third is an extended subgroup analysis of the REVISE trial, evaluating the effect of pantoprazole on a range of additional clinical outcomes among patients with and without COVID-19.

Evidence describing the association between acid suppression and poor prognosis among patients with COVID-19 has emerged, but results have been variable. To prevent complications among critically ill patients with COVID-19, interim WHO guidance in 2020 suggested H2RAs or PPIs for patients with risk factors for GI bleeding, including mechanical ventilation for ≥ 48 h, coagulopathy, renal replacement therapy, liver disease, multiple comorbidities, and higher organ failure score [25]. Surviving Sepsis Guidelines for the management of critically ill patients with COVID-19 do not address stress ulcer prophylaxis [26]. Randomized trial data on the effect of PPIs among patients with COVID-19 is insufficient to guide clinical practice, and this study will provide direct evidence on this topic.

Limitations of this substudy include the risk of missing 90-day outcomes due to high levels of inter-hospital transfers experienced during COVID-19 [27]. Patients transferred to centers not participating in REVISE or other loss to follow-up. Our propensity analysis cannot adjust for all possible factors predisposing to infection with SARS-CoV-2. We will have no information on patients eligible for REVISE who were not approached for the trial due to concerns about bleeding risk due to COVID-19 therapies or increased risk of VAP due to COVID-19 infection itself. In addition, the number of patients enrolled in REVISE with COVID-19 depends on the pandemic status in participating centers, such that no sample size calculation is possible; thus, we will interpret the findings in light of the 95% confidence intervals.

Strengths of this study include the timely development of a protocol addressing essential research questions relevant to a high-risk subgroup of critically ill mechanically ventilated patients that emerged during the pandemic. The main REVISE Trial [NCT03374800], and this COVID-19 Cohort Study [NCT05715567] are registered. We have specified a priori hypotheses for each of the 3 objectives. As this study was developed after the REVISE Trial began recruitment, we are using both retrospective and prospective data collection methods to identify relevant patients with COVID-19. All necessary data is derived from the hospital stay, except 90-day mortality for those discharged alive; thus, we anticipate minimal missing data.

Trial status

As of June 1, 2023, more than 90% of relevant data for REVISE patients with COVID-19 are in the REVISE database to inform this work. REVISE enrollment is anticipated to close byNovember 2023; 90-day follow-up, chart closure, and analyses for this study will follow later in 2024.

For peer review only - http://bmjopen.bmj.com/site/about/guidelines.xhtml

Acknowledgements
The trial was designed by the REVISE Steering Committee including the National and International Management Committees, the REVISE Investigators and Research Coordinators, the Canadian Critical Care Trials Group, and the Australian and New Zealand Intensive Care Society Clinical Trials Group. We are grateful to others at the Methods Center at McMaster University (Lisa Buckingham, France Clarke, Mary Copland, Karlo Matic, Ashley Sawyer, Alyson Takaoka) and The George Institute (Fatima Butt, Anna Cheng, Conrad Nangla, Fiona Osborne, Tina Schneider, Isabella Schoeler, Anna Tippet) for their expertise.

Authors' contributions
Concept and design: Y Arabi, A Binnie, D Cook, A Deane, B Dennis, J Dionne, S English, S Finfer, G Guyatt, M Hardie, D Heels-Ansdell, S Knowles, F Lauzier, J Marshall, J Muscedere, J Myburgh, M Ostermann, L Thabane, J Tsang, and N Zytaruk. Acquisition, analysis, or interpretation of the data: D Cook, B Dennis, M Hardie, D Heels-Ansdell, S Knowles, L Saunders, L Thabane, and N Zytaruk. Drafting of the manuscript: D Cook, B Dennis, D Heels-Ansdell, and N Zytaruk. Statistical analysis: B Dennis, D Heels-Ansdell, and L Thabane. Obtained funding: A Ahmed, A Binnie, D Cook, B Dennis, J Dionne, G Guyatt, D Heels-Ansdell, L Thabane, and J Tsang. Administrative, technical, or material support: D Cook, A Deane, B Dennis, S Finfer, G Guyatt, M Hardie, D Heels-Ansdell, S Knowles, F Lauzier, D Rajbhandari, L Saunders, A Poole, L Thabane, and N Zytaruk. Data integrity: D Cook, B Dennis, M Hardie, D Heels-Ansdell, S Knowles, L Saunders, and N Zytaruk. Ethics approval and consent to participate: not applicable for this retrospective chart review protocol. Approved final manuscript: all authors.

Funding
REVISE is funded by peer-reviewed grants [Canadian Institutes of Health Research 201610PJT-378226-PJT-CEBA-18373 and Canadian Institutes of Health Research 202207CL3-492565-CTP-CEBA-19215], the Hamilton Academy of Health Sciences Organization [HAH-22–009], Physician Services Incorporated (R22-33) and funds from St. Joseph's Healthcare Hamilton and McMaster University. The National Health and Medical Research Council of Australia grant [GNT1124675] funds enrollment in Australia. REVISE was approved by the National Institute for Health Research (NIHR) in the UK as a Portfolio Study [CPMS ID 45782], eligible for support from the NIHR Clinical Research Network. [https://www.nihr.ac.uk/researchers/collaborations-services-and-support-for-your-research/run-your-study/crn-portfolio.htm]. This trial received no support from the commercial or private sector. The funders/sponsors have no role in the conception, design, conduct, oversight, analysis, interpretation, write-up, review, or approval of the manuscript or the decision to submit the manuscript for publication.
Role of the funders: The funders/sponsors have no role in the conception, design, conduct, oversight, analysis, interpretation, write-up, review, or approval of the manuscript or the decision to submit the manuscript for publication.
Composition and responsibilities of participating centers and steering committee: Led by the Canadian Critical Care Trials Group and the Australian & New Zealand Intensive Care Society Clinical Trials Group, the REVISE Methods Centers are located at McMaster University in Hamilton for international centers and The George Institute in Sydney for Australia. These centers (REVISE Methods Centre, The George Institute) housed the primary data management and endpoint adjudication teams, who worked in close collaboration to ensure standardization of data collection and adjudication procedures. The REVISE Canadian Steering Committee comprises Drs. Guyatt, Cook, Alhazzani, Marshall, Lauzier, English, Muscedere, Hall, Xie, and Thabane. The International Management Committee includes Drs. Finfer and Myburgh, Deane, Ostermann, and Arabi joined by Canadian leaders Drs. Guyatt, Cook, Alhazzani, Marshall, and Thabane. These teams meet approximately quarterly, to anticipate and pre-empt challenges and generate solutions to the operational and financial hurdles characterizing global trials. The Data Safety & Monitoring Committee includes Professor Roberts (University of Oxford), Dr. McAuley (Queen's University Belfast), and Chair Dr. Thomlinson (University of Toronto).

Availability of data and materials
Readers are welcome to contact the research team for further information. There are no original data associated with this research protocol.
The datasets analyzed during the current study would only be available following relevant research ethics board discussion and approval. However, the statistical code will be available and any requested details of the protocol.

Declarations

Ethics approval and consent to participate
The main REVISE trial was approved in participating hospitals by all relevant ethics committees and regulatory authorities prior to starting enrollment. The Hamilton Integrated Research Ethics Board (HiREB) under Clinical Trials Ontario (CTO Study ID 1360) is the main REB of record, which also approved this substudy. Ethics review for the additional COVID-19-specific data collection was undertaken as needed to comply with local regulations in each participating center.

Consent for publication
Not applicable for this protocol.

Competing interests
All authors are involved in the REVISE Trial in some capacity. Otherwise, the authors declare that they have no competing interests.

Author details
[1]Department of Medicine, McMaster University, Hamilton, ON, Canada. [2]Department of Health Research Methods, Evidence, and Impact, McMaster University, Hamilton, ON, Canada. [3]Biostatistics Unit, St. Joseph's Healthcare Hamilton, Hamilton, ON, Canada. [4]Division of Critical Care, Research Institute, St. Joseph's Healthcare Hamilton, Hamilton, ON, Canada. [5]Departments of Medicine and Health Research Methods, Evidence, and Impact, McMaster University, McMaster University Health Sciences Center, Room 2C11, 1200 Main Street West, Hamilton, ON L8N 3Z5, Canada. [6]Department of Critical Care Medicine, Niagara Health System, St. Catharines, ON, Canada. [7]Department of Gastroenterology and Hepatology, Stanford University, Palo Alto, CA, USA. [8]Departments of Anesthesiology and Medicine and Critical Care Medicine, Université Laval, Québec, Québec, Canada. [9]Department of Critical Care Medicine, University of Melbourne, Melbourne Medical School, Parkville, VIC, Australia. [10]Intensive Care Department, Ministry of the National Guard-Health Affairs, Riyadh, Kingdom of Saudi Arabia. [11]King Saud Bin Abdulaziz University for Health Sciences, Riyadh, Kingdom of Saudi Arabia. [12]King Abdullah International Medical Research Center, Riyadh, Kingdom of Saudi Arabia. [13]Interdepartmental Division of Critical Care, University of Toronto, Toronto, ON, Canada. [14]Critical Care Program, The George Institute for Global Health, Faculty of Medicine, University of New South Wales, Sydney, NSW, Australia. [15]Intensive Care Unit, St. George Hospital, Sydney, Australia. [16]Department of Critical Care Medicine, Queen's University, Kingston, ON, Canada. [17]Department of Medicine, University of Ottawa, Ottawa, ON, Canada. [18]Clinical Epidemiology Program, Ottawa Hospital Research Institute, Ottawa, ON, Canada. [19]Department of Critical Care, King's College London, Guy's & St Thomas' Hospital, London, UK.

Received: 3 July 2023 Accepted: 16 August 2023
Published online: 30 August 2023

References
1. Richardson S, Hirsch JS, Narasimhan M, Crawford JM, McGinn T, Davidson KW, Barnaby DP, Becker LB, Chelico JD, Cohen SL, et al. Presenting characteristics, comorbidities, and outcomes among 5700 patients hospitalized with COVID-19 in the New York City area. JAMA. 2020;323(20):2052.
2. Guan W-J, Ni Z-Y, Hu Y, Liang W-H, Ou C-Q, He J-X, Liu L, Shan H, Lei C-L, Hui DSC, et al. Clinical characteristics of coronavirus disease 2019 in China. N Engl J Med. 2020;382(18):1708–20.
3. Zhang P, Zhu L, Cai J, Lei F, Qin J-J, Xie J, Liu Y-M, Zhao Y-C, Huang X, Lin L, et al. Association of inpatient use of angiotensin-converting enzyme inhibitors and angiotensin II receptor blockers with mortality among patients with hypertension hospitalized with COVID-19. Circ Res. 2020;126(12):1671–81.
4. Reynolds HR, Adhikari S, Pulgarin C, Troxel AB, Iturrate E, Johnson SB, Hausvater A, Newman JD, Berger JS, Bangalore S, et al. Renin–angiotensin–aldosterone system inhibitors and risk of COVID-19. N Engl J Med. 2020;382(25):2441–8.
5. Lopes RD, Macedo AVS, De BarrosESilva PGM, Moll-Bernardes RJ, Dos Santos TM, Mazza L, Feldman A, D'AndréaSabaArruda G, De Albuquerque

For peer review only

DC, Camiletti AS, et al. Effect of discontinuing vs continuing angiotensin-converting enzyme inhibitors and angiotensin II receptor blockers on days alive and out of the hospital in patients admitted with COVID-19. JAMA. 2021;325(3):254.
6. Xiang Y, Wong KC, So HC. Exploring drugs and vaccines associated with altered risks and severity of COVID-19: a UK Biobank cohort study of all ATC level-4 drug categories reveals repositioning opportunities. Pharmaceutics. 2021;13(9):1514.
7. Lee SW, Ha EK, Yeniova AÖ, Moon SY, Kim SY, Koh HY, Yang JM, Jeong SJ, Moon SJ, Cho JY, et al. Severe clinical outcomes of COVID-19 associated with proton pump inhibitors: a nationwide cohort study with propensity score matching. Gut. 2021;70(1):76–84.
8. Aby ES, Tappata M, Rodin H, Debes JD. Assessment of proton-pump inhibitor use, race, socioeconomic status, and mortality in individuals with COVID-19. Eur J Gastroenterol Hepatol. 2022;34(2):239.
9. Hariyanto TI, Prasetya IB, Kurniawan A. Proton pump inhibitor use is associated with increased risk of severity and mortality from coronavirus disease 2019 (COVID-19) infection. Dig Liver Dis. 2020;52(12):1410–2.
10. Krag M, Perner A, Wetterslev J, Wise MP, Borthwick M, Bendel S, McArthur C, Cook D, Nielsen N, Pelosi P, et al. Prevalence and outcome of gastrointestinal bleeding and use of acid suppressants in acutely ill adult intensive care patients. Intensive Care Med. 2015;41(5):833–45.
11. Cook D, Guyatt G. Prophylaxis against upper gastrointestinal bleeding in hospitalized patients. N Engl J Med. 2018;378(26):2506–16.
12. Re-EValuating the Inhibition of Stress Erosions (REVISE) Trial (REVISE), [NCT03374800]. Clinical trial registry from clinicalTrials.gov; 2018. https://classic.clinicaltrials.gov/ct2/show/NCT03374800. Accessed 28 Aug 2023.
13. Ippolito M, Misseri G, Catalisano G, Marino C, Ingoglia G, Alessi M, Consiglio E, Gregoretti C, Giarratano A, Cortegiani A. Ventilator-associated pneumonia in patients with COVID-19: a systematic review and meta-analysis. Antibiotics (Basel). 2021;10(5):545.
14. Dexamethasone in hospitalized patients with COVID-19. N Engl J Med. 2020;384(8):693–704. https://www.nejm.org/doi/full/10.1056/NEJMoa2021436.
15. Therapeutic anticoagulation with heparin in critically ill patients with COVID-19. N Engl J Med. 2021;385(9);777–89. https://www.nejm.org/doi/full/10.1056/NEJMoa2021436.
16. Chan AW, Tetzlaff JM, Altman DG, Laupacis A, Gøtzsche PC, Krleža-Jerić K, Hróbjartsson A, Mann H, Dickersin K, Berlin JA, et al. SPIRIT 2013 statement: defining standard protocol items for clinical trials. Ann Intern Med. 2013;158(3):200–7.
17. Cook D, Guyatt G, Marshall J, Leasa D, Fuller H, Hall R, Peters S, Rutledge F, Griffith L, McLellan A, et al. A comparison of sucralfate and ranitidine for the prevention of upper gastrointestinal bleeding in patients requiring mechanical ventilation. N Engl J Med. 1998;338(12):791–7.
18. Cook DJ, Fuller HD, Guyatt GH, Marshall JC, Leasa D, Hall R, Winton TL, Rutledge F, Todd T, Roy P, et al. Risk factors for gastrointestinal bleeding in critically ill patients. N Engl J Med. 1994;330(6):377–81.
19. Cook DJ, Griffith LE, Walter SD, Guyatt GH, Meade MO, Heyland DK, Kirby A, Tryba M. Crit Care. 2001;5(6):368.
20. Krag M, Marker S, Perner A, Wetterslev J, Wise MP, Schefold JC, Keus F, Guttormsen AB, Bendel S, Borthwick M, et al. Pantoprazole in patients at risk for gastrointestinal bleeding in the ICU. N Engl J Med. 2018;379(23):2199–208.
21. SAS 9.4. In. Edited by Inc SI. Cary, NC.
22. Hirota K. H_2 antagonists, proton pump inhibitors and COVID-19. J Anesth. 2022;36(3):329–31.
23. Rosenbaum PR, Rubin DB. The central role of the propensity score in observational studies for causal effects. Biometrika. 1983;70(1):41–55.
24. Schandelmaier S, Briel M, Varadhan R, Schmid CH, Devasenapathy N, Hayward RA, Gagnier J, Borenstein M, van der Heijden GJMG, Dahabreh IJ, et al. Development of the Instrument to assess the Credibility of Effect Modification Analyses (ICEMAN) in randomized controlled trials and meta-analyses. Can Med Assoc J. 2020;192(32):E901–6.
25. World Health O. Clinical management of severe acute respiratory infection (SARI) when COVID-19 disease is suspected: interim guidance, 13 March 2020. In. Geneva: World Health Organization; 2020.
26. Alhazzani W, Evans L, Alshamsi F, Møller MH, Ostermann M, Prescott HC, Arabi YM, Loeb M, Ng Gong M, Fan E, et al. Surviving sepsis campaign guidelines on the management of adults with coronavirus disease 2019 (COVID-19) in the ICU: first update. Crit Care Med. 2021;49(3):e219–34.
27. Pett E, Leung HL, Taylor E, Chong MSF, Hla TTW, Sartori G, Sathianathan V, Husain T, Suntharalingam G, Rosenberg A, et al. Critical care transfers and COVID-19: managing capacity challenges through critical care networks. J Intensive Care Soc. 2022;23(2):203–9.

Publisher's Note

Springer Nature remains neutral with regard to jurisdictional claims in published maps and institutional affiliations.

Ready to submit your research? Choose BMC and benefit from:

- fast, convenient online submission
- thorough peer review by experienced researchers in your field
- rapid publication on acceptance
- support for research data, including large and complex data types
- gold Open Access which fosters wider collaboration and increased citations
- maximum visibility for your research: over 100M website views per year

At BMC, research is always in progress.

Learn more biomedcentral.com/submissions

BMC

For peer review only
