# Supplementary material for: Proton pump inhibitors in invasively ventilated patients with SARS-CoV-2: a substudy of the re-evaluating the inhibition of stress erosions trial
Source: BMJ Open. 2026 Aug 31;16(8):e119937. doi: 10.1136/bmjopen-2026-119937 (PMC13536124; doi:10.1136/bmjopen-2026-119937)
Supplement: Supplementary file 4 [file bmjopen-2026-119937.reviewer_comments.pdf]

# PEER REVIEW HISTORY

BMJ Open publishes all reviews undertaken for accepted manuscripts. Reviewers are asked to complete a checklist review form and are provided with free text boxes to elaborate on their assessment. These free text comments are reproduced below.

## ARTICLE DETAILS

### Title (Provisional)

Proton Pump Inhibitors in Invasively Ventilated Patients with SARS-CoV-2: A Substudy of the Re-EValuating the Inhibition of Stress Erosions (REVISE) Trial

### Authors

Dennis, Brittany; Heels-Ansdell, Diane; Quazi, Ibrahim; Basmaji, John; Thabane, Lehana; Guyatt, Gordon; Saunders, Lois; Hand, Lori; Loy, Olivia; Zytaruk, Nicole; Hardie, Miranda; Deane, Adam; Marshall, John; Arabi, Yaseen; Lauzier, François; Dionne, Joanna C.; Burns, Karen EA; Alhazzani, Waleed; Vazquez-Grande, Gloria; Geagea, Anna; Myburgh, John; Muscedere, John; English, Shane; Binnie, Alexandra; Tsang, Jennifer LY; Hunt, Miranda; Ostermann, Marlies; Al-Fares, Abdulrahman A; Knowles, Serena; Ahmed, Aijaz; Cook, Deborah J; The REVISE Investigators, The Canadian Critical Care Trials Group

---

## VERSION 1 - REVIEW

---

| | |
|---|---|
| **Reviewer** | **1** |
| **Name** | **Dack, Kyle** |
| **Affiliation** | **University of Bristol, MRC Integrative Epidemiology Unit** |
| **Date** | **16-May-2026** |
| **COI** | **None** |

---

Overall this is a nice paper, the design and methodology are appropriate and reported well. The presentation of the results is clear, and the interpretation looks reasonable. The variables could be reported in more details in the methods, to ensure alignment with STROBE. The propensity score matching could also be evaluated more formally by comparing SMD before and after matching.

Abstract:

1. If space allows it could be helpful to add the study dates to give some context of the time this analysis represents.

Methods:

2. The methods clearly describe the study design, setting, and how this subset of patients were determined. However, there is a lack of information regarding the variables of interest, and it is recommended in STROBE to specify these in the methods. Some of this information is in the Analysis section, but this is mixed with other elements of the methods and it would be clearer to have a separate section for:

-The intervention (relevant to objective 3) - e.g. dose, comparison, any important procedural details.

-Clinical outcomes - definitions, how each was measured, followup timepoints, and their status in the analysis (e.g. primary or secondary - the Analysis subheading mentioned secondary outcomes but primary were not directly specified).

-Covariates (used in PSM/multivariable regression) - Any brief information about measurement methods, units or timing to ensure accurate interpretation.

Adding this information would help readers to more accurately understand and evaluate what the results represent. This would also improve the Analysis subsection as the remaining information would be solely related to the statistical methodology.

3. PSM: The overall approach sounds reasonable but more technical details could be added to ensure it can be fully appraised and replicated. For example, the matching procedure (caliper use, replacement strategy, matching algorithm), matching diagnostics, or how balance was evaluated. Ideally, balance is often evaluated by reporting pre- and post-matching baseline descriptives for cases/controls, with the SMD before and after matching. Supplementary Table S2 shows part of this, but adding the pre-matched descriptives along with SMD for each would strengthen confidence in the matching process.

4. APACHE II was adjusted for in mortality-related models, but not other models, nor the PSM. What was the reason for this?

Results:

5. The results are clearly and accurately reported. The statistics all appear consistent. My only relevant comments relate to those already stated in the methods: expanding Supplementary Table 2 to more formally evaluate matching balance, and adding more variable details to the methods because there are some variables in the results not mentioned previously in the paper.

Discussion:

6. The discussion is reasonable. It would be informative if the authors could elaborate more on confounding - it is always possible for unmeasured confounding to exist, but in the authors judgement were there any specific factors which could be relevant? If not, this is a strength which could be stated. The limitation regarding sample size could be more directly linked to the interaction results - e.g. it could be noted that HR in Table 4 are relatively wide and this uncertainty would feed into the power to detect an interaction effect.

---


| | |
|---|---|
| **Reviewer** | **2** |
| **Name** | **Richardson, Alice** |
| **Affiliation** | **Australian National University, NCEPH** |
| **Date** | **22-Jun-2026** |
| **COI** | **None** |

Referee's report on "Proton Pump Inhibitors in Invasively Ventilated Patients with SARS-CoV-2: A Substudy of the Re-EValuating the Inhibition of Stress Erosions (REVISE) Trial"

In this paper the authors report on the results of a sub-study in the REVISE trial. The research questions are (1) what are the clinical characteristics of critically ill patients with SARS-COV-2 (2) how do the clinical outcomes of a propensity-batched non-infected cohort compare (3) does pantoprazole's treatment effect differ by SARS-COV-2 infection status.

This is a pre-planned substudy of the REVISE trial and the protocol of REVISE has been published. There is therefore a strong evidence base to proceed with publication of this paper. There are also several commendable aspects to the paper; in particular the development of a patient-important bleeding outcome in conjunction with patients and their families is great to see.

In regards to the propensity-score analysis that forms the core of the paper, I feel like several aspects of a typical propensity analysis are missing which lower the usefulness of the paper in terms of its use for health technology assessment, for instance. This is the main reason I ticked "No" for "Are the statistics described fully". Could the authors add (potentially as Supplementary Material) an indication of the distribution of variables pre- and post-matching, and the distribution of the propensity scores themselves, at the very least?

I also commend the authors on the use of the ICEMAN tool (page 7, line 47) to assess the credibility of their effect modification analyses. However it is not very clear how that plays out in the Results or the Discussion. The authors should consider including their ICEMAN checklists in the Supplementary materials; or they should clarify which pieces of the results are being presented in accordance with ICEMAN principles.

The estimates and confidence intervals in Table 3 look to be highly consistent between approaches (propensity-matching and multivariable regression) and this is mentioned in the Results. The authors should consider whether they need to say anything more in the Discussion about whether the analyses are actually doing something different with coincidentally similar results, or are the two analyses should be expected to come out similarly.

**Minor issues**

Page 4 line 50: "other infected patient may *not* have been symptomatic"?

Page 7 line 12: a matched triple rather than a matched pair, I think.

Page 7 line 27: "involving evaluating the association …" not "involved evaluating the association …". This sentence is very long and would benefit from being split into two or even tree.

Could the authors please clarify in the paper that REVISE was not originally planned to investigate COVID-19, because the trial began enrolling patients in July 2019 (page 6 line 4), before COVID-19 was heard of.

What does “peer-review funded” mean (page 6 line 11).

Reference 9 (page 20 line 35) is missing authors.

---

## VERSION 1 - AUTHOR RESPONSE

Reviewer: 1: Dr. Kyle Dack, University of Bristol
Comments to the Author: Overall this is a nice paper, the design and methodology are appropriate and reported well. The presentation of the results is clear, and the interpretation looks reasonable. The variables could be reported in more details in the methods, to ensure alignment with STROBE. The propensity score matching could also be evaluated more formally by comparing SMD before and after matching.

Abstract:
1. If space allows it could be helpful to add the study dates to give some context of the time this analysis represents.

** Thank you for this suggestion. We have added the study dates to the Abstract of this report as follows:

“Participants: From July 2019 to October 2023, 4,821 eligible participants were enrolled in REVISE whether or not they had SARS-CoV-2 infection.”

Methods:
2. The methods clearly describe the study design, setting, and how this subset of patients were determined. However, there is a lack of information regarding the variables of interest, and it is recommended in STROBE to specify these in the methods. Some of this information is in the Analysis section, but this is mixed with other elements of the methods and it would be clearer to have a separate section for:

-The intervention (relevant to objective 3) - e.g. dose, comparison, any important procedural details.

** We pulled out information about the intervention and embellished it in its own subsection as follows:

*“Trial Intervention*
Study drug (40 mg intravenous pantoprazole or matching placebo) was prescribed in the ICU until discontinuation of invasive mechanical ventilation, the occurrence of a prespecified indication or contraindication to PPI use, or death, up to 90 days.”

-Clinical outcomes - definitions, how each was measured, followup timepoints, and their status in the analysis (e.g. primary or secondary - the Analysis subheading mentioned secondary outcomes but primary were not directly specified).

** In the third paragraph of the Introduction we had indicated the primary outcomes when introducing the REVISE trial, as follows:

“This prospective substudy was pre-planned, nested within an international randomized blinded trial in critically ill patients, investigating the effect of pantoprazole 40 mg intravenously compared to placebo on the primary *efficacy* outcome of clinically important upper GI bleeding and the primary *safety* outcome of 90-day mortality.”

** However we also created a new section to refer to 4 prior publications including for the main trial, for the clinical outcomes for this substudy, how they were defined and the timing of ascertainment, and hope that is suitable.

*"Outcomes*
The clinical outcomes for REVISE and this substudy were outlined in the trial registration [NCT03374800], trial protocol[14], trial statistical analysis plan,[15] trial report,[13] and protocol for this substudy.[12]"

-Covariates (used in PSM/multivariable regression) - Any brief information about measurement methods, units or timing to ensure accurate interpretation.

** This is a great idea. We created a new subsection as follows:

*"Covariates for Matching*
The patients' age was abstracted from the medical record in years. Biological sex was abstracted from the medical record; gender was not considered. Pre-hospital acid suppression and use of corticosteroids in the week pre-randomization was documented by research personnel from the patient's medical record, clarified with a hospital or community pharmacist or substitute decision-maker if necessary. The ICU admission category of medical vs surgical/trauma was classified by research personnel based on the APACHE admission diagnostic categories. Days from the start of pandemic to enrollment was calculated from March 11, 2020."

Adding this information would help readers to more accurately understand and evaluate what the results represent. This would also improve the Analysis subsection as the remaining information would be solely related to the statistical methodology.

** Thank you for this request to have clear sections about these variables of interest. We have done so and rearranged a few paragraphs in the Methods accordingly. We also labelled the section related to COVID-19 status with the subsection named "*Characterizing Patients with COVID-19"*.

3. PSM: The overall approach sounds reasonable but more technical details could be added to ensure it can be fully appraised and replicated. For example, the matching procedure (caliper use, replacement strategy, matching algorithm), matching diagnostics, or how balance was evaluated. Ideally, balance is often evaluated by reporting pre- and post-matching baseline descriptives for cases/controls, with the SMD before and after matching. Supplementary Table S2 shows part of this, but adding the pre-matched descriptives along with SMD for each would strengthen confidence in the matching process.

** We are pleased to add more technical detail for the PSM as requested. We used optimal propensity matching at a 2:1 ratio, matching two non-COVID patients to each COVID patient. This method identified the set of matched triplets that minimized the overall difference in propensity scores across all possible matching combinations. We added this to the Analysis section:

"This method identified the set of matched triplets that minimized the overall difference in propensity scores across all possible matching combinations. We used optimal propensity matching at a 2:1 ratio, matching two non-COVID patients to each COVID patient. This method identified the set of matched triplets that minimized the overall difference in propensity scores across all possible matching combinations."

** Also, we have added a column to Table S2, the distributions of confounders in the non-COVID patients pre-matching besides post-matching column, which demonstrates that the matching achieved balance between the groups in the known confounders. We believe that this descriptive summary of the confounders adequately demonstrates covariates balance between the groups. We would be happy to provide additional balance diagnostic if you believe that is needed. We are not sure that clinical

readers will be familiar with this type of data presentation, but we retained this in the revised Supplemental Table 2.

** To recap, in revised Supplementary Table 2, we show the distribution of the confounder variables before and after propensity score matching, as well as the distribution of propensity scores for the COVID and non-COVID groups. The new sections of the table summarize the propensity score distributions using the mean, standard deviation, median (Q1, Q3), and range (minimum, maximum). There was substantial overlap in propensity scores between the two groups, enabled each COVID patient to be matched with non-COVID patients. Therefore, the new title for Supplemental Table 2 is: "Supplementary Table S2: Patient Characteristics and Propensity Scores According to SARS-CoV-2 Status in the Propensity-Matched Cohort", excerpted here:

**"Supplementary Table S2: Patient Characteristics and Propensity Scores According to SARS-CoV-2 Status in the Propensity-Matched Cohort**

| | SARS-CoV-2 Patients | Non- SARS-CoV-2 Patients | Propensity-matched Non- SARS-CoV-2 Patients (2:1) |
|---|---|---|---|
| Number of patients, n (%) | 467 | 4010 | 934 |
| Age (years), mean ± SD | 61 ± 14 | 58 ± 17 | 62 ± 13 |
| Female, n (%) | 187 (40.0) | 1439 (35.9) | 365 (39.1) |
| Prehospital acid suppression, n (%) | 104 (22.3) | 920 (22.9) | 217 (23.2) |
| ICU admission category, n (%) | | | |
| Medical | 440 (94.2) | 2819 (70.3) | 878 (94.0) |
| Surgical/trauma | 27 (5.8) | 1191 (29.7) | 56 (6.0) |
| Corticosteroids in the week pre randomization, n (%) | 362 (77.5) | 1206 (30.1) | 726 (77.7) |
| Days from start of pandemic to enrollment, mean ± SD | 669 ± 280 | 787 ± 344 | 692 ± 339 |
| Propensity Score | | | |
| mean ± SD | 0.221 ± 0.110 | 0.091 ± 0.100 | 0.219 ± 0.110 |
| median (IQR) | 0.257 (0.145-0.305) | 0.048 (0.024-0.112) | 0.245 (0.146-0.295) |
| minimum | 0.006 | 0.004 | 0.006 |
| maximum | 0.411 | 0.439 | 0.411 |

**Legend Supplementary Table S2:** This table shows the baseline characteristics of patients with SARS-CoV-2 and propensity-matched patients without SARS-CoV-2. This table also shows the propensity scores according to SARS-CoV-2 Status in the propensity-matched cohort.
SARS-CoV-2=severe acute respiratory syndrome coronavirus-2
SD=standard deviation
ICU=intensive care unit
IQR=interquartile range"

4. APACHE II was adjusted for in mortality-related models, but not other models, nor the PSM. What was the reason for this?

** Thank you for asking. The illness severity score, the APACHE II score, was designed and validated to predict risk of death among critically ill patients, hence the decision to adjust mortality

related models by the APACHE II score. This illness severity score has not been shown to predict other clinical outcomes among critically ill patients, hence we did not adjust for illness severity in the other models, nor the PSM. In this way, we also followed the analytic approach used in our main trial report. We did not modify the text in this regard.

Results:
5. The results are clearly and accurately reported. The statistics all appear consistent. My only relevant comments relate to those already stated in the methods: expanding Supplementary Table 2 to more formally evaluate matching balance, and adding more variable details to the methods because there are some variables in the results not mentioned previously in the paper.

** Thank you for asking about this refinement. We have added further details about the variables to the Analysis section, as above. Also, we have expanded Supplementary Table 2 with an additional column to more formally demonstrate matching balance, and we added additional rows in Supplementary Table 2 to show propensity scores, as fully described and excerpted below.

Discussion:
6. The discussion is reasonable. It would be informative if the authors could elaborate more on confounding - it is always possible for unmeasured confounding to exist, but in the authors judgement were there any specific factors which could be relevant? If not, this is a strength which could be stated. The limitation regarding sample size could be more directly linked to the interaction results - e.g. it could be noted that HR in Table 4 are relatively wide and this uncertainty would feed into the power to detect an interaction effect.

** We acknowledge the possibility of unmeasured confounding in any analyses such as these. One example of a possible confounder would be transplantation status. Patients who have had organ transplantation are typically maintained with immunosuppressive medications; they may have worse clinical outcomes if they become critically ill (regardless of their reason for admission to the ICU) than other patients, and they may also be more likely to develop COVID-19 in the first place. However, we did not consider this variable in the analyses as we anticipate that very few of the REVISE patients had prior organ transplantation, such that considering this variable would not influence the results. Of course we cannot be sure that there are no other unmeasured confounders, so we believe it is most conservative to acknowledge that the possibility remains; we hope it is satisfactory to retain the final sentence in the Limitations sentence as such:

"Finally, our propensity analysis could not adjust for all possible factors predisposing to SARS-CoV-2 leading to possible unrecognized confounding."

** Regarding additional statements about the sample size, we enhanced the Limitations section about the sample size, relating it to the confidence intervals around the hazard ratios more generally.

"The confidence intervals around the hazard ratios for some results are relatively wide, suggesting uncertainty about the power to detect an interaction effect if one existed."

Reviewer: 2: Dr. Alice Richardson, Australian National University
Comments to the Author: In this paper the authors report on the results of a sub-study in the REVISE trial. The research questions are (1) what are the clinical characteristics of critically ill patients with SARS-COV-2 (2) how do the clinical outcomes of a propensity-batched non-infected cohort compare (3) does pantoprazole's treatment effect differ by SARS-COV-2 infection status.

This is a pre-planned substudy of the REVISE trial and the protocol of REVISE has been published. There is therefore a strong evidence base to proceed with publication of this paper. There are also several commendable aspects to the paper; in particular the development of a patient-important bleeding outcome in conjunction with patients and their families is great to see.

** Thank you for this summary of our approach, highlighting the pre-planned, published protocol for this analysis in *Trials*.

In regards to the propensity-score analysis that forms the core of the paper, I feel like several aspects of a typical propensity analysis are missing which lower the usefulness of the paper in terms of its use for health technology assessment, for instance. This is the main reason I ticked “No” for “Are the statistics described fully”. Could the authors add (potentially as Supplementary Material) an indication of the distribution of variables pre- and post-matching, and the distribution of the propensity scores themselves, at the very least?

** ** We are pleased to add more technical detail for the PSM as requested. We used optimal propensity matching at a 2:1 ratio, matching two non-COVID patients to each COVID patient. This method identified the set of matched triplets that minimized the overall difference in propensity scores across all possible matching combinations. We have added a column to Table S2, the distributions of confounders in the non-COVID patients pre-matching besides post-matching column, which demonstrates that the matching achieved balance between the groups in the known confounders. We believe that this descriptive summary of the confounders adequately demonstrates covariates balance between the groups. We would be happy to provide additional balance diagnostics if you think that is needed. We are not sure that clinical readers will be familiar with this type of data presentation, but we retained this in the revised Supplemental Table 2.

To recap, and as summarized for Reviewer 1, in revised Supplementary Table 2, we show the distribution of the confounder variables before and after propensity score matching, as well as the distribution of propensity scores for the COVID and non-COVID groups. The new sections of the table summarize the propensity score distributions using the mean, standard deviation, median (Q1, Q3), and range (minimum, maximum). There was substantial overlap in propensity scores between the two groups, enabled each COVID patient to be matched with non-COVID patients. Therefore, the new title for Supplemental Table 2 is: “Supplementary Table S2: Patient Characteristics and Propensity Scores According to SARS-CoV-2 Status in the Propensity-Matched Cohort”, excerpted here:

**“Supplementary Table S2: Patient Characteristics and Propensity Scores According to SARS-CoV-2 Status in the Propensity-Matched Cohort**

| | SARS-CoV-2 Patients | Non- SARS-CoV-2 Patients | Propensity-matched Non- SARS-CoV-2 Patients (2:1) |
|---|---|---|---|
| Number of patients, n (%) | 467 | 4010 | 934 |
| Age (years), mean ± SD | 61 ± 14 | 58 ± 17 | 62 ± 13 |
| Female, n (%) | 187 (40.0) | 1439 (35.9) | 365 (39.1) |
| Prehospital acid suppression, n (%) | 104 (22.3) | 920 (22.9) | 217 (23.2) |
| ICU admission category, n (%) | | | |
| Medical | 440 (94.2) | 2819 (70.3) | 878 (94.0) |
| Surgical/trauma | 27 (5.8) | 1191 (29.7) | 56 (6.0) |
| Corticosteroids in the week pre randomization, n (%) | 362 (77.5) | 1206 (30.1) | 726 (77.7) |
| Days from start of pandemic to enrollment, mean ± SD | 669 ± 280 | 787 ± 344 | 692 ± 339 |
| Propensity Score | | | |

| | SARS-CoV-2 Patients | Non- SARS-CoV-2 Patients | Propensity-matched Non- SARS-CoV-2 Patients (2:1) |
|---|---|---|---|
| mean ± SD | 0.221 ± 0.110 | 0.091 ± 0.100 | 0.219 ± 0.110 |
| median (IQR) | 0.257 (0.145-0.305) | 0.048 (0.024-0.112) | 0.245 (0.146-0.295) |
| minimum | 0.006 | 0.004 | 0.006 |
| maximum | 0.411 | 0.439 | 0.411 |

**Legend Supplementary Table S2:** This table shows the baseline characteristics of patients with SARS-CoV-2 and propensity-matched patients without SARS-CoV-2. This table also shows the propensity scores according to SARS-CoV-2 Status in the propensity-matched cohort.
SARS-CoV-2=severe acute respiratory syndrome coronavirus-2
SD=standard deviation
ICU=intensive care unit
IQR=interquartile range"

I also commend the authors on the use of the ICEMAN tool (page 7, line 47) to assess the credibility of their effect modification analyses. However it is not very clear how that plays out in the Results or the Discussion. The authors should consider including their ICEMAN checklists in the Supplementary materials; or they should clarify which pieces of the results are being presented in accordance with ICEMAN principles.

** Thank you for asking about how we considered the ICEMAN criteria. Our approach for this substudy was written in the Legend for Table 4, so not very visible:

"We did not apply criteria to assess subgroup credibility because all p-values were above 0.10, according to our statistical analysis plan."

We have now repeated this message in the text as well in the Analysis section, which mirrors our analytic approach for the main REVISE trial. In the statistical analysis plan for the main REVISE trial, we had stated that we would only apply the ICEMAN criteria to subgroup findings if the p-value for the interaction term was <0.1.

"We will include an interaction term between each subgroup variable and treatment group to test for subgroup effects. For any interaction p-value below 0.10, we will conduct a formal assessment of subgroup credibility using the only structured 4-category (high to very low credibility) instrument for assessing subgroup effects developed by an international expert panel, rigorously tested by multiple stakeholders with detailed guidance for each judgment [29=ICEMAN in original REVISE SAP]."

Thus, we acted in alignment for this REVISE substudy.  The Analysis section is expanded accordingly:

"As per the REVISE trial statistical analysis plan, if any subgroup effects were identified, using the interaction term when p-values were <0.10, we planned to assess the credibility of subgroup effects, interpreted using ICEMAN.[18]"

The estimates and confidence intervals in Table 3 look to be highly consistent between approaches (propensity-matching and multivariable regression) and this is mentioned in the Results. The authors should consider whether they need to say anything more in the Discussion about whether the analyses are actually doing something different with coincidentally similar results, or are the two analyses should be expected to come out similarly.

** We considered two methods of confounders adjustment, propensity score matching at the design stage and multivariable regression model at the analysis stage. We preferred multivariable regression approach as there was no loss of data and it studied simultaneously all types of associations: outcomes and exposures; outcomes and confounders; and confounders and exposures via design matrix. A few of our outcomes were rare where propensity matching was a powerful tool for confounder adjustment. As propensity matching achieved confounder balance between the groups, we observed similar results by both approaches, as anticipated.

** As the Reviewer noted, the point estimates and confidence intervals for the propensity-matching and multivariable regression are similar. To the Discussion in the Strengths paragraph, we have added this:

"The point estimates and confidence intervals for the propensity-matching and multivariable regression analyses are similar, increasing our confidence in the inferences about these results."

Minor issues

Page 4 line 50: "other infected patient may *not* have been symptomatic"?

** Thank you for catching this error, which we corrected by adding the word 'not' in the bullet presented under the Article Summary Strengths and Limitations of this Study:

"Our definition of COVID-19 is limited to patients who received COVID-19 specific treatment; other infected patients may not have been symptomatic."

Page 7 line 12: a matched triple rather than a matched pair, I think.

** Thank you. We changed this phrase to 'triplet'.

Page 7 line 27: "involving evaluating the association …" not "involved evaluating the association …". This sentence is very long and would benefit from being split into two or even tree.

** We have broken up this long sentence for clarity, as written below:

"A second post hoc analysis to evaluate the association between COVID-19 status and ventilator-associated pneumonia was repeated after excluding patients with baseline pneumonia. This involved using both propensity-matching and multivariable regression methods. Propensity scores were re-estimated in the reduced cohort and prespecified models were refit."

Could the authors please clarify in the paper that REVISE was not originally planned to investigate COVID-19, because the trial began enrolling patients in July 2019 (page 6 line 4), before COVID-19 was heard of.

** Indeed, as the pandemic started after REVISE was underway, the REVISE trial was not originally planned to investigate COVID-19. For the clinical reasons cited in the Introduction, we planned this substudy to help understand important features of this cohort. As per your suggestion, we made this clear in the Limitations section of the Discussion, and linked it to the sentence that follows:

"The REVISE trial was not designed *a priori* to study COVID-19, as the pandemic was declared after the trial had launched. Furthermore, no pre-specified sample size calculation was possible to discern an effect modification based on SARS-CoV-2 status."

What does "peer-review funded" mean (page 6 line 11).

** This means that the protocol for this study underwent a peer review process at a granting agency to obtain external funding. This study was not funded by a pharmaceutical or device company, hence

mention of ‘peer-review’. The phrase ‘separately’ was meant to indicate that additional funding for this study was needed distinct from, and after, the funding for the REVISE trial was obtained. As there is a full paragraph on Funding and Role of the Funders at the end of the manuscript, including a grant from Physicians Services Incorporation of Ontario, we simply deleted the phrase ‘peer-review’ to state:

“This multi-center cohort design was registered [NCT05715567], funded, and guided by a protocol and statistical analysis plan.[12]”

Reference 9 (page 20 line 35) is missing authors.

** Thank you for alerting us to this. This manuscript was coauthored by three research groups as per the journal byline. We modified it to read:

“The REMAP-CAP, ACTIV-4a, and ATTACC Investigators. Therapeutic Anticoagulation with Heparin in Critically Ill Patients with Covid-19. *New England Journal of Medicine* 2021, 385(9):777-789.”

Additional Modifications:
We made some other light edits to the document without changing any content. We also expanded the Acknowledgements for this report by adding:

“We thank the patients and families participating in this trial, and all the research personnel and bedside clinicians who supported this work.”

---

## VERSION 2 - REVIEW

---

| | |
|---|---|
| **Reviewer** | **2** |
| **Name** | **Richardson, Alice** |
| **Affiliation** | **Australian National University, NCEPH** |
| **Date** | **03-Aug-2026** |
| **COI** | |

---

The authors have addressed thoroughly all the matters I raised in my report on the initial submission, thankyou.

However one more matter has come to my attention that I think the authors need to clarify. In Table 3, does the column header “Adjusted Ratio of Length of Stay/Duration (95% CI)” refer to an incident rate ratio? It’s unclear because of the slash which suggests the ratio is of duration and length of stay. This column header and the related one could be revised to “Adjusted Incident Rate Ratio of Duration/Length of Stay (95% CI)”. Furthermore the approach of simply analysing the log of the length of stay/duration then exponentiating the results in order to populate the table is old-fashioned. A more modern approach is to model the count of days in the duration/length of stay as a Poisson outcome using a generalised linear model. The authors should explain why they preferred the transformation model and

note that alternatives exist. Or they could try the alternative though I suspect that the results will be similar given their statistical strength.

I am happy to recommend that the paper undergo minor revisions to address the point above.

---

## VERSION 2 - AUTHOR RESPONSE

**Reviewer Comment:** However one more matter has come to my attention that I think the authors need to clarify. In Table 3, does the column header "Adjusted Ratio of Length of Stay/Duration (95% CI)" refer to an incident rate ratio? It's unclear because of the slash which suggests the ratio is of duration and length of stay. This column header and the related one could be revised to "Adjusted Incident Rate Ratio of Duration/Length of Stay (95% CI)". Furthermore the approach of simply analysing the log of the length of stay/duration then exponentiating the results in order to populate the table is old-fashioned. A more modern approach is to model the count of days in the duration/length of stay as a Poisson outcome using a generalised linear model. The authors should explain why they preferred the transformation model and note that alternatives exist. Or they could try the alternative though I suspect that the results will be similar given their statistical strength. I am happy to recommend that the paper undergo minor revisions to address the point above.

**Response:** Thank you for this positive assessment of our report, and this additional question about analyzing the duration of mechanical ventilation, ICU stay and hospital stay.

We appreciate the question about Table 3 and the clarification of the labels. The slash in "Adjusted Ratio of Length of Stay/Duration (95%CI)" was meant as "or" and not meant as "divided by". We have changed the column header to "Adjusted Ratio of Duration (95% CI)" to avoid this confusion.

To recap, the Analysis section of the text reads as follows (we added duration of ventilation to duration of ICU and hospital stay):

"ICU and hospital length of stay, and duration of mechanical ventilation were highly skewed to the right. Thus, natural logarithms of these three variables were regressed on COVID-19 status and *a priori* confounders and adjusted (exponentiated) relative effect of COVID-19 on length of stays were reported."

We followed our protocol and SAP published in *Trials,* ensuring that analyses aligned with the study objectives, promoting reproducibility. This was referenced in our submission (this is stated for objective 3 "For continuous outcomes, we will perform linear regression which will include an interaction term. We will report mean differences with 95% CIs. If needed, we will perform the linear regression on the log-transformed outcome."

While we acknowledge that other approaches exist, the data were overdispersed, violated a Poisson assumption, mean=variance. In the data, variances were significantly higher than means. Also, the log transformation of length of stay variables were normal or approximately normal. Thus, normality assumptions for linear regression were met. We hope that this information is acceptable, added to the rationale of following our prespecified plan to preserve research integrity and transparency.

We thank you for such a detailed assessment of this manuscript and for the thoughtful, constructive feedback provided. We appreciate your consideration of this manuscript in *BMJ Open.*
